# Structural Analysis of Selenium Coordination Compounds and Mesoporous TiO_2_-Based Photocatalysts for Hydrogen Generation

**DOI:** 10.1021/acs.inorgchem.4c05325

**Published:** 2025-04-16

**Authors:** Rodrigo Cervo, Cândida Alíssia Brandl, Tanize Bortolotto, Camila Nunes Cechin, Natália de Freitas Daudt, Bernardo Almeida Iglesias, Ernesto Schulz Lang, Bárbara Tirloni, Roberta Cargnelutti

**Affiliations:** † Department of Chemistry, Federal University of Santa Maria (UFSM), # Av. Roraima, n.1000, 97105-900 Santa Maria, RS, Brazil; ‡ Department of Mechanical Engineering, Federal University of Santa Maria (UFSM), # Av. Roraima, n.1000, 97105-900 Santa Maria, RS, Brazil

## Abstract

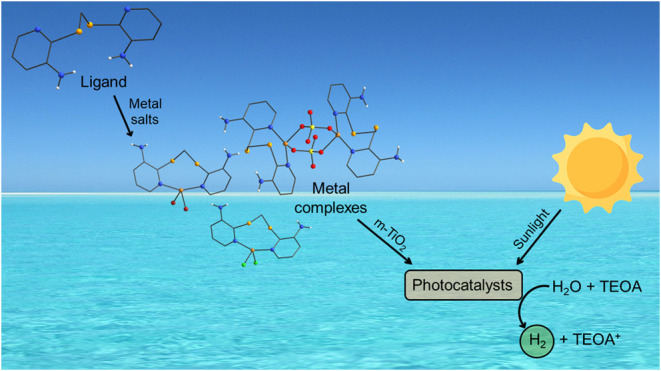

This study reports the synthesis of ten coordination compounds (**1**–**10**) derived from the ligand bis((3-aminopyridin-2-yl)selanyl)methane (**L**) and different metal centers (Co^II^, Cu^I^, Cu^II^, Zn^II^, and Ag^I^). Single crystals of the complexes were obtained via slow diffusion from overlaid solutions of ligand **L** and the corresponding metal. Their crystalline structures were determined by single-crystal X-ray diffraction (SCXRD) and further characterized using spectroscopic, spectrometric, and voltammetric techniques. Complexes **1**–**5**, **7**, and **10** were evaluated as cocatalysts of mesoporous titanium dioxide (m-TiO_2_) for photocatalytic hydrogen production via water photolysis under solar light simulation, using triethanolamine (TEOA) as the sacrificial agent. The results showed that complexes **4**, **5**, **7**, and **10** enhanced m-TiO_2_ photocatalytic activity, achieving hydrogen evolution rates at least four times higher than standard m-TiO_2_ and P25. Among these, the photocatalyst m-TiO_2_-**7** (**7** = [Cu_2_(μ-SO_4_)_2_**L**_**2**_]) exhibited the highest hydrogen production, reaching approximately 7800 μmol/g over a 6-h experiment–nearly 26 times greater than pure m-TiO_2_ (300 μmol/g). These findings highlight the potential of organoselenium metal complexes for the development of novel photocatalytic materials based on nonprecious metals.

## Abstract 2

New organoselenium nonprecious metal complexes were synthesized and used as cocatalysts for mesoporous titanium dioxide (m-TiO_2_) in hydrogen gas (H_2_) production via water photolysis under simulated solar light, using triethanolamine (TEOA) as the sacrificial agent. The results demonstrated remarkable catalytic activity, yielding 26 times more H_2_ than pure m-TiO_2_ for the photocatalyst m-TiO_2_-**7**.

## Introduction

The search for clean and renewable energy sources is one of the main focuses of study in the current era, as the excessive use of fossil fuels has been causing negative environmental impacts.^1^ The constant emission of pollutants from their combustion is a source of concern, as it is associated with the alarming climate changes the planet has been experiencing in recent times. Additionally, their extensive use makes them increasingly scarce.^1^

Therefore, it is essential to develop new energy sources that are clean, renewable, and capable of meeting current demand. In this context, the use of solar light is one of the most relevant alternatives under study today. Being abundant, inexhaustible, and globally distributed, solar radiation is one of the most attractive means of producing renewable energy, aiming to replace fossil fuels. In addition to its conversion into electrical energy through photovoltaic cells, it can also induce photochemical reactions associated with the formation of clean fuels.^1a,1b^

One of the most important fuels in the study of new sustainable energy sources, which can be obtained with the help of solar light, is hydrogen gas (H_2_(g)). Hydrogen generation is a globally relevant research area, as its combustion releases high amounts of energy while producing only water as a byproduct.^1^ Furthermore, it is a key fuel for green energy conversion in fuel cells.^2^

In 1972, Fujishima and Honda pioneered H_2_(g) production through water splitting by heterogeneous photocatalysis, using a photoelectrochemical cell with TiO_2_ under ultraviolet (UV) radiation.^2^ Since then, extensive research has focused on developing and improving the photocatalytic process of H_2_(g) production from water decomposition. Many materials have been investigated as semiconductors in this process, especially metal oxides and sulfides.^1^ Moreover, TiO_2_ remains a widely studied material due to its low cost, low toxicity, corrosion resistance, and high durability.^1^ However, several challenges in its application remain unresolved.

Although TiO_2_ is a good semiconductor, its practical applications are limited considering its activation by absorption of solar light radiation, since it is primarily UV active due to its large energy gap (3.2 eV for anatase and 3.0 eV for rutile).^1a,1d,1g^ In an industrial photocatalytic process, this is not ideal, as it would require artificial UV light. Solar light cannot be efficiently utilized in pure TiO_2_ since only about 4% of solar radiation is UV, while 46% falls within the visible range.^1a,1d^ Additionally, TiO_2_ has a high rate of electron–hole (e^–^/h^+^) recombination, which halts the photocatalytic effect after a short period of irradiation, reducing *H*_2_(*g*) production.^1d^

Numerous strategies have been explored to overcome the limitations of TiO_2_ and enhance its photocatalytic efficiency, particularly by improving its utilization of visible light in solar radiation. One approach involves the use of sacrificial agents, which act as electron donors in the photocatalytic process, as well as the combination of TiO_2_ with other materials capable of absorbing visible light.^1^ A common method to enhance its photocatalytic activity is the deposition of precious metals (e.g., Nb, Ru, Pd, Pt, and Au) on TiO_2_.^3^ However, the high cost and scarcity of these metals pose significant challenges for large-scale applications. Consequently, there is an increasing need to explore alternatives based on nonprecious metals.

Another notable approach to improving the visible light absorption capacity of TiO_2_ is the use of metal complexes as cocatalysts.^1e^ Furthermore, metal complexes have well-established photosensitivity and have been extensively studied in photochemistry.^1e^ Therefore, the use of coordination compounds as cocatalysts represents an effective way to expanding the photoresponse of TiO_2_ during visible-light-driven water photolysis.^1e,1f,1b^

**Figure 1:**
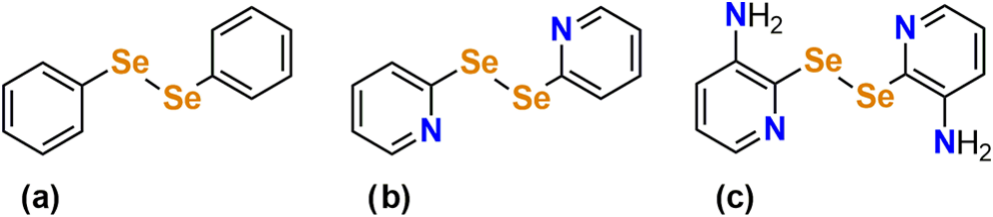
Molecular structures of some diselanes: (a) 1,2-diphenyldiselane, (b) 1,2-bis(pyridin-2-yl)diselane (**(pySe)**_**2**_), (c) 1,2-bis(3-aminopyridin-2-yl)diselane (**(3-apySe)**_**2**_).

Considering the development of new coordination compounds with potential applications as cocatalysts for photocatalytic H_2_(g) production,^4a^ many studies in the literature focus on the chemical synthesis of diselane-type molecules, also known as organic diselenides.^4^ Diselanes are highly significant in the chemistry of organic selenium compounds, both for their pharmacological properties and their broad synthetic importance.^4b−4d^ Organoselenium compounds, particularly diselane derivatives, have gained attention in coordination chemistry due to their ability to form stable complexes with transition metals.^4^ One of the most well-known molecules in this class is 1,2-diphenyldiselane (Figure 1a), which is widely used in the synthesis of new organoselenium compounds and in the study of biological systems.^4e−4j^

In coordination chemistry, pyridinic diselanes stand out, as this type of ligand contains soft (Se) and intermediate (pyridinic N) donor atoms, allowing for diverse coordination modes that can influence catalytic behavior.^5a,5b^ One of the best-known examples of this class is 1,2-bis(pyridin-2-yl)diselane (**(pySe)**_**2**_) (Figure 1b), which has been widely studied in various syntheses and applications reported in the literature.^5^ Another example receiving increasing attention in current research is 1,2-bis(3-aminopyridin-2-yl)diselane (**(3-apySe)**_**2**_) (Figure 1c).^5c^ Thus, complexes involving pyridinic diselanes and suitable metal centers hold promise for various applications, including their potential use in photocatalysis.^4k,5c^ Despite the promising properties of organoselenium coordination compounds, their application in photocatalytic hydrogen evolution remains largely unexplored, and their combination with TiO_2_ has yet to be fully investigated.

Given the main challenges in the current research landscape, our work focuses on developing efficient TiO_2_-based photocatalysts derived from organoselenium metal complexes of nonprecious metals. Specifically, we report the synthesis and structural analysis of the first coordination compounds derived from the novel ligand bis((3-aminopyridin-2-yl)selanyl)methane (**L**) and investigate their potential application as cocatalysts for mesoporous titanium dioxide (m-TiO_2_) in *H*_2_(*g*) production via water photolysis under solar light simulation, using triethanolamine (TEOA) as the sacrificial agent.

## Experimental Section

### Safety Statement: No Uncommon Hazards are Noted

#### Materials and Methods

All solvents and reagents were used as received from the suppliers (Synth or Sigma-Aldrich) without further purification. A detailed description of all characterization methods employed in this work, as well as the synthesis procedures–including the starting material **(3-apySe)**_**2**_, ligand **L**, complexes **1**–**10**, and m-TiO_2_–and the photocatalysis process (photocatalytic system, hydrogen production protocol, and impregnation process of m-TiO_2_), is provided in the Supporting Information (SI). The ligand and complexes were characterized, when applicable, by single-crystal X-ray diffraction (SCXRD), powder X-ray diffraction (PXRD), vibrational spectroscopy–including Fourier-transform infrared (FT-IR) and confocal Raman–nuclear magnetic resonance spectroscopy (NMR) (^1^H, ^13^C, ^77^Se, COSY, HSQC, and HMBC), ultraviolet–visible spectroscopy (UV–vis) in solution, diffuse reflectance spectroscopy (DRS) in the solid state, high-resolution mass spectrometry (HRMS), cyclic voltammetry (CV), elemental analysis (EA), and melting point (m.p.) determination. The surface area and average pore size of m-TiO_2_ were determined using the BET method, while its average pore volume was assessed by the BJH method. The m-TiO_2_ and photocatalysts were characterized using spectroscopic methods (FT-IR, confocal Raman, and DRS), PXRD, scanning electron microscopy (SEM), energy-dispersive X-ray spectroscopy (EDS), and solid-state photoluminescence (PL) measurements, the latter specifically for **L**, **7**, m-TiO_2_, and m-TiO_2_-**7**.

## Results and Discussion

**Scheme 1:**
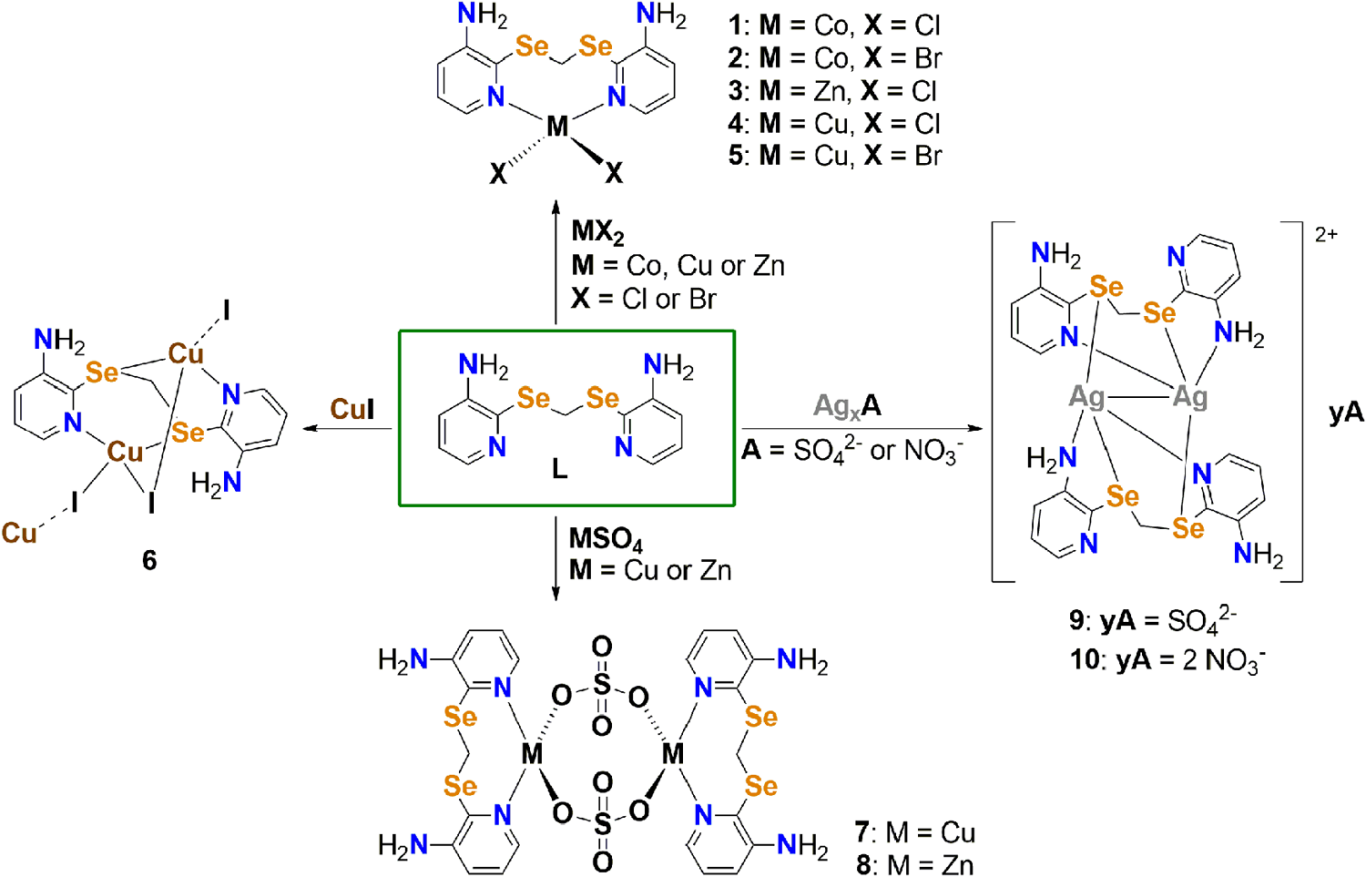
Representation of Complexes **1**–**10**, Derived from Ligand **L** and Their Respective Metal Salts

The ligand bis((3-aminopyridin-2-yl)selanyl)methane (**L**), described in this work, derived from **(3-apySe)**_**2**_, exhibits significant complexing potential, and a wide variety of its coordination compounds remains to be explored. Given the versatility and structural diversity of **(pySe)**_**2**_ derivatives already reported in the literature, particularly **(3-apySe)**_**2**_, ten new complexes containing ligand **L** were synthesized and are represented in Scheme 1. Among these, six compounds are derived from metal halides (**1–6**), three from metal sulfates (**7–9**), and one from silver nitrate (**10**). All compounds were obtained as single crystals through slow diffusion from overlaid solutions of ligand **L** and the corresponding metal salt. The experimental procedures for obtaining complexes **1–10** are described in the SI (Synthesis Procedures).

### Single-Crystal X-ray Diffraction (SCXRD)

The ligand **L** and all the complexes **1–10** were characterized by SCXRD. Selected bond lengths and angles, along with all structural projections of complexes **1–10** with anisotropic displacement parameters, are presented in the SI (Tables S7–S9 and Figures S2–S7).

According to the crystallographic data obtained for complexes **1–10**, the coordination tendency adopted by **L** became evident, as described by Pearson’s hard and soft acids and bases theory (HSAB). It was observed that atoms responsible for donating electronic density of intermediate nature (pyridinic N) showed a preference for hard cations (Co^II^, Cu^II^, and Zn^II^). On the other hand, atoms donating electronic density of soft nature (Se) favored coordination with soft metal centers (Cu^I^ and Ag^I^). Therefore, the ligand **L** acted in a bidentate manner in complexes **1–5**, **7**, and **8**, coordinating to the metals through both pyridinic nitrogen atoms. Additionally, selenium atoms played a crucial role in the formation of the structures of compounds **6**, **9**, and **10**.

For all complexes (except **9** and **10**), the metal coordination sphere was satisfied by the respective anions from the metal salts used, specifically halides for **1–6** and sulfates for **7** and **8**. In the case of **9** and **10**, the sulfate and nitrate anions acted only as counterions, since the strong coordination of **L** to the Ag^I^ centers fully satisfied their coordination sphere.

**Figure 2:**
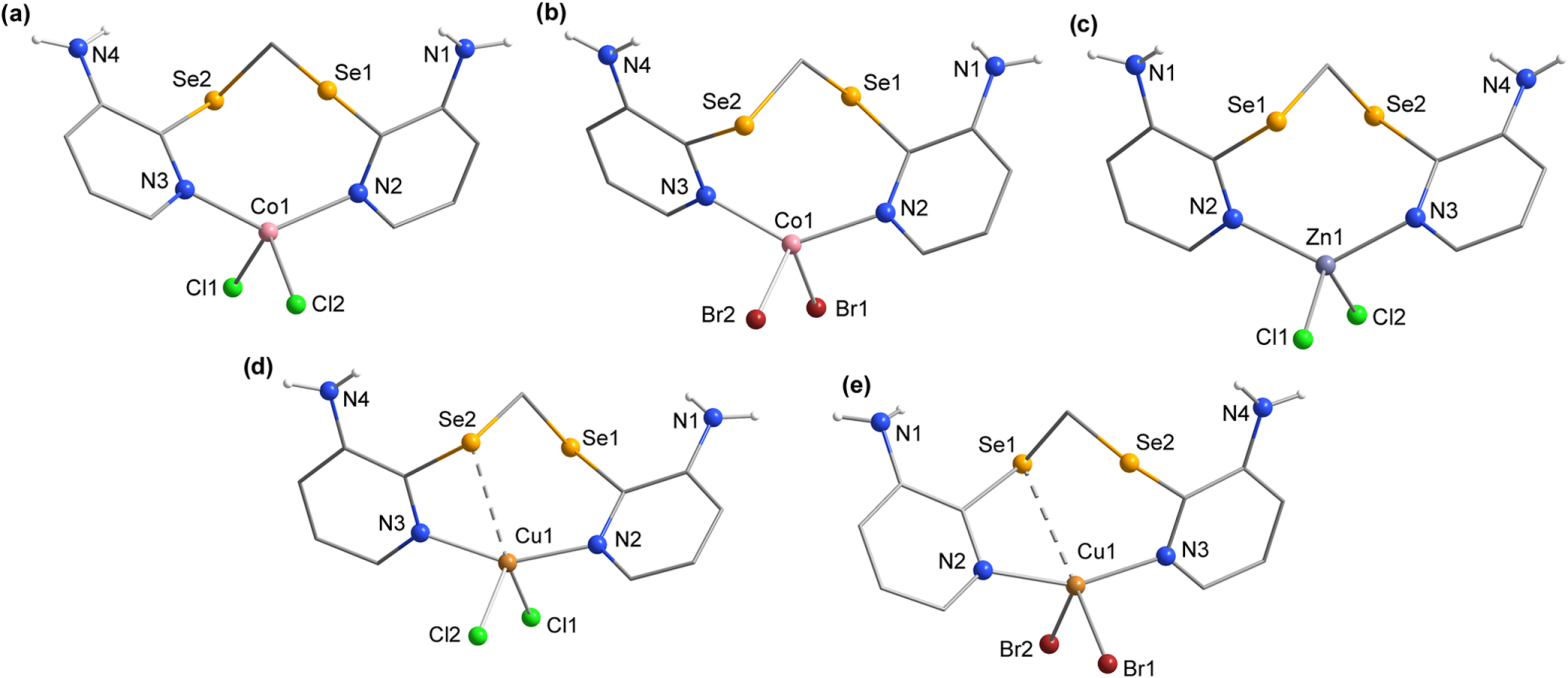
Structural projections of the complexes: (a) **1**, (b) **2**, (c) **3**, (d) **4**, (e) **5**. Aromatic hydrogen atoms and CHCl_3_ molecules have been omitted for clarity.

The complexes derived from Co^II^ (**1** and **2**), Zn^II^ (**3**), and Cu^II^ (**4** and **5**) halides are shown in Figure 2. Complexes **4** and **5** crystallized with one molecule of CHCl_3_ and half a molecule of CHCl_3_, respectively (Figure S3). Additionally, for these five complexes (except for **4**), their asymmetric units contain two independent structures of the respective compound, with the same coordination environment (Figures S2–S3). The main difference between complexes **1–5** is the presence of Se···Cu secondary bonds in **4** and **5** (dashed lines).

The coordination mode adopted by ligand **L** in these complexes (bidentate, through the pyridinic nitrogens) using their respective metal halides is well established in the literature for molecules of this nature. For example, the complexes derived from metal halides reported by Dias et al.,^6^ using **(3-apySe)**_**2**_ as a ligand, exhibit a similar coordination mode, as do the complexes described by Cervo et al., which are derived from a selane-type ligand (3-amine-2-((pyridin-2-ylmethyl)selanyl)pyridine).^6^

**Figure 3:**
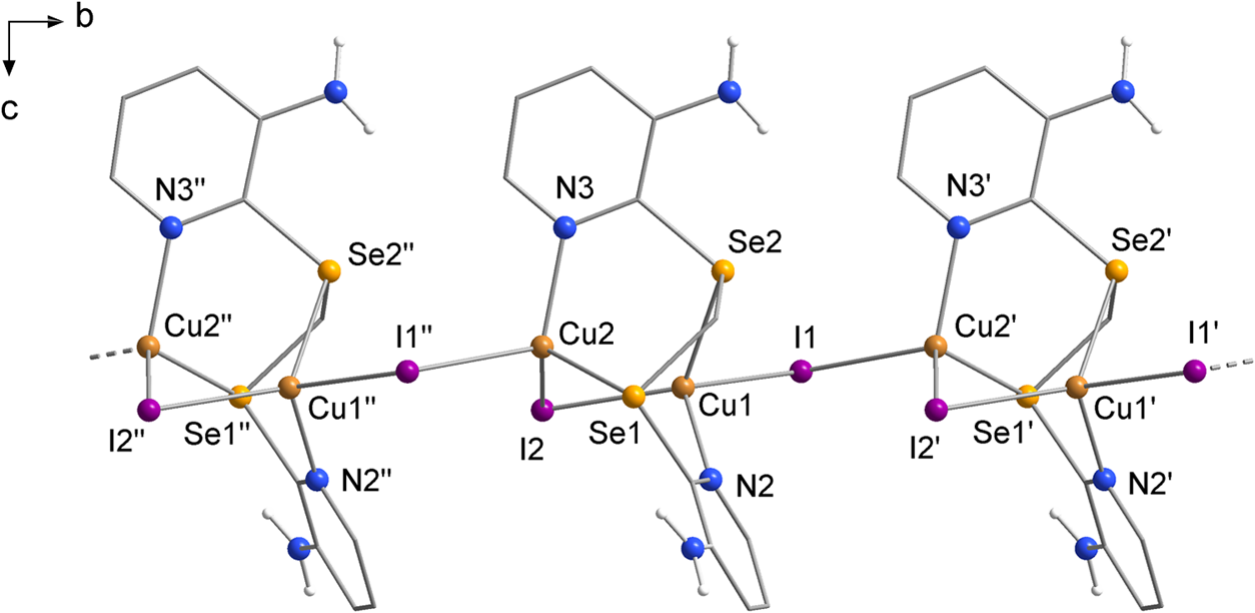
Structural projection of the polymeric complex **6**, with growth along the crystallographic *b* axis. Some hydrogen atoms have been omitted for clarity. Symmetry operations: (′) = (3/2 – *x*, 1/2 + *y*, *z*) and (″) = (3/2 – *x*, – 1/2 + *y*, *z*).

Complex **6**, derived from Cu^I^, is the only coordination polymer among the ten isolated examples, and its polymeric structural projection is shown in Figure 3. The ligand coordinates two different Cu^I^ centers through a pyridinic nitrogen atom and a selenium atom. The coordination spheres of the metals, as well as their oxidation states, are satisfied by two bridging iodide anions (μ-I^–^). One of the iodides (I2) forms an intramolecular bridge between the two Cu^I^ centers, while the other iodide (I1) bridges to a copper from another **L** unit, promoting the one-dimensional polymer growth of the structure along the *b* crystallographic axis.

No complexes similar to product **6** were found in the literature, considering the coordination mode adopted by **L**. The structures reported by Cargnelutti et al. are the closest described so far.^7^ The compound [Cu_4_I_2_(L3)_2_] (L3 = bis(pyridin-2-ylselanyl)methane)^7a^ is a tetramer of Cu^I^ with two units of ligand L3, μ-I^–^, and μ_3_-I^–^, where only one selenium atom from each L3 unit participates in the complexation process. Similarly, in the polymeric complex [Cu_2_I_2_(L4)]_*n*_ (L4 = 2-((pyridin-2-ylmethyl)selanyl)pyridine),^7b^ the coordination mode is comparable to that of **6**, although this ligand is a selane. Its polymer growth is also assisted by μ-I^–^ and μ_3_-I^–^. In all three cases, the tendency for iodide bridges to form is evident.

**Figure 4:**
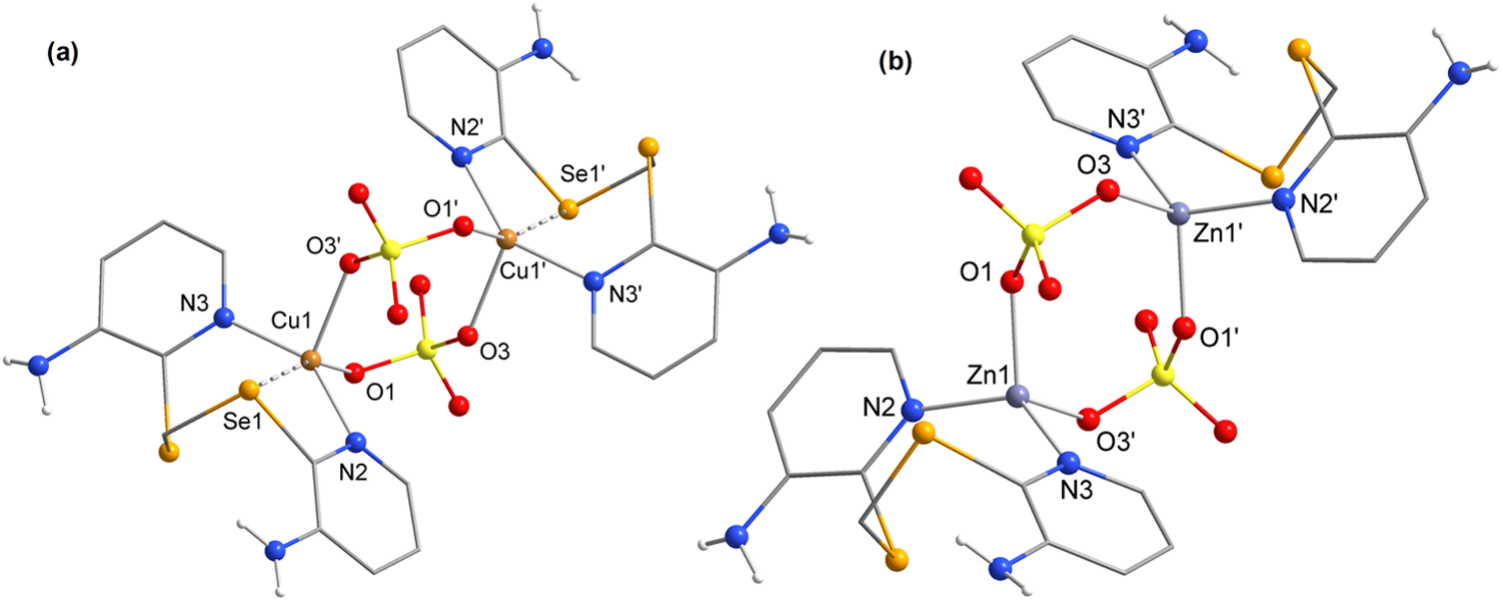
Structural projections of the complexes (a) **7** and (b) **8**. Aromatic hydrogen atoms have been omitted for clarity. Symmetry operations: **7** (′) = (1 – *x*, 1 – *y*, 2 – *z*), **8** (′) = (1 – *x*, 2 – *y*, 1 – *z*).

In complexes **7** and **8**, derived from Cu^II^ and Zn^II^ sulfates, respectively, the ligand **L** adopts a coordination mode similar to that in **1–5** (bidentate, through the pyridinic nitrogens). Both complexes are dimeric, and their structural projections are shown in Figure 4. Two **L** molecules coordinate with two metal centers (Cu^II^ for **7** and Zn^II^ for **8**), with both metals having their coordination spheres satisfied by oxygen atoms from two bridging sulfate ions (μ-SO_4_^2–^). Furthermore, the main structural difference between **7** and **8** is the presence of secondary Se···Cu bonds in **7**.

**Figure 5:**
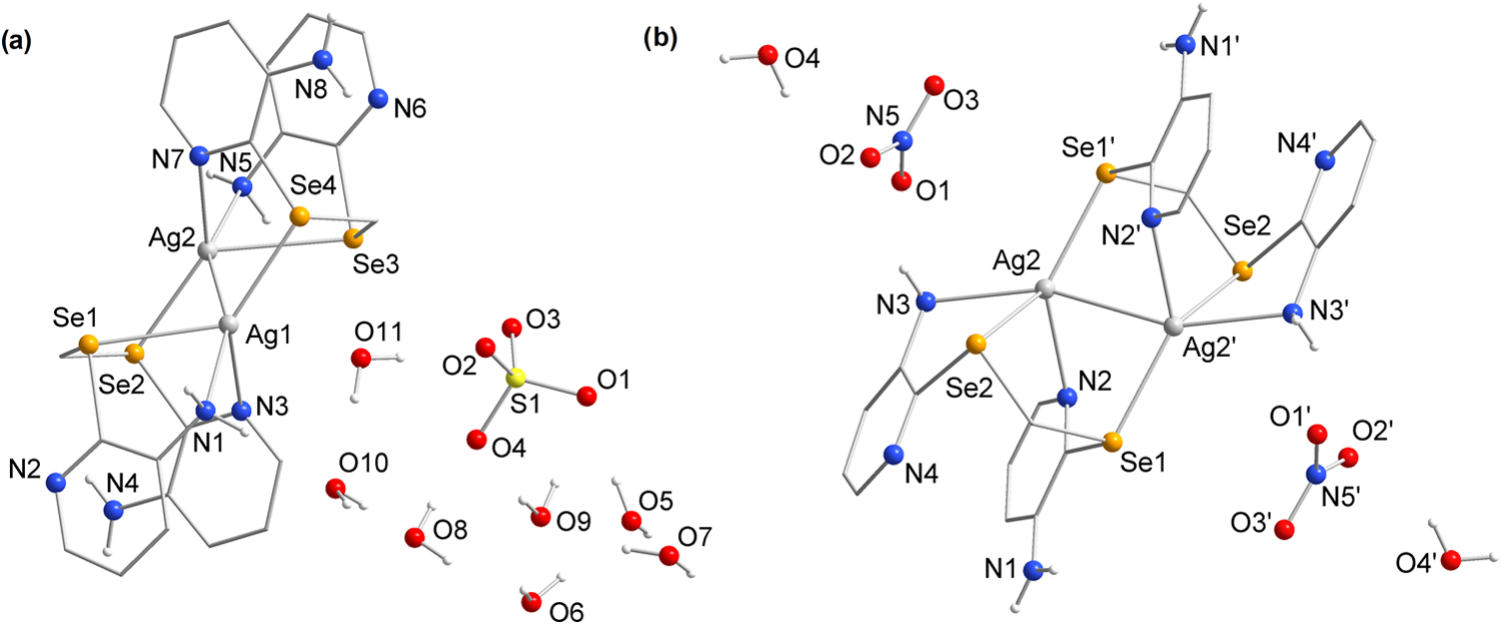
Structural projections of the cluster complexes (a) **9** and (b) **10**. Aromatic hydrogen atoms have been omitted for clarity. Symmetry operation: (′) = (−*x*, 1 – *y*, 1 – *z*).

The cluster complexes **9** and **10**, derived from Ag^I^ salts, are structurally very similar, and their projections are shown in Figure 5. Both consist of the same cationic core, formed by two equivalents of **L** and two equivalents of Ag^I^. Additionally, they contain nearby anionic units (counterions originating from the respective salts used) and water molecules as crystallization solvents.

Another important point is that compound **9** is only partially stable in its crystalline form, as determined by SCXRD, since partial loss of crystallinity occurs if the crystals are not handled at low temperatures. Elemental analysis (EA) results were more consistent with the tetrahydrated form of the compound, indicating the loss of three water molecules from its structure. Compound **10**, on the other hand, did not undergo any dehydration process and showed EA values consistent with its measured structure (the EA values of the complexes are provided in Table S6).

In the structures of cluster complexes **9** and **10**, each Ag^I^ center is coordinated by two nitrogen (N) atoms and two selenium (Se) atoms, in addition to an Ag–Ag bond of 2.9315(5) Å for **9** and 2.9240(7) Å for **10**. In each Ag^I^ unit, one N atom is pyridinic, and the other is an amine N, while one Se atom comes from an **L** unit with three coordinated sites (e.g., N1, N3, and Se1 coordinated to Ag1 in complex **9**), and the other from an **L** unit with a single coordinated site (e.g., Se4 and Ag1 in **9**).

Using the mathematical parameters **τ**_**4**_ and **τ**_**5**_ described in the literature,^8^ the geometries of the metal centers in complexes **1–10** were defined. All tetracoordinated complexes (**1**, **2**, **3**, **6**, **8**) adopt distorted trigonal pyramidal geometries, while the pentacoordinated complexes (**4**, **5**, **7**, **9**, **10**) exhibit distorted square pyramidal geometries. The polyhedral representations, as well as the **τ** parameter values, can be found in the SI (Table S10 and Figure S8).

### Powder X-ray Diffraction (PXRD)

In parallel with the structural determination of ligand **L** and complexes **1–10** by SCXRD, they were also analyzed by PXRD. In general, the PXRD diffractograms are consistent with the theoretical diffractograms generated from SCXRD data (Figures S9–S18).

**Figure 6:**
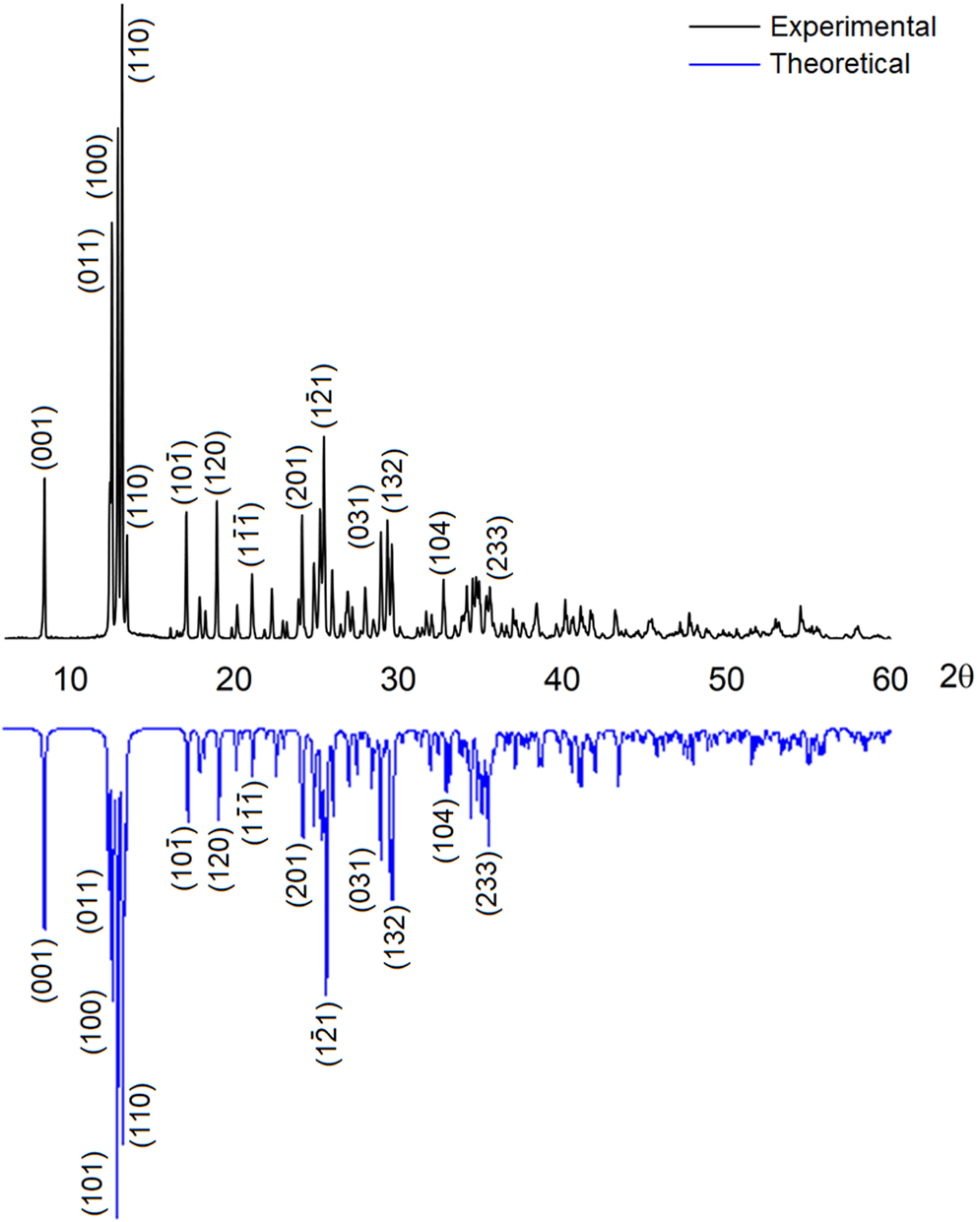
Comparison of the theoretical and experimental powder diffractograms of complex **8**.

PXRD analysis was particularly crucial for complex **8**, as the product was isolated in precipitate form, and its crystalline structure was elucidated from trace amounts of the compound obtained via slow diffusion reactions. Figure 6 shows the comparison between the theoretical and experimental diffractograms of compound **8**, along with the indexing of the main crystallographic planes. These results confirm that the complex retains the same structure in both its monocrystalline and precipitate forms.

The average crystallite sizes (in nm) of ligand **L** and complexes **1–10** were calculated using Scherrer’s equation and are listed in Table 1. The broadening of peaks observed in the diffractogram of complex **4** (Figure S13) may be related to its smaller average crystallite size, as Scherrer’s equation establishes an inverse relationship between crystallite size and peak width.^9^

**Table 1:** Average Crystallite Sizes of Ligand **L** and Complexes **1–10**

compound	average size (nm)	compound	average size (nm)
**L**	69.09		
**1**	48.87	**6**	70.73
**2**	47.49	**7**	63.14
**3**	71.88	**8**	67.84
**4**	30.03	**9**	87.52
**5**	89.69	**10**	68.85

### Fourier-Transform Infrared (FT-IR) and Confocal Raman Spectroscopy

Vibrational spectroscopy (FT-IR and confocal Raman) provided information on the absorptions associated with the bonds formed upon metal complexation, along with other spectral evidence, which were key factors in evaluating the structures of ligand **L** and complexes **1–10**. All assignments were made based on literature data,^10^ as well as comparisons between the spectra. All FT-IR and Raman spectra, along with tables containing the main assigned bands, are provided in the SI (Tables S11–S12 and Figures S19–S42).

In addition to the characteristic NH_2_ vibrations of 3389 cm^–1^ for ν_a_(NH_2_) (asymmetric stretching), 3295 cm^–1^ for ν_s_(NH_2_) (symmetric stretching), and 3175 cm^–1^ for δ_ot_(NH_2_) (overtone bending band), other important structural aspects of ligand **L** are evident in the FT-IR analyses. The typical spectral pattern of three bands, for 2,3-disubstituted pyridines^9^ can be observed in the spectrum of **L** between 1910 and 1798 cm^–1^, and between 1923 and 1778 cm^–1^ for the **(3-apySe)**_**2**_ (Figures S19–S20).

**Figure 7:**
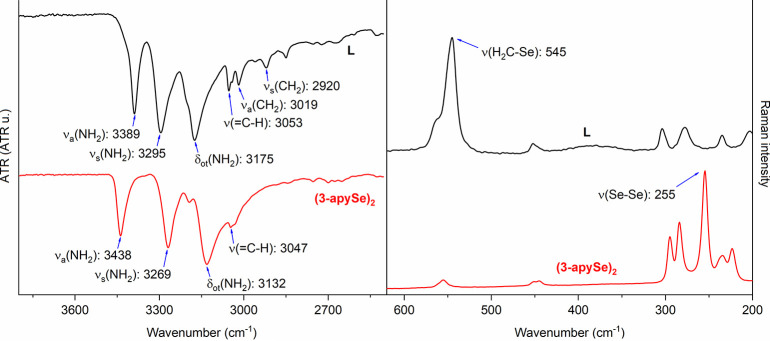
Comparison of the FT-IR (left) and Raman (right) spectra of ligand **L** and diselane **(3-apySe)**_**2**_, with emphasis on the regions of 3000 and 500 cm^–1^.

Considering the methylene carbon as the main structural difference between **L** and the **(3-apySe)**_**2**_, Figure 7 (left) shows the comparison between the two spectra, emphasizing on the main NH_2_ vibrations and aromatic hydrogens stretchings of both molecules (3053 cm^–1^ for **L** and 3047 cm^–1^ for **(3-apySe)**_**2**_). Additionally, the asymmetric and symmetric CH_2_ stretchings (3019 and 2920 cm^–1^) are present only in the structure of **L**. Complementarily, Raman spectroscopy was important for the assignment of the H_2_C–Se stretching (545 cm^–1^) present in **L**, alternating with the Se–Se stretching (255 cm^–1^) of the starting material **(3-apySe)**_**2**_ (Figure 7 right).

In all cases, the variation of the wavenumber of the main vibrational modes in the complexes is evident when compared to the free ligand. Specifically, the ν_a_(NH_2_) and ν_s_(NH_2_) have values of 3389 and 3295 cm^–1^ in **L**, respectively, as well as the δ_ot_(NH_2_) at 3175 cm^–1^. In the complexes, these wavenumbers range from 3451 to 3368 cm^–1^ for the ν_a_(NH_2_), from 3329 to 3255 cm^–1^ for the ν_s_(NH_2_), and from 3223 to 3159 cm^–1^ for the δ_ot_(NH_2_). Complexes **9** and **10** have higher wavenumbers for the three main NH_2_ vibrational modes compared to **L**. This is due to the participation of the NH_2_ group in the complexation of the silver centers, resulting in an increase of 36 to 29 cm^–1^ for the ν_a_(NH_2_) and ν_s_(NH_2_), and an increase of 45 to 40 cm^–1^ for the δ_ot_(NH_2_). Additionally, the NH_2_ overtone bending bands usually have higher values in the complexes than in the free ligand.

Furthermore, it was observed that the wavenumbers of the C=N stretching (1621 cm^–1^ in **L**) tend to vary, being lower in complexes **1–6** (ranging from 1623 to 1596 cm^–1^), and higher in analogs **7–10** (ranging from 1640 to 1620 cm^–1^). Additionally, some variations in other vibrations are observed. For example, the stretchings ν_a_(CH_2_) and ν_s_(CH_2_) (3019 and 2920 cm^–1^ for **L**) can range from 3072 to 2947 cm^–1^ and from 2999 to 2927 cm^–1^ in the complexes, respectively. The aromatic hydrogens vibrations, such as ν_s_(=C–H) (3053 cm^–1^) and δ(=C–H) (789 cm^–1^) for **L**, range from 3103 to 3023 cm^–1^ and 813 to 790 cm^–1^ in the complexes. More details are described in Table S11–S12.

**Figure 8:**
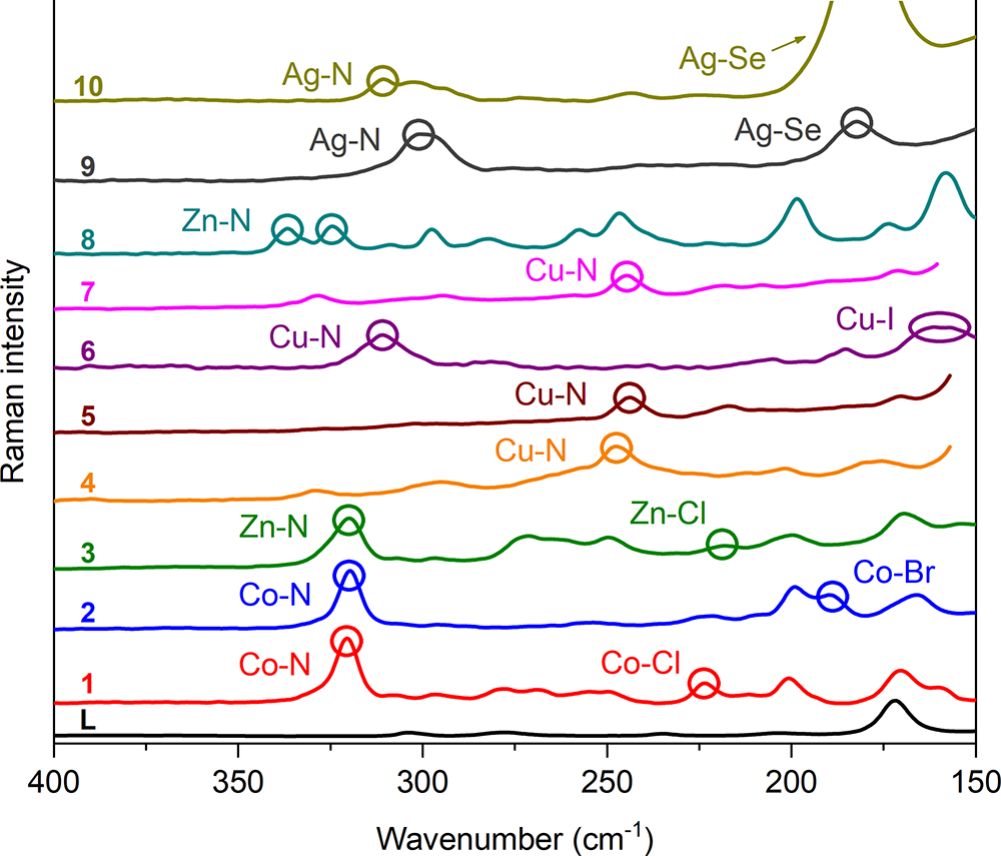
Comparison of the Raman spectra of ligand **L** and complexes **1–10**, highlighting the M–N, M–X, and Ag–Se stretching modes. For scaling reasons, the peak for complex **10** has been clipped.

The absorptions attributed to the M–N stretching modes (M = Co, Cu, Zn, Ag), in the approximate range of 340 to 230 cm^–1^, are typical indicators of ligand **L** complexation in complexes **1–10**. Additionally, absorptions related to the M–X bonds (X = Cl, Br, I) for **1–6**, in the range of 220 to 150 cm^–1^, and the Ag–Se stretching for **9** and **10**, at 186 and 173 cm^–1^, are observed. Figure 8 presents a comparison of the Raman spectra, emphasizing these characteristic stretching vibration bands involving the metal centers.

**Figure 9:**
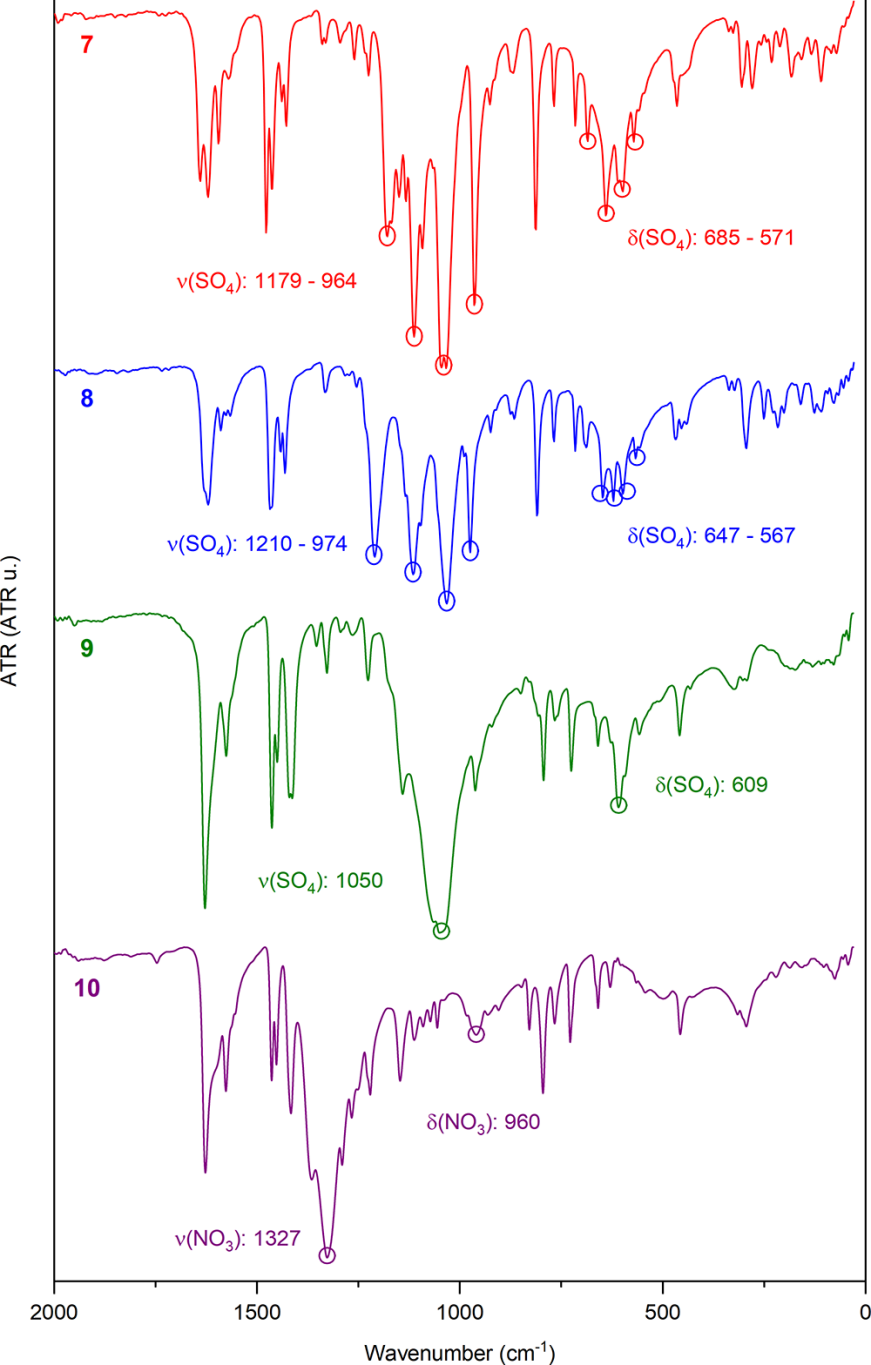
Comparison of the FT-IR spectra of ligand **L** and complexes **7**–**10**, highlighting the characteristic bands of sulfate and nitrate anions.

Complexes **7–9** exhibit typical bands corresponding to the sulfate anion, while complex **10** shows characteristic bands of nitrate, as depicted in Figure 9. Additionally, the FT-IR spectra provide detailed information about the coordination modes adopted by the anions in each structure.

According to the literature, the sulfate ion exhibits characteristic absorptions in two regions of the FT-IR spectrum: very intense absorptions in the range of 1200 to 950 cm^–1^, corresponding to the stretching vibration bands, and less intense absorptions in the range of 650 to 550 cm^–1^, corresponding to the bending bands. Additionally, the number of bands observed in each region varies depending on the symmetry of the sulfate ion, which is influenced by its coordination mode.^10a,10b^ Uncoordinated sulfate ions, which act solely as counterions, have high symmetry (*T*_d_) (**9**). As different forms of complexation occur, their symmetry decreases, and more vibrational modes become observable. In contrast, bridged coordinated sulfate presents four characteristic stretching bands and four bending bands (**7** and **8**). Similarly, nitrate acting as a counterion with greater symmetry (*D*_3_h) can be observed in complex **10**.

### Nuclear Magnetic Resonance (NMR)

**Figure 10:**
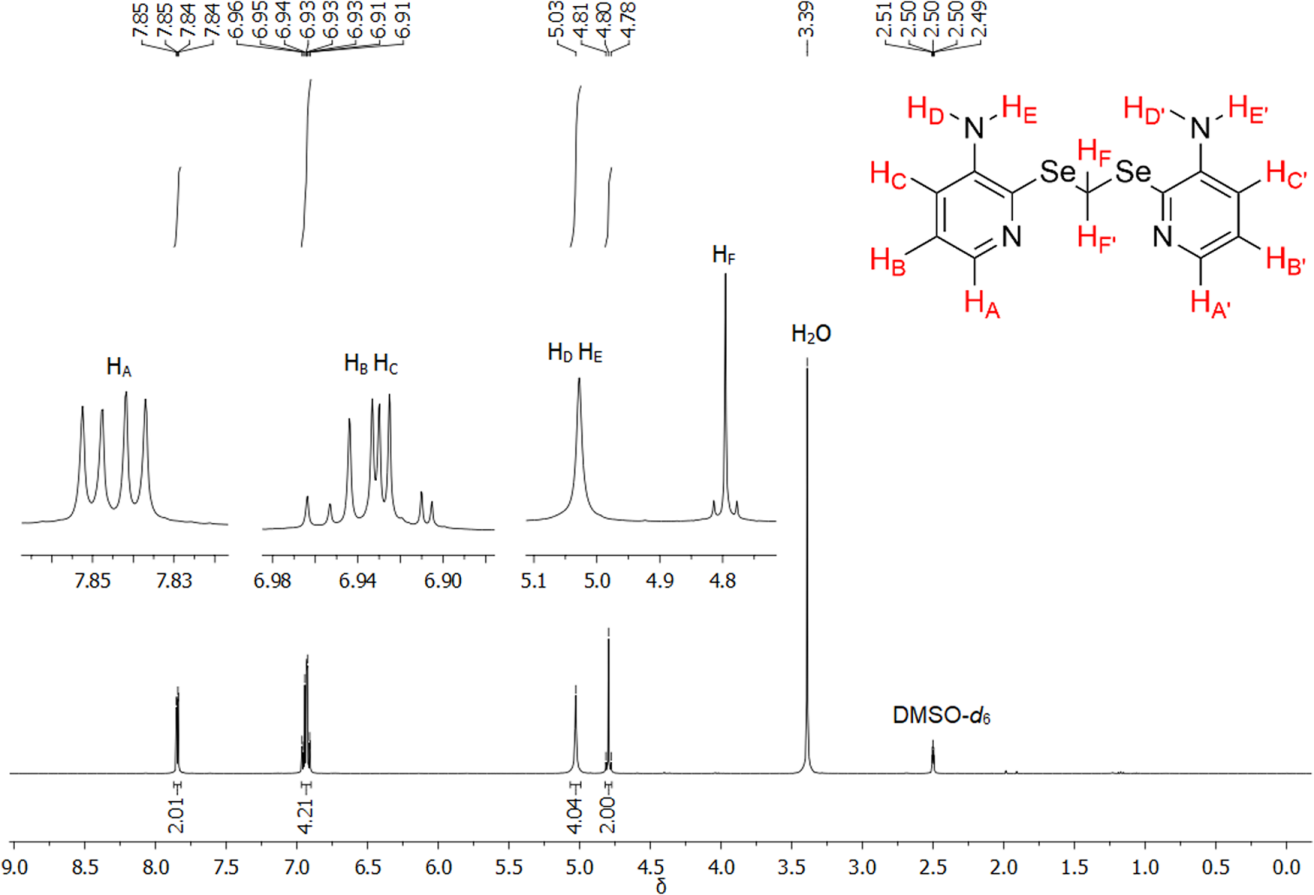
^1^H NMR spectrum (400 MHz, DMSO-*d*_6_) of ligand **L**.

The 1D (^1^H, ^13^C, and ^77^Se) and 2D (COSY, HSQC, and HMBC) NMR analyses were carried out for the ligand **L** and complex **3** (the only diamagnetic complex sufficiently soluble for the experiment), using DMSO-*d*_6_ as the solvent. The ^1^H NMR spectrum (400 MHz, DMSO-*d*_6_) of ligand **L** is shown in Figure 10. The assignments were made based on the spectra of **(3-apySe)**_**2**_ (Figure S43), literature data,^11^ and with the aid of 2D NMR experiments.

A typical signal for this type of molecule is the singlet (s) with satellite bands, corresponding to the H_F_ hydrogens. This is due to the coupling between the nuclei of ^1^H and ^77^Se with a ^2^*J*_HSe_ = 14.5 Hz (^77^Se: natural abundance = 7.63% and spin = 1/2),^11^ resulting in a genuine singlet centered halfway through a small doublet (d). The singlet for the amine hydrogens (H_D_ and H_E_) is also a characteristic signal. In the aromatic region, three doublets of doublets (dd) related to the H_A_, H_B_, and H_C_ hydrogens can be observed in the spectra of **L** and **(3-apySe)**_**2**_, similar to the data described in the literature.^11^

**Figure 11:**
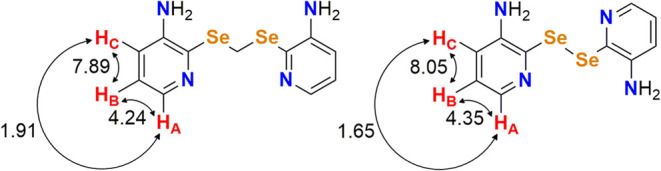
Comparison of coupling constants (in Hz) between ligand **L** and **(3-apySe)**_**2**_.

The assignments of the aromatic hydrogens in the structure of **L** were made based on the spectrum of **(3-apySe)**_**2**_, where the distinct coupling constants (*J*) allowed differentiation between them, especially between H_B_ and H_C_. For better visualization, Figure 11 presents a comparison of the *J* values for the aromatic hydrogens of ligand **L** and **(3-apySe)**_**2**_. It was observed in the spectrum of **L** that the short-distance couplings between H_A_ and H_B_ were 4.2 Hz, between H_B_ and H_C_ were 7.9 Hz, and the long-distance coupling for H_A_ and H_C_ was 1.9 Hz. For **(3-apySe)**_**2**_, the respective couplings are 4.4 Hz (H_A_ and H_B_), 8.0 Hz (H_B_ and H_C_), and 1.6 Hz (H_A_ and H_C_). Therefore, the consistency observed in the *J* values for the two molecules allowed precise differentiation of the aromatic hydrogens. Furthermore, the 2D COSY NMR experiment of ligand **L** (Figures S50–S51) also exemplifies the short-distance couplings observed for H_A_, H_B_, and H_C_, as well as the singlets of H_D_, H_E_, and H_F_.

**Figure 12:**
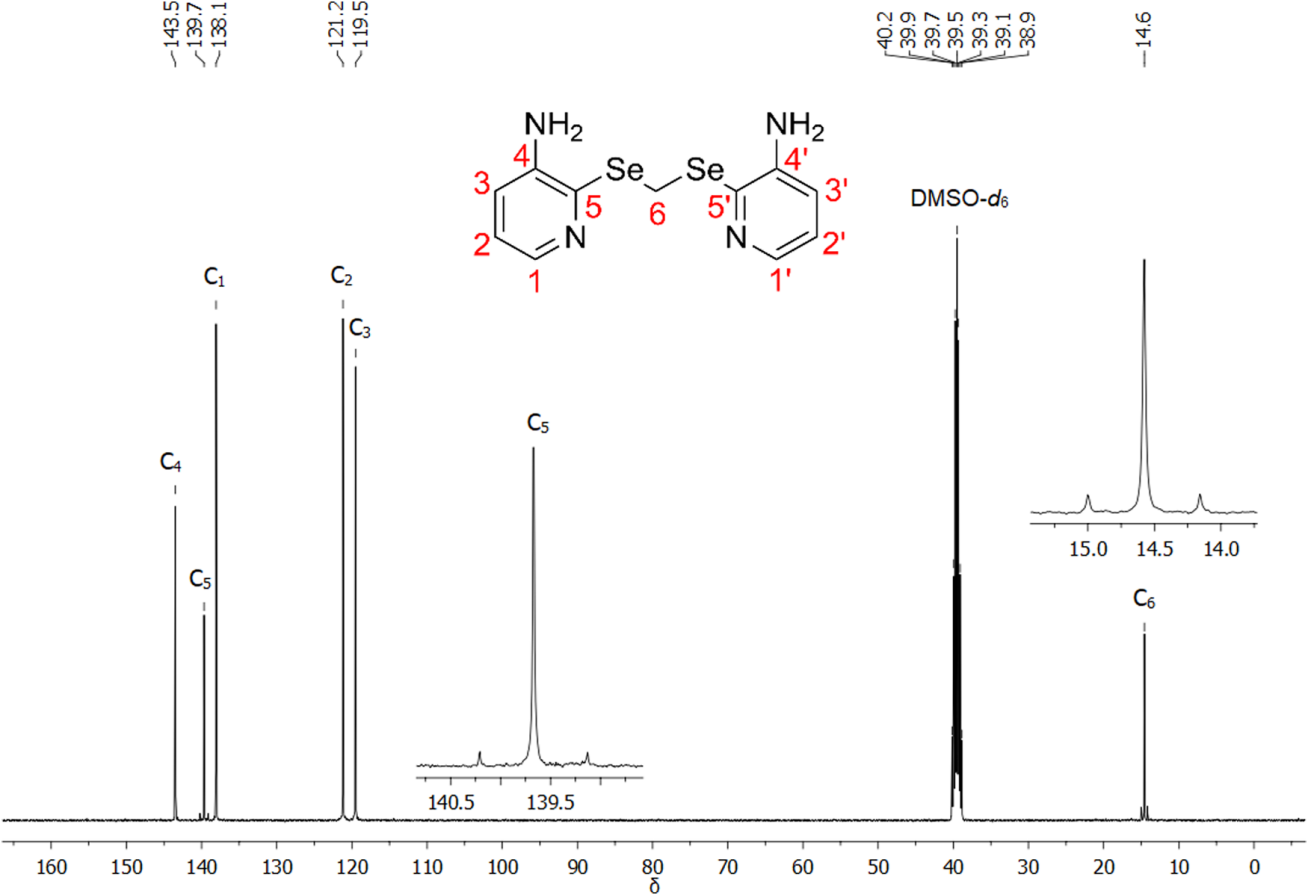
^13^C NMR spectrum (100 MHz, DMSO-*d*_6_) of ligand **L**.

The ^13^C NMR spectrum (100 MHz, DMSO-*d*_6_) of ligand **L** is shown in Figure 12. Similarly to the hydrogen nuclei, the carbon assignments were made based on literature data^11a^ and consolidated by 1D and 2D NMR experiments. Additionally, satellite bands resulting from the coupling between the ^13^C and ^77^Se nuclei are observed for C_5_ (^1^*J*_CSe_ = 107.9 Hz) and C_6_ (^1^*J*_CSe_ = 84.2 Hz).

Although the differentiation between carbons C_2_ and C_3_ may seem complex, the specific *J* values for the aromatic hydrogens make it possible to distinguish between them. Similarly, the non-hydrogenated carbons C_4_ and C_5_ can be difficult to identify without additional information, even though the ^13^C spectrum shows the satellite bands for C_5_, corresponding to the ^1^*J*_CSe_ coupling. Based on the same principle, 2D NMR HSQC and HMBC experiments (Figures S52–S58) were carried out to understand the connectivities between the hydrogen and carbon atoms and to complement the studies of the 1D NMR.

Through the HSQC experiment, it was possible to observe the direct couplings (^1^*J*) between the methylene carbon and its hydrogens (H_F_/C_6_), and between the three hydrogenated carbons of the aromatic ring, C_1_, C_2_, and C_3_ (with H_A_, H_B_, and H_C_, respectively). Additionally, from the long-distance couplings observed in the HMBC spectrum, only one ^3^*J* coupling between H_F_ and C_5_ was observed, which is important for the differentiation between C_4_ and C_5_.

**Figure 13:**
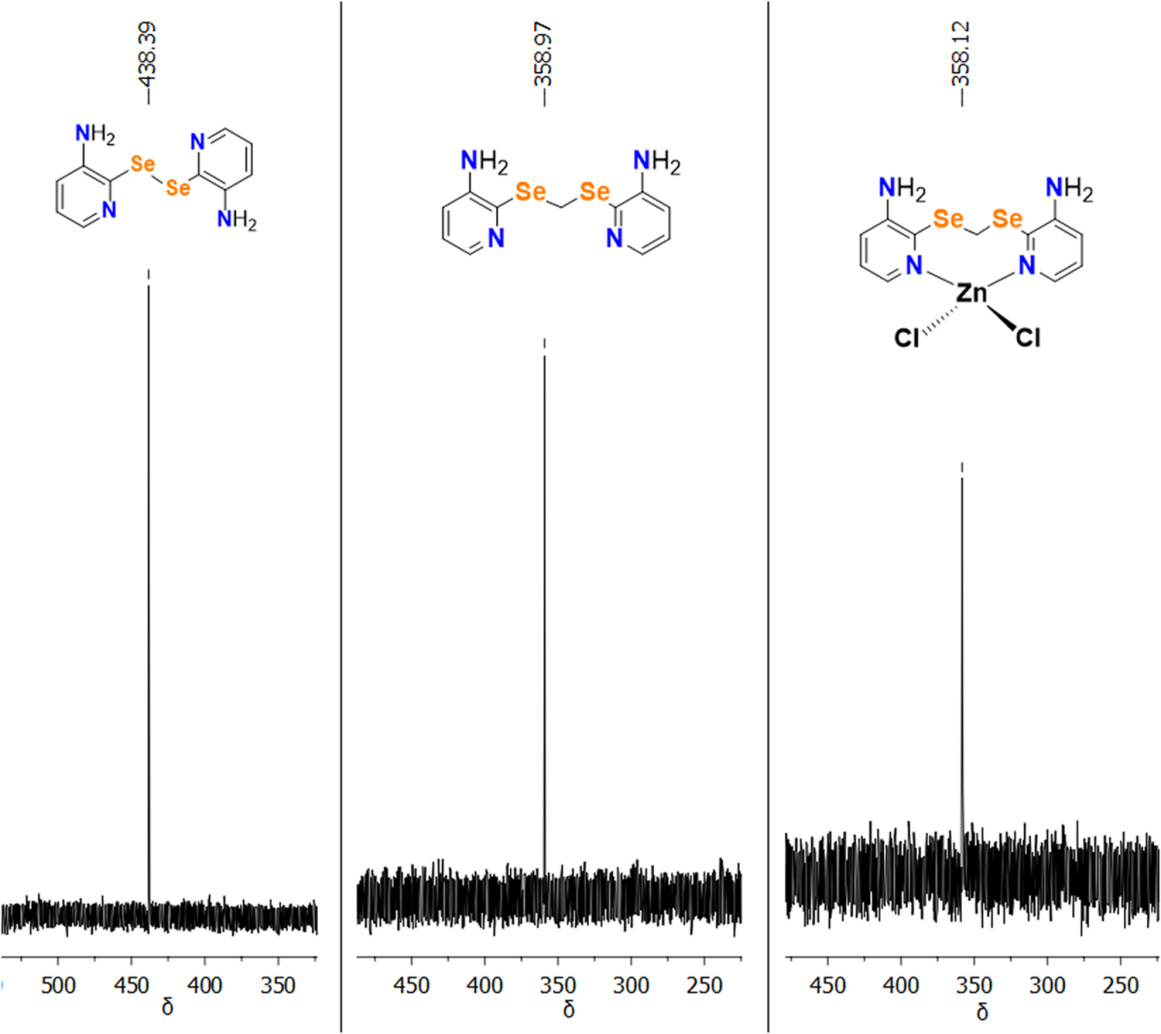
Comparison of ^77^Se NMR spectra (76 MHz, DMSO-*d*_6_) of diselane **(3-apySe)**_**2**_, ligand **L**, and complex **3**.

All 1D and 2D NMR techniques performed for ligand **L** were also applied to compound **3**. In general, in terms of signal multiplicities, integrations, spectral profiles, and couplings, all the information is equivalent (Figures S59–S61). Based on the chemical shifts observed in the ^77^Se NMR spectra, it is possible to confirm the presence of ligand **L** as the only selenium species in the structure of complex **3**. Figure 13 presents the comparison of the signals for the diselane **(3-apySe)**_**2**_, ligand **L**, and complex **3**.

### High-Resolution Mass Spectrometry (HRMS)

The complexes **1–10** were subjected to HRMS analyses. Although all were tested, compounds **6–8** did not exhibit useful fragmentations, and therefore, the data analysis did not allow for their structural elucidation. Notably, the complexes were evaluated by elemental analysis, which ruled out possible impurities in the samples. The other compounds (**1–5**, **9**, and **10**) were evaluated by HRMS, and Table 2 presents their main characteristic fragments (all spectra are provided in the SI, Figures S62–S80).

**Table 2:** Main Characteristic Fragments Observed by HRMS Analysis

complex	fragment	mass-to-charge ratio (*m*/*z*)
**1–5**, **9**, **10**	[**L** + H]^+^	360.95 (approximate)
**1**	[**L**CoCl]^+^	453.8467
**2**	[**L**CoBr]^+^	497.7931
**3**	[**L**ZnCl]^+^	458.8403
**4**	[**L**CuCl]^+^	457.8427
**5**	[**L**CuBr]^+^	501.7916
**9**	[**L**Ag]^+^	466.8484
**9**	[**L**_**2**_Ag]^+^	826.7912
**10**	[**L**Ag]^+^	466.8500
**10**	[**L**_**2**_Ag]^+^	826.7952

All complexes share the common fragment [**L** + H]^+^, corresponding to the ligand **L**, with an approximate *m*/*z* ratio of 360.95. Compounds **1–5** exhibit the characteristic fragment [**L**MX]^+^ (M = Co, Cu, Zn; X = Cl, Br), originating from the loss of a halogen unit from the metal coordination sphere. The silver-derived complexes (**9** and **10**) display the fragments [**L**_**2**_Ag]^+^ and [**L**Ag]^+^ with approximate *m*/*z* ratios of 826.79 and 466.85, respectively, characterized by the loss of an Ag^I^ center and an **L** unit.

### Ultraviolet–Visible (UV–Vis) Spectroscopy

Through UV–vis spectroscopy in solution, the electronic transitions involving the frontier orbitals (mainly) of ligand **L** and complexes **1–10** were characterized. The solutions were prepared by dissolving the products in DMF, as it was the same solvent used for the impregnation process of m-TiO_2_. In cases where it was not possible to fully solubilize the compound, DMSO was used as an alternative, aiming to maintain the initial concentration of the solution for subsequent calculations of ε_max_ (molar absorptivity coefficient at the maximum wavelength, described in the SI Synthesis Procedures). However, some complexes were not fully soluble, and their ε_max_ values were not determined (**6**, **8**, and **9**).

**Figure 14:**
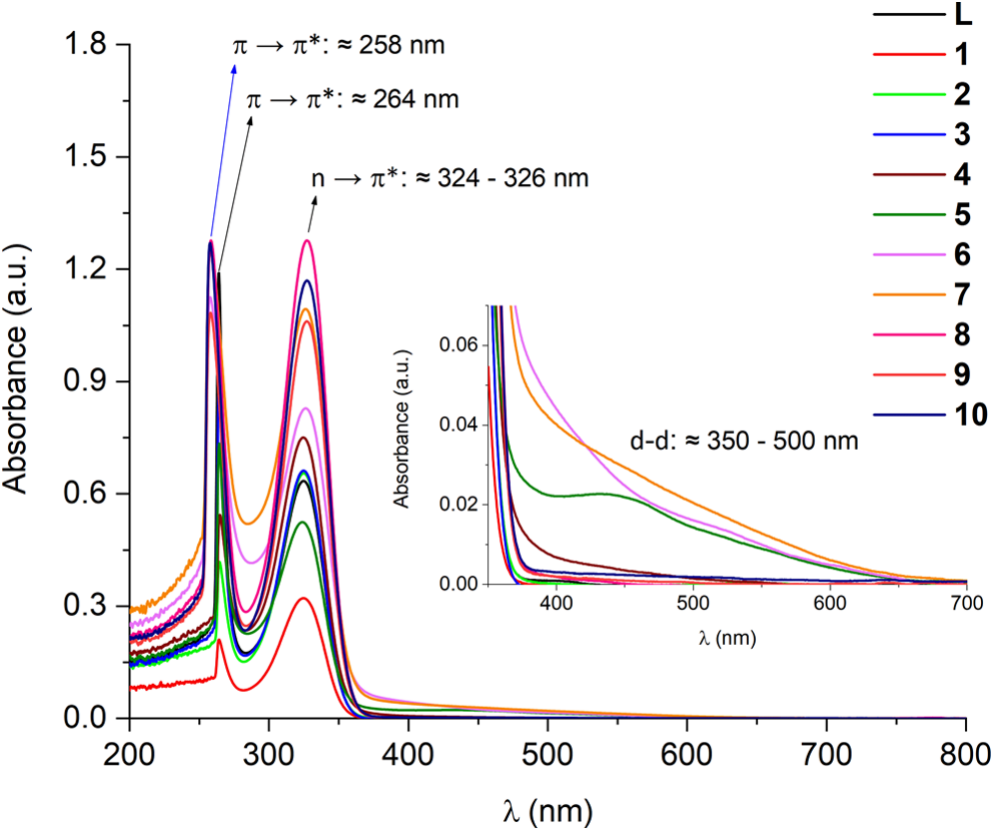
UV–vis spectra in solution of ligand **L** and complexes **1–10**. Solutions in DMF: **L**, **1**–**5**; in DMSO: **6**–**10**.

According to the experimental results, the ligand **L** and complexes **1–10** demonstrate the same absorption profile in solution, presenting similar characteristic electronic transitions, represented in Figure 14. Considering the nature of the ligand, the bands around 260 and 325 nm can be attributed to the intraligand transitions of the type π→π* and n→π*, respectively. Complementarily, ligand field transitions of the d-d type at approximately 350 to 500 nm can be observed for the complexes.^6,12^ The minimal wavelength variation of the π → π* transitions, in some complexes, is due to the solvents used in the preparation of the solutions (258 nm in DMSO or 264 nm in DMF). This distinct interaction with the medium can be evidenced in the qualitative spectra of compounds **7** and **8** in DMF (Figure S81). Additionally, the coordination of **L** to the metal centers did not cause considerable displacement in any of the intraligand transitions in the products, supported by reports in the literature where spectra of complexes are similar to their ligands.^13^

According to the experimental results, ligand **L** and complexes **1–10** demonstrate the same absorption profile in solution, presenting similar characteristic electronic transitions, as shown in Figure 14. Considering the nature of the ligand, the bands around 260 and 325 nm can be attributed to the intraligand transitions of the type π→π* and n→π*, respectively. Additionally, ligand field transitions of the d-d type, occurring at approximately 350 to 500 nm, can be observed for the complexes.^6b,12^ The minimal wavelength variation of the π→π* transitions in some complexes is due to the solvents used in the preparation of the solutions (258 nm in DMSO or 264 nm in DMF). This distinct interaction with the medium can be evidenced in the qualitative spectra of compounds **7** and **8** in DMF (Figure S81). Furthermore, the coordination of **L** to the metal centers did not cause significant displacement in any of the intraligand transitions in the products, supported by reports in the literature where spectra of complexes are similar to their ligands.^13^

The stability of the compounds in solution was evaluated through absorbance measurements over time, with data collected every 30 min for 6 h (Figures S82–S84). In general, the ligand and complexes remained stable during this period, except for the silver derivatives (**9** and **10**). Colorless crystals of **9** were only partially solubilized in DMSO, and only the soluble fraction was subjected to testing. Although the time-dependent spectra suggested the compound’s stability, the preparation of the solution indicated otherwise, as the insoluble crystals turned black, signaling decomposition. In contrast, the crystals of **10** were completely soluble. The appearance of a band in the visible region (350–600 nm) over the course of the experiment indicates the formation of decomposition products (Figure S84). These absorptions may be associated with electronic transitions of the TCML type (charge transfer from the metal to the ligand), involving species in solution derived from the Ag^I^ center (low oxidation state and electron-rich) and ligand **L** (a good π acceptor).^14^

**Figure 15:**
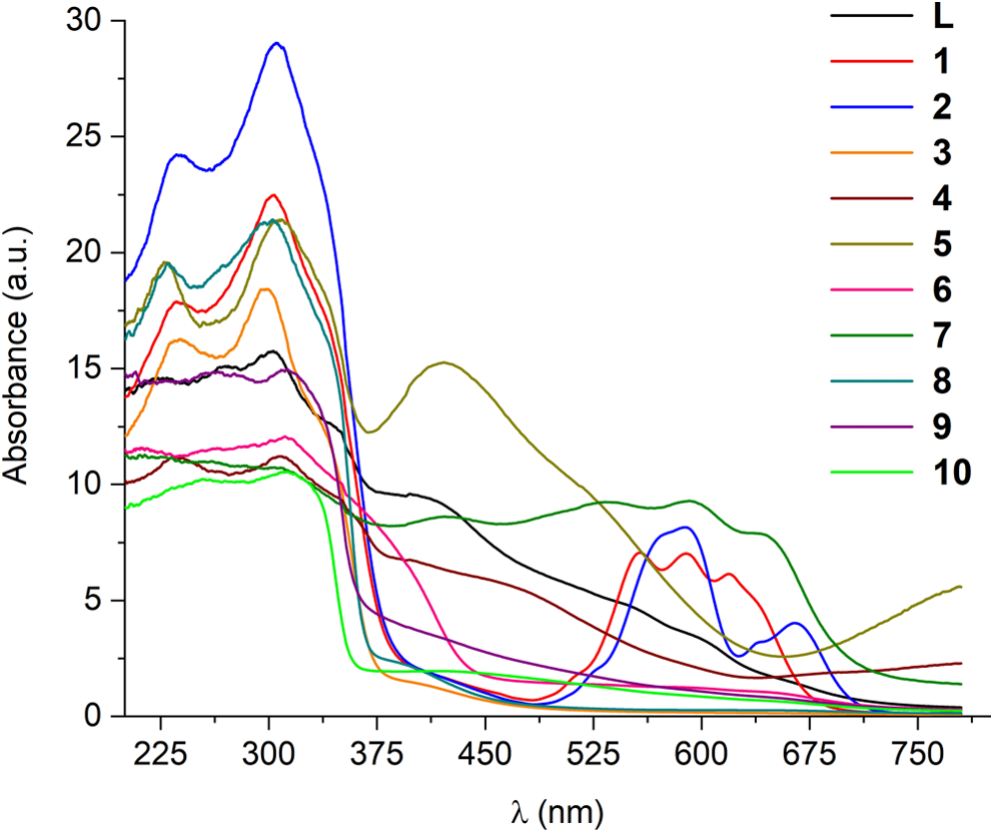
Absorbance spectra of ligand **L** and complexes **1–10**, calculated from diffuse reflectance data.

To evaluate the compounds as potential cocatalysts for m-TiO_2_, their energy gap (*E*_g_) values were determined from UV–vis spectroscopy data obtained by diffuse reflectance in the solid state (DRS) (Figures S85–S89). To complement the electronic transitions of the compounds recorded in solution, the diffuse reflectance data were converted into absorbance using the Kubelka–Munk function.^15^ The absorbance spectra of **L** and complexes **1–10**, calculated from this conversion, are presented in Figure 15. This approach allows for better visualization of the d-d transitions of the Cu^II^ and Co^II^ complexes in the visible region (400–700 nm).

The *E*_g_ values were estimated using Tauc plots, calculated by converting the diffuse reflectance data into absorbance (Figures S85–S89). The estimated *E*_g_ values for m-TiO_2_, ligand **L**, and complexes **1–10** are listed in Table 3. With a diverse range of *E*_g_ values, varying from 1.74 to 3.33 eV, these compounds show potential as cocatalysts for m-TiO_2_ in the water photolysis process.

**Table 3:** Estimated *E*_g_ Values of m-TiO_2_, Ligand **L**, and Complexes **1–10**

compound	*E*_g_ (eV)	compound	*E*_g_ (eV)
m-TiO_2_	3.41	**5**	2.22
**L**	2.36	**6**	2.77
**1**	1.81	**7**	1.74
**2**	1.74	**8**	3.25
**3**	3.23	**9**	3.26
**4**	2.86	**10**	3.33

### Photocatalytic Production of Hydrogen Gas

**Figure 16:**
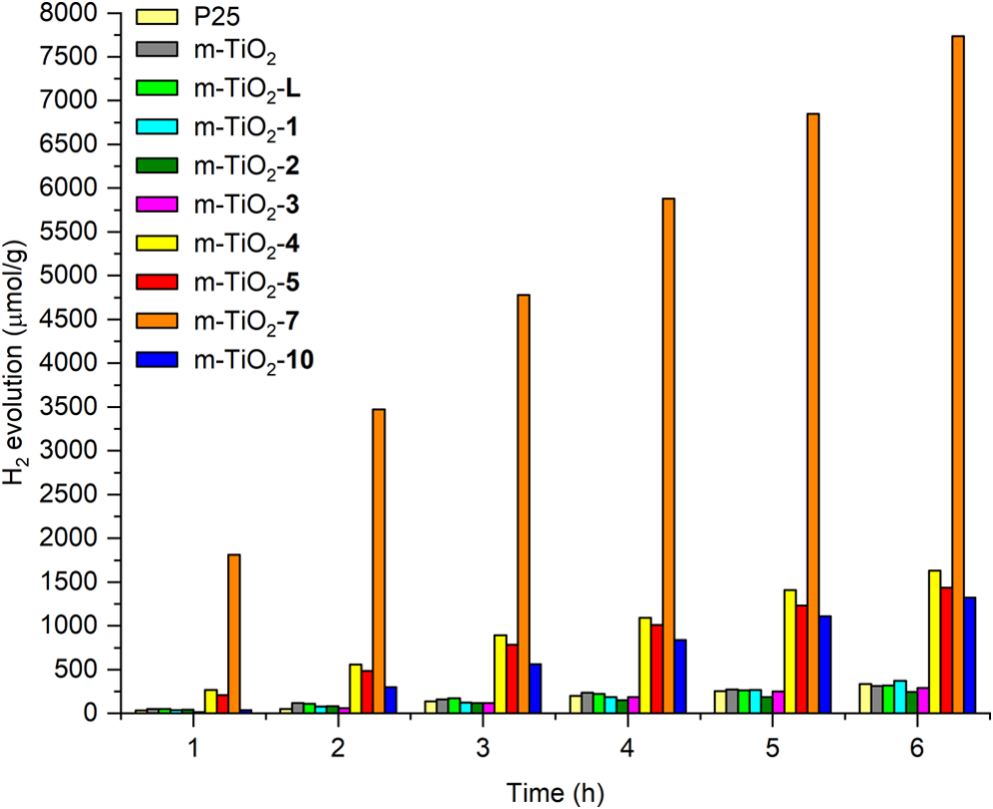
Results of H_2_(g) evolution for the photocatalysts m-TiO_2_-**n** (**n** = **L**, **1**–**5**, **7**, or **10**), compared to pure m-TiO_2_ and P25. Sacrificial agent: TEOA (10% in aqueous solution); light source: 300 W Xe/Hg lamp; photocatalyst/solution concentration: 0.5 g/L.

Following the impregnation methodology for m-TiO_2_ (SI, Photocatalysis), materials labeled as m-TiO_2_-**n** were prepared (Figure S98), where **n** represents the compound used as cocatalyst (*n* = **L**, **1–5**, **7**, or **10**). Due to the low solubility of complexes **6**, **8**, and **9**, they were not used as cocatalysts for m-TiO_2_. The photocatalysts were evaluated for hydrogen gas production under photocatalytic conditions. The results, obtained over a 6-h experiment, are illustrated in Figure 16 and compared to pure m-TiO_2_ and P25 (particle size: 21 nm; Sigma-Aldrich).

The best performance was achieved with the photocatalyst m-TiO_2_-**7**, which exhibited high photocatalytic efficiency, producing approximately 26 times more H_2_(g) than the reference standards m-TiO_2_ and P25 (as shown in Table 4). The materials m-TiO_2_-**4**, -**5**, and -**10** also performed well, producing about 4 to 5 times more H_2_(g) than the reference standards. In contrast, the analogs m-TiO_2_-**L**, -**1**, -**2**, and -**3** did not show significant photocatalytic H_2_(g) production compared to the pure support solids.

**Table 4:** Approximate H_2_(g) Evolution for the Evaluated Materials

photocatalysts	H_2_(g) (μmol/g)	photocatalysts	H_2_(g) (μmol/g)
P25 and m-TiO_2_	300		
m-TiO_2_-**L**	300	m-TiO_2_-**4**	1600
m-TiO_2_-**1**	370	m-TiO_2_-**5**	1400
m-TiO_2_-**2**	250	m-TiO_2_-**7**	7800
m-TiO_2_-**3**	300	m-TiO_2_-**10**	1300

To summarize the best results of our work in conjunction with relevant literature, Table 5 presents some metal complexes used as cocatalysts of TiO_2_ in the photocatalytic production of H_2_. All materials were tested in the same conditions as in our study: TEOA (10% in aqueous solution) as the sacrificial agent, a 300 W Hg/Xe lamp as a light source, a photocatalyst/solution concentration of 0.5 g/L, and a total irradiation period of 6 h, with hourly H_2_ sampling. Notably, cocatalysts based on less noble metals, such as Cu and Ni, demonstrated satisfactory photocatalytic performance. In addition, the m-TiO_2_-**7** photocatalyst outperforms previously reported examples under similar experimental conditions, both in terms of hourly hydrogen evolution and cumulative hydrogen production after 6 h.

**Table 5:** Examples of TiO_2_-Based Materials Modified by Cocatalysts for H_2_(g) Production^a^

cocatalyst	H_2_ evolution rate (μmol/g·h)	H_2_ evolution (μmol/g) after 6 h	reference
[Cu_2_(μ-SO_4_)_2_**L**_**2**_] (**7**)	1300	7800	this work
[CuCl_2_**L**] (**4**)	266.7	1600	this work
[CuBr_2_**L**] (**5**)	233.3	1400	this work
[Ag_2_**L**_**2**_](NO_3_)_2_ (**10**)	216.7	1300	this work
[Zn(μ-HSeO_3_)_2_(bipy)]_*n*_	269.2	1615	(16)
[Zn(μ-HSeO_3_)_2_(phen)]_*n*_	55.8	335.5	(16a)
[Co(2-Py_2_Te_2_-κ*N,N′*)Cl_2_]	102.5	615	(16b)
[Co(2-Py_2_Te_2_-κ*N,N′*)Br2]	97.8	587	(16b)
[CuCl_2_(LC)_2_]^b^	1052.5	6315	(16c)
[MnCl_2_(LC)_2_]^b^	<25	<150	(16c)
[FeCl_2_(LC)_2_]^b^	<25	<150	(16c)
[CoCl_2_(LC)_2_]^b^	<25	<150	(16c)
[NiCl_2_(LC)_2_]^b^	<25	<150	(16c)
Ni(LT)^c^	1029.8	6179	(16d)
Ni(LT)^c^	383.5	2301	(16d)
(H_2_LT)^c^	106.3	638	(16d)
Cu(LT)^c^	60	360	(16d)

a To all examples: the sacrificial agent was TEOA (10% in aqueous solution), the light source was a 300 W Hg/Xe lamp, and the photocatalyst/solution concentration was 0.5 g/L.

b LC: chromone-derived ligand (C_19_H_11_NO_5_).

c H_2_LT: thiophene-2,5-dicarboxaldehyde-bis-thiosemicarbazone (C_8_H_10_N_6_S_3_), LT (C_8_H_8_N_6_S_3_).

**Figure 17:**
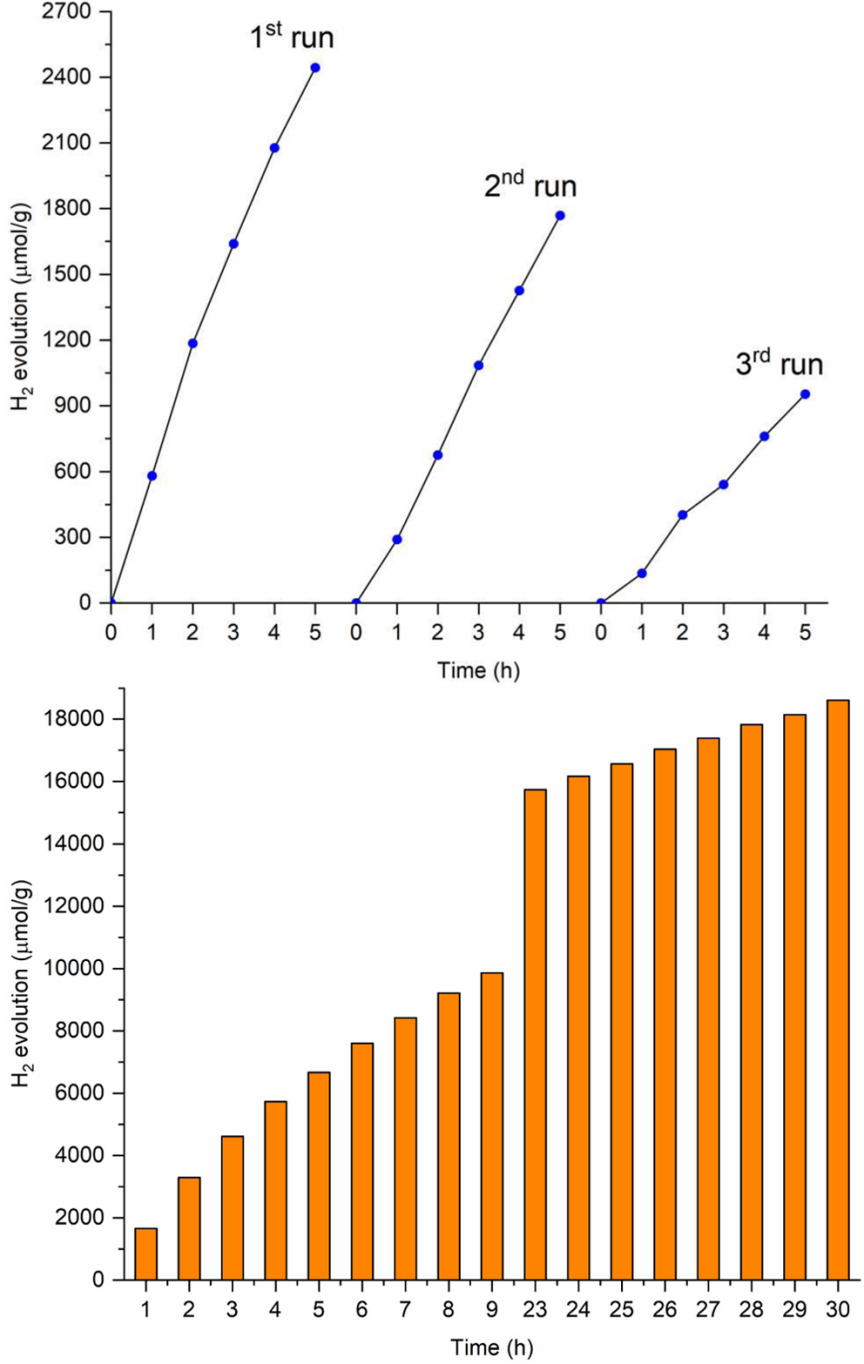
Results of H_2_(g) evolution in the recycling experiments with the m-TiO_2_-**7** photocatalyst (top) and over 30 h (bottom).

Considering the m-TiO_2_-**7** photocatalyst as the most efficient, recycling experiments were conducted to evaluate its performance over continuous photocatalytic tests. Initially, 10 mg of the previously used photocatalyst was recovered for reuse. The photocatalyst was then irradiated through three cycles of 5 h each, with the reactor being opened and the solution purged with argon at the end of each cycle to remove the produced H_2_(g). The results are presented in Figure 17 (top), showing a decrease in H_2_(g) production after each cycle compared to its initial use.

Despite the reduction in photocatalytic efficiency, the photocatalyst still exhibited significant activity, producing approximately 1000 μmol/g of H_2_(g) after the third cycle–about four times more than pure m-TiO_2_. Additionally, 30-h irradiation tests (Figure 17, bottom) demonstrated a gradual increase in photocatalytic efficiency over extended periods, reaching approximately 18,600 μmol/g of H_2_(g) by the end of the experiment. These results indicate that the amount of TEOA is sufficient and does not become fully depleted over the 30-h period. Furthermore, they suggest that the m-TiO_2_-**7** photocatalyst can withstand prolonged irradiation and only experiences a decline in efficiency when exposed to atmospheric air, as observed in the recycling tests. However, the exact cause of this reduction and the changes occurring upon air exposure remain unclear. Identifying this limitation as a key challenge of our study, further investigations will be conducted to better understand the observed phenomena.

### m-TiO_2_-**n**: Loading Determination

The loading of the m-TiO_2_-**n** photocatalysts was determined using the Lambert–Beer law and UV–vis spectroscopy^17^ (Figures S90–S95). The results are summarized in Table 6. Notably, the m-TiO_2_-**7** photocatalyst exhibited the highest photocatalytic efficiency among the materials evaluated, despite all of them showing considerably low loading values. These results indicate that even a very small amount of complex **7** can significantly enhance the photocatalytic activity of m-TiO_2_. Calculations were not performed for m-TiO_2_-**10**, as degradation of the complex was observed during the impregnation process (m-TiO_2_-**10** appeared gray, Figure S98), likely due to the presence of Ag^0^.

**Table 6:** Immobilization of Compounds on m-TiO_2_ and Loading of m-TiO_2_-**n** Photocatalysts

photocatalysts	immobilization of compound (wt %)	loading (μmol/g)
m-TiO_2_-**L**	0.0909	2.550
m-TiO_2_-**1**	0.278	5.707
m-TiO_2_-**2**	0.200	3.456
m-TiO_2_-**3**	0.150	3.030
m-TiO_2_-**4**	0.108	2.191
m-TiO_2_-**5**	0.0834	1.303
m-TiO_2_-**7**	0.306	2.960

### m-TiO_2_-**n**: PXRD

The powder diffraction patterns were recorded for all photocatalysts (Figures S99–S100). The diffractogram of m-TiO_2_ was compared to the theoretical standard of the anatase crystalline phase,^18^ and only its main crystallographic planes were observed. Due to the low impregnation rate of the cocatalysts on the solid support, the diffractograms of the m-TiO_2_-**n** photocatalysts were virtually identical to that of pure m-TiO_2_.

The average crystallite size (in nm) determined for the photocatalysts is presented in Table 7, with all values being very close to each other. Similar to complex **4**, the small values may be associated with peak broadening observed in the diffractograms.

**Table 7:** Average Crystallite Sizes of the Photocatalysts

photocatalysts	average size (nm)	photocatalysts	average size (nm)
m-TiO_2_	22.95		
m-TiO_2_-**L**	15.26	m-TiO_2_-**4**	15.41
m-TiO_2_-**1**	17.90	m-TiO_2_-**5**	19.11
m-TiO_2_-**2**	16.20	m-TiO_2_-**7**	15.55
m-TiO_2_-**3**	16.57	m-TiO_2_-**10**	19.23

### m-TiO_2_-**n**: Vibrational Spectroscopy

**Figure 18:**
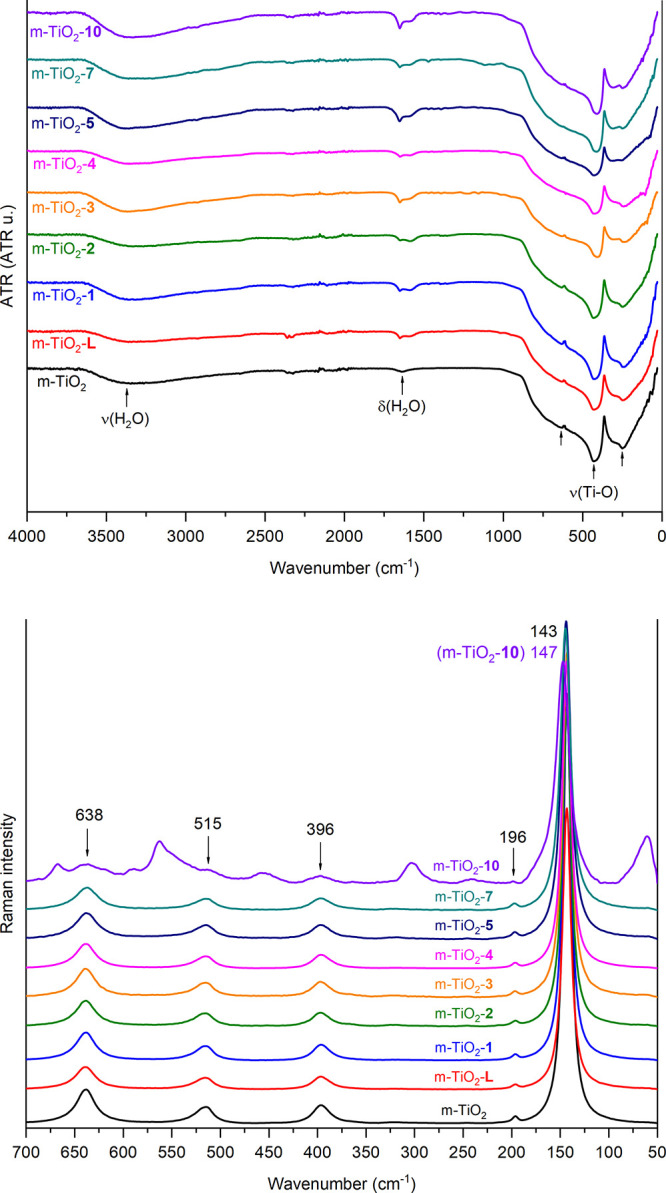
FT-IR (top) and Raman (bottom) spectra of m-TiO_2_-**n** photocatalysts.

The photocatalysts were evaluated by vibrational spectroscopy, and Figure 18 (top) shows the comparison of the FT-IR spectra of m-TiO_2_ and m-TiO_2_-**n**. The main absorptions observed are related to the stretching and bending vibration bands of the hydration water of the materials (3600 to 2800 cm^–1^ and 1650 to 1580 cm^–1^, respectively), and the Ti–O stretching between 630 and 250 cm^–1^.^19^ Since the impregnation levels are very low, the FT-IR spectra of the m-TiO_2_-**n** photocatalysts are nearly identical to that of pure m-TiO_2_.

The comparison of the Raman spectra of the photocatalysts is also shown in Figure 18 (bottom). For m-TiO_2_, its typical peaks corresponding to the spectral pattern of the anatase phase are observed,^19c,19d^ and, in general, the spectra of the other photocatalysts are nearly identical. An exception is m-TiO_2_-**10** (Figure S101), which, in addition to the characteristic peaks of the anatase phase, exhibits additional peaks related to the decomposition of complex **10** during its impregnation process. This may be associated with the plasmonic effect, possibly resulting from the deposition of nanoparticulate silver on the surface of m-TiO_2_, which could enhance the Raman spectrum absorptions by amplifying the free electrons on the material’s surface.^20^ Additionally, the DRS and absorbance spectra of the photocatalysts are presented in Figures S102–S103.

### m-TiO_2_-**n**: Scanning Electron Microscopy (SEM) and Energy-Dispersive X-ray Spectroscopy (EDS)

SEM and EDS analyses were performed for m-TiO_2_ as well as for the m-TiO_2_-**n** photocatalysts that exhibited satisfactory photocatalytic activity (**n** = **4**, **5**, **7**, and **10**).

**Figure 19:**
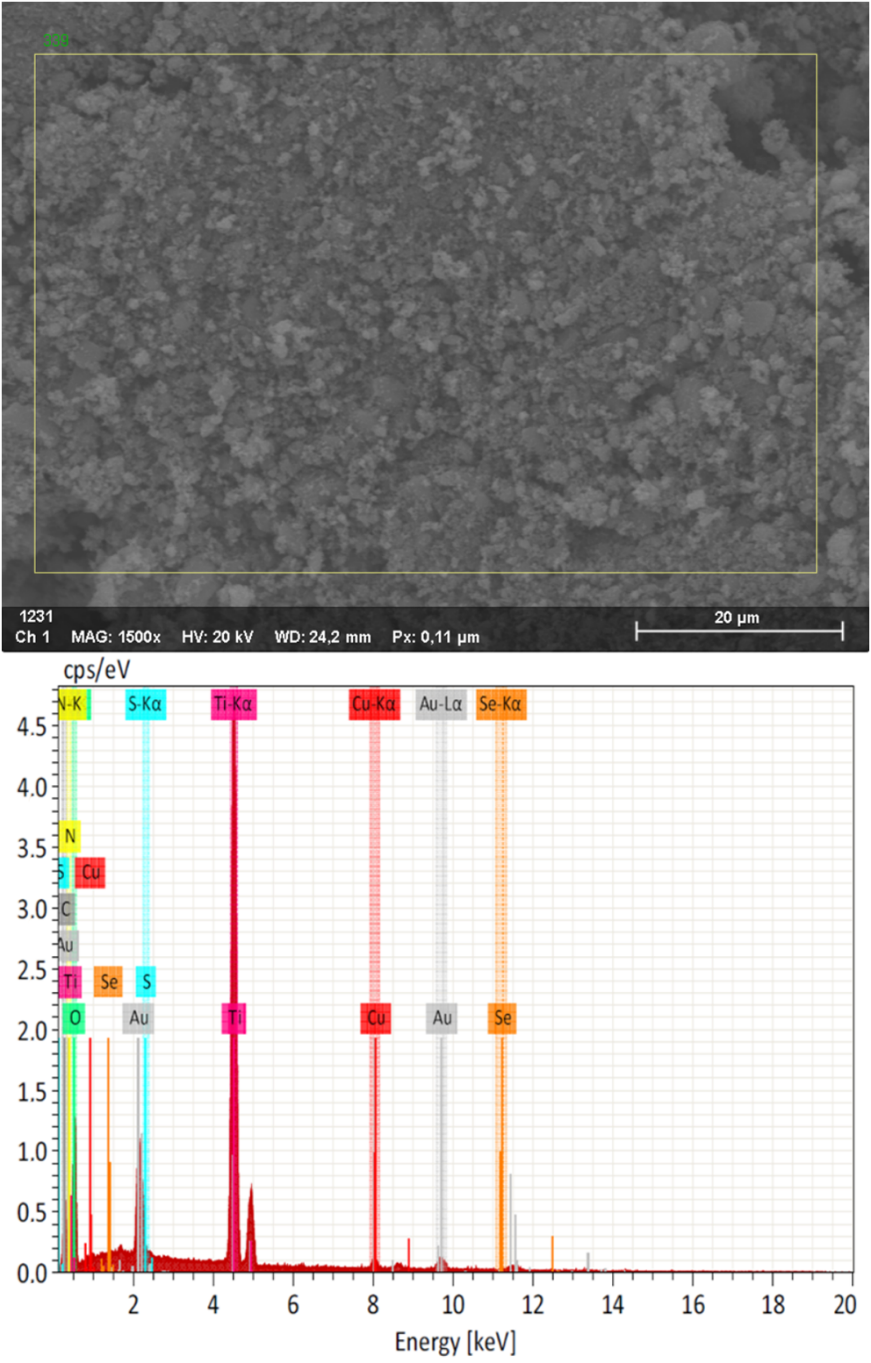
SEM image (top) and EDS spectrum (bottom) of the m-TiO_2_-**7** photocatalyst.

For the m-TiO_2_-**7** photocatalyst, Figure 19 (top) presents its SEM image, which reveals particles with irregular sizes and poorly defined shapes. The other photocatalysts exhibit similar characteristics (Figure S104), as this is an inherent feature of m-TiO_2_. Its EDS spectrum (Figure 19, bottom) displays characteristic peaks corresponding to the expected elements, confirming its chemical composition (the presence of gold is due to sample metallization). Likewise, the other photocatalysts show the same behavior (Figures S105–S106).

**Figure 20:**
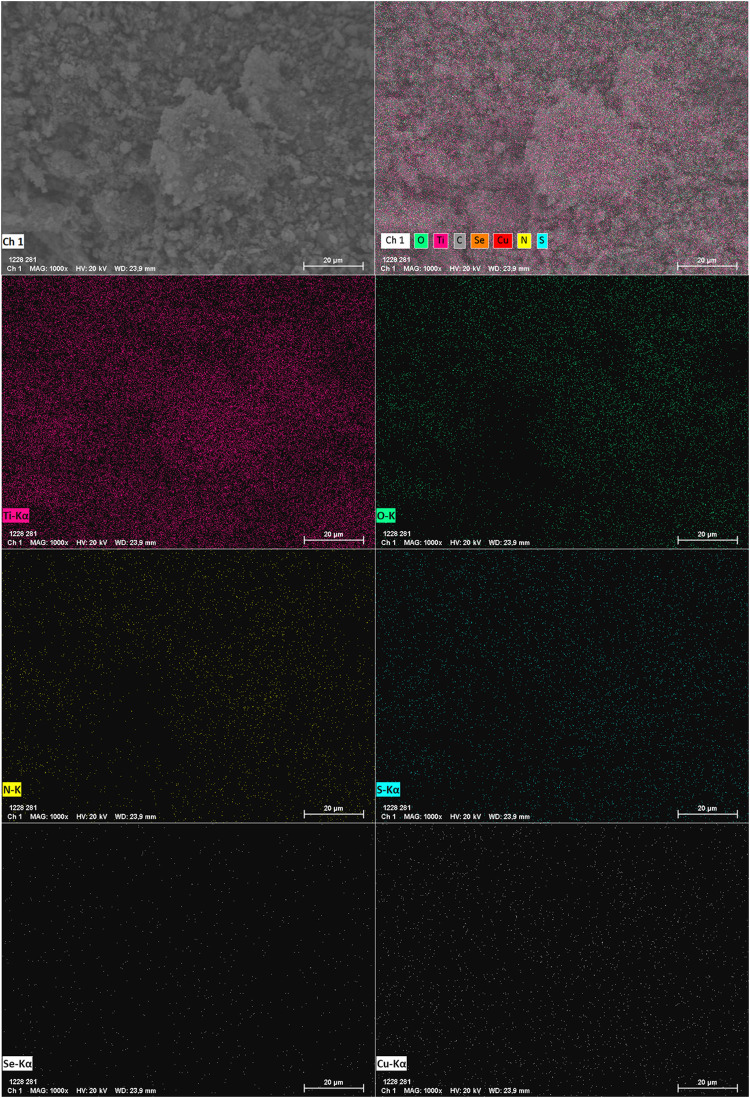
Elemental mapping of the m-TiO_2_-**7** photocatalyst. The mapping of C was omitted, as it is present in the carbon tape used for sample preparation. Image saturation and lightness have been adjusted for clarity.

Elemental mapping (Figure 20) further indicates that the elements identified by EDS are homogeneously distributed across the material’s surface. Similarly, the elemental mappings of the other photocatalysts (Figures S107–S110) demonstrate uniform distribution, confirming the efficiency of the preparation method. These characteristics are maintained in m-TiO_2_-**7** even after photocatalytic tests, as described in the postphotocatalysis analysis (Figures S111–S113).

### Cyclic Voltammetry (CV)

To understand the redox processes of the compounds used for the impregnation of m-TiO_2_, they were evaluated by CV (Figures S96–S97).

For the ligand **L**, a reduction process at *E*_1/2_ = −0.40 V (vs NHE) and two oxidation processes (*E*_ap_ = +1.06 V, *E*_ap_ = +1.53 V) were observed. Considering the nature of the ligand and data from the literature,^21^ anionic radical species are initially formed (*E*_cp_ = −0.49 V) and subsequently oxidized (*E*_ap_ = −0.32 V) at *E*_1/2_ = −0.40 V. Concurrently, processes involving the formation of intermediate cationic radical species, followed by their reaction with O_2_ to form selenoxides (Se = O), can be observed at *E*_ap_ = +1.06 V and *E*_ap_ = +1.53 V. Furthermore, the reduction of the oxidized species is observed at *E*_cp_ = −0.30 V. Similarly, the reduction and oxidation processes of the complexes were evaluated and are summarized in Table 8.

**Table 8:** Electrochemical Data (in V vs. NHE) for Ligand **L** and Complexes **1**–**5**, **7**, and **10**

compound	*E*_red1_^a^	*E*_red2_^a^	*E*_ox1_^b^	*E*_ox2_^b^	*E*_ox3_^b^
**L**	–0.40^c^	–0.30	1.06	1.53	
**1**	–0.41^c^		1.05		
**2**	–0.47				
**3**	–0.47	–0.22	1.40		
**4**	–0.78		0.50^c^	1.13	–0.40
**5**	–0.85		0.66^c^	1.21	
**7**	–1.00		0.70	1.13	
**10**	–0.71	–0.12	1.10	0.40	

a *E*_cp_ = cathodic peak potential.

b *E*_ap_ = anodic peak potential.

c *E*_1/2_ = (*E*_ap_ + *E*_cp_)/_2_.

**Figure 21:**
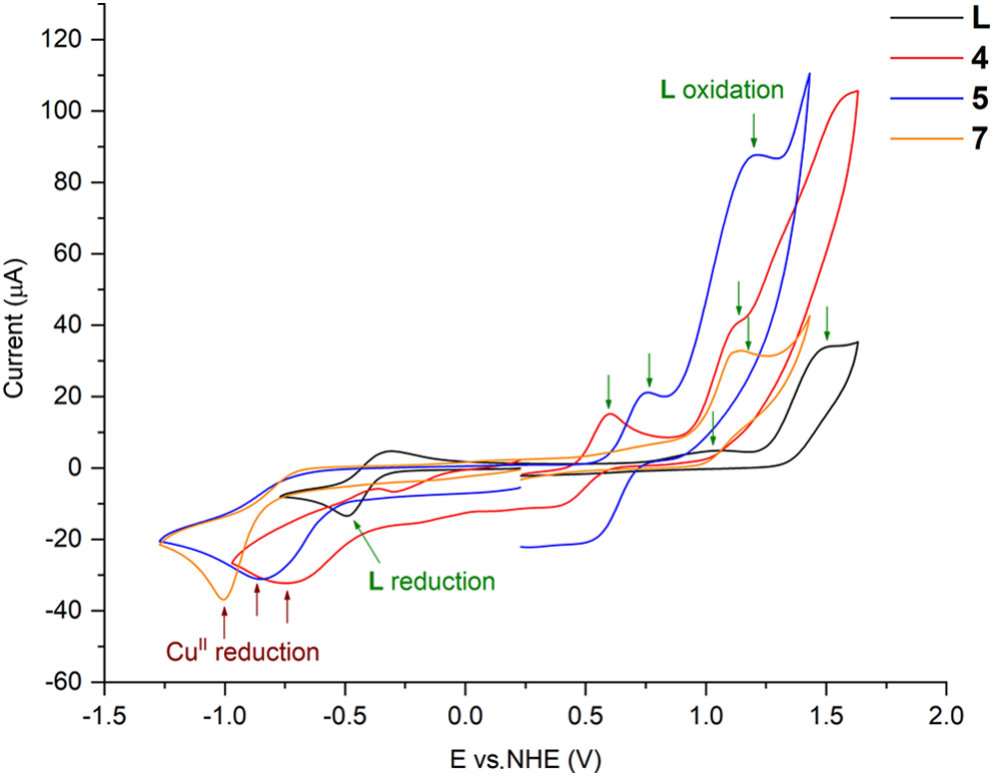
Comparison of the voltammograms of ligand **L** and complexes **4**, **5**, and **7**.

Some trends can be observed, particularly among the Cu^II^-derived complexes (**4**, **5**, and **7**). The three derivatives exhibit the reduction of Cu^II^ to Cu^I^ at potentials of *E*_cp_ = −0.78 V, *E*_cp_ = −0.85 V, and *E*_cp_ = −1.00 V, respectively.^21d,21e^ Based on the basic principle that species with higher electron density are more difficult to reduce (or easier to oxidize), it is noted that the difficulty in reducing the metal increases in the order: **4** < **5** < **7**. This trend may be related to the contribution of the inorganic ligands associated with these compounds, where the bridging sulfate (in **7**) likely donates more electron density to the copper centers than the halides (in **4** and **5**). Conversely, the oxidation processes of **L** occur more easily in the complexes than in its isolated form. Figure 21 presents a comparison of the voltammograms of **L**, **4**, **5**, and **7**.

For compound **10**, the oxidation of Ag^0^ to Ag^I^ at *E*_ap_ = +0.40 V and the reduction of Ag^I^ to Ag^0^ at *E*_cp_ = −0.12 V were observed.^21f,21g^ Redox processes of **L** were also detected at *E*_cp_ = −0.71 V and *E*_ap_ = +1.10 V. In the case of the two Co^II^ complexes (**1** and **2**), precipitate deposition on the working electrode surface was observed during the experiment, preventing efficient contact with the solution and compromising the acquisition of higher-quality voltammograms. For compound **5**, only the redox processes of **L** were observed.

### Electron Transfer Mechanism of m-TiO_2_-**7**

**Scheme 2:**
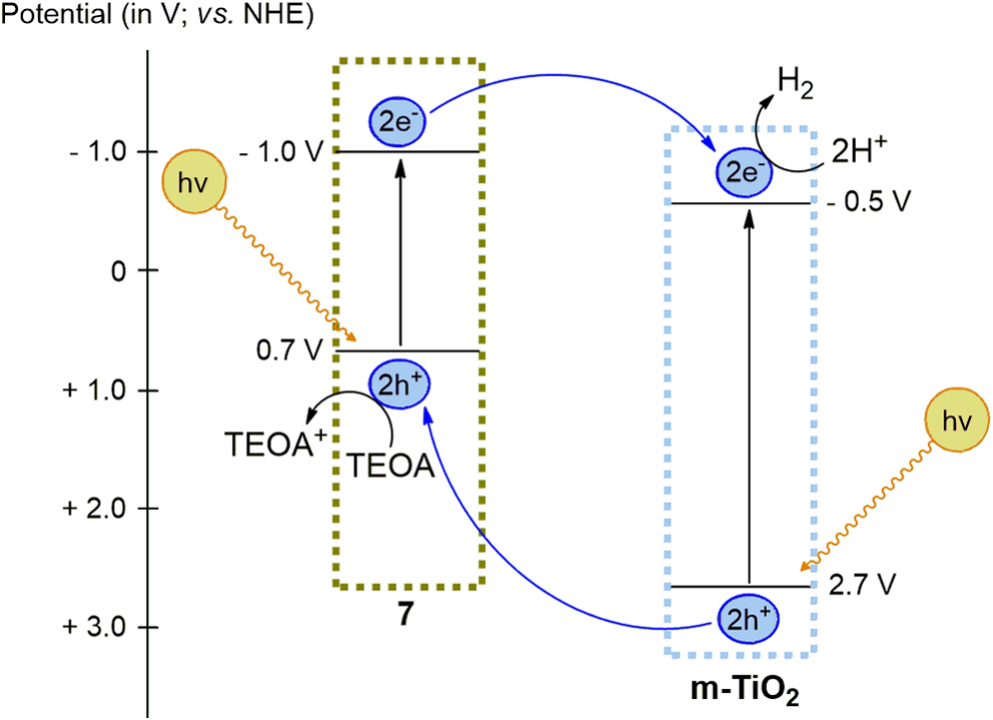
Proposed Electron Transfer Mechanism of m-TiO_2_-**7** in the Water Photolysis Process Using TEOA as the Sacrificial Agent

As previously noted, the Cu^II^ complexes were the most efficient in the H_2_(g) production tests, especially the photocatalyst m-TiO_2_-**7**. In this context, the synergism between m-TiO_2_ and complex **7** can be schematized, considering the potentials associated with the most favorable redox processes, as demonstrated in the proposal represented in Scheme 2.

Through the photoexcitation process, charge migration occurs in opposite directions: the photogenerated electrons (e^–^) migrate to m-TiO_2_, while the holes (h^+^) accumulate in compound **7**. Thus, the photocatalyst m-TiO_2_-**7** acts as an efficient charge separator, reducing the recombination of e^–^/h^+^ pairs and thereby enhancing photocatalytic reactions on the material’s surface.^22^ To support the proposed mechanism, solid-state photoluminescence (PL) spectroscopy (Figure S114–S115) was employed to evaluate the possible interaction between complex **7** and m-TiO_2_. It was observed that the ligand **L** and complex **7** do not exhibit a significant emission band compared to pure m-TiO_2_ and m-TiO_2_-**7** at approximately 320 nm, further supporting the possible electron transfer from **7** to m-TiO_2_.

The same charge transfer mechanism can be applied to the other photocatalysts, m-TiO_2_-**n** (**n** = **4**, **5**, and **10**), although their photocatalytic behavior differs significantly. According to literature, the redox potential of metal complexes plays a crucial role in photocatalytic efficiency.^23^ Therefore, the different redox potentials of our complexes may be responsible for the variations in performance observed. In particular, the energy of the frontier orbitals, especially the LUMO (Lowest Unoccupied Molecular Orbital), could influence the electron transfer mechanism.

Among the studied photocatalysts, m-TiO_2_-**7** exhibits the largest energy difference between the LUMO of complex **7** (−1.0 V) and the conduction band (CB) of TiO_2_ (−0.5 V), which could enhance electron transfer from the complex to TiO_2_. In contrast, m-TiO_2_-**4**, -**5**, and -**10** have smaller energy differences (LUMO ranging from −0.71 to −0.85 V), which, being closer to the TiO_2_ CB, may lead to less efficient charge separation compared to complex **7**. Meanwhile, the remaining complexes (**1**–**3**) have LUMO energies lower than −0.47 V, which could result in poor charge separation and, consequently, their low observed photocatalytic activity.

## Conclusions

In this work, ten metal complexes (**1–10**) derived from the novel ligand bis((3-aminopyridin-2-yl)selanyl)methane (**L**) and various metal centers (Co^II^, Cu^I^, Cu^II^, Zn^II^, and Ag^I^) were synthesized. The ligand and complexes were structurally characterized by SCXRD and extensively analyzed using complementary techniques. The more soluble compounds (**L**, **1**–**5**, **7**, and **10**) were employed as cocatalysts for m-TiO_2_. These photocatalysts were characterized and evaluated for photocatalytic hydrogen production via water photolysis under solar light simulation, using TEOA as a sacrificial agent. Photocatalytic tests revealed that m-TiO_2_**-4**, **-5**, and **-10** produced hydrogen gas efficiently–yielding approximately four to five times (1300–1600 μmol/g) more hydrogen than pure m-TiO_2_ (300 μmol/g). Notably, m-TiO_2_-**7** demonstrated exceptional catalytic activity by producing nearly 26 times more hydrogen (7800 μmol/g). Despite their very low loading values, the complexes significantly enhanced the photocatalytic performance of m-TiO_2_. Furthermore, complex **7** ([Cu_2_(μ-SO_4_)_2_**L**_**2**_]) highlighted the potential of organoselenium copper(II) complexes to dramatically improve m-TiO_2_ efficiency, underscoring the importance of exploring nonprecious metal complexes as cocatalysts. In summary, this study contributes to the synthesis and exploration of novel organoselenium-based metal complexes and their application as efficient cocatalysts in photocatalytic hydrogen production, fostering the development of new materials that advance sustainable energy solutions.

## Supporting Information Available

Material and methods; synthesis procedures and experimental details; X-ray diffraction (SCXRD and PXRD) data and structural projections; additional spectra (FT-IR, Raman, NMR, HRMS, UV–vis, CV, PL, and EDS) and images (SEM, photocatalysts, and photocatalytic system) (PDF)
