# Supplementary material for: Structural Analysis of Selenium Coordination Compounds and Mesoporous TiO2-Based Photocatalysts for Hydrogen Generation
Source: Inorg Chem. 2025 Apr 16;64(16):7902–19. doi: 10.1021/acs.inorgchem.4c05325 (PMC12042259; doi:10.1021/acs.inorgchem.4c05325)
Supplement: Supplementary file 1 — ic4c05325_si_001.pdf [file ic4c05325_si_001.pdf]

# Supporting Information

# Structural Analysis of Selenium Coordination Compounds and Mesoporous $TiO_2$-Based Photocatalysts for Hydrogen Generation

Rodrigo Cervo,*[,a] Cândida Alíssia Brandl,[a] Tanize Bortolotto,[a] Camila Nunes Cechin,[a] Natália de Freitas Daudt,[b] Bernardo Almeida Iglesias,[a] Ernesto Schulz Lang,[a] Bárbara Tirloni,[a] and Roberta Cargnelutti*[,a]

[a] Department of Chemistry, Federal University of Santa Maria (UFSM), Av. Roraima, n.1000, 97105-900, Santa Maria, RS, Brazil.

[b] Department of Mechanical Engineering, Federal University of Santa Maria (UFSM), Av. Roraima, n.1000, 97105-900, Santa Maria, RS, Brazil.

Corresponding Authors

*R. Cervo: rodrigo.cervo@acad.ufsm.br and R. Cargnelutti: roberta.cargnelutti@ufsm.br.

SUMMARY

**CHARACTERIZATION METHODS** ..... 7
**Single crystal X-ray diffraction (SCXRD)** ..... 7
**Powder X-ray diffraction (PXRD)** ..... 7
**Fourier transform infrared spectroscopy (FT-IR)** ..... 7
**Confocal Raman spectroscopy** ..... 7
**Nuclear magnetic resonance spectroscopy (NMR)** ..... 7
**Ultraviolet-visible (UV-Vis) spectroscopy in solution, and determination of molar absorptivity values (ε)** ..... 8
**UV-Vis diffuse reflectance spectroscopy (DRS) in the solid state, and determination of energy gap values ($E_g$)** ..... 8
**High-resolution mass spectrometry (HRMS)** ..... 9
**Cyclic voltammetry (CV)** ..... 9
**Elemental analysis (CHN)** ..... 9
**Melting point determination (m.p.)** ..... 9
**Scanning electron microscopy (SEM) and Energy-dispersive X-ray spectroscopy (EDS)** ..... 9
**Solid-state photoluminescence spectroscopy (PL)** ..... 9
**Textural characterization of m-$TiO_2$** ..... 9
**PHOTOCATALYSIS** ..... 10
**Photocatalytic system** ..... 10
**Figure S1.** Photocatalytic system. Legend: (O) quartz reactor, (□) solar simulator, (Δ) intensity controller, (◊) gas chromatograph. ..... 10
**Protocol for hydrogen production tests** ..... 10
**Impregnation process of m-$TiO_2$** ..... 10
**SYNTHESIS PROCEDURES** ..... 11
**1,2-bis(3-aminopyridine)diselane (3-apySe)$_2$** ..... 11
**Ligand bis((3-aminopyridin-2-yl)selenyl)methane (L)** ..... 11
**General synthesis procedure for complexes 1–10** ..... 12
**Table S1.** Synthesis conditions for complexes **1–10**. ..... 12
**Complex 1: [$CoCl_2L$]** ..... 12
**Complex 2: [$CoBr_2L$]** ..... 13
**Complex 3: [$ZnCl_2L$]** ..... 13
**Complex 4: [$CuCl_2L$]** ..... 13
**Complex 5: [$CuBr_2L$]·0,5$CHCl_3$** ..... 14
**Complex 6: [$Cu_2(\mu\text{-}I)_2L$]$_n$** ..... 14
**Complex 7: [$Cu_2(\mu\text{-}SO_4)_2L_2$]** ..... 14
**Complex 8: [$Zn_2(\mu\text{-}SO_4)_2L_2$]** ..... 14
**Complex 9: [$Ag_2L_2$]$SO_4$·7$H_2O$** ..... 15
**Complex 10: [$Ag_2L_2$]$(NO_3)_2$·2$H_2O$** ..... 15
**Mesoporous titanium dioxide (m-$TiO_2$)** ..... 15
**SINGLE CRYSTAL X-RAY DIFFRACTION (SCXRD)** ..... 17
**Table S2.** Diffraction intensity collection and refinement data for ligand **L** and complexes **1** and **2**. 17
**Table S3.** Diffraction intensity collection and refinement data for complexes **3**, **4**, and **5**. ..... 18

**Table S4.** Diffraction intensity collection and refinement data for complexes **6**, **7**, and **8**. .............. 19
**Table S5.** Diffraction intensity collection and refinement data for complexes **9** and **10**. ................. 20
**Figure S2.** Structural projection of the two independent units of the complexes: (a) **1** (**1A** and **1B**), (b) **2** (**2A** and **2B**), and (c) **3** (**3A** and **3B**). Anisotropic displacement parameters are shown with 50% occupancy probability. ........................................................................................................ 21
**Figure S3.** Structural projection of the complexes: (a) **4** and (b) the two independent units of **5** (**5A** and **5B**). Anisotropic displacement parameters are shown with 50% occupancy probability. .......... 22
**Figure S4.** Structural projection of polymer **6** along the crystallographic direction [100]. Anisotropic displacement parameters are shown with 50% occupancy probability. Symmetry operations: ' = (3/2 - x, ½ + y, z); " = (3/2 - x, -1/2 + y, z). ........................................................................................ 22
**Figure S5.** Structural projection of the complexes: (a) **7** and (b) **8**. Anisotropic displacement parameters are shown with 50% occupancy probability. Symmetry operations: (**7**) ' = (1 - x, 1 - y, 2 - z); (**8**) ' = (1 - x, 2 - y, 1 - z). .......................................................................................... 23
**Figure S6.** Structural projection of ligand **L**. Anisotropic displacement parameters are shown with 50% occupancy probability. ............................................................................................... 23
**Figure S7.** Structural projection of the complexes: (a) **9** and (b) **10**. Anisotropic displacement parameters are shown with 50% occupancy probability. Symmetry operation: ' = (-x, 1 - y, 1 - z). . 24
**Table S6.** EA results for complexes **9** and **10**. ............................................................................ 24
**Table S7.** Selected bond lengths and angles for complexes **1–4**. Standard deviations are given in parentheses. ........................................................................................................................ 25
**Table S8.** Selected bond lengths and angles for complexes **5–8**. Standard deviations are given in parentheses. ........................................................................................................................ 26
**Table S9.** Selected bond lengths and angles for complexes **9** and **10**. Standard deviations are given in parentheses. ..................................................................................................................... 27
**Table S10.** Geometries of the metal centers of complexes **1–10**, assigned based on the calculations of the parameters $\tau_4$ and $\tau_5$. .......................................................................................... 27
**Figure S8.** Polyhedral representation of the geometries of the metal centers of the complexes: (a) **1**; (b) **2**; (c) **3**; (d) **4**; (e) **5**; (f) **6**; (g) **7**; (h) **8**; (i) **9**; (j) **10**. For better clarity, only one independent unit of complexes **1–3**, **5**, and one polyhedron from complex **6** are shown. ............................................... 28
**POWDER X-RAY DIFFRACTION (PXRD)** ........................................................................... **29**
**Figure S9.** Theoretical and experimental powder diffractograms of ligand **L**. ................................ 29
**Figure S10.** Theoretical and experimental powder diffractograms of complex **1**. ........................... 29
**Figure S11.** Theoretical and experimental powder diffractograms of complex **2**. ........................... 30
**Figure S12.** Theoretical and experimental powder diffractograms of complex **3**. ........................... 30
**Figure S13.** Theoretical and experimental powder diffractograms of complex **4**. ........................... 31
**Figure S14.** Theoretical and experimental powder diffractograms of complex **5**. ........................... 31
**Figure S15.** Theoretical and experimental powder diffractograms of complex **6**. ........................... 32
**Figure S16.** Theoretical and experimental powder diffractograms of complex **7**. ........................... 32
**Figure S17.** Theoretical and experimental powder diffractograms of complex **9**. ........................... 33
**Figure S18.** Theoretical and experimental powder diffractograms of complex **10**. ......................... 33
**VIBRATIONAL SPECTROSCOPY (FT-IR AND RAMAN)** ...................................................... **34**
**Table S11.** Main bands observed (in $cm^{-1}$) in the FT-IR spectra of ligand **L** and complexes **1–10**. . 34

**Table S12.** Main bands observed (in $cm^{-1}$) in the Raman spectra of ligand **L** and complexes **1–10**. 35
**FT-IR spectra** .... **36**
**Figure S19.** FT-IR spectrum of **(3-apySe)$_2$**. .... 36
**Figure S20.** FT-IR spectrum of ligand **L**. .... 36
**Figure S21.** FT-IR spectrum of complex **1**. .... 37
**Figure S22.** FT-IR spectrum of complex **2**. .... 37
**Figure S23.** FT-IR spectrum of complex **3**. .... 38
**Figure S24.** FT-IR spectrum of complex **4**. .... 38
**Figure S25.** FT-IR spectrum of complex **5**. .... 39
**Figure S26.** FT-IR spectrum of complex **6**. .... 39
**Figure S27.** FT-IR spectrum of complex **7**. .... 40
**Figure S28.** FT-IR spectrum of complex **8**. .... 40
**Figure S29.** FT-IR spectrum of complex **9**. .... 41
**Figure S30.** FT-IR spectrum of complex **10**. .... 41
**Raman spectra** .... **42**
**Figure S31.** Raman spectrum of **(3-apySe)$_2$**. .... 42
**Figure S32.** Raman spectrum of ligand **L**. .... 42
**Figure S33.** Raman spectrum of complex **1**. .... 43
**Figure S34.** Raman spectrum of complex **2**. .... 43
**Figure S35.** Raman spectrum of complex **3**. .... 44
**Figure S36.** Raman spectrum of complex **4**. .... 44
**Figure S37.** Raman spectrum of complex **5**. .... 45
**Figure S38.** Raman spectrum of complex **6**. .... 45
**Figure S39.** Raman spectrum of complex **7**. .... 46
**Figure S40.** Raman spectrum of complex **8**. .... 46
**Figure S41.** Raman spectrum of complex **9**. .... 47
**Figure S42.** Raman spectrum of complex **10**. .... 47
**NUCLEAR MAGNETIC RESONANCE (NMR)** .... **48**
**1D NMR spectra** .... **48**
**Figure S43.** $^{1}H$ NMR (400 MHz, DMSO-$d_6$) spectrum of **(3-apySe)$_2$**. .... 48
**Figure S44.** $^{13}C$ NMR (100 MHz, DMSO-$d_6$) spectrum of **(3-apySe)$_2$**. .... 48
**Figure S45.** $^{77}Se$ NMR (76 MHz, DMSO-$d_6$) spectrum of **(3-apySe)$_2$**. .... 49
**Figure S46.** $^{77}Se$ NMR (76 MHz, DMSO-$d_6$) spectrum of ligand **L**. .... 49
**Figure S47.** $^{1}H$ NMR (400 MHz, DMSO-$d_6$) spectrum of complex **3**. .... 50
**Figure S48.** $^{13}C$ NMR (100 MHz, DMSO-$d_6$) spectrum of complex **3**. .... 50
**Figure S49.** $^{77}Se$ NMR (76 MHz, DMSO-$d_6$) spectrum of complex **3**. .... 51
**2D NMR spectra** .... **51**
**Figure S50.** COSY NMR spectrum of ligand **L**. .... 51
**Figure S51.** COSY NMR spectrum of ligand **L** (expansion). .... 52
**Figure S52.** HSQC NMR spectrum of ligand **L**. .... 52
**Figure S53.** HSQC NMR spectrum of ligand **L** (first expansion). .... 53
**Figure S54.** HSQC NMR spectrum of ligand **L** (second expansion). .... 53

**Figure S55.** HMBC NMR spectrum of ligand **L**. 54
**Figure S56.** HMBC NMR spectrum of ligand **L** (first expansion). 54
**Figure S57.** HMBC NMR spectrum of ligand **L** (second expansion). 55
**Figure S58.** HMBC NMR spectrum of ligand **L** (third expansion). 55
**Figure S59.** COSY NMR spectrum of complex **3**. 56
**Figure S60.** HSQC NMR spectrum of complex **3**. 56
**Figure S61.** HMBC NMR spectrum of complex **3**. 57
**HIGH-RESOLUTION MASS SPECTROMETRY (HRMS) 57**
**Figure S62.** HRMS spectrum of complex **1**. 57
**Figure S63.** HRMS spectrum of complex **1** (expansion). 58
**Figure S64.** HRMS spectrum of complex **2**. 58
**Figure S65.** HRMS spectrum of complex **2** (expansion). 59
**Figure S66.** HRMS spectrum of complex **3**. 59
**Figure S67.** HRMS spectrum of complex **3** (expansion). 60
**Figure S68.** HRMS spectrum of complex **4**. 60
**Figure S69.** HRMS spectrum of complex **4** (expansion). 61
**Figure S70.** HRMS spectrum of complex **5**. 61
**Figure S71.** HRMS spectrum of complex **5** (expansion). 62
**Figure S72.** HRMS spectrum of complex **6**. 62
**Figure S73.** HRMS spectrum of complex **7**. 63
**Figure S74.** HRMS spectrum of complex **8**. 63
**Figure S75.** HRMS spectrum of complex **9**. 64
**Figure S76.** HRMS spectrum of complex **9** (first expansion). 64
**Figure S77.** HRMS spectrum of complex **9** (second expansion). 65
**Figure S78.** HRMS spectrum of complex **10**. 65
**Figure S79.** HRMS spectrum of complex **10** (first expansion). 66
**Figure S80.** HRMS spectrum of complex **10** (second expansion). 66
**ULTRAVIOLET-VISIBLE SPECTROSCOPY (UV-VIS) 67**
**Figure S81.** UV-Vis spectra of complexes **7** and **8** in DMF. 67
**Stability in solution over time 67**
**Figure S82.** Stability in solution over time of ligand **L** and complex **1**. 67
**Figure S83.** Stability in solution over time of complexes **2–7**. 68
**Figure S84.** Stability in solution over time of complexes **8–10**. 69
**Solid-state spectra (DRS) and correspondingTauc plots 69**
**Figure S85.** DRS spectrum and Tauc plot of m-$TiO_2$. 69
**Figure S86.** DRS spectra and Tauc plots of ligand **L** and complexes **1** and **2**. 70
**Figure S87.** DRS spectra and Tauc plots of complexes **3–5**. 71
**Figure S88.** DRS spectra and Tauc plots of complexes **6–8**. 72
**Figure S89.** DRS spectra and Tauc plots of complexes **9** and **10**. 73
**Spectra of the dilutions and their respective straight-line equation graphs 73**
**Figure S90.** Dilution series and straight-line graph of ligand **L**. 73
**Figure S91.** Dilutions series and straight-line graphs of ligand **L** and complex **1**. 74
**Figure S92.** Dilutions series and straight-line graphs of complexes **2** and **3**. 75

**Figure S93.** Dilutions series and straight-line graphs of complexes **3** and **4**. ................................. 76
**Figure S94.** Dilutions series and straight-line graphs of complexes **5** and **7**. ................................. 77
**Figure S95.** Dilution series and straight-line graph of complex **7**. .................................................. 78
**CYCLIC VOLTAMMETRY (CV)** ........................................................................................ **78**
**Figure S96.** Voltammograms of ligand **L** and complexes **1–3**. ...................................................... 78
**Figure S97.** Voltammograms of complexes **4**, **5**, **7**, and **10**. ........................................................ 79
**CHARACTERIZATION OF THE PHOTOCATALYSTS** ..................................................................... **80**
**Figure S98.** Photocatalysts m-$TiO_2$-**n**. ............................................................................................ 80
**Figure S99.** Theoretical and experimental powder diffractograms of m-$TiO_2$. ................................ 80
**Figure S100.** Powder diffractograms of the photocatalysts. ............................................................. 81
**Figure S101.** Raman spectrum of m-$TiO_2$-**10**. ................................................................................. 81
**Figure S102.** DRS spectra of the photocatalysts. ............................................................................. 82
**Figure S103.** Absorbance spectra of the photocatalysts calculated from DRS data. ...................... 82
**Figure S104.** Scanning electron microscopy (SEM) images of the photocatalysts. ......................... 83
**Figure S105.** Energy-dispersive X-ray spectroscopy (EDS) spectrum of m-$TiO_2$. .......................... 83
**Figure S106.** EDS spectra of the photocatalysts. .............................................................................. 84
**Figure S107.** Elemental mapping of m-$TiO_2$. ..................................................................................... 85
**Figure S108.** Elemental mapping of m-$TiO_2$-**4**. ................................................................................ 86
**Figure S109.** Elemental mapping of m-$TiO_2$-**5**. ................................................................................ 87
**Figure S110.** Elemental mapping of m-$TiO_2$-**10**. .............................................................................. 88
**Figure S111.** FT-IR and Raman spectra of m-$TiO_2$-**7** post-photocatalysis. .................................... 89
**Figure S112.** EDS spectrum of m-$TiO_2$-**7** post-photocatalysis. ....................................................... 89
**Figure S113.** Elemental mapping of m-$TiO_2$-**7** post-photocatalysis. ............................................... 90
**Figure S114.** Solid-state photoluminescence (PL) emission spectra of ligand **L**, complex **7**, m-$TiO_2$, and m-$TiO_2$-**7**, along with their respective excitation wavelengths ($\lambda_{exc}$). The $\lambda_{exc}$ values were chosen based on the maximum absorbance wavelengths observed in the absorbance spectra obtained from DRS data. ........................................................................................................................... 91
**Figure S115.** PL emission spectra of ligand **L** and complex **7** at different $\lambda_{exc}$. ............................... 92
**REFERENCES** ............................................................................................................ **93**

## CHARACTERIZATION METHODS

### Single crystal X-ray diffraction (SCXRD)

The data collection for the monocrystals of ligand **L** and complexes **1**–**10** were carried out on a Bruker D8 Venture diffractometer using Mo K$\alpha$ radiation (0.71073 Å) or Bruker D8 Quest with Cu K$\alpha$ radiation (1.54178 Å). The structures were solved using the Intrinsic Phasing method with the ShelXle program.[1a] Refinements were performed using the ShelX program package through full matrix/least squares of the structural factors $F^2$, with anisotropic thermal displacement parameters for all non-hydrogen atoms.[1b] Hydrogen atoms were included in the refinement in calculated positions based on the complete geometry of the molecular fragments, as groups attached to the corresponding non-hydrogen atoms. The graphical representations of the crystalline structures were generated using the Diamond program.[1c]

### Powder X-ray diffraction (PXRD)

The powder X-ray diffraction data were collected using a Bruker D8 Advance diffractometer equipped with a LynxEye detector under the following conditions: Cu K$\alpha$ radiation ($\lambda$ = 1.54056 Å) with 0.5 mm nickel filter; an increment of 0.02°; a counting time of 0.3 s per step; data collected from 6° to 60° 2$\theta$ with $\theta$-$\theta$ geometry at room temperature; Bragg-Brentano diffraction mode; operating at 40 kV and 40 mA; a divergence slit of 0.2°; a second Soller slit of 2.5°; and receiving slit of 8 mm.

The theoretical diffractograms were generated, and the crystallographic plane indexing was performed using the Mercury 4.2.0 program,[2] based on data obtained by SCXRD. For the photocatalysts, literature data corresponding to m-$TiO_2$ (CCDC identifier: 1671224)[3] were used as a theoretical standard. The average size was calculated using Scherrer's equation.[4]

### Fourier transform infrared spectroscopy (FT-IR)

Spectral data in the infrared region were obtained using a Bruker Vertex 70 spectrometer. The analyses were performed in attenuated total reflectance (ATR) mode using a Platinum ATR accessory with a diamond crystal, coupled to the spectrometer. All spectra were recorded in the spectral range of 30–4000 $cm^{-1}$, and all measurements were conducted directly on small amounts of sample. The following symbology was adopted for the assignment of vibrational modes in the interpretation of the spectra: $\nu$ = stretching; $\nu_a$ = asymmetric stretching; $\nu_s$ = symmetric stretching; $\delta$ = bending; $\delta_{ip}$ = in-plane bending; $\delta_{oop}$ = out-of-plane bending; $\delta_{ot}$ = overtone bending.

### Confocal Raman spectroscopy

The measurements by confocal Raman spectroscopy were performed using a Bruker Senterra spectrometer equipped with an Olympus objective lens (20x magnification) and 785 nm laser. The laser power, spectral window, number of co-additions, and exposure time were based on the sensitivity of each sample to the technique. The same symbology as in the FT-IR spectra was adopted for the assignments.

### Nuclear magnetic resonance spectroscopy (NMR)

The 1D ($^1$H, $^{13}$C and $^{77}$Se) and 2D (COSY, HSQC and HMBC) NMR spectral data were obtained using a Bruker Avance III HD 400 spectrometer. The spectral were recorded at frequencies of 400 MHz for $^1$H, 100 MHz for $^{13}$C,

and 76 MHz for $^{77}Se$. Tetramethylsilane (TMS) was used as an internal standard for $^{1}H$ and $^{13}C$ nuclei, while **(3-apySe)$_2$** in DMSO-$d_6$ was used as an external standard for $^{77}Se$. Ligand **L** and complex **3** were dissolved in DMSO-$d_6$, and measurements were performed at 298 K. Chemical shifts ($\delta$) are expressed in parts per million (ppm), and the coupling constants ($J$) in hertz (Hz). The chemical shift in the $^{77}Se$ spectrum for **(3-apySe)$_2$** in DMSO-$d_6$ was referenced using a solution of 1,2-diphenyldiselenane in DMSO-$d_6$ (470 ppm) as an external standard.[5] The following symbology was adopted for signals multiplicities: s (singlet); dd (doublet of doublets).

**Ultraviolet-visible (UV-Vis) spectroscopy in solution, and determination of molar absorptivity values ($\varepsilon$)**

Spectral data in the UV-Vis region were obtained using a Shimadzu UV-Vis 2600 spectrophotometer in DMF or DMSO. The analyses were performed using 1.00 cm quartz cuvettes. The concentration of the stock solution was set as $1.00 \cdot 10^{-3}$ M, followed by dilution to $0.0100 \cdot 10^{-3}$ M. DMF was used as the standard solvent for solution preparation, as it was also used in the synthesis of the photocatalysts. When the complex was not fully soluble in DMF, DMSO was used as an alternative solvent. The of the stability of the complexes in solution was evaluated by measuring absorbance over time, with data collected every 30 minutes for 6 hours. All measurements were performed in the spectral range of 200–800 nm. The following symbology was adopted symbologies: $\lambda$ = wavelength; $\lambda_{max}$ = wavelength referring to maximum absorption; $\varepsilon_{max}$ = molar absorptivity coefficient of $\lambda_{max}$. The $\varepsilon_{max}$ values were calculated using the Lambert-Beer law ($A = \varepsilon\, b\, c$).

To determine molar absorptivity ($\varepsilon$), solutions of precise molar concentration were prepared. As a standard for the initial solution preparation, 5.00 mg of the compound was dissolved in DMF, and volumes measured in 25.0 mL volumetric flasks. A series of dilutions were then performed, and their spectra were recorded to construct A vs. C (absorbance vs. concentration) plots. From these data, absorbance values at specific wavelengths ($\lambda$) were extracted from the spectra ($\lambda$ varies according to the sample). The $\varepsilon$ values for each compound were determined from the slope of A vs. C plot at specific $\lambda$. All experiments were conducted in duplicate, and the average of the values was used for subsequent calculations.

**UV-Vis diffuse reflectance spectroscopy (DRS) in the solid state, and determination of energy gap values ($E_g$)**

Analyses in the UV-Vis region in the solid state were carried out using a Shimadzu UV-Vis 2600 spectrophotometer, equipped with an ISR-2600Plus integrating sphere. The diffuse reflectance spectra of the compounds were obtained in the spectral range of 220–800 nm, using a $BaSO_4$ pellet as a reference.

The energy gap ($E_g$) of the samples was estimated from the diffuse reflectance spectra. The data were transformed from reflectance to absorbance through the Kubelka-Munk function: $F(R) = (\alpha/s) = (1 - r)^2/(2r)$, where the absorption coefficient of the material ($\alpha$) is related to the diffuse reflectance of the sample (r) and the scattering coefficient of the material (s). The sample was considered as a continuous powder layer of infinite thickness, with light scattering independent of $\lambda$.[6] The $E_g$ values were estimated through Tauc plots of the Kubelka-Munk function in the format $(F(R)h\nu)^2$ vs energy ($h\nu$, in eV), where the linear portion of the graph was extrapolated to the x-axis (considering the first ascending curve as being the first allowed optical transition). Assuming that $\alpha$ is dependent on energy, it can be expressed by the relation: $(\alpha\, h\nu)^2 = B(h\nu - E_g)$, where (h) is Planck's constant, ($\nu$) is the photon frequency, and (B) a constant.[6c, 7] All calculations were carried out using the OriginPro 2018 software, version 9.5.1.195.[8]

**High-resolution mass spectrometry (HRMS)**

HRMS data of the complexes were obtained using an Agilent 6210 ESI-TOF instrument in positive mode ($ESI^+$), detecting cationic fragments. The complexes were solubilized in DMF or DMSO, and the data are presented as the mass-to-charge ratio (m/z). Fragment assignment was performed with the aid of theoretical isotopic standards generated by the mMass 5.5.0 program and compared to the experimental data.[9]

**Cyclic voltammetry (CV)**

CV analyses were performed using a Metrohm Eco Chemie AutoLab PGSTAT128N potentiostat/galvanostat. Voltammograms were recorded in DMF at room temperature and under an open atmosphere, with scan rates ranging from 100 to 200 mV $s^{-1}$. Tetrabutylammonium hexafluorophosphate ($Bu_4NPF_6$) was used as a supporting electrolyte (approximately 0.100 mol $L^{-1}$ in DMF). A standard three-component system was used, consisting of a glassy carbon working electrode, a platinum pseudo-reference electrode, and a platinum counter (auxiliary) electrode. Redox potentials were corrected using ferrocene (redox pair $Fc/Fc^+$) as an internal standard to calibrate the pseudo-reference electrode ($E_{1/2}$ = +0.40 V in DMF), and the results were expressed relative to the normal hydrogen electrode (NHE; $Fc/Fc^+$ vs. NHE = +0.63 V).[10]

**Elemental analysis (CHN)**

The percentage composition of C, H, and N was determined using a PerkinElmer CHN 2400 elemental analyzer, with measurements performed in duplicate.

**Melting point determination (m.p.)**

The melting points of the compounds were determined in triplicate using a MicroQuímica MQAPF-301 digital melting point apparatus, which operates in the range of 0–360 °C. The results were not corrected.

**Scanning electron microscopy (SEM) and Energy-dispersive X-ray spectroscopy (EDS)**

The SEM and EDS data of the m-$TiO_2$-**n** photocatalysts were obtained using a Bruker Quantax 200 spectrometer coupled to a JEOL JSM-6360 scanning electron microscope, operating at 20 kV. The samples were previously deposited on brass sample holders with carbon adhesive tape and metallized with gold using a Denton Desk II sputter coater.

**Solid-state photoluminescence spectroscopy (PL)**

The PL spectra were recorded using a Horiba FluoroMax Plus spectrofluorometer in the spectral range of 350–600 nm, with excitation and emission slit widths of 5.0 nm.

**Textural characterization of m-$TiO_2$**

The textural characterization of m-$TiO_2$ was performed by $N_2$(g) adsorption/desorption analyses, using the BET method to determine surface area and average pore size, and the BJH method to determine average pore volume. Data on the textural properties of m-$TiO_2$ were collected using a Micromeritics Surface ASAP 2020N Area and Porosity Analyzer. The samples were previously dried at 150–250 °C under vacuum.

## PHOTOCATALYSIS

### Photocatalytic system

Photocatalytic activity tests were carried out using the system represented below. Hydrogen gas ($H_2(g)$) production from photolysis of water was recorded using a Shimadzu CG-2014 gas chromatograph. A solar simulator equipped with a Xe/Hg lamp (300 W, 1370 W/m$^2$) was used as the radiation source. The quartz reactor, where the sample was placed, featured a cooling jacket and had an internal tube diameter of 24.0 mm.

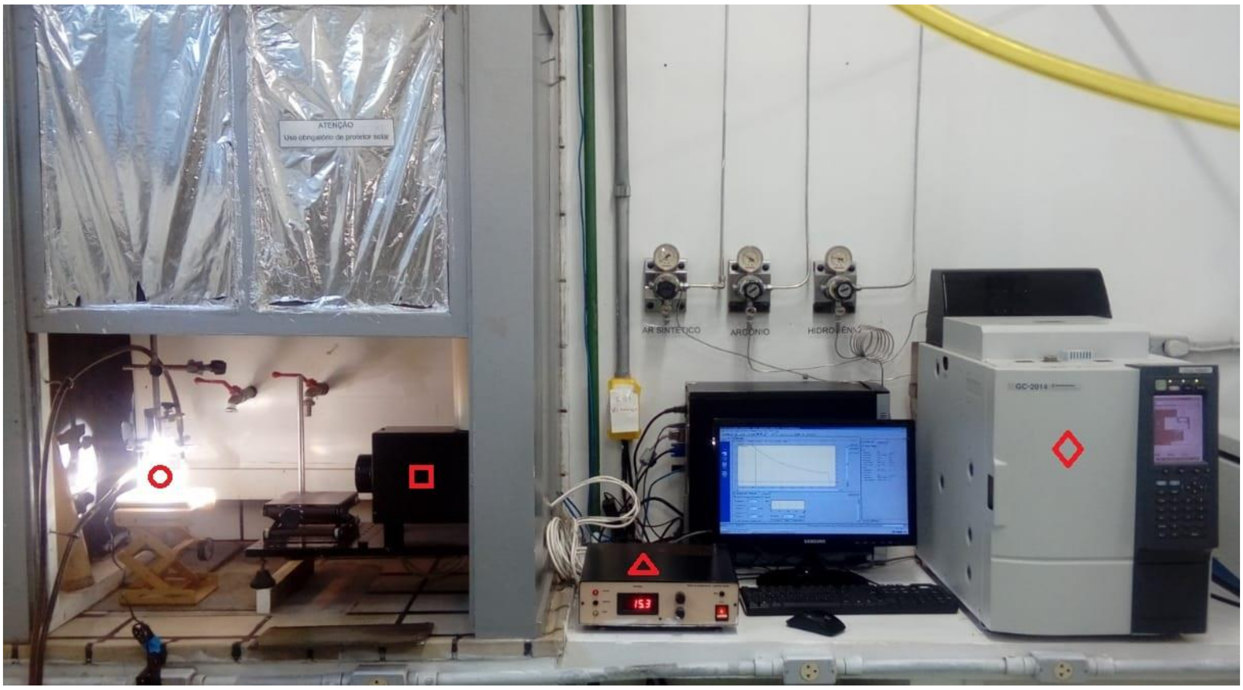

**Figure S1.** Photocatalytic system. Legend: (O) quartz reactor, (□) solar simulator, (Δ) intensity controller, (◇) gas chromatograph.

### Protocol for hydrogen production tests

First, 1.80 mL of triethanolamine (sacrificial reagent), 18.2 mL of deionized water, and 10.0 mg of the desired photocatalyst (impregnated m-$TiO_2$) were added to a flask. The mixture was briefly sonicated in a water bath and then transferred to the quartz reactor. The system was sealed and purged with argon gas for 10 min to remove any residual gases, especially $O_2(g)$. After purging, a 300 µL gas aliquot was collected from inside the reactor using an analytical syringe and injected into the chromatograph to confirm the absence of $H_2(g)$ before the start of the experiment. The reactor was then exposed to the light beam generated by the solar light simulator for 6 h. During exposure, 300 µL gas aliquots were collected and injected into the chromatograph every one hour to monitor $H_2(g)$ production. Throughout the process, the mixture inside in the reactor was kept under constant agitation to ensure system homogeneity.

### Impregnation process of m-$TiO_2$

For photocatalyst preparation, the standard ratio of 0.0100 g of the complex and 0.200 g of m-$TiO_2$ (5% mass/mass) was used. Initially, the desired mass of the complex was weighed and transferred to a light-protected flask equipped

with a magnetic stirring system. Then, 1.50 mL of DMF was added and stirred until the compound was completely dissolved. Subsequently, the weighed m-$TiO_2$ was added, and the system was kept under stirring for 72 h. After the designated time, the suspension was completely transferred to a pre-weighed Falcon® tube, shaken using a vortex shaker, and centrifuged (5000 rpm for 10 min) to remove any unimpregnated complex. The supernatant was collected, and the agitation and centrifugation steps were repeated until the solution became completely colorless. At the end of the process, the impregnated m-$TiO_2$ was retained in the tube for subsequent vacuum drying. The collected supernatant fractions were combined in a 50 mL volumetric flask, brought to volume with DMF, and analyzed by UV-Vis spectroscopy in triplicate. The absorbance results were used to calculate the impregnation efficiency and the complex loading as a cocatalyst for m-$TiO_2$.

## SYNTHESIS PROCEDURES

### 1,2-bis(3-aminopyridine)diselane (3-apySe)$_2$

The synthesis was performed according to the literature:[11] in a 100 mL two-necked flask equipped with a magnetic stirring system and reflux condenser, 40.0 mL of PEG 400 and elemental selenium (12.7 mmol; 1.00 g) were added under an argon atmosphere and heated to 50 °C. Sodium tetrahydridoborate (23.2 mmol; 0.878 g) was then added, and selenium was reduced for 2 h. Meanwhile, a mixture of 3-amino-2-chloropyridine (10.6 mmol; 1.36 g), p-toluenesulfonic acid monohydrate (21.1 mmol; 4.01 g) and 20.0 mL of PEG 400 was prepared in a beaker. After the selenium reduction, this mixture was added to the reaction system and stirred for 24 h. The reaction medium was then neutralized with a 1.00 M sodium bicarbonate solution and stirred under ambient conditions for 1 h.
Next, 100 mL of deionized water was added, and the mixture was extracted approximately 15 times, with 200 mL of ethyl acetate. After each extraction, the organic solvent was removed using a rotary evaporator for reuse in subsequent extractions. This intensive extraction procedure was necessary due to the amphiphilic behavior of PEG. Finally, the organic phase was dried over $MgSO_4$, and the solvent was removed using a rotary evaporator, yielding the product as an orange solid. The product was washed with ethyl acetate to remove residual of PEG.
$C_{10}H_{10}N_4Se_2$: 344.13 g·mol$^{-1}$; orange crystalline solid, stable. Yield: 70.0% (1.28 g) based on 3-amino-2-chloropyridine. m.p. = 188 – 190 °C. FT-IR (cm$^{-1}$): $\nu_a(NH_2)$ = 3438; $\nu_s(NH_2)$ = 3269; $\delta_{ot}(NH_2)$ = 3132; $\nu$(=C–H) = 3047; $\nu$(C=N) = 1612; $\nu$(C=C) = 1572, 1456, 1417; $\nu(C–NH_2)$ = 1319; $\delta_p(NH_2)$ = 1045; $\delta$(=C–H) = 793; $\delta_{oop}(NH_2)$ = 663; $\delta_p(C–NH_2)$ = 444. Raman (cm$^{-1}$): $\nu$(C=N) = 1623; $\nu$(C=C) = 1573, 1458, 1418; $\nu(C–NH_2)$ = 1325; $\delta_{ip}(NH_2)$ = 1038; $\delta$(=C–H) = 794; $\delta_{oop}(NH_2)$ = 663; $\nu$(C–Se) = 555; $\delta_{ip}(C–NH_2)$ = 451; $\nu$(Se–Se) = 255. $^1$H NMR (400 MHz, DMSO-$d_6$), $\delta$(ppm): 7.74 (dd; *J* = 4.4 and 1.6 Hz; 2H); 7.06 (dd; *J* = 8.0 and 4.4 Hz; 2H); 7.00 (dd; *J* = 8.1 and 1.7 Hz; 2H); 5.78 (s, 4H); $^{13}$C NMR (100 MHz, DMSO-$d_6$), $\delta$(ppm): 145.6; 137.4; 135.8; 124.4; 120.7; $^{77}$Se NMR (76 MHz, DMSO-$d_6$), $\delta$(ppm): 438.

### Ligand bis((3-aminopyridin-2-yl)selenyl)methane (L)

The reaction procedure was based on the literature:[12] in a 100 mL two-necked flask equipped with a magnetic stirring system, 25.0 mL of EtOH and **(3-apySe)$_2$** (3.00 mmol; 1.03 g) were added under an argon atmosphere and cooled to 0 °C using an ice bath. Subsequently, sodium tetrahydridoborate (8.40 mmol; 0.318 g) was added, followed by another 25.0 mL of EtOH. The reduction was carried out at low temperature for 30 min. After this period,

the cooling was removed and dibromomethane (3.15 mmol; 0.220 mL) was added to the system. The reaction was monitored by TLC. After 3 h, the system was neutralized with a saturated aqueous $NH_4Cl$ solution and diluted in deionized water. The aqueous phase was extracted four times (30.0 mL each) with ethyl acetate. The organic phase was dried over $MgSO_4$, and the solvent was removed using a rotary evaporator, yielding an orange solid. The crude product was purified by column chromatography, using ethyl acetate/hexane (60/40%) as eluents. The ligand **L** was obtained as a crystalline orange solid, with a yield of 84.0%. A fraction of the product was recrystallized in THF to obtain single crystals suitable for SCXRD measurements.

$C_{11}H_{12}N_4Se_2$: 358.16 g·mol$^{-1}$; orange crystalline solid, stable. Yield: 84.0% (0.906 g) based on **(3-apySe)$_2$**. m.p. = 98 – 100 °C. EA (%): theoretical = C: 36.89; H: 3.38; N: 15.64; experimental = C: 37.38; H: 3.38; N: 15.37. FT-IR (cm$^{-1}$): $\nu_a(NH_2)$ = 3389; $\nu_s(NH_2)$ = 3295; $\delta_{ot}(NH_2)$ = 3175; $\nu$(=C–H) = 3053; $\nu_a(CH_2)$ = 3019; $\nu_s(CH_2)$ = 2920; $\nu$(C=N) = 1621; $\nu$(C=C) = 1561, 1449, 1423; $\nu(C–NH_2)$ = 1306; $\delta_{ip}(NH_2)$ = 1045; $\delta$(=C–H) = 789; $\delta_{oop}(NH_2)$ = 663; $\nu(H_2C–Se)$ = 549; $\delta_{ip}(C–NH_2)$ = 442. Raman (cm$^{-1}$): $\nu$(C=N) = 1623; $\nu$(C=C) = 1570, 1448, 1425; $\nu(C–NH_2)$ = 1305; $\delta_{ip}(NH_2)$ = 1047; $\delta$(=C–H) = 793; $\delta_{oop}(NH_2)$ = 665; $\nu(H_2C–Se)$ = 545; $\delta_{ip}(C–NH_2)$ = 452. $^1$H NMR (400 MHz, DMSO-$d_6$), $\delta$(ppm): 7.84 (dd; *J* = 4.2 and 1.9 Hz; 2H; $H_A$); 6.95 (dd; *J* = 7.9 and 4.2 Hz; 2H; $H_B$); 6.92 (dd; *J* = 7.9 and 1.9 Hz; 2H; $H_C$); 5.03 (s; 4H; $H_D$ and $H_E$); 4.80 (s; 2H; $H_F$). $^{13}$C NMR (100 MHz, DMSO-$d_6$), $\delta$(ppm): $C_4$ = 143.5; $C_5$ = 139.7; $C_1$ = 138.1; $C_2$ = 121.2; $C_3$ = 119.5; $C_6$ = 14.6. $^{77}$Se NMR (76 MHz, DMSO-$d_6$), $\delta$(ppm) = 359. UV-Vis [DMF; 10.0 µM]: $\lambda_{max}$ = 264.0 nm; $\varepsilon_{max}$ = 119050 M$^{-1}$·cm$^{-1}$. $E_g$ = 2.36 eV.

### General synthesis procedure for complexes 1–10

In a flask, the ligand **L** (0.100 mmol; 35.8 mg) was dissolved in approximately 2.00 mL of **solvent A**. In another flask, the **metal salt** was dissolved in approximately 2.00 mL of **solvent B**. The denser solution was transferred to a test tube, and the less dense solution was slowly added to it. The tube was left undisturbed, allowing slow diffusion between the phases. After one day, crystals of the products formed, and were subsequently filtered, washed with ethyl ether, and air-dried.

**Table S1.** Synthesis conditions for complexes **1–10**.

| Complex | Solvent A | Metal salt (mmol) | Solvent B |
|---|---|---|---|
| **1** | THF | $CoCl_2·6H_2O$ (0.100) | EtOH |
| **2** | THF | $CoBr_2$ (0.100) | EtOH |
| **3** | EtOH | $ZnCl_2$ (0.100) | THF |
| **4*** | THF | $CuCl_2·2H_2O$ (0.100) | EtOH |
| **4** | $CHCl_3$ | $CuCl_2·2H_2O$ (0.100) | MeOH |
| **5** | $CHCl_3$ | $CuBr_2$ (0.100) | MeOH |
| **6** | THF | CuI (0.200) | MeCN |
| **7** | THF | $CuSO_4·5H_2O$ (0.100) | MeOH |
| **8#** | EtOH | $ZnSO_4·7H_2O$ (0.100) | $H_2O$ |
| **9** | THF | $Ag_2SO_4$ (0.0500) | $H_2O$ |
| **10** | THF | $AgNO_3$ (0.100) | $H_2O$ |

[*] precipitate, not suitable for characterization by SCXRD; [#] only traces of product.

### Complex 1: [CoCl$_2$L]

$C_{11}H_{12}N_4Se_2CoCl_2$: 488.06 g·mol$^{-1}$; teal crystalline solid, stable. Yield: 84.4% (41.2 mg) based on ligand **L**. m.p. = 238 – 240 °C (dec.). EA (%): theoretical = C: 27.07; H: 2.48; N: 11.48; experimental = C: 27.36; H: 2.58; N: 11.34. FT-IR (cm$^{-1}$): $\nu_a(NH_2)$ = 3406; $\nu_s(NH_2)$ = 3314, 3280; $\delta_{ot}(NH_2)$ = 3219, 3204, 3191; $\nu$(=C–H) = 3065; $\nu_a(CH_2)$ = 2997;

$\nu_s(CH_2)$ = 2929; ν(C=N) = 1609; ν(C=C) = 1566, 1455, 1428; $\nu(C–NH_2)$ = 1318; $\delta_{ip}(NH_2)$ = 1043; δ(=C–H) = 798; $\delta_{oop}(NH_2)$ = 685; $\delta_{ip}(C–NH_2)$ = 457. Raman ($cm^{-1}$): ν(C=N) = 1602; ν(C=C) = 1578, 1459, 1431; $\nu(C–NH_2)$ = 1323; $\delta_{ip}(NH_2)$ = 1046; δ(=C–H) = 797; $\delta_{oop}(NH_2)$ = 684; $\nu(H_2C–Se)$ = 541; $\delta_{ip}(C–NH_2)$ = 447; ν(Co–N) = 320; ν(Co–Cl) = 224. HRMS (m/z): 360.9518 (calc. 360.9465), [**L** + H]$^+$; 382.9343 (calc. 382.9285), [**L**+ Na]$^+$; 453.8467 (calc. 453.8413), [**L**CoCl]$^+$; 813.7887 (calc. 813.7805), [**L₂**CoCl]$^+$; 871.7370 (calc. 871.7386), [$\mathbf{L_2}CoCl_2$ + Na]$^+$; 942.6599 (calc. 942.6514), [$\mathbf{L_2}Co_2Cl_3$]$^+$. UV-Vis [DMF; 10.0 µM]: $\lambda_{max}$ = 324.6 nm; $\varepsilon_{max}$ = 32160 $M^{-1}\cdot cm^{-1}$. $E_g$ = 1.81 eV.

**Complex 2: [$CoBr_2$L]**

$C_{11}H_{12}N_4Se_2CoBr_2$: 488.06 g·mol$^{-1}$; teal crystalline solid, stable. Yield: 70.7% (40.8 mg) based on ligand **L**. m.p. = 230 – 232 °C (dec.). EA (%): theoretical = C: 22.90; H: 2.10; N: 9.71; experimental = C: 23.34; H: 2.12; N: 9.68. FT-IR ($cm^{-1}$): $\nu_a(NH_2)$ = 3419, 3402; $\nu_s(NH_2)$ = 3312; $\delta_{ot}(NH_2)$ = 3201, 3183; ν(=C–H) = 3063; $\nu_a(CH_2)$ = 2999; $\nu_s(CH_2)$ = 2927; ν(C=N) = 1608; ν(C=C) = 1566, 1458, 1427; $\nu(C–NH_2)$ = 1318; $\delta_{ip}(NH_2)$ = 1042; δ(=C–H) = 795; $\delta_{oop}(NH_2)$ = 684; $\delta_{ip}(C–NH_2)$ = 454. Raman ($cm^{-1}$): ν(C=N) = 1600; ν(C=C) = 1577, 1457, 1429; $\nu(C–NH_2)$ = 1322; $\delta_{ip}(NH_2)$ = 1046; δ(=C–H) = 797; $\delta_{oop}(NH_2)$ = 683; $\nu(H_2C–Se)$ = 544; $\delta_{ip}(C–NH_2)$ = 448; ν(Co–N) = 320; ν(Co–Br) = 190. HRMS (m/z): 360.9503 (calc. 360.9465), [**L** + H]$^+$; 382.9312 (calc. 382.9285), [**L**+ Na]$^+$; 497.7931 (calc. 497.7908), [**L**CoBr]$^+$; 857.7326 (calc. 857.7300), [**L₂**CoBr]$^+$; 959.6377 (calc. 959.6376), [$\mathbf{L_2}CoBr_2$ + Na]$^+$; 1074.5021 (calc. 1074.4999), [$\mathbf{L_2}Co_2Br_3$]$^+$. UV-Vis [DMF; 10.0 µM]: $\lambda_{max}$ = 324.8 nm; $\varepsilon_{max}$ = 65520 $M^{-1}\cdot cm^{-1}$. $E_g$ = 1.74 eV.

**Complex 3: [$ZnCl_2$L]**

$C_{11}H_{12}N_4Se_2ZnCl_2$: 494.45 g·mol$^{-1}$; yellow crystalline solid, stable. Yield: 85.8% (42.4 mg) based on ligand **L**. m.p. = 224 – 226 °C. EA (%): theoretical = C: 26.72; H: 2.45; N: 11.33; experimental = C: 27.20; H: 2.45; N: 11.19. FT-IR ($cm^{-1}$): $\nu_a(NH_2)$ = 3387; $\nu_s(NH_2)$ = 3300; $\delta_{ot}(NH_2)$ = 3204, 3177; ν(=C–H) = 3083; $\nu_a(CH_2)$ = 3038; $\nu_s(CH_2)$ = 2932; ν(C=N) = 1609; ν(C=C) = 1568, 1454, 1432; $\nu(C–NH_2)$ = 1321; δ(=C–H) = 807; $\delta_{ip}(C–NH_2)$ = 447; ν(Zn–N) = 277. Raman ($cm^{-1}$): ν(C=N) = 1601; ν(C=C) = 1577, 1459, 1431; $\nu(C–NH_2)$ = 1323; $\delta_{ip}(NH_2)$ = 1046; δ(=C–H) = 800; $\delta_{oop}(NH_2)$ = 685; $\nu(H_2C–Se)$ = 542; $\delta_{ip}(C–NH_2)$ = 446; ν(Zn–N) = 320; ν(Zn–Cl) = 218. $^1$H NMR (400 MHz, DMSO-$d_6$), δ(ppm): 7.84 (dd; *J* = 4.3 and 1.8 Hz; 2H; $H_A$); 6.95 (dd; *J* = 7.9 and 4.3 Hz; 2H; $H_B$); 6.91 (dd; *J* = 7.9 and 1.8 Hz; 2H; $H_C$); 5.02 (s; 4H; $H_D$ and $H_E$); 4.77 (s; 2H; $H_F$). $^{13}$C NMR (100 MHz, DMSO-$d_6$), δ(ppm): $C_4$ = 143.6; $C_5$ = 139.7; $C_1$ = 138.2; $C_2$ = 121.4; $C_3$ = 119.7; $C_6$ = 14.7. $^{77}$Se NMR (76 MHz, DMSO-$d_6$), δ(ppm) = 358. HRMS (m/z): 360.9518 (calc. 360.9465), [**L** + H]$^+$; 382.9335 (calc. 382.9285), [**L**+ Na]$^+$; 458.8403 (calc. 458.8372), [**L**ZnCl]$^+$; 782.8132 (calc. 782.7998), [**L₂**Zn - H]$^+$; 818.7809 (calc. 818.7765), [**L₂**ZnCl]$^+$; 916.6781 (calc. 916.6667), [$\mathbf{L_2}Zn_2Cl_2$ - H]$^+$; 952.6471 (calc. 952.6433), [$\mathbf{L_2}Zn_2Cl_3$]$^+$. UV-Vis [DMF; 10.0 µM]: $\lambda_{max}$ = 264.2 nm; $\varepsilon_{max}$ = 87910 $M^{-1}\cdot cm^{-1}$. $E_g$ = 3.23 eV.

**Complex 4: [$CuCl_2$L]**

General synthesis procedure with THF/EtOH: $C_{11}H_{12}N_4Se_2CuCl_2$: 492.61 g·mol$^{-1}$; brown crystalline solid, stable. Yield: 88.0% (43.2 mg) based on ligand **L**. m.p. = 158 – 160 °C. EA (%): theoretical = C: 26.82; H: 2.46; N: 11.37; experimental = C: 27.22; H: 2.47; N: 11.25. FT-IR ($cm^{-1}$): $\nu_a(NH_2)$ = 3393; $\nu_s(NH_2)$ = 3307, 3255; $\delta_{ot}(NH_2)$ = 3218, 3195, 3162; ν(=C–H) = 3066; $\nu_a(CH_2)$ = 3038; $\nu_s(CH_2)$ = 2984; ν(C=N) = 1623; ν(C=C) = 1561, 1469, 1454; $\nu(C–NH_2)$ = 1316; δ(=C–H) = 790; $\delta_{ip}(C–NH_2)$ = 458. Raman ($cm^{-1}$): $\delta_{ip}(NH_2)$ = 1044; $\delta_{oop}(NH_2)$ = 683; $\nu(H_2C–Se)$ = 543;

ν(Cu–N) = 248. HRMS (m/z): 360.9514 (calc. 360.9465), [**L** + H]$^+$; 382.9312 (calc. 382.9285), [**L**+ Na]$^+$; 457.8427 (calc. 457.8377), [**L**CuCl]$^+$; 914.6763 (calc. 914.6676), [**L₂**$Cu_2Cl_2$ - H]$^+$; 950.6518 (calc. 950.6442), [**L₂**$Cu_2Cl_3$]$^+$. UV-Vis [DMF; 10.0 µM]: $\lambda_{max}$ = 324.4 nm; $\varepsilon_{max}$ = 75060 $M^{-1}\cdot cm^{-1}$. $E_g$ = 2.86 eV.
General synthesis procedure with $CHCl_3$/MeOH: $C_{11}H_{12}N_4Se_2CuCl_2\cdot CHCl_3$ (structure measured by SCXRD): 611.97 g·mol$^{-1}$; partially stable brown crystals that lose the $CHCl_3$ molecule when exposed to air. Yield: 66.7% (20.4 mg) based on $CuCl_2\cdot 2H_2O$. m.p. = 158 – 160 °C. EA (%): theoretical (without $CHCl_3$) = C: 26.82; H: 2.46; N: 11.37; experimental = C: 26.95; H: 2.33; N: 11.10.

**Complex 5: [$CuBr_2$L]·0,5$CHCl_3$**

$C_{11}H_{12}N_4Se_2CuBr_2\cdot 0{,}5CHCl_3$: 641.20 g·mol$^{-1}$; brown crystalline solid, stable. Yield: 86.4% (55.4 mg) based on ligand **L**. m.p. = 130 – 132 °C. EA (%): theoretical = C: 21.54; H: 1.96; N: 8.74; experimental = C: 21.54; H: 1.98; N: 8.73. FT-IR (cm$^{-1}$): $\nu_a(NH_2)$ = 3398, 3372; $\nu_s(NH_2)$ = 3305, 3287; $\delta_{ot}(NH_2)$ = 3210, 3183; ν(=C–H) = 3071; $\nu_a(CH_2)$ = 2997; $\nu_s(CH_2)$ = 2942; ν(C=N) = 1613, 1596; ν(C=C) = 1579, 1463, 1425; ν(C–$NH_2$) = 1329; δ(=C–H) = 797; $\delta_{oop}(NH_2)$ = 685; $\delta_{ip}$(C–$NH_2$) = 445. Raman (cm$^{-1}$): $\delta_{ip}(NH_2)$ = 1043; $\delta_{oop}(NH_2)$ = 686; ν($H_2$C–Se) = 539; ν(Cu–N) = 244. HRMS (m/z): 360.9521 (calc. 360.9465), [**L** + H]$^+$; 382.9354 (calc. 382.9285), [**L**+ Na]$^+$; 501.7916 (calc. 501.7872), [**L**CuBr]$^+$; 924.6605 (calc. 924.6560). UV-Vis [DMF; 10.0 µM]: $\lambda_{max}$ = 264.2 nm; $\varepsilon_{max}$ = 73430 $M^{-1}\cdot cm^{-1}$. $E_g$ = 2.22 eV.

**Complex 6: [$Cu_2(\mu$-I$)_2$L]$_n$**

$C_{11}H_{12}N_4Se_2CuI_2$: 739.06 g·mol$^{-1}$; yellowish-brown crystalline solid, stable. Yield: 85.3% (63.0 mg) based on ligand **L**. m.p. = 178 – 180 °C. EA (%): theoretical = C: 17.88; H: 1.64; N: 7.58; experimental = C: 18.54; H: 1.69; N: 7.88. FT-IR (cm$^{-1}$): $\nu_a(NH_2)$ = 3451, 3429; $\nu_s(NH_2)$ = 3295, 3284; $\delta_{ot}(NH_2)$ = 3186, 3159; ν(=C–H) = 3060; $\nu_a(CH_2)$ = 3011; $\nu_s(CH_2)$ = 2936; ν(C=N) = 1602; ν(C=C) = 1559, 1449, 1421; ν(C–$NH_2$) = 1317; δ(=C–H) = 799; $\delta_{oop}(NH_2)$ = 672; $\delta_{ip}$(C–$NH_2$) = 449. Raman (cm$^{-1}$): ν(C=C) = 1451, 1426; ν(C–$NH_2$) = 1319; $\delta_{ip}(NH_2)$ = 1041; $\delta_{oop}(NH_2)$ = 674; ν($H_2$C–Se) = 540; $\delta_{ip}$(C–$NH_2$) = 451; ν(Cu–N) = 313; ν(Cu–I) = 155. UV-Vis [DMSO]: $\lambda_{max}$ = 257.8 nm. $E_g$ = 2.77 eV.

**Complex 7: [$Cu_2(\mu$-$SO_4)_2$L$_2$]**

$C_{22}H_{24}N_8Se_4Cu_2S_2O_8$: 1035.54 g·mol$^{-1}$; brown crystalline solid, stable. Yield: 75.9% (39.3 mg) based on ligand **L**. m.p. = 175 – 177 °C (dec.). EA (%): theoretical = C: 25.52; H: 2.34; N: 10.82; experimental = C: 25.66; H: 2.38; N: 10.72. FT-IR (cm$^{-1}$): $\nu_a(NH_2)$ = 3400, 3368; $\nu_s(NH_2)$ = 3313; $\delta_{ot}(NH_2)$ = 3205; ν(=C–H) = 3103, 3087; $\nu_a(CH_2)$ = 3067; $\nu_s(CH_2)$ = 2999; ν(C=N) = 1640, 1620; ν(C=C) = 1594, 1477, 1462, 1439, 1427; ν($SO_4$) = 1179 - 964; δ(=C–H) = 813; δ($SO_4$) = 685 - 571; $\delta_{ip}$(C–$NH_2$) = 465. Raman (cm$^{-1}$): ν(C=C) = 1463, 1428; ν(C–$NH_2$) = 1332; $\delta_{oop}(NH_2)$ = 686; ν($H_2$C–Se) = 540; ν(Cu–N) = 245. UV-Vis [DMSO, 10.0 µM]: $\lambda_{max}$ = 258.0 nm; $\varepsilon_{max}$ = 125990 $M^{-1}\cdot cm^{-1}$. $E_g$ = 1.74 eV.

**Complex 8: [$Zn_2(\mu$-$SO_4)_2$L$_2$]**

In a flask equipped with a magnetic stirring system, ligand **L** (0.100 mmol; 35.8 mg), $ZnSO_4\cdot 7H_2O$ (0.100 mmol; 28.8 mg), approximately 3.00 mL of MeOH, and 1.00 mL of deionized water were added. As the reaction proceeded,

the product precipitated as a yellowish powder. After 24 hours, the precipitate was filtered, washed with ethyl ether, and air-dried. Traces of the product in the form of yellow single crystals can be obtained following the general synthesis procedure with EtOH/$H_2O$.

$C_{22}H_{24}N_8Se_4Zn_2S_2O_8$: 1039.20 g·mol$^{-1}$; yellowish crystalline solid, stable. Yield: 74.1% (38.5 mg) based on ligand **L**. m.p. = 230 – 232 °C (dec.). EA (%): theoretical = C: 25.43; H: 2.33; N: 10.78; experimental = C: 25.89; H: 2.50; N: 10.80. FT-IR (cm$^{-1}$): $\nu_a(NH_2)$ = 3407, 3384; $\nu_s(NH_2)$ = 3323, 3298; $\delta_{ot}(NH_2)$ = 3223, 3205; ν(=C–H) = 3088; $\nu_a(CH_2)$ = 3072; $\nu_s(CH_2)$ = 2994; ν(C=N) = 1620; ν(C=C) = 1589, 1466, 1430; $\nu(SO_4)$ = 1210 - 974; δ(=C–H) = 809; $\delta(SO_4)$ = 647 - 567; $\delta_{ip}(C–NH_2)$ = 468. Raman (cm$^{-1}$): ν(C=N) = 1620; ν(C=C) = 1579, 1465, 1431; $\nu(C–NH_2)$ = 1328; $\delta_{ip}(NH_2)$ = 1051; δ(=C–H) = 809; $\delta_{oop}(NH_2)$ = 686; $\nu(H_2C–Se)$ = 543; ν(Zn–N) = 324, 337. UV-Vis [DMSO]: $\lambda_{max}$ = 258.2 and 327.2 nm. $E_g$ = 3.25 eV.

**Complex 9: $[Ag_2L_2]SO_4·7H_2O$**

$C_{22}H_{24}N_8Se_4Ag_2SO_4·7H_2O$ (determined by SCXRD): 1154.23 g·mol$^{-1}$; colorless crystalline solid, sensitive to light. It undergoes partial dehydration, losing three water molecules (according to EA results).

$C_{22}H_{24}N_8Se_4Ag_2SO_4·4H_2O$ (confirmed by EA): 1100.18 g·mol$^{-1}$. Yield: 87.6% (48.2 mg) based on ligand **L**. m.p. = 120 – 122 °C (dec.). EA (%): theoretical = C: 24.02; H: 2.93; N: 10.19; experimental = C: 24.14; H: 2.88; N: 10.04. FT-IR (cm$^{-1}$): $\nu(H_2O)$ = 3600 - 2800; $\nu_a(NH_2)$ = 3418; $\nu_s(NH_2)$ = 3329; $\delta_{ot}(NH_2)$ = 3215, 3193; ν(=C–H) = 3023; $\nu_a(CH_2)$ = 2947; ν(C=N) = 1628; ν(C=C) = 1576, 1463, 1413; $\nu(SO_4)$ = 1050; δ(=C–H) = 793; $\delta(SO_4)$ = 609; $\delta_{ip}(C–NH_2)$ = 458. Raman (cm$^{-1}$): ν(C=C) = 1423; $\delta_{ip}(NH_2)$ = 1036; ν $(SO_4)$ = 966; δ(=C–H) = 802; $\delta_{oop}(NH_2)$ = 663; $\delta(SO_4)$ = 632; $\nu(H_2C–Se)$ = 538; $\delta_{ip}(C–NH_2)$ = 458; ν(Ag–N) = 301; ν(Ag–Se) = 182. HRMS (m/z): 360.9520 (calc. 360.9465), [**L** + H]$^+$; 382.9324 (calc. 382.9285), [**L**+ Na]$^+$; 466.8484 (calc. 466.8443), [**L**Ag]$^+$; 742.8757 (calc. 742.8677), [$\mathbf{L_2}$ + Na]$^+$; 758.8480 (calc. 758.8416), [$\mathbf{L_2}$ + K]$^+$; 826.7912 (calc. 826.7836), [$\mathbf{L_2}$Ag]$^+$. UV-Vis [DMSO]: $\lambda_{max}$ = 258.0 nm. $E_g$ = 3.26 eV.

**Complex 10: $[Ag_2L_2](NO_3)_2·2H_2O$**

$C_{22}H_{24}N_{10}Se_4Ag_2O_6·2H_2O$: 1092.10 g·mol$^{-1}$; colorless crystalline solid, sensitive to light. Yield: 72.2% (39.4 mg) based on ligand **L**. m.p. = 140 – 142 °C (dec.). EA: theoretical = C: 24.28; H: 2.22; N: 12.87; experimental = C: 24.44; H: 2.51; N: 12.77. FT-IR (cm$^{-1}$): $\nu(H_2O)$ = 3569; $\nu_a(NH_2)$ = 3425; $\nu_s(NH_2)$ = 3328; $\delta_{ot}(NH_2)$ = 3220, 3199; ν(=C–H) = 3044; $\nu_a(CH_2)$ = 3021; $\nu_s(CH_2)$ = 2949; ν(C=N) = 1627; ν(C=C) = 1576; $\nu(NO_3)$ = 1327; $\delta(NO_3)$ = 960; δ(=C–H) = 795 ; $\delta_{oop}(NH_2)$ = 659; $\delta_{ip}(C–NH_2)$ = 457. Raman (cm$^{-1}$): ν(C=C) = 1354; $\delta_{ip}(NH_2)$ = 1044; $\delta_{oop}(NH_2)$ = 666; $\nu(H_2C–Se)$ = 540; $\delta_{ip}(C–NH_2)$ = 455; ν(Ag–N) = 311; ν(Ag–Se) = 180. HRMS (m/z): 360.9506 (calc. 360.9465), [**L** + H]$^+$; 466.8500 (calc. 466.8443), [**L**Ag]$^+$; 826.7952 (calc. 826.7836), [$\mathbf{L_2}$Ag]$^+$. UV-Vis [DMSO, 10.0 µM]: $\lambda_{max}$ = 257.6 nm; $\varepsilon_{max}$ = 127030 M$^{-1}$·cm$^{-1}$. $E_g$ = 3.33 eV.

**Mesoporous titanium dioxide (m-$TiO_2$)**

The synthesis methodology was based on the literature:[13] initially, Pluronic® F-127 (0.100 mmol; 1.230 g) was dissolved in a mixture of $H_2O$/EtOH (9/23 mL) under stirring for 1 h, with slight heating if necessary. Then, concentrated HCl was slowly added to acidify the medium to pH ~3. Subsequently, titanium(IV) isopropoxide ($Ti(iPrO)_4$, 20.0 mmol; 6.14 mL) was added dropwise, leading to the immediate formation of a white suspension.

The mixture was stirred for 24 h at 50 °C, after which a gel was observed. The gel was thoroughly washed with water to remove Pluronic® F-127 residues and other impurities. Finally, the synthesized $TiO_2$ underwent thermal treatment at 500 °C for 6 h.

m-$TiO_2$ (textural properties): surface area (BET) = 42.4 $m^2/g$; average pore size (BET) = 18.9 nm; average pore volume (BJH) = 0.200 $cm^3/g$; average particle size = 106.9 nm. The crystalline phase observed by PXRD: anatase. Average crystallite size: 23.3 nm. Estimated energy gap ($E_g$): 3.41 eV. FT-IR ($cm^{-1}$): $\nu(H_2O)$ = 3600 - 2800; $\delta(H_2O)$ = 1641; $\nu$(Ti–O) = 630, 430, 251. Raman ($cm^{-1}$), anatase phase standard: 638, 515, 396, 196, 143.

## SINGLE CRYSTAL X-RAY DIFFRACTION (SCXRD)

**Table S2.** Diffraction intensity collection and refinement data for ligand **L** and complexes **1** and **2**.

| | **L** | **1** | **2** |
|---|---|---|---|
| CCDC | 2389831 | 2389832 | 2389833 |
| Empirical formula | $C_{11}H_{12}N_4Se_2$ | $C_{11}H_{12}Cl_2CoN_4Se_2$ | $C_{11}H_{12}Br_2CoN_4Se_2$ |
| Molar mass (g·mol$^{-1}$) | 358.17 | 488.00 | 576.92 |
| Temperature (K) | 100(2) | 296(2) | 300(2) |
| Radiation; λ (Å) | Mo Kα; 0.71073 | Cu Kα; 1.54178 | Mo Kα; 0.71073 |
| Crystal system; space group | orthorhombic, *Pbcn* | triclinic, $P\bar{1}$ | triclinic, $P\bar{1}$ |
| **a** (Å) | 7.587(2) | 7.7790(11) | 7.854(2) |
| **b** (Å) | 11.850(2) | 14.678(2) | 15.068(5) |
| **c** (Å) | 13.974(3) | 15.899(3) | 16.136(6) |
| α (°) | 90 | 115.746(8) | 117.242(7) |
| β (°) | 90 | 103.518(6) | 103.026(6) |
| γ (°) | 90 | 90.961(8) | 91.247(7) |
| Volume (Å$^3$) | 1256.2(5) | 1575.3(4) | 1636.9(9) |
| Z; calculated density (g·cm$^{-3}$) | 4; 1.894 | 4; 2.058 | 4; 2.341 |
| Linear abs. coefficient (mm$^{-1}$) | 5.869 | 16.903 | 10.371 |
| F(000) | 696 | 940 | 1084 |
| Crystal size (mm) | 0.165 x 0.154 x 0.107 | 0.370 x 0.242 x 0.106 | 0.221 x 0.085 x 0.041 |
| Angular scan region θ (°) | 2.915 to 30.522 | 3.203 to 72.238 | 2.584 to 30.565 |
| Index scan region | -8 ≤ h ≤ 10 | -9 ≤ h ≤ 9 | -11 ≤ h ≤ 8 |
| | -13 ≤ k ≤ 16 | -17 ≤ k ≤ 18 | -21 ≤ k ≤ 21 |
| | -19 ≤ l ≤ 18 | -19 ≤ l ≤ 19 | -23 ≤ l ≤ 23 |
| Num. of collected reflections | 9706 | 80219 | 35800 |
| Num. of independent reflections [R(int)] | 1921 [0.0283] | 6202 [0.0464] | 10006 [0.0377] |
| Measurement completeness (%) | 99.8 | 99.7 | 99.6 |
| Absorption correction | multi-scan | multi-scan | multi-scan |
| Min. and max. transmission | 0.6280 and 0.7461 | 0.2934 and 0.7536 | 0.5281and 0.7461 |
| Data/restrictions/parameters | 1921 / 2 / 84 | 6202 / 8 / 385 | 10006 / 8 / 385 |
| Goodness-of-fit on $F^2$ | 1.067 | 1.081 | 1.013 |
| Final R index [I > 2σ(I)] | $R_1$ = 0.0254 | $R_1$ = 0.0350 | $R_1$ = 0.0323 |
| | $wR_2$ = 0.0514 | $wR_2$ = 0.0952 | $wR_2$ = 0.0607 |
| R index (all data)* | $R_1$ = 0.0368 | $R_1$ = 0.0355 | $R_1$ = 0.0666 |
| | $wR_2$ = 0.0549 | $wR_2$ = 0.0957 | $wR_2$ = 0.0702 |
| Residual electron density (e·Å$^{-3}$) | -0.612 and 0.421 | -1.025 and 0.552 | -0.757 and 0.441 |

*R = $|F_0 - F_c|$ I $|F_0|$; $wR_2 = [w(F_0^2 – F_c^2)^2 / (wF_0^2)]^{-1/2}$.

**Table S3.** Diffraction intensity collection and refinement data for complexes **3**, **4**, and **5**.

| | **3** | **4** | **5** |
|---|---|---|---|
| CCDC | 2389834 | 2389835 | 2389836 |
| Empirical formula | $C_{11}H_{12}Cl_2ZnN_4Se_2$ | $C_{12}H_{13}Cl_5CuN_4Se_2$ | $C_{23}H_{25}Br_4Cl_3Cu_2N_8Se_4$ |
| Molar mass ($g \cdot mol^{-1}$) | 494.44 | 611.97 | 1282.42 |
| Temperature (K) | 296(2) | 301(2) | 302(2) |
| Radiation; λ (Å) | Cu Kα; 1.54178 | Mo Kα; 0.71073 | Mo Kα; 0.71073 |
| Crystal system; space group | triclinic, $P\bar{1}$ | monoclinic, $P2_1/n$ | triclinic, $P\bar{1}$ |
| **a** (Å) | 7.8387(10) | 8.752(3) | 8.875(3) |
| **b** (Å) | 14.6623(18) | 13.984(5) | 9.821(4) |
| **c** (Å) | 15.820(2) | 16.175(5) | 22.604(8) |
| α (°) | 115.472(9) | 90 | 83.433(10) |
| β (°) | 103.747(7) | 95.122(7) | 82.479(10) |
| γ (°) | 91.342(9) | 90 | 72.019(14) |
| Volume (Å$^3$) | 1578.0(4) | 1971.8(11) | 1852.2(11) |
| Z; calculated density ($g \cdot cm^{-3}$) | 4; 2.081 | 4; 2.062 | 2; 2.299 |
| Linear abs. coefficient ($mm^{-1}$) | 10.509 | 5.480 | 9.640 |
| F(000) | 952 | 1180 | 1208 |
| Crystal size (mm) | 0.297 x 0.230 x 0.120 | 0.214 x 0.165 x 0.064 | 0.146 x 0.075 x 0.047 |
| Angular scan region θ (°) | 3.218 to 72.171 | 1.928 to 30.568 | 2.187 to 30.570 |
| Index scan region | $-9 \leq h \leq 9$ | $-10 \leq h \leq 12$ | $-12 \leq h \leq 12$ |
| | $-17 \leq k \leq 18$ | $-19 \leq k \leq 20$ | $-14 \leq k \leq 14$ |
| | $-19 \leq l \leq 19$ | $-23 \leq l \leq 23$ | $-32 \leq l \leq 32$ |
| Num. of collected reflections | 35302 | 25964 | 61513 |
| Num. of independent reflections [R(int)] | 6204 [0.0314] | 6047 [0.0603] | 11313 [0.0597] |
| Measurement completeness (%) | 99.7 | 99.8 | 99.5 |
| Absorption correction | multi-scan | multi-scan | multi-scan |
| Min. and max. transmission | 0.4180 and 0.7536 | 0.6002 and 0.7461 | 0.6315 and 0.7461 |
| Data/restrictions/parameters | 6204 / 8 / 385 | 6047 / 4 / 229 | 11313 / 8 / 421 |
| Goodness-of-fit on $F^2$ | 1.097 | 1.015 | 1.025 |
| Final R index [$I > 2\sigma(I)$] | $R_1 = 0.0253$ | $R_1 = 0.0451$ | $R_1 = 0.0564$ |
| | $wR_2 = 0.0649$ | $wR_2 = 0.0823$ | $wR_2 = 0.1235$ |
| R index (all data)* | $R_1 = 0.0264$ | $R_1 = 0.1085$ | $R_1 = 0.1292$ |
| | $wR_2 = 0.0659$ | $wR_2 = 0.1003$ | $wR_2 = 0.1497$ |
| Residual electron density ($e \cdot Å^{-3}$) | -0.638 and 0.369 | -0.894 and 0.658 | -0.952 and 1.468 |

*$R = |F_0 - F_c| \text{ I } |F_0|$; $wR_2 = [w(F_0^2 – F_c^2)^2 / (wF_0^2)]^{-1/2}$.

**Table S4.** Diffraction intensity collection and refinement data for complexes **6**, **7**, and **8**.

| | **6** | **7** | **8** |
|---|---|---|---|
| CCDC | 2389837 | 2389838 | 2389839 |
| Empirical formula | $C_{11}H_{12}Cu_2I_2N_4Se_2$ | $C_{22}H_{24}Cu_2N_8O_8S_2Se_4$ | $C_{22}H_{24}Zn_2N_8O_8S_2Se_4$ |
| Molar mass (g·mol$^{-1}$) | 739.05 | 1035.53 | 1039.19 |
| Temperature (K) | 302(2) | 302(2) | 100(2) |
| Radiation; λ (Å) | Mo Kα; 0.71073 | Mo Kα; 0.71073 | Mo Kα; 0.71073 |
| Crystal system; space group | orthorhombic, *Pbca* | triclinic, $P\bar{1}$ | triclinic, $P\bar{1}$ |
| **a** (Å) | 8.077(2) | 7.857(3) | 7.7300(16) |
| **b** (Å) | 13.486(3) | 9.733(3) | 10.109(3) |
| **c** (Å) | 31.387(10) | 11.054(4) | 10.979(3) |
| α (°) | 90 | 86.403(11) | 83.081(9) |
| β (°) | 90 | 71.954(14) | 71.424(10) |
| γ (°) | 90 | 72.867(9) | 71.776(10) |
| Volume (Å$^3$) | 3418.8(15) | 767.6(5) | 772.2(3) |
| Z; calculated density (g·cm$^{-3}$) | 8; 2.872 | 1; 2.240 | 1; 2.235 |
| Linear abs. coefficient (mm$^{-1}$) | 10.355 | 6.324 | 6.463 |
| F(000) | 2704 | 502 | 504 |
| Crystal size (mm) | 0.238 x 0.110 x 0.089 | 0.182 x 0.149 x 0.145 | 0.134 x 0.128 x 0.069 |
| Angular scan region θ (°) | 2.836 to 30.581 | 1.939 to 30.568 | 1.957 to 30.579 |
| Index scan region | $-11 \leq h \leq 11$ | $-11 \leq h \leq 11$ | $-11 \leq h \leq 11$ |
| | $-14 \leq k \leq 29$ | $-13 \leq k \leq 13$ | $-14 \leq k \leq 14$ |
| | $-44 \leq l \leq 44$ | $-15 \leq l \leq 14$ | $-15 \leq l \leq 15$ |
| Num. of collected reflections | 70865 | 23627 | 35446 |
| Num. of independent reflections [R(int)] | 5242 [0.0267] | 4704 [0.0302] | 4729 [0.0297] |
| Measurement completeness (%) | 99.8 | 99.9 | 99.8 |
| Absorption correction | multi-scan | multi-scan | multi-scan |
| Min. and max. transmission | 0.4931 and 0.7461 | 0.5643 and 0.7461 | 0.6001 and 0.7461 |
| Data/restrictions/parameters | 5242 / 4 / 202 | 4704 / 4 / 214 | 4729 / 4 / 220 |
| Goodness-of-fit on $F^2$ | 1.068 | 1.045 | 1.085 |
| Final R index [I > 2σ(I)] | $R_1 = 0.0250$ | $R_1 = 0.0403$ | $R_1 = 0.0247$ |
| | $wR_2 = 0.0588$ | $wR_2 = 0.1006$ | $wR_2 = 0.0894$ |
| R index (all data)* | $R_1 = 0.0317$ | $R_1 = 0.0521$ | $R_1 = 0.0281$ |
| | $wR_2 = 0.0621$ | $wR_2 = 0.1083$ | $wR_2 = 0.0865$ |
| Residual electron density (e·Å$^{-3}$) | -0.984 and 1.188 | -0.781 and 1.120 | -1.173 and 1.077 |

*$R = |F_0 - F_c| \text{ I } |F_0|$; $wR_2 = [w(F_0^2 – F_c^2)^2 / (wF_0^2)]^{-1/2}$.

**Table S5.** Diffraction intensity collection and refinement data for complexes **9** and **10**.

| | **9** | **10** |
|---|---|---|
| CCDC | 2389840 | 2389841 |
| Empirical formula | $C_{22}H_{38}Ag_2N_8O_{11}SSe_4$ | $C_{22}H_{28}Ag_2N_{10}O_8Se_4$ |
| Molar mass (g·mol$^{-1}$) | 1154.24 | 1092.12 |
| Temperature (K) | 100(2) | 100(2) |
| Radiation; $\lambda$ (Å) | Mo K$\alpha$; 0.71073 | Mo K$\alpha$; 0.71073 |
| Crystal system; space group | triclinic, $P\bar{1}$ | triclinic, $P\bar{1}$ |
| **a** (Å) | 11.3001(14) | 6.8376(15) |
| **b** (Å) | 13.740(3) | 10.814(2) |
| **c** (Å) | 13.897(3) | 11.1034(12) |
| $\alpha$ (°) | 115.164(4) | 79.090(10) |
| $\beta$ (°) | 96.479(10) | 73.989(15) |
| $\gamma$ (°) | 112.242(10) | 77.274(10) |
| Volume (Å$^3$) | 1708.4(5) | 762.5(3) |
| Z; calculated density (g·cm$^{-3}$) | 2; 2.244 | 1; 2.378 |
| Linear abs. coefficient (mm$^{-1}$) | 5.535 | 6.123 |
| F(000) | 1120 | 524 |
| Crystal size (mm) | 0.164 x 0.069 x 0.060 | 0.280 x 0.101 x 0.047 |
| Angular scan region $\theta$ (°) | 2.017 to 30.574 | 2.546 to 30.568 |
| Index scan region | $-16 \leq h \leq 16$ | $-8 \leq h \leq 9$ |
| | $-19 \leq k \leq 19$ | $-15 \leq k \leq 15$ |
| | $-19 \leq l \leq 19$ | $-14 \leq l \leq 15$ |
| Num. of collected reflections | 69640 | 19588 |
| Num. of independent reflections [R(int)] | 10448 [0.0269] | 4665 [0.0229] |
| Measurement completeness (%) | 99.8 | 99.8 |
| Absorption correction | multi-scan | multi-scan |
| Min. and max. transmission | 0.6072 and 0.7461 | 0.5581 and 0.7461 |
| Data/restrictions/parameters | 10448 / 25 / 487 | 4665 / 7 / 226 |
| Goodness-of-fit on $F^2$ | 1.070 | 1.126 |
| Final R index [$I > 2\sigma(I)$] | $R_1 = 0.0197$ | $R_1 = 0.0195$ |
| | $wR_2 = 0.0463$ | $wR_2 = 0.0417$ |
| R index (all data)* | $R_1 = 0.0236$ | $R_1 = 0.0243$ |
| | $wR_2 = 0.0478$ | $wR_2 = 0.0435$ |
| Residual electron density (e·Å$^{-3}$) | -1.139 and 2.040 | -0.807 and 0.555 |

*$R = |F_0 - F_c| \text{ I } |F_0|$; $wR_2 = [w(F_0^2 – F_c^2)^2 / (wF_0^2)]^{-1/2}$.

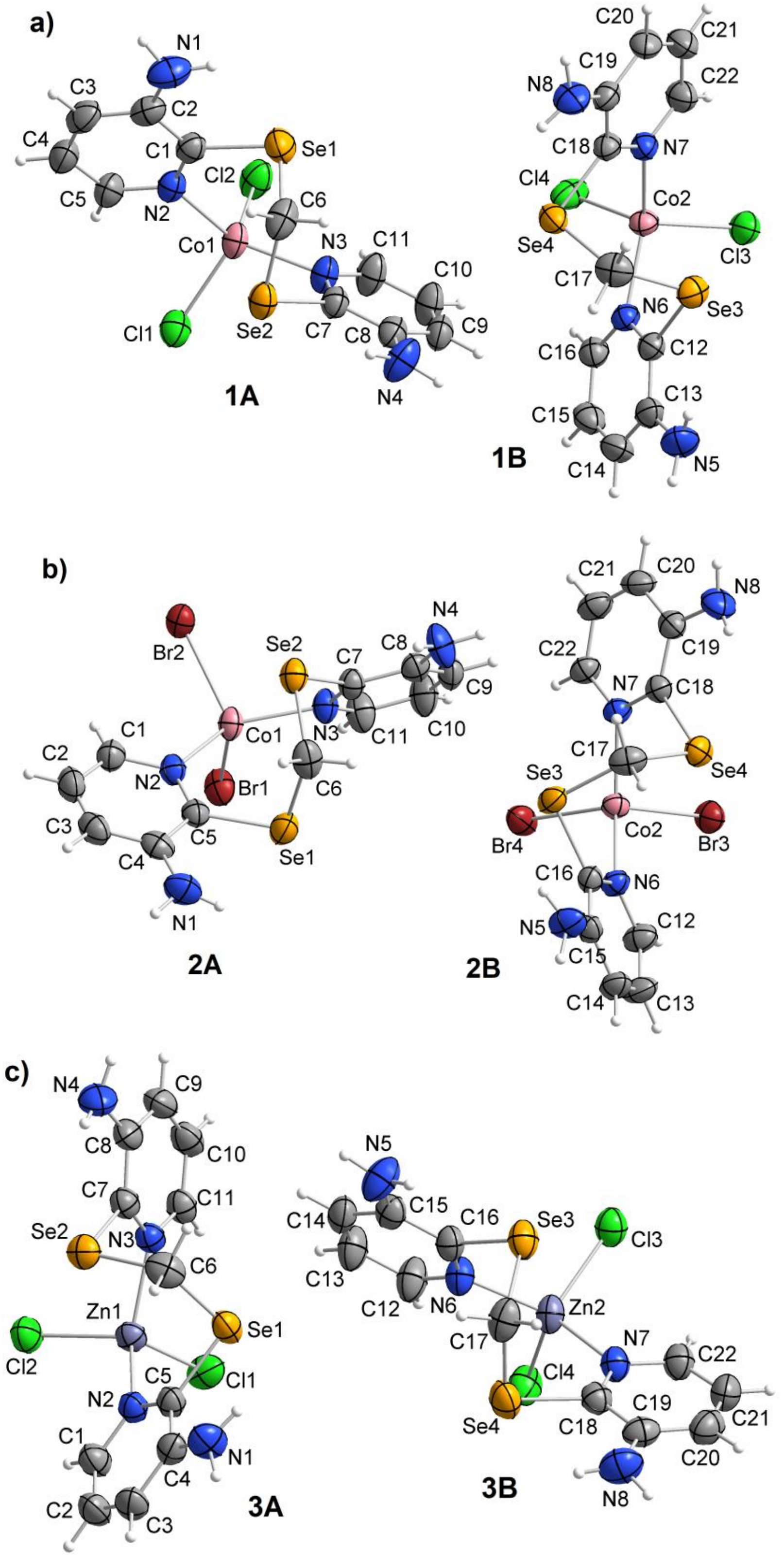


**Figure S2.** Structural projection of the two independent units of the complexes: (a) **1** (**1A** and **1B**), (b) **2** (**2A** and **2B**), and (c) **3** (**3A** and **3B**). Anisotropic displacement parameters are shown with 50% occupancy probability.

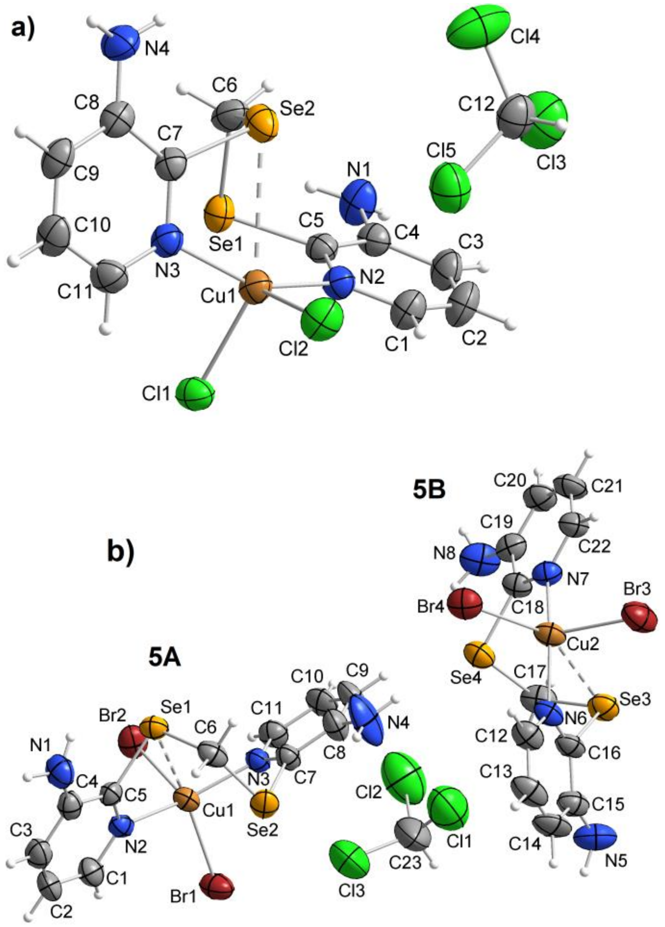


**Figure S3.** Structural projection of the complexes: (a) **4** and (b) the two independent units of **5** (**5A** and **5B**). Anisotropic displacement parameters are shown with 50% occupancy probability.

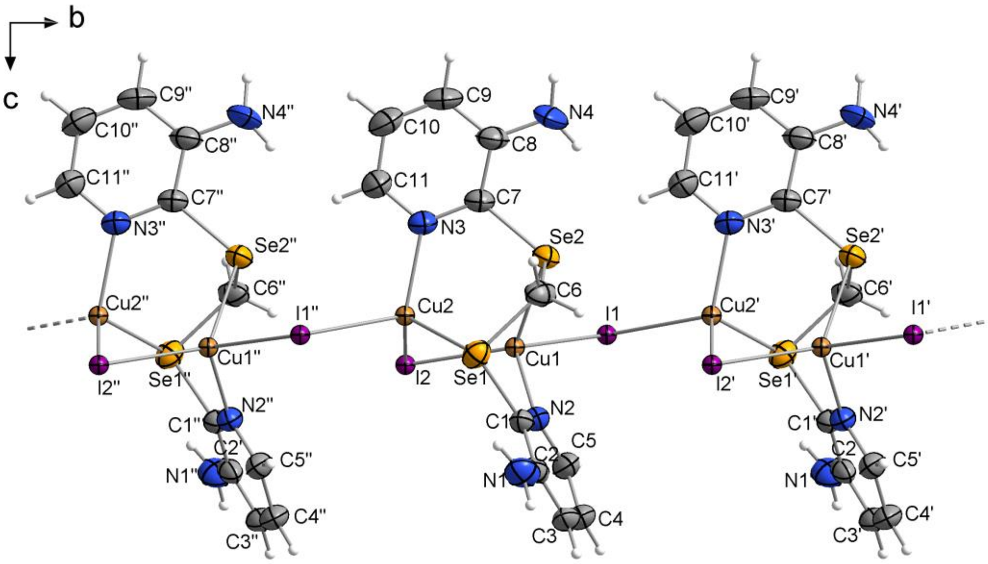


**Figure S4.** Structural projection of polymer **6** along the crystallographic direction [100]. Anisotropic displacement parameters are shown with 50% occupancy probability. Symmetry operations: ' = (3/2 - x, ½ + y, z); " = (3/2 - x, -1/2 + y, z).

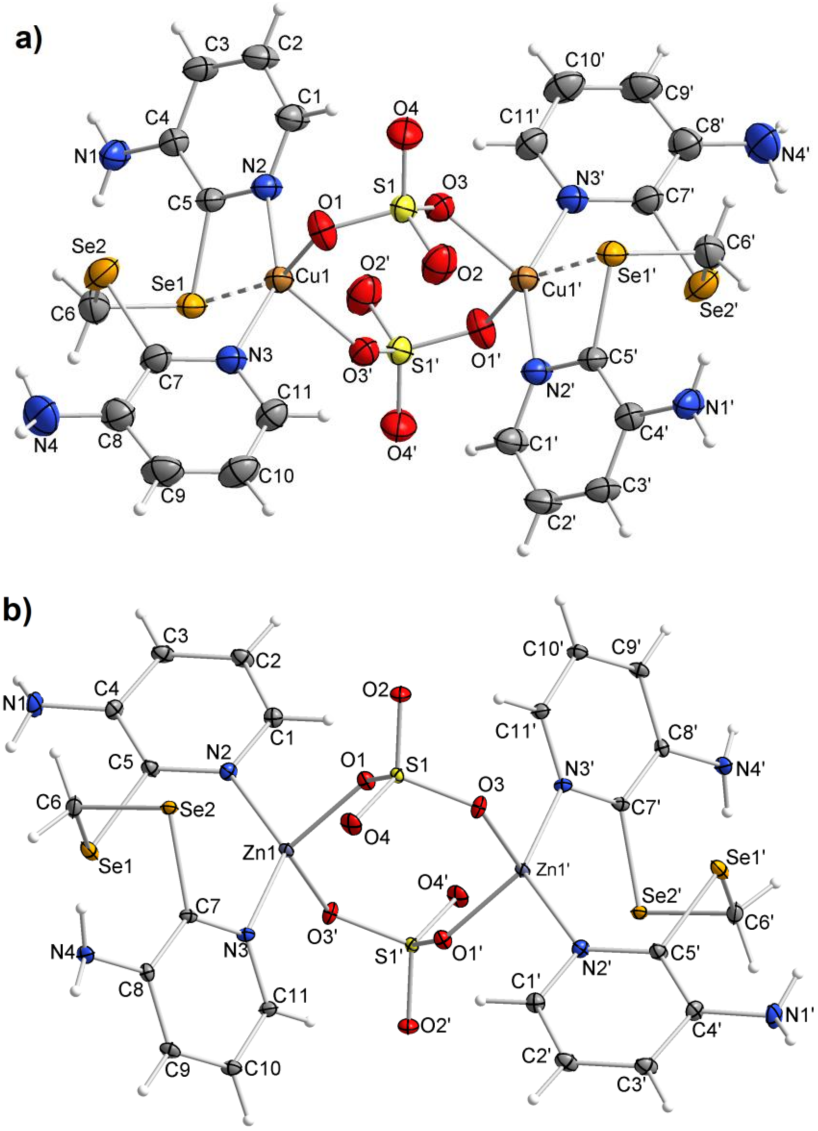


**Figure S5.** Structural projection of the complexes: (a) **7** and (b) **8**. Anisotropic displacement parameters are shown with 50% occupancy probability. Symmetry operations: (**7**) ‘ = (1 - x, 1 - y, 2 - z); (**8**) ‘ = (1 - x, 2 - y, 1 - z).

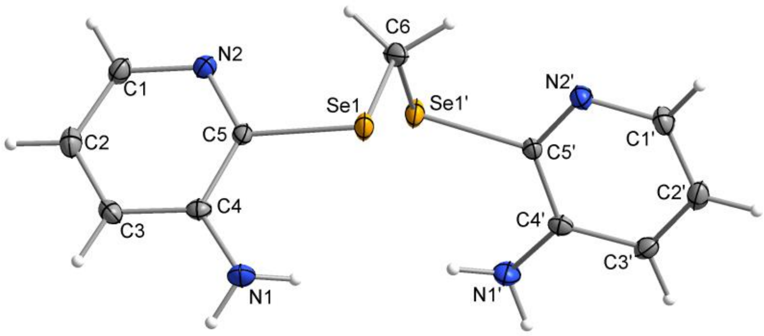


**Figure S6.** Structural projection of ligand **L**. Anisotropic displacement parameters are shown with 50% occupancy probability.

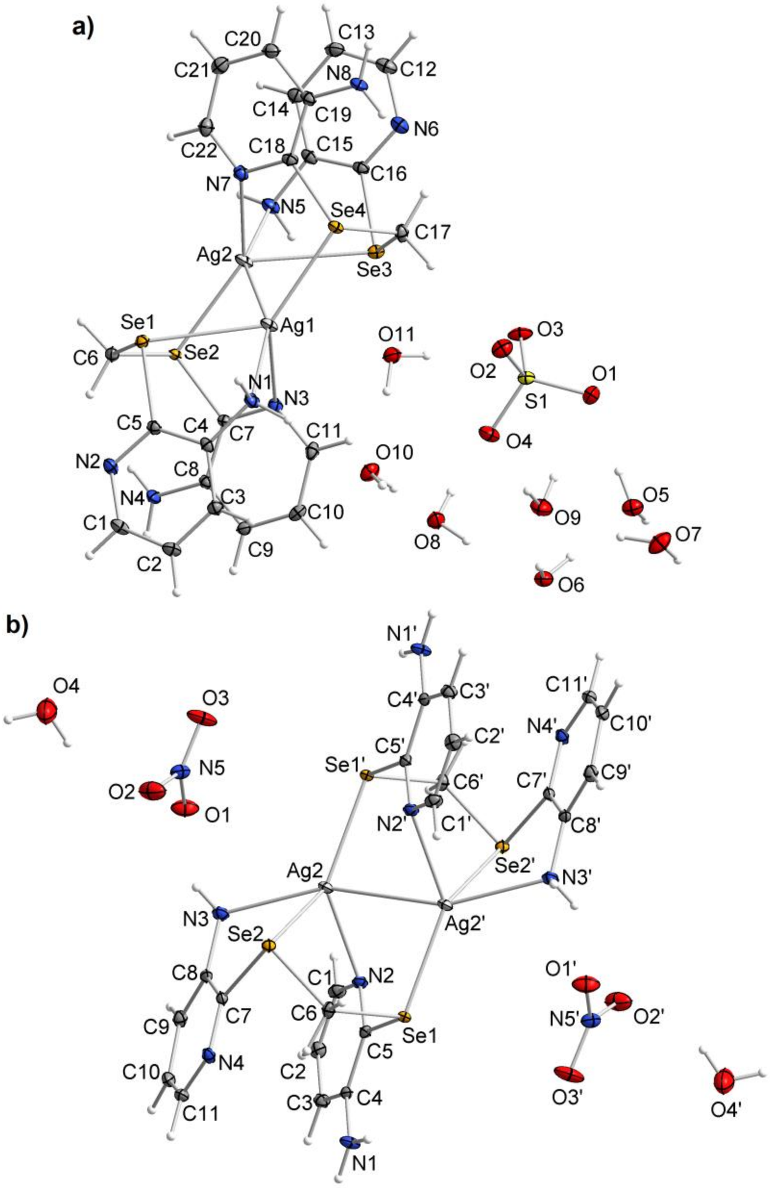


**Figure S7.** Structural projection of the complexes: (a) **9** and (b) **10**. Anisotropic displacement parameters are shown with 50% occupancy probability. Symmetry operation: ‘ = (-x, 1 - y, 1 - z).

**Table S6.** EA results for complexes **9** and **10**.

| Complex | EA theoretical | EA experimental |
|---|---|---|
| **9 (7$H_2O$)**[SCXRD] | C: 22.89; H: 3.32; N: 9.71 | C: 24.14; H: 2.88; N: 10.04 |
| **9 (4$H_2O$)**[EA] | C: 24.02; H: 2.93; N: 10.19 | |
| **10** | C: 24.28; H: 2.22; N: 12.87 | C: 24.44; H: 2.51; N: 12.77 |

[SCXRD] structure measured by SCXRD; [EA] structure confirmed by EA.

**Table S7.** Selected bond lengths and angles for complexes **1–4**. Standard deviations are given in parentheses.

| **Bond lengths (Å)** | | **Bond angles (°)** | |
|---|---|---|---|
| Complex **1** | | Complex **1** | |
| Co1–N2 | 2.061(2) | N2–Co1–N3 | 132.45(10) |
| Co1–N3 | 2.046(2) | N2–Co1–Cl1 | 102.66(8) |
| Co1–Cl1 | 2.2901(10) | N2–Co1–Cl2 | 104.11(7) |
| Co1–Cl2 | 2.2946(9) | N3–Co1–Cl1 | 105.50(8) |
| Co2–N6 | 2.062(2) | N3–Co1–Cl2 | 100.25(8) |
| Co2–N7 | 2.046(2) | Cl1–Co1–Cl2 | 111.35(4) |
| Co2–Cl3 | 2.2718(9) | N6–Co2–N7 | 129.64(9) |
| Co2–Cl4 | 2.2805(9) | N6–Co2–Cl3 | 100.93(7) |
| | | N6–Co2–Cl4 | 104.11(7) |
| | | N7–Co2–Cl3 | 105.63(7) |
| | | N7–Co2–Cl4 | 103.47(7) |
| | | Cl3–Co2–Cl4 | 113.24(4) |
| Complex **2** | | Complex **2** | |
| Co1–N2 | 2.060(2) | N2–Co1–N3 | 134.06(10) |
| Co1–N3 | 2.045(2) | N2–Co1–Br1 | 102.77(7) |
| Co1–Br1 | 2.4423(7) | N2–Co1–Br2 | 103.47(7) |
| Co1–Br2 | 2.4433(8) | N3–Co1–Br1 | 99.57(7) |
| Co2–N6 | 2.052(2) | N3–Co1–Br2 | 105.13(8) |
| Co2–N7 | 2.054(2) | Br1–Co1–Br2 | 111.05(3) |
| Co2–Br3 | 2.4184(9) | N6–Co2–N7 | 131.77(9) |
| Co2–Br4 | 2.4234(7) | N6–Co2–Br3 | 105.05(7) |
| | | N6–Co2–Br4 | 103.53(7) |
| | | N7–Co2–Br3 | 100.73(7) |
| | | N7–Co2–Br4 | 104.31(7) |
| | | Br3–Co2–Br4 | 110.82(2) |
| Complex **3** | | Complex **3** | |
| Zn1–N2 | 2.0676(18) | N2–Zn1–N3 | 126.80(7) |
| Zn1–N3 | 2.0670(16) | N2–Zn1–Cl1 | 104.24(6) |
| Zn1–Cl1 | 2.2660(7) | N2–Zn1–Cl2 | 105.58(5) |
| Zn1–Cl2 | 2.2615(7) | N3–Zn1–Cl1 | 104.57(5) |
| Zn2–N6 | 2.0519(19) | N3–Zn1–Cl2 | 101.39(5) |
| Zn2–N7 | 2.0617(19) | Cl1–Zn1–Cl2 | 114.84(3) |
| Zn2–Cl3 | 2.2775(8) | N6–Zn2–N7 | 129.80(8) |
| Zn2–Cl4 | 2.2823(7) | N6–Zn2–Cl3 | 105.16(6) |
| | | N6–Zn2–Cl4 | 101.38(6) |
| | | N7–Zn2–Cl3 | 102.43(6) |
| | | N7–Zn2–Cl4 | 105.10(6) |
| | | Cl3–Zn2–Cl4 | 113.14(3) |
| Complex **4** | | Complex **4** | |
| Cu1–N2 | 1.972(3) | N2–Cu1–N3 | 150.65(13) |
| Cu1–N3 | 1.972(3) | N2–Cu1–Cl1 | 97.29(10) |
| Cu1–Cl1 | 2.3289(13) | N2–Cu1–Cl2 | 100.56(10) |
| Cu1–Cl2 | 2.3241(13) | N2–Cu1···Se2 | 88.06(9) |
| Cu1···Se2 | 2.8955(11) | N3–Cu1–Cl1 | 98.09(10) |
| | | N3–Cu1–Cl2 | 97.55(10) |
| | | N3–Cu1···Se2 | 65.88(9) |
| | | Cl1–Cu1–Cl2 | 109.72(5) |
| | | Cl1–Cu1···Se2 | 147.39(4) |
| | | Cl2–Cu1···Se2 | 100.75(4) |

**Table S8.** Selected bond lengths and angles for complexes **5–8**. Standard deviations are given in parentheses.

| **Bond lengths (Å)** | | **Bond angles (°)** | |
|---|---|---|---|
| Complex **5** | | Complex **5** | |
| Cu1–N2 | 1.971(5) | N2–Cu1–N3 | 153.2(2) |
| Cu1–N3 | 1.971(5) | N2–Cu1–Br1 | 99.18(15) |
| Cu1–Br1 | 2.4188(13) | N2–Cu1–Br2 | 96.50(15) |
| Cu1–Br2 | 2.5452(13) | N2–Cu1···Se1 | 66.48(15) |
| Cu1···Se1 | 2.8498(14) | N3–Cu1–Br1 | 96.71(16) |
| Cu2–N6 | 1.954(5) | N3–Cu1–Br2 | 97.61(16) |
| Cu2–N7 | 1.962(5) | N3–Cu1···Se1 | 90.43(16) |
| Cu2–Br3 | 2.4621(15) | Br1–Cu1–Br2 | 111.37(4) |
| Cu2–Br4 | 2.5308(15) | Br1–Cu1···Se1 | 154.65(4) |
| Cu2···Se3 | 2.9410(15) | Br2–Cu1···Se1 | 91.61(4) |
| | | N6–Cu2–N7 | 151.7(2) |
| | | N6–Cu2–Br3 | 94.27(17) |
| | | N6–Cu2–Br4 | 99.04(18) |
| | | N6–Cu2···Se3 | 65.11(17) |
| | | N7–Cu2–Br3 | 101.49(18) |
| | | N7–Cu2–Br4 | 95.10(18) |
| | | N7–Cu2···Se3 | 91.01(17) |
| | | Br3–Cu2–Br4 | 115.73(5) |
| | | Br3–Cu2···Se3 | 91.10(4) |
| | | Br4–Cu2···Se3 | 150.49(4) |
| Complex **6** | | Complex **6** | |
| Cu1–N2 | 2.055(3) | N2–Cu1–I1 | 107.01(8) |
| Cu1–I1 | 2.6813(6) | N2–Cu1–I2 | 107.67(8) |
| Cu1–I2 | 2.5934(6) | N2–Cu1–Se2 | 103.91(9) |
| Cu1–Se2 | 2.4703(8) | Se2–Cu1–I1 | 92.85(2) |
| Cu2–N3 | 2.078(3) | Se2–Cu1–I2 | 123.795(19) |
| Cu2–I1'' | 2.6881(6) | I1–Cu1–I2 | 119.55(2) |
| Cu2–I2 | 2.6020(8) | N3–Cu2–I1'' | 108.36(8) |
| Cu2–Se1 | 2.5212(7) | N3–Cu2–I2 | 114.18(9) |
| | | N3–Cu2–Se1 | 102.37(9) |
| | | Se1–Cu2–I1'' | 97.77(2) |
| | | Se1–Cu2–I2 | 119.86(2) |
| | | I1''–Cu1–I2 | 112.544(19) |
| Complex **7** | | Complex **7** | |
| Cu1–N2 | 1.972(3) | N2–Cu1–N3 | 147.99(12) |
| Cu1–N3 | 2.002(3) | N2–Cu1–O1 | 96.30(12) |
| Cu1–O1 | 1.944(3) | N2–Cu1–O3' | 111.47(11) |
| Cu1–O3' | 2.130(3) | N2–Cu1···Se1 | 68.83(8) |
| Cu1···Se1 | 2.6898(10) | N3–Cu1–O1 | 91.82(12) |
| | | N3–Cu1–O3' | 95.35(11) |
| | | N3–Cu1···Se1 | 94.87(9) |
| | | O1–Cu1–O3' | 107.30(11) |
| | | O1–Cu1···Se1 | 160.58(9) |
| | | O3'–Cu1···Se1 | 90.22(7) |
| Complex **8** | | Complex **8** | |
| Zn1–N2 | 2.0525(19) | N2–Zn1–N3 | 130.68(8) |
| Zn1–N3 | 2.0076(19) | N2–Zn1–O1 | 98.55(7) |
| Zn1–O1 | 1.9550(16) | N2–Zn1–O3' | 96.65(7) |
| Zn1–O3' | 1.9816(17) | N3–Zn1–O1 | 118.02(7) |
| | | N3–Zn1–O3' | 100.21(7) |
| | | O1–Zn1–O3' | 109.85(7) |

**Table S9.** Selected bond lengths and angles for complexes **9** and **10**. Standard deviations are given in parentheses.

| **Bond lengths (Å)** | | **Bond angles (°)** | |
|---|---|---|---|
| Complex **9** | | Complex **9** | |
| Ag1–N1 | 2.4606(15) | N1–Ag1–N3 | 90.71(5) |
| Ag1–N3 | 2.3778(14) | N1– Ag1–Se1 | 72.32(4) |
| Ag1–Se1 | 2.7872(4) | N1– Ag1–Se4 | 123.79(3) |
| Ag1–Se4 | 2.5811(5) | N1– Ag1–Ag2 | 156.36(3) |
| Ag1–Ag2 | 2.9315(5) | N3– Ag1–Se1 | 91.57(4) |
| Ag2–N5 | 2.5001(16) | N3– Ag1–Se4 | 129.94(4) |
| Ag2–N7 | 2.2949(14) | N3– Ag1–Ag2 | 77.12(4) |
| Ag2–Se2 | 2.5555(5) | Se1– Ag1–Se4 | 130.397(12) |
| Ag2–Se3 | 2.8868(4) | Se1– Ag1–Ag2 | 87.637(19) |
| | | Se4– Ag1–Ag2 | 78.732(16) |
| | | N5–Ag2–N7 | 94.86(5) |
| | | N5– Ag2–Se2 | 111.47(4) |
| | | N5– Ag2–Se3 | 68.46(4) |
| | | N5– Ag2–Ag1 | 155.89(4) |
| | | N7– Ag2–Se2 | 137.55(4) |
| | | N7– Ag2–Se3 | 92.29(4) |
| | | N7– Ag2–Ag1 | 85.25(4) |
| | | Se3– Ag2–Se2 | 127.688(12) |
| | | Se3– Ag2–Ag1 | 87.435(19) |
| | | Se2– Ag2–Ag1 | 83.263(18) |
| Complex **10** | | Complex **10** | |
| Ag2–N2 | 2.3689(15) | N2–Ag2–N3 | 88.09(6) |
| Ag2–N3 | 2.4243(16) | N2– Ag2–Se1’ | 131.81(4) |
| Ag2–Se1’ | 2.5563(6) | N2– Ag2–Se2 | 94.18(4) |
| Ag2–Se2 | 2.7799(5) | N2– Ag2–Ag2’ | 79.23(4) |
| Ag2–Ag2’ | 2.9240(7) | N3– Ag2–Se1’ | 120.94(4) |
| | | N3– Ag2–Se2 | 73.69(4) |
| | | N3– Ag2–Ag2’ | 156.71(4) |
| | | Se1’– Ag2–Se2 | 128.772(13) |
| | | Se1’– Ag2–Ag2’ | 81.607(13) |
| | | Se2– Ag2–Ag2’ | 87.723(14) |

**Table S10.** Geometries of the metal centers of complexes **1–10**, assigned based on the calculations of the parameters $\tau_4$ and $\tau_5$.

| **Complex** | **Parameter ($\tau_4$ /$\tau_5$)** | **Geometry (distorted)** |
|---|---|---|
| **1** | 0.82 (Co1) and 0.83 (Co2) ($\tau_4$) | trigonal pyramidal |
| **2** | 0.82 (Co1) and 0.83 (Co2) ($\tau_4$) | trigonal pyramidal |
| **3** | 0.84 (Zn1) and 0.83 (Zn2) ($\tau_4$) | trigonal pyramidal |
| **4** | 0.054 ($\tau_5$) | square pyramidal |
| **5** | 0.024 (Cu1) and 0.020 (Cu2) ($\tau_5$) | square pyramidal |
| **6** | 0.83 (Cu1) and 0.89 (Cu2) ($\tau_4$) | trigonal pyramidal |
| **7** | 0.21 ($\tau_5$) | square pyramidal |
| **8** | 0.79 ($\tau_4$) | trigonal pyramidal |
| **9** | 0.43 (Ag1) and 0.30 (Ag2) ($\tau_5$) | square pyramidal |
| **10** | 0.42 ($\tau_5$) | square pyramidal |

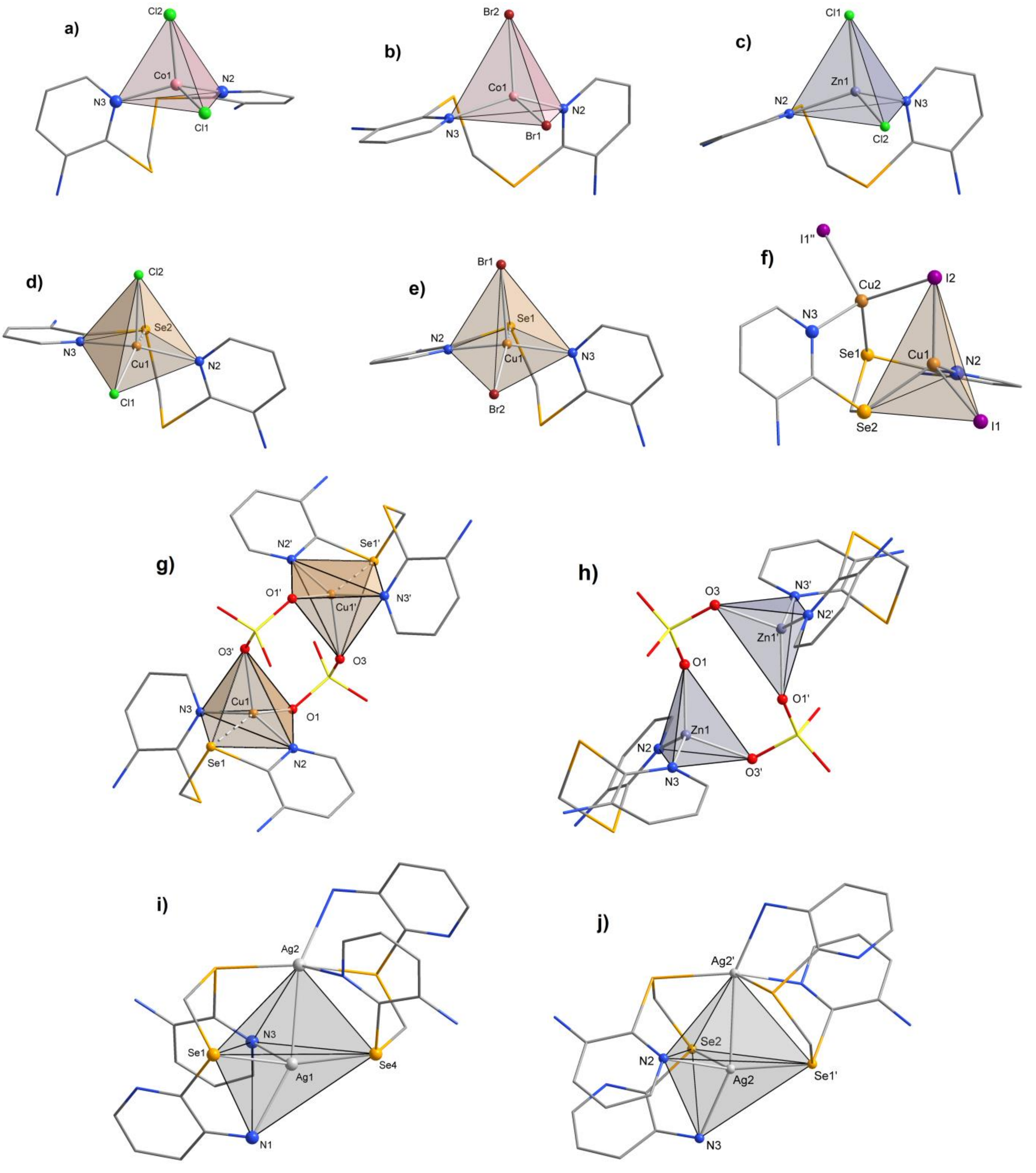


**Figure S8.** Polyhedral representation of the geometries of the metal centers of the complexes: (a) **1**; (b) **2**; (c) **3**; (d) **4**; (e) **5**; (f) **6**; (g) **7**; (h) **8**; (i) **9**; (j) **10**. For better clarity, only one independent unit of complexes **1–3**, **5**, and one polyhedron from complex **6** are shown.

## POWDER X-RAY DIFFRACTION (PXRD)

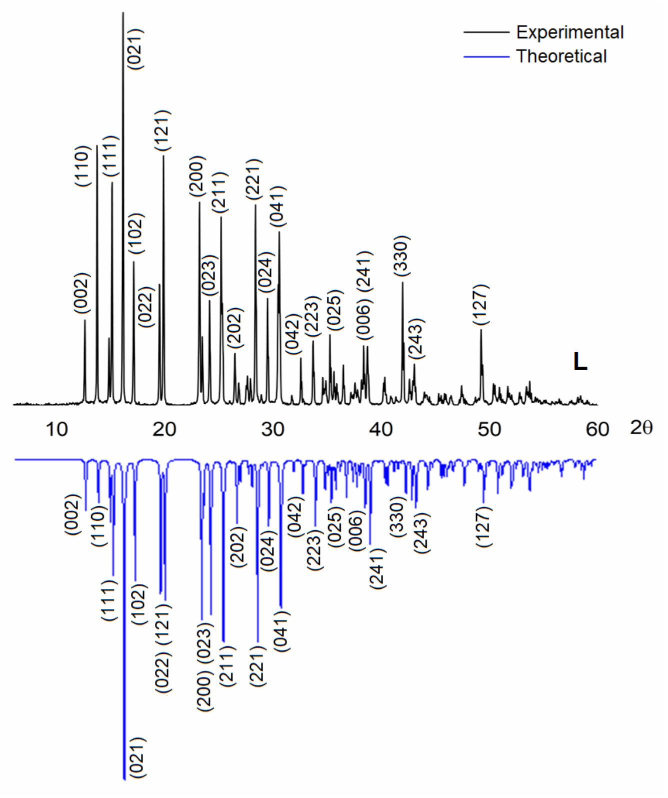


**Figure S9.** Theoretical and experimental powder diffractograms of ligand **L**.

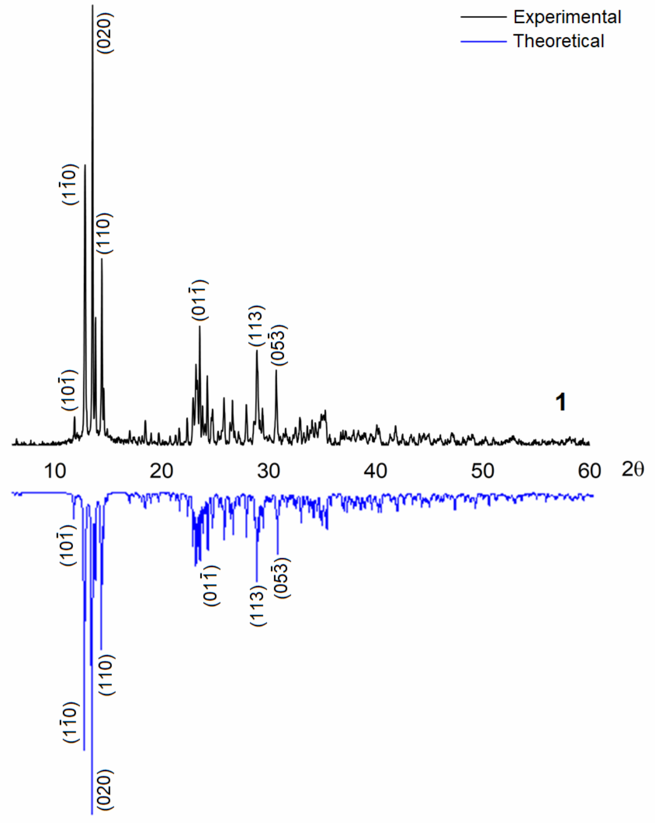


**Figure S10.** Theoretical and experimental powder diffractograms of complex **1**.

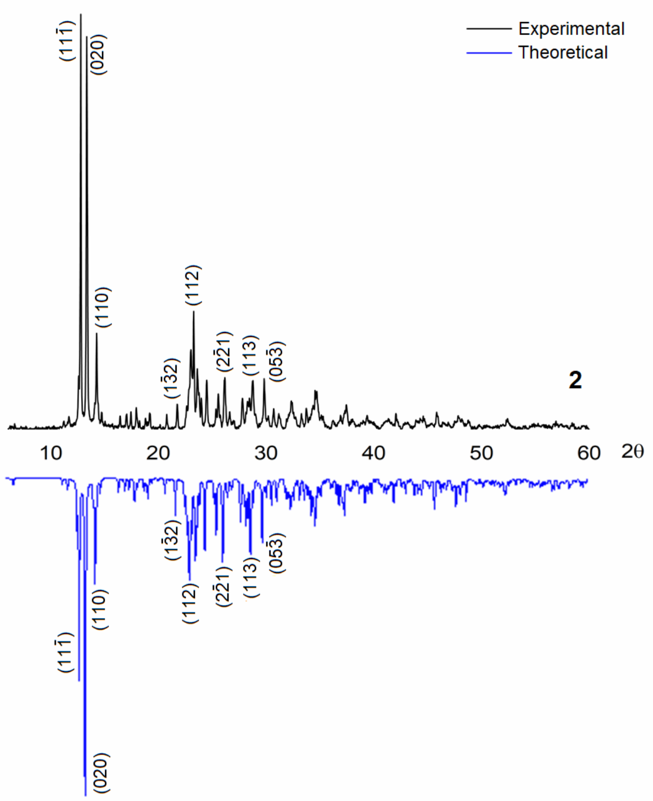


**Figure S11.** Theoretical and experimental powder diffractograms of complex **2**.

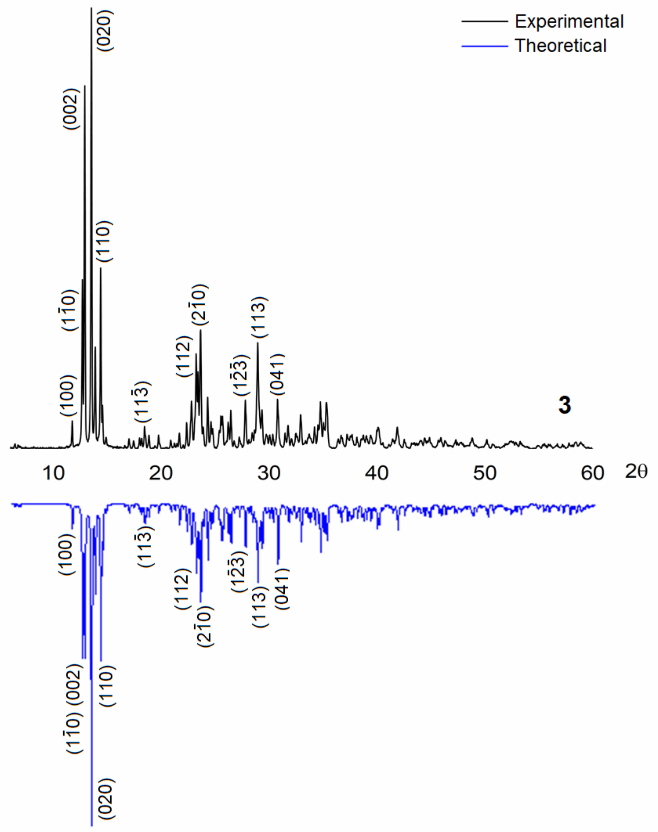


**Figure S12.** Theoretical and experimental powder diffractograms of complex **3**.

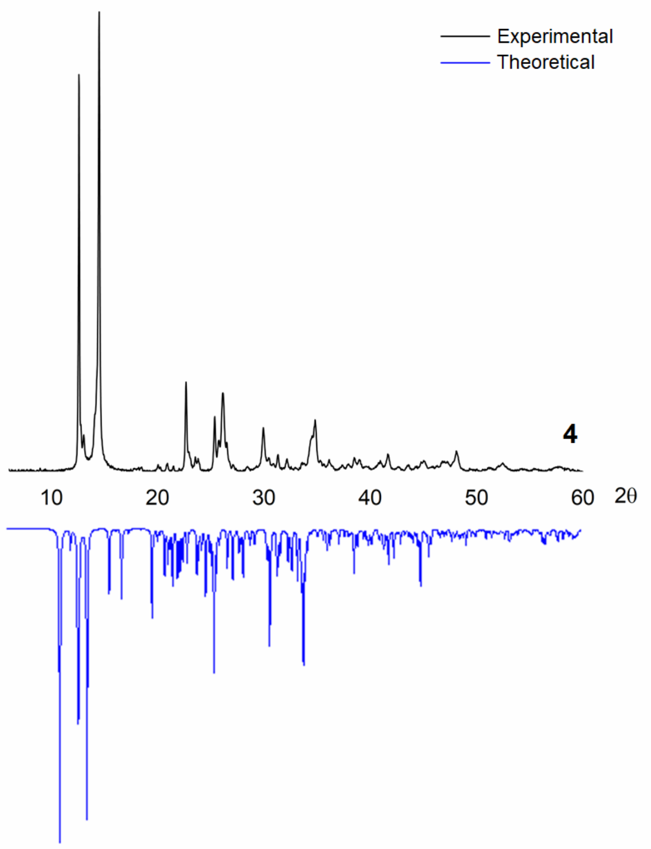


**Figure S13.** Theoretical and experimental powder diffractograms of complex **4**.

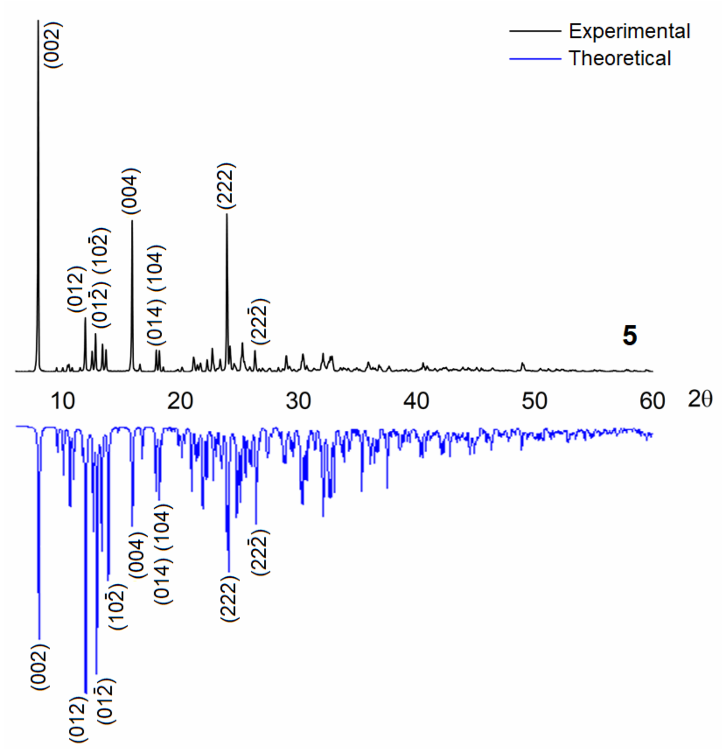


**Figure S14.** Theoretical and experimental powder diffractograms of complex **5**.

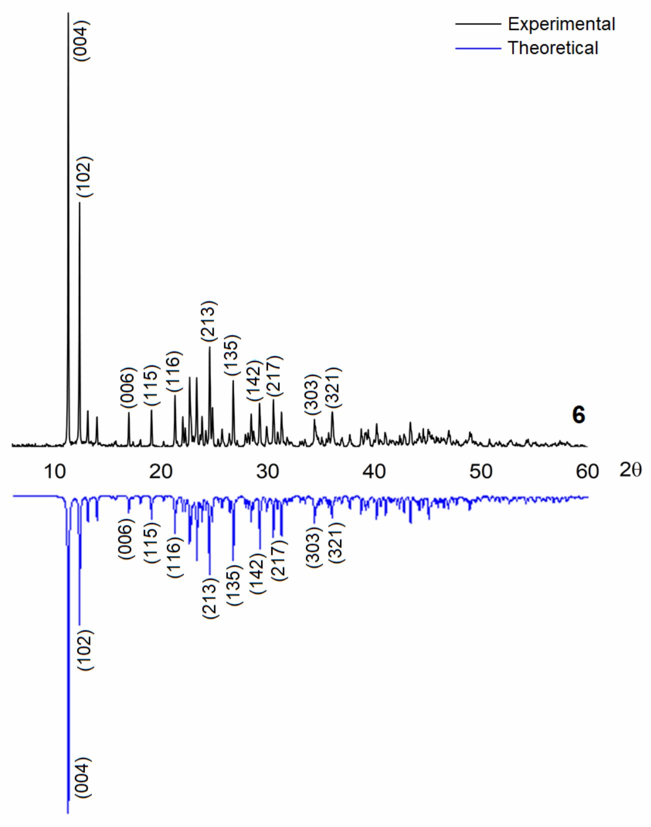


**Figure S15.** Theoretical and experimental powder diffractograms of complex **6**.

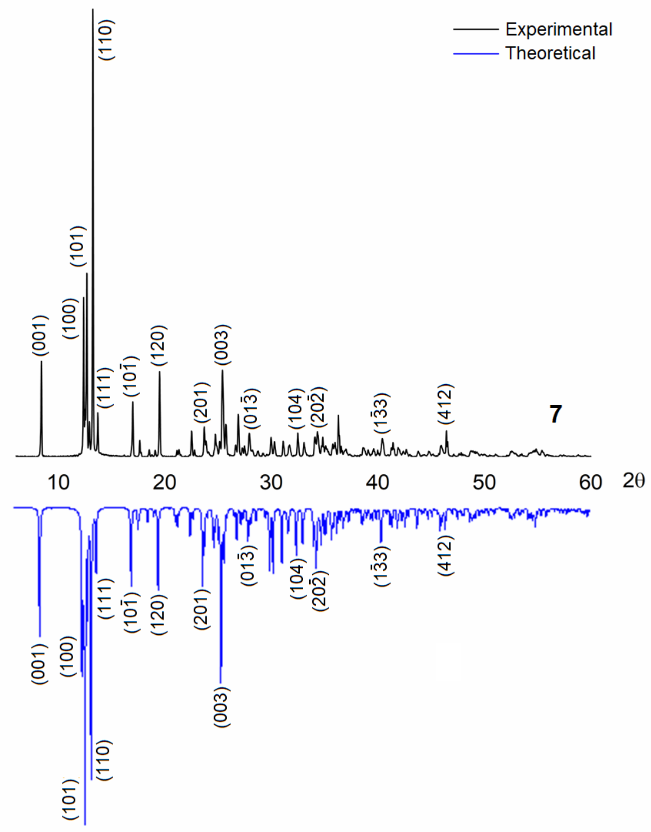


**Figure S16.** Theoretical and experimental powder diffractograms of complex **7**.

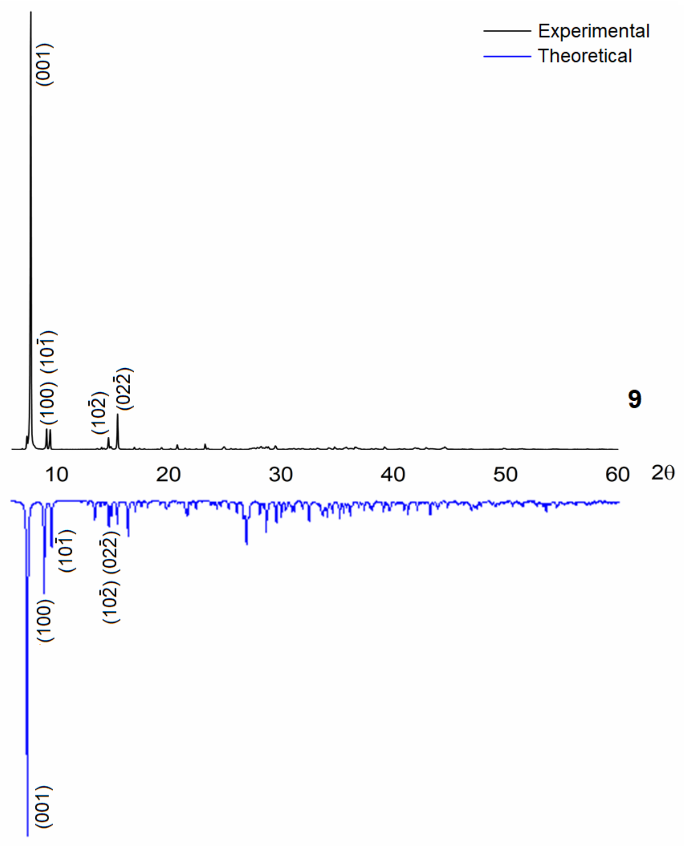


**Figure S17.** Theoretical and experimental powder diffractograms of complex **9**.

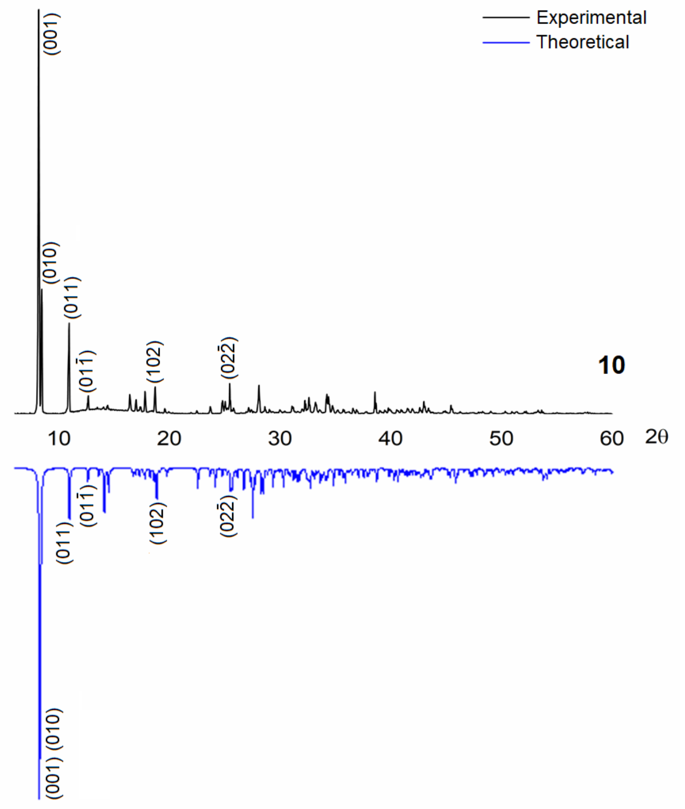


**Figure S18.** Theoretical and experimental powder diffractograms of complex **10**.

## VIBRATIONAL SPECTROSCOPY (FT-IR AND RAMAN)

**Table S11.** Main bands observed (in $cm^{-1}$) in the FT-IR spectra of ligand **L** and complexes **1–10**.

| **Assignments** | **L** | **1** | **2** | **3** | **4** | **5** | **6** |
|---|---|---|---|---|---|---|---|
| $\nu_a(NH_2)$ | 3389 | 3406 | 3419, 3402 | 3387 | 3393 | 3398, 3372 | 3451, 3429 |
| $\nu_s(NH_2)$ | 3295 | 3314, 3280 | 3312 | 3300 | 3307, 3255 | 3305, 3287 | 3295, 3284 |
| $\delta_{ot}(NH_2)$ | 3175 | 3219, 3204, 3191 | 3201, 3183 | 3204, 3177 | 3218, 3195, 3162 | 3210, 3183 | 3186, 3159 |
| $\nu(=C–H)$ | 3053 | 3065 | 3063 | 3083 | 3066 | 3071 | 3060 |
| $\nu_a(CH_2)$ | 3019 | 2997 | 2999 | 3038 | 3038 | 2997 | 3011 |
| $\nu_s(CH_2)$ | 2920 | 2929 | 2927 | 2932 | 2984 | 2942 | 2936 |
| $\nu(C=N)$ | 1621 | 1609 | 1608 | 1609 | 1623 | 1613, 1596 | 1602 |
| $\nu(C=C)$ | 1561, 1449, 1423 | 1566, 1455, 1428 | 1566, 1458, 1427 | 1568, 1454, 1432 | 1561, 1469, 1454 | 1579, 1463, 1425 | 1559, 1449, 1421 |
| $\nu(C–NH_2)$ | 1306 | 1318 | 1318 | 1321 | 1316 | 1329 | 1317 |
| $\delta_{ip}(NH_2)$ | 1045 | 1043 | 1042 | - | - | - | - |
| $\delta(=C–H)$ | 789 | 798 | 795 | 807 | 790 | 797 | 799 |
| $\delta_{oop}(NH_2)$ | 663 | 685 | 684 | - | - | 685 | 672 |
| $\nu(H_2C–Se)$ | 549 | - | - | - | - | - | - |
| $\delta_{ip}(C–NH_2)$ | 442 | 457 | 454 | 447 | 458 | 445 | 449 |
| $\nu(Co–N)$ | - | 327 | 326 | - | - | - | - |
| $\nu(Cu–N)$ | - | - | - | - | 243 | 244 | 312 |
| $\nu(Zn–N)$ | - | - | - | 325 | - | - | - |
| $\nu(Co–Cl)$ | - | 222 | - | - | - | - | - |
| $\nu(Co–Br)$ | - | - | 187 | - | - | - | - |
| $\nu(Zn–Cl)$ | - | - | - | 214 | - | - | - |
| $\nu(Cu–I)$ | - | - | - | - | - | - | 152 |

| **Assignments** | **L** | **7** | **8** | **9** | **10** |
|---|---|---|---|---|---|
| $\nu(H_2O)^{\#}$ | - | - | - | 3600 – 2800 | 3569 |
| $\nu_a(NH_2)$ | 3389 | 3400, 3368 | 3407, 3384 | 3418 | 3425 |
| $\nu_s(NH_2)$ | 3295 | 3313 | 3323, 3298 | 3329 | 3328 |
| $\delta_{ot}(NH_2)$ | 3175 | 3205 | 3223, 3205 | 3215, 3193 | 3220, 3199 |
| $\nu(=C–H)$ | 3053 | 3103, 3087 | 3088 | 3023 | 3044 |
| $\nu_a(CH_2)$ | 3019 | 3067 | 3072 | 2947 | 3021 |
| $\nu_s(CH_2)$ | 2920 | 2999 | 2294 | - | 2949 |
| $\nu(C=N)$ | 1621 | 1640, 1620 | 1620 | 1628 | 1627 |
| $\nu(C=C)$ | 1561, 1449, 1423 | 1594, 1477, 1462, 1439, 1427 | 1589, 1466, 1430 | 1576, 1463, 1413 | 1576 |
| $\nu(SO_4)$ | - | 1179 – 964* | 1210 – 974* | 1050 | - |
| $\nu(NO_3)$ | - | - | - | - | 1327 |
| $\delta(=C–H)$ | 789 | 813 | 809 | 793 | 795 |
| $\delta(SO_4)$ | - | 685 – 571* | 647 – 567* | 609 | - |
| $\delta(NO_3)$ | - | - | - | - | 960 |
| $\delta_{oop}(NH_2)$ | 663 | - | - | - | 659 |
| $\delta_{ip}(C–NH_2)$ | 442 | 465 | 468 | 458 | 457 |
| $\nu(Cu–N)$ | - | 231 | - | - | - |
| $\nu(Zn–N)$ | - | - | 337, 323 | - | - |
| $\nu(Ag–N)$ | - | - | - | 293 | 315 |
| $\nu(Ag–Se)$ | - | - | - | 173 | 186 |

$\nu$ = stretching; $\nu_a$ = asymmetric stretching; $\nu_s$ = symmetric stretching; $\delta$ = bending; $\delta_{ip}$ = in-plane bending; $\delta_{oop}$ = out-of-plane bending; $\delta_{ot}$ = overtone bending. $^{\#}$crystallization waters; *spectral absorption range of the sulfate.

**Table S12.** Main bands observed (in $cm^{-1}$) in the Raman spectra of ligand **L** and complexes **1–10**.

| **Assignments** | **L** | **1** | **2** | **3** | **4** | **5** | **6** |
|---|---|---|---|---|---|---|---|
| $\nu$(C=N) | 1623 | 1602 | 1600 | 1601 | - | - | - |
| $\nu$(C=C) | 1570, 1448, 1425 | 1578, 1459, 1431 | 1577, 1457, 1429 | 1577, 1459, 1431 | - | - | 1451, 1426 |
| $\nu$(C–$NH_2$) | 1305 | 1323 | 1322 | 1323 | - | - | 1319 |
| $\delta_{ip}$($NH_2$) | 1047 | 1046 | 1046 | 1046 | 1044 | 1043 | 1041 |
| $\delta$(=C–H) | 793 | 797 | 797 | 800 | - | - | - |
| $\delta_{oop}$($NH_2$) | 665 | 684 | 683 | 685 | 683 | 686 | 674 |
| $\nu$($H_2$C–Se) | 545 | 541 | 544 | 542 | 543 | 539 | 540 |
| $\delta_{ip}$(C–$NH_2$) | 452 | 447 | 448 | 446 | - | - | 451 |
| $\nu$(Co–N) | - | 320 | 320 | - | - | - | - |
| $\nu$(Cu–N) | - | - | - | - | 248 | 244 | 313 |
| $\nu$(Zn–N) | - | - | - | 320 | - | - | - |
| $\nu$(Co–Cl) | - | 224 | - | - | - | - | - |
| $\nu$(Co–Br) | - | - | 190 | - | - | - | - |
| $\nu$(Zn–Cl) | - | - | - | 218 | - | - | - |
| $\nu$(Cu–I) | - | - | - | - | - | - | 155 |

| **Assignments** | **L** | **7** | **8** | **9** | **10** |
|---|---|---|---|---|---|
| $\nu$(C=N) | 1623 | - | 1620 | - | - |
| $\nu$(C=C) | 1570, 1448, 1425 | 1463, 1428 | 1579, 1465, 1431 | 1423 | 1354 |
| $\nu$(C–$NH_2$) | 1305 | 1332 | 1328 | - | - |
| $\delta_{ip}$($NH_2$) | 1047 | - | 1051 | 1036 | 1044 |
| $\nu$($SO_4$) | - | - | - | 966 | - |
| $\delta$(=C–H) | 793 | - | 809 | 802 | - |
| $\delta_{oop}$($NH_2$) | 665 | 686 | 686 | 663 | 666 |
| $\delta$($SO_4$) | - | - | - | 632 | - |
| $\nu$($H_2$C–Se) | 545 | 540 | 543 | 538 | 540 |
| $\delta_{ip}$(C–$NH_2$) | 452 | - | - | 458 | 455 |
| $\nu$(Cu–N) | - | 245 | - | - | - |
| $\nu$(Zn–N) | - | - | 337, 324 | - | - |
| $\nu$(Ag–N) | - | - | - | 301 | 311 |
| $\nu$(Ag–Se) | - | - | - | 182 | 180 |

$\nu$ = stretching; $\nu_a$ = asymmetric stretching; $\nu_s$ = symmetric stretching; $\delta$ = bending; $\delta_{ip}$ = in-plane bending; $\delta_{oop}$ = out-of-plane bending.

**FT-IR spectra**

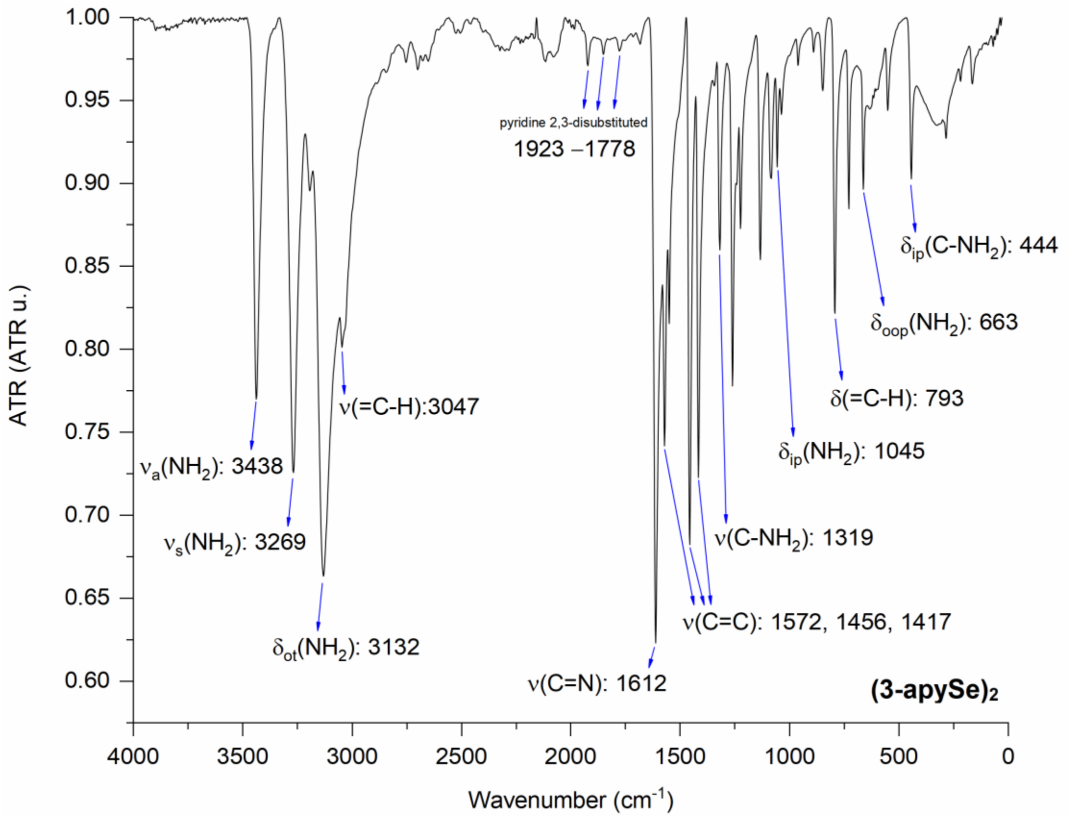


**Figure S19.** FT-IR spectrum of $\mathbf{(3\text{-}apySe)_2}$.

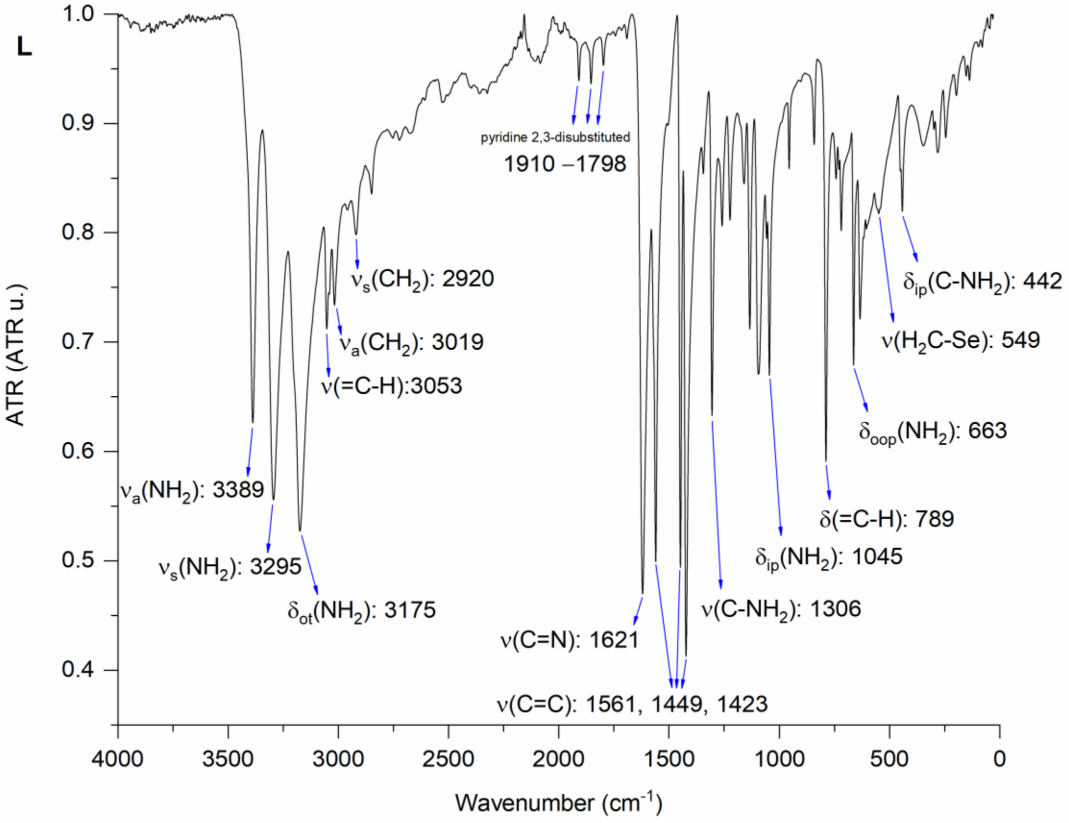


**Figure S20.** FT-IR spectrum of ligand **L**.

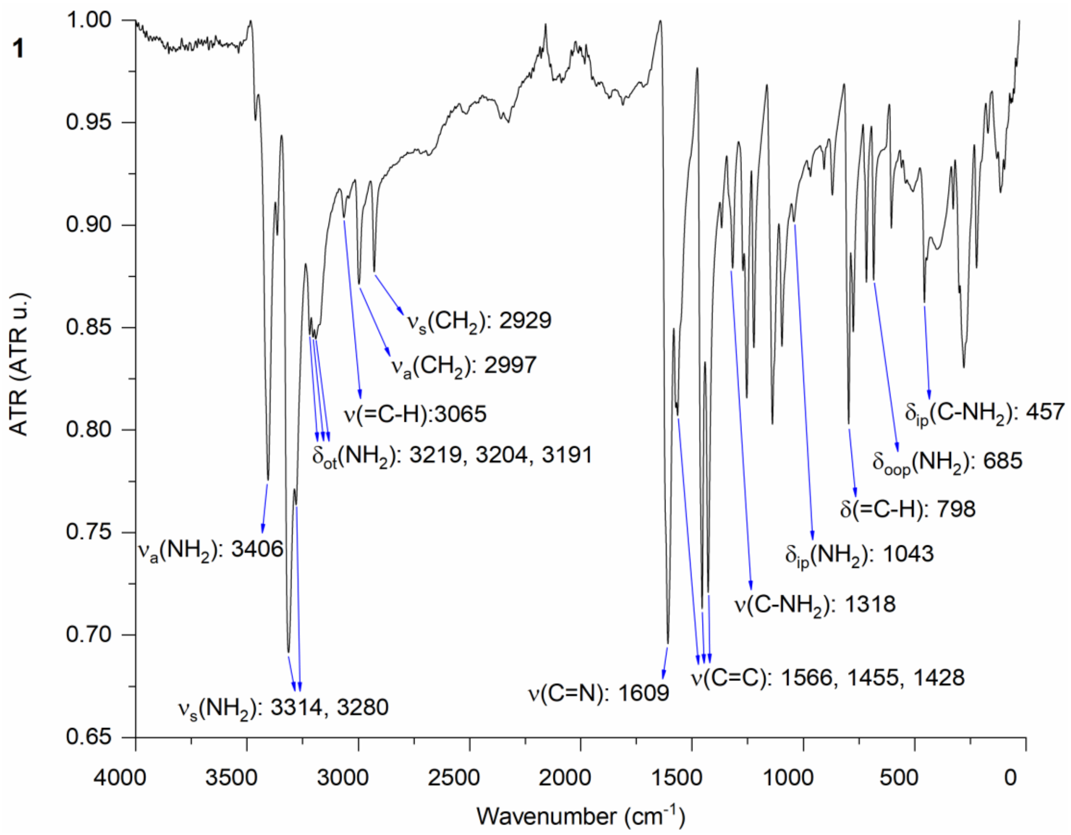


**Figure S21.** FT-IR spectrum of complex **1**.

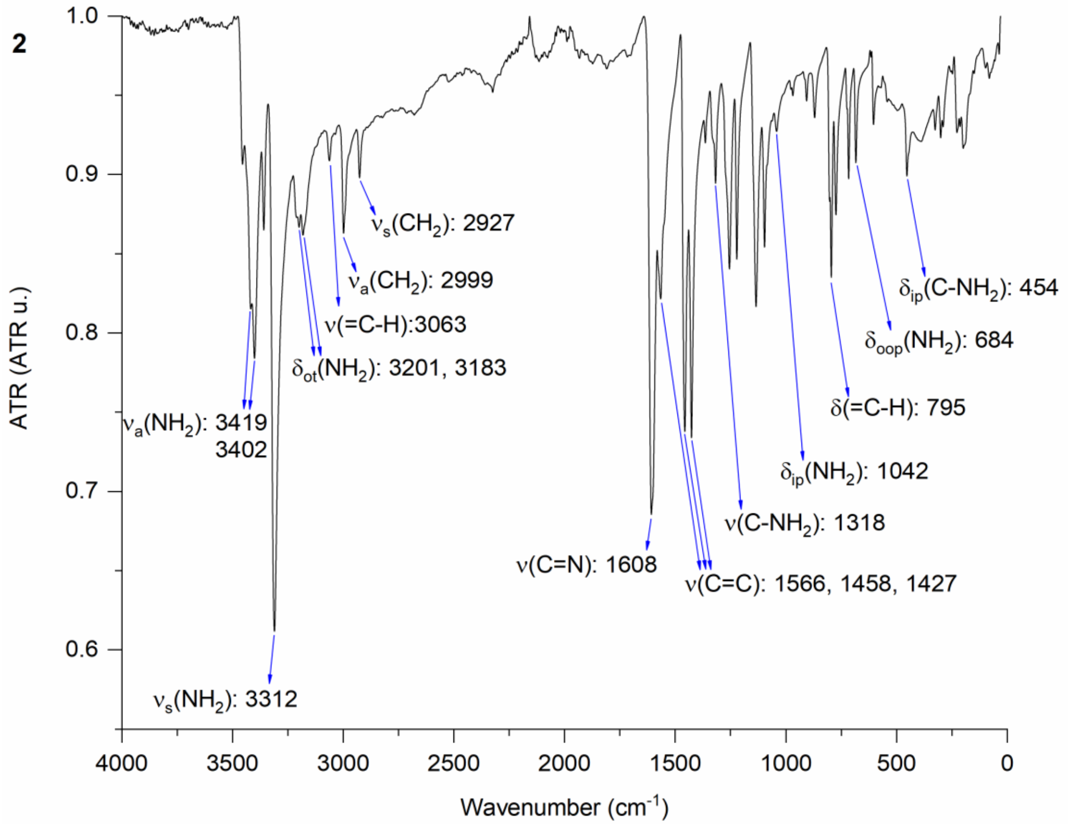


**Figure S22.** FT-IR spectrum of complex **2**.

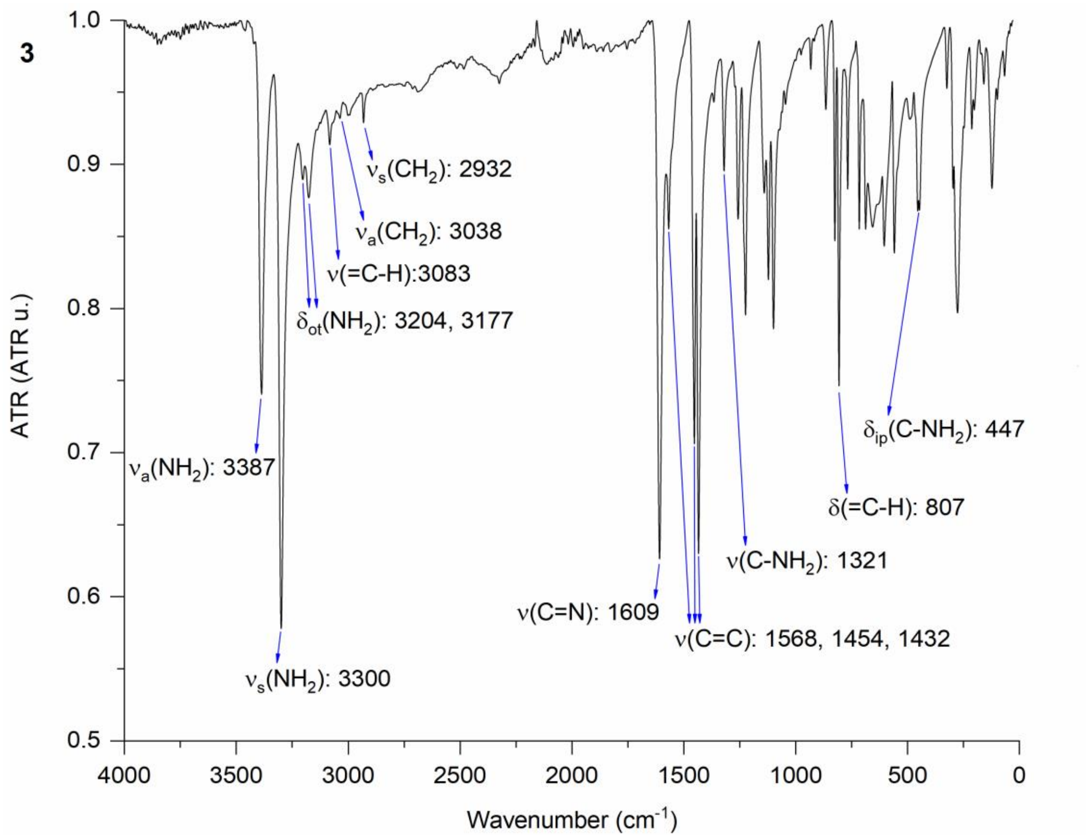


**Figure S23.** FT-IR spectrum of complex **3**.

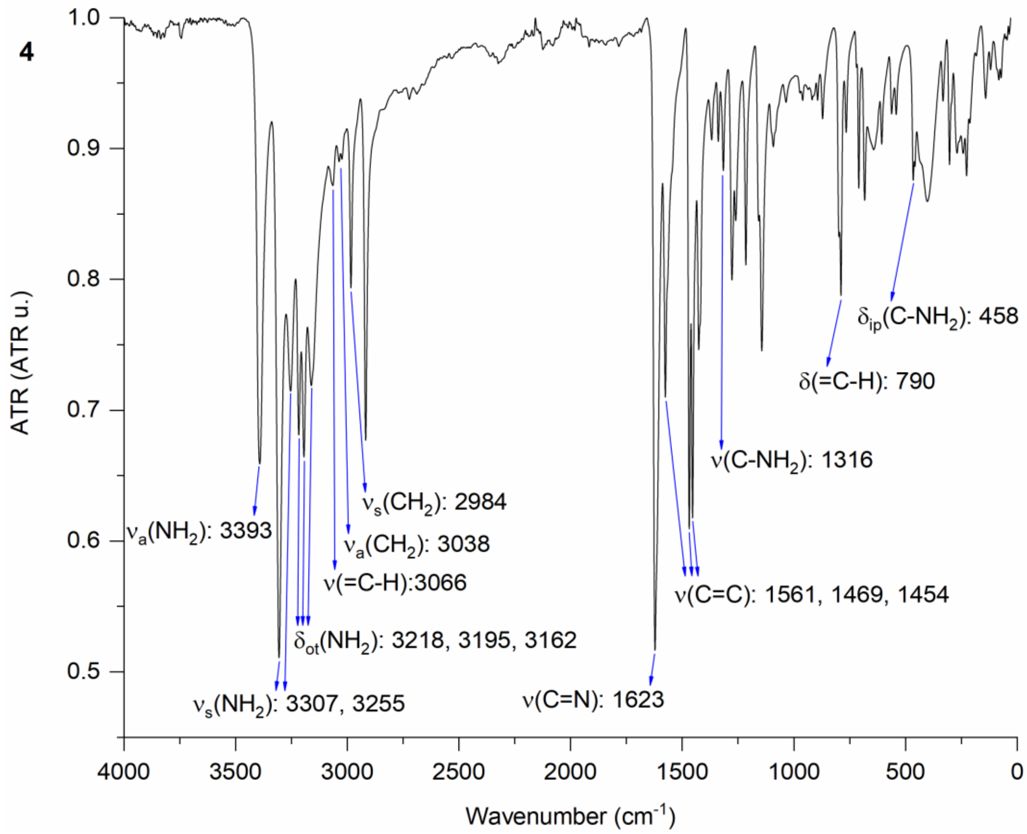


**Figure S24.** FT-IR spectrum of complex **4**.

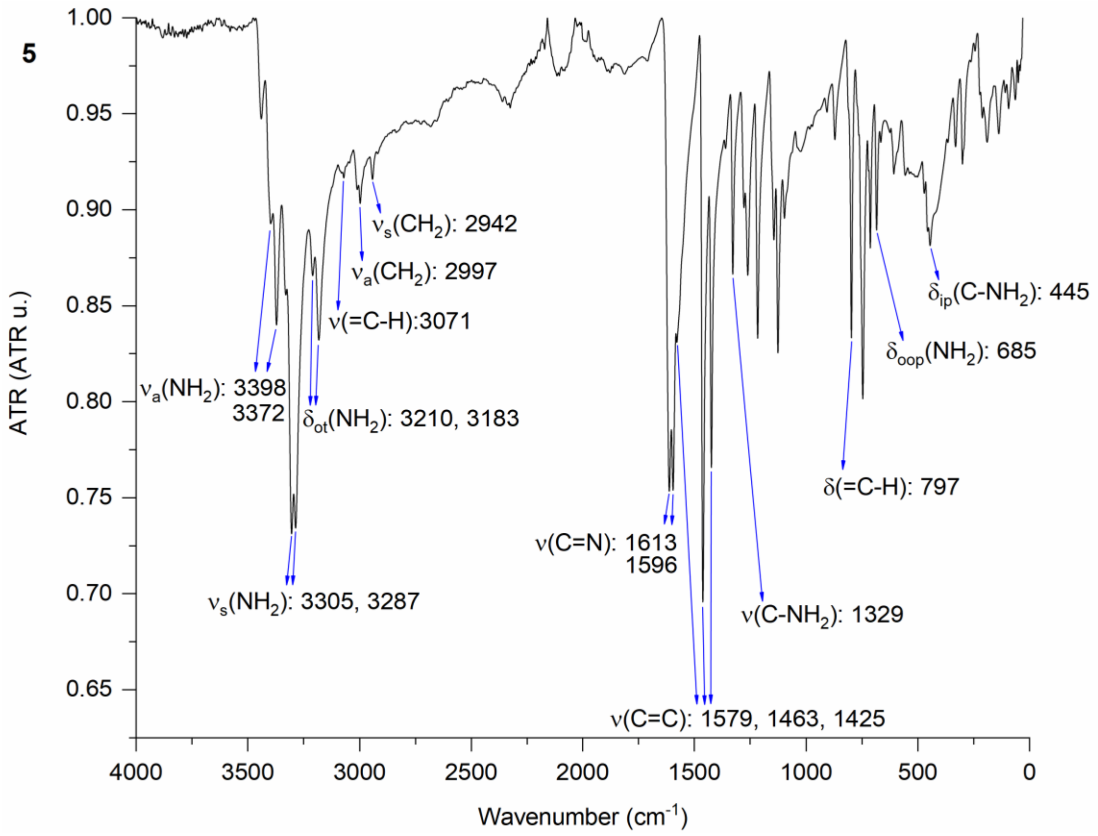


**Figure S25.** FT-IR spectrum of complex **5**.

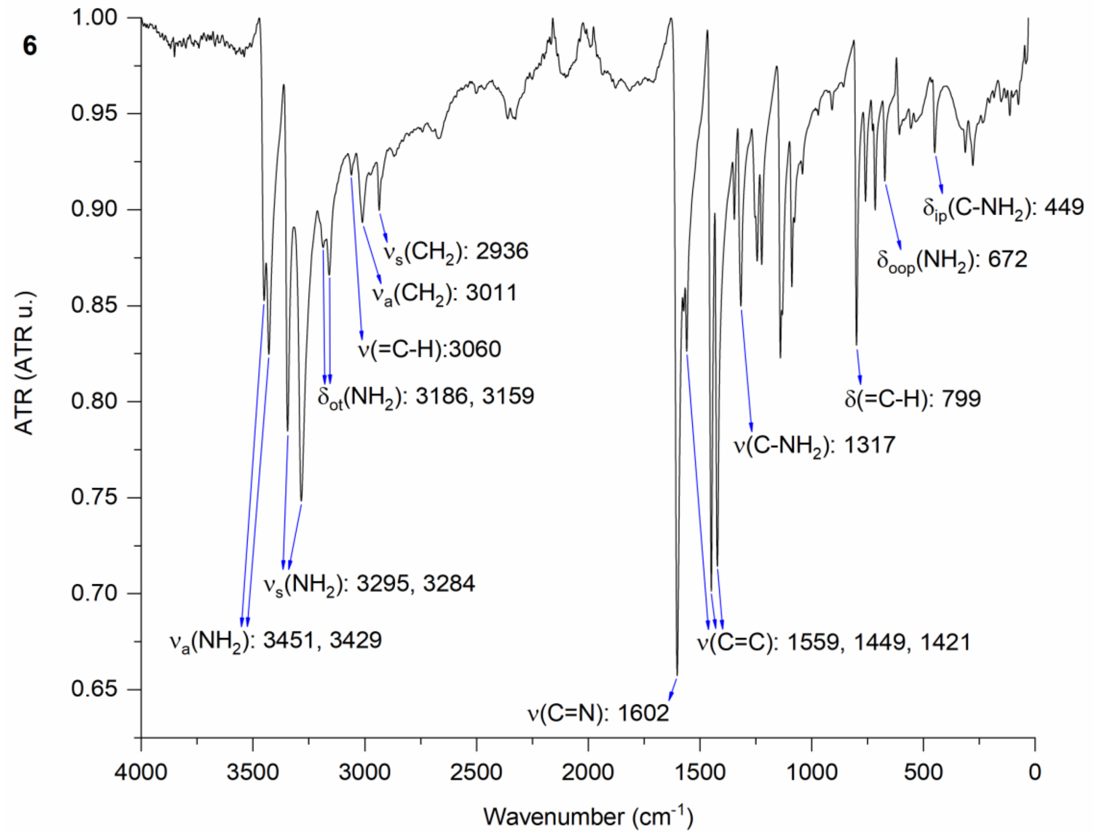


**Figure S26.** FT-IR spectrum of complex **6**.

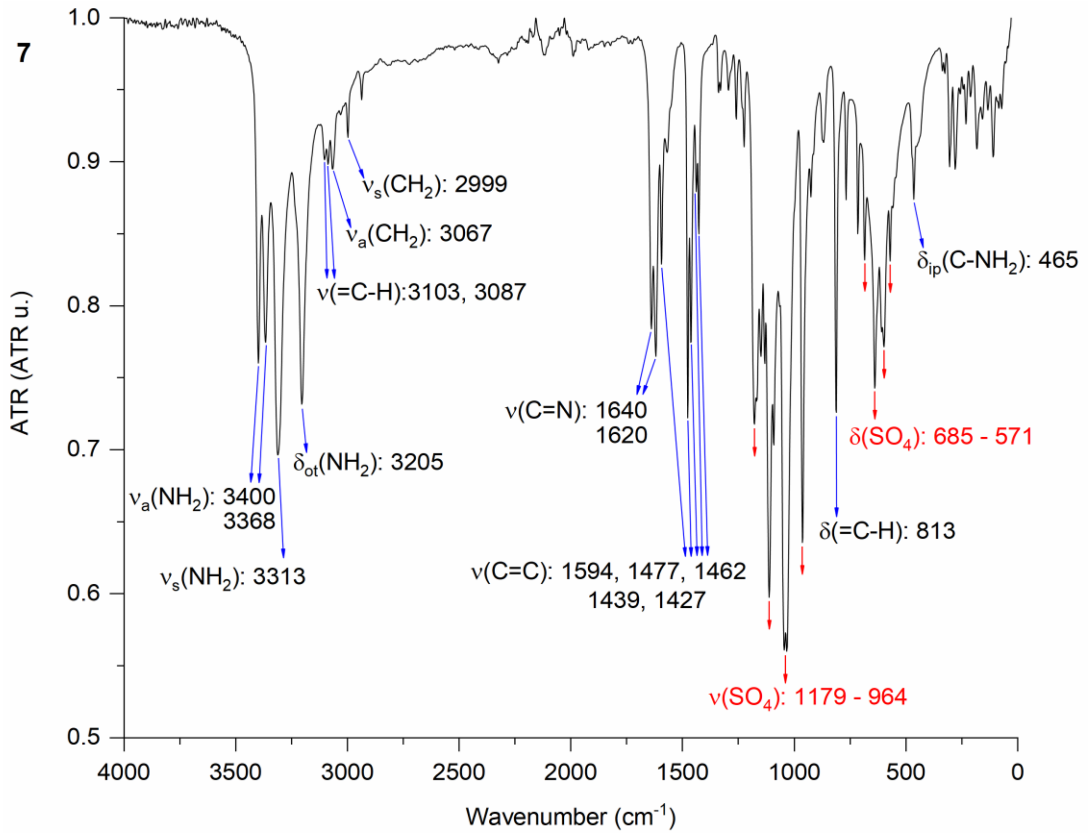


**Figure S27.** FT-IR spectrum of complex **7**.

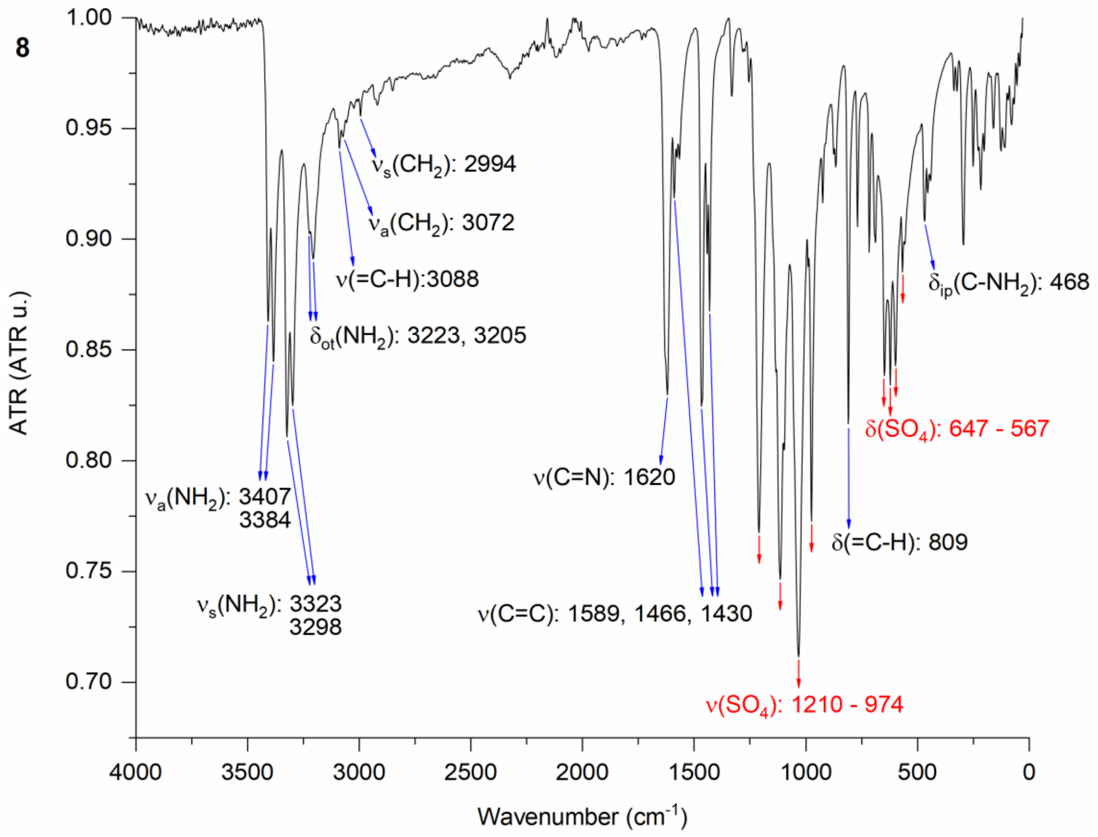


**Figure S28.** FT-IR spectrum of complex **8**.

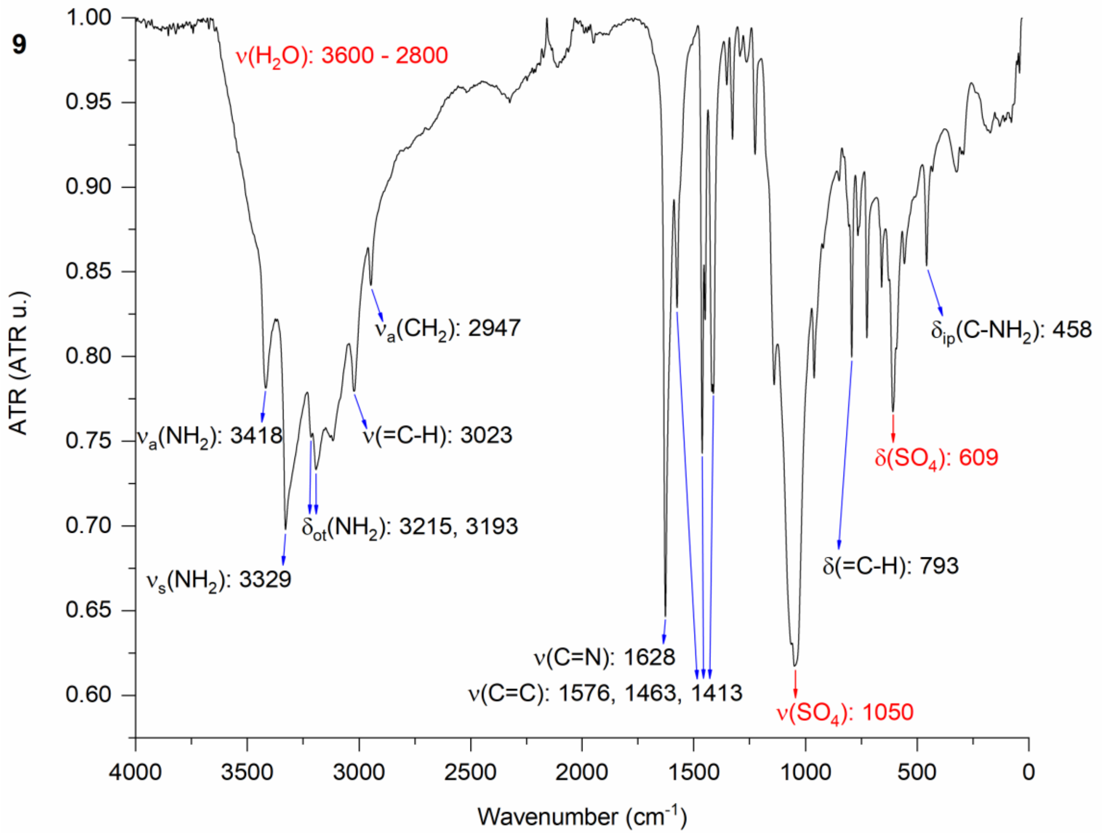


**Figure S29.** FT-IR spectrum of complex **9**.

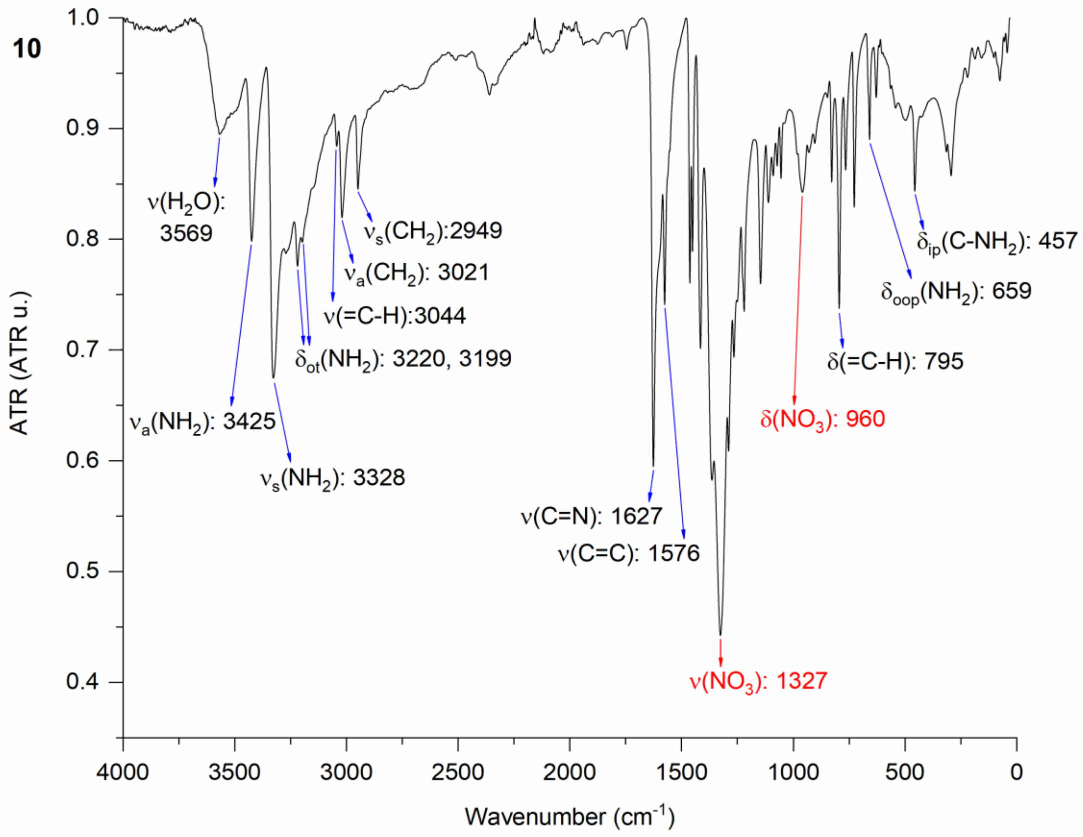


**Figure S30.** FT-IR spectrum of complex **10**.

**Raman spectra**

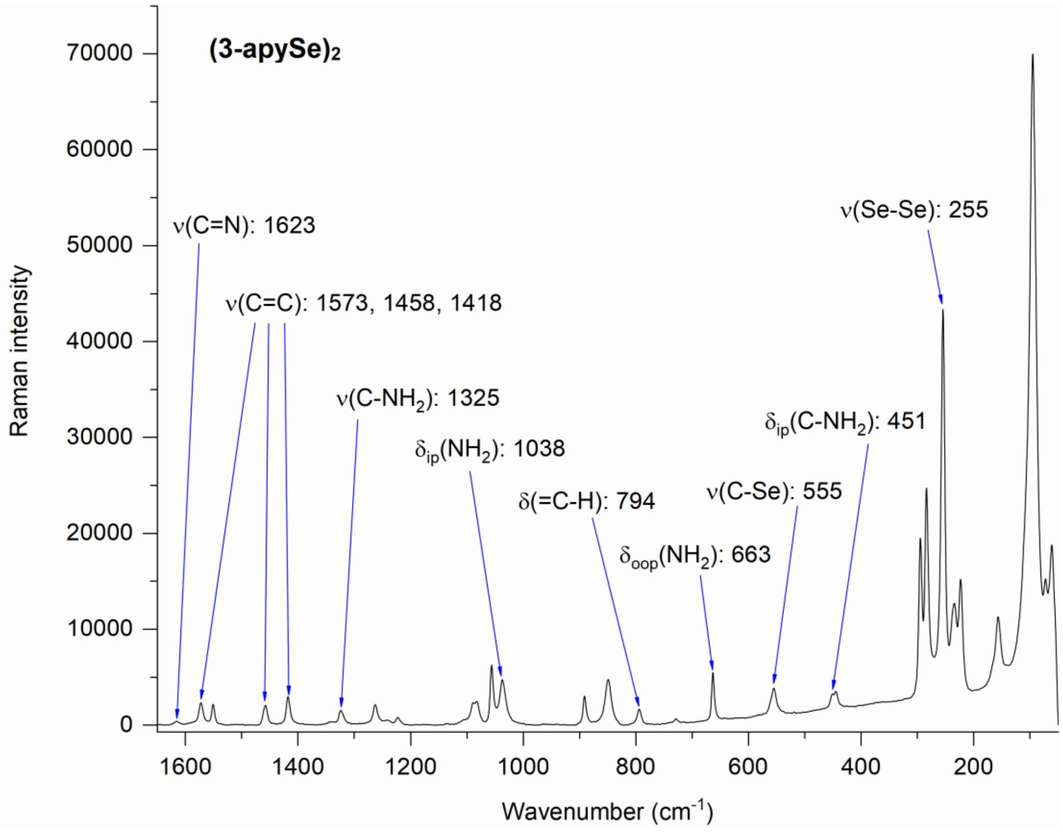


**Figure S31.** Raman spectrum of **(3-apySe)**$_2$.

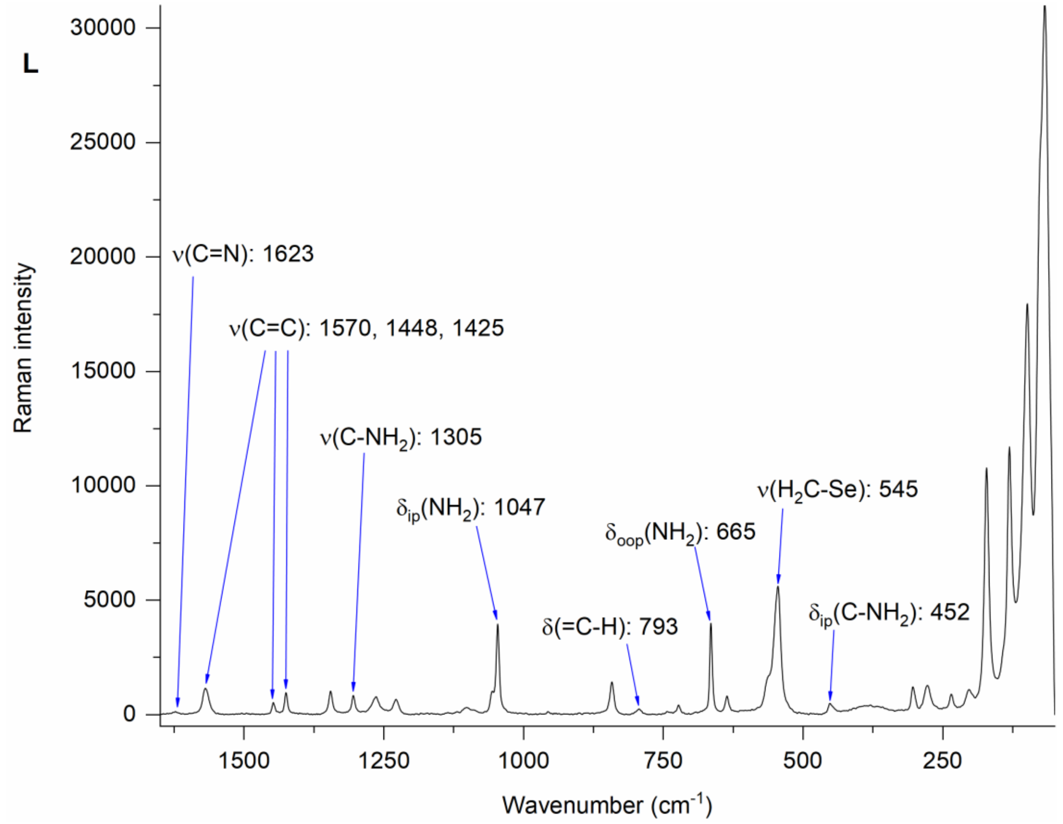


**Figure S32.** Raman spectrum of ligand **L**.

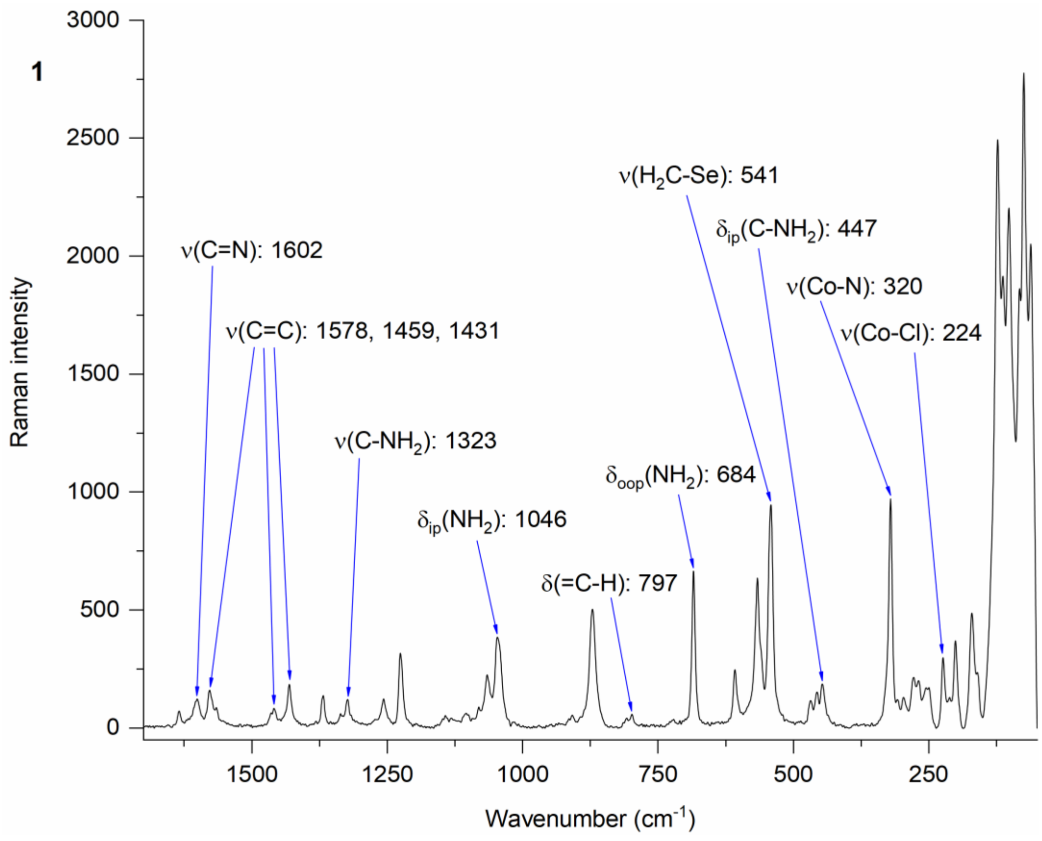


**Figure S33.** Raman spectrum of complex **1**.

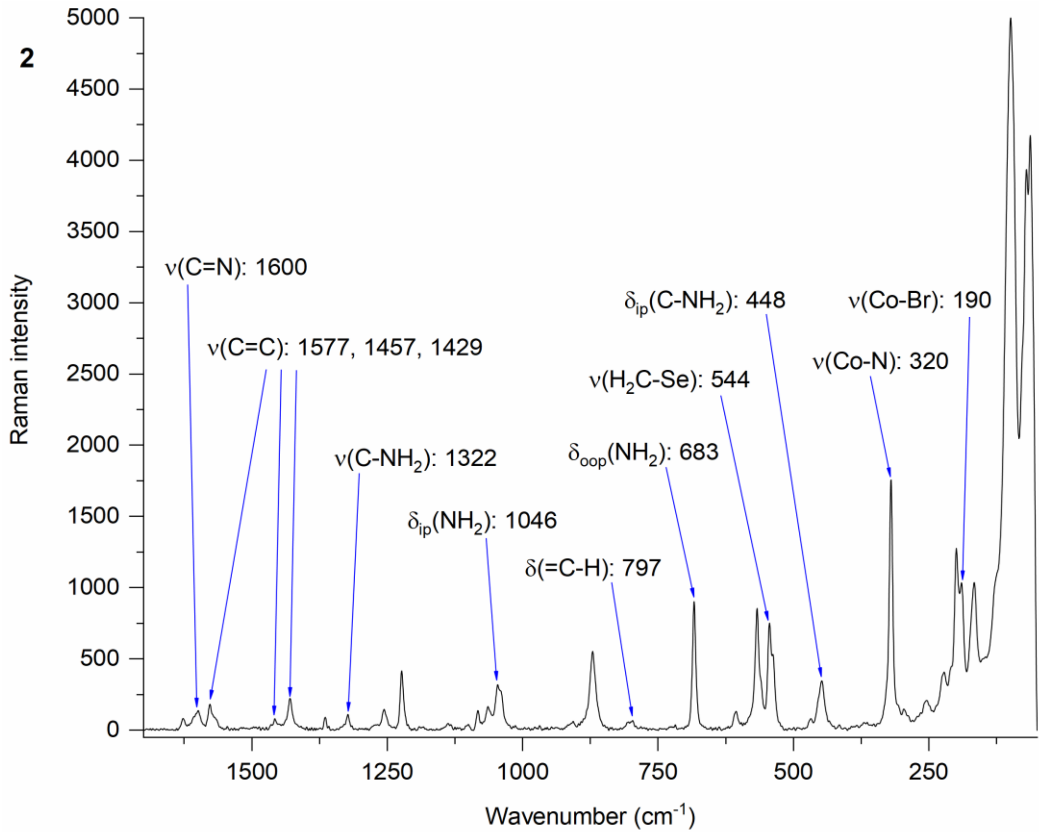


**Figure S34.** Raman spectrum of complex **2**.

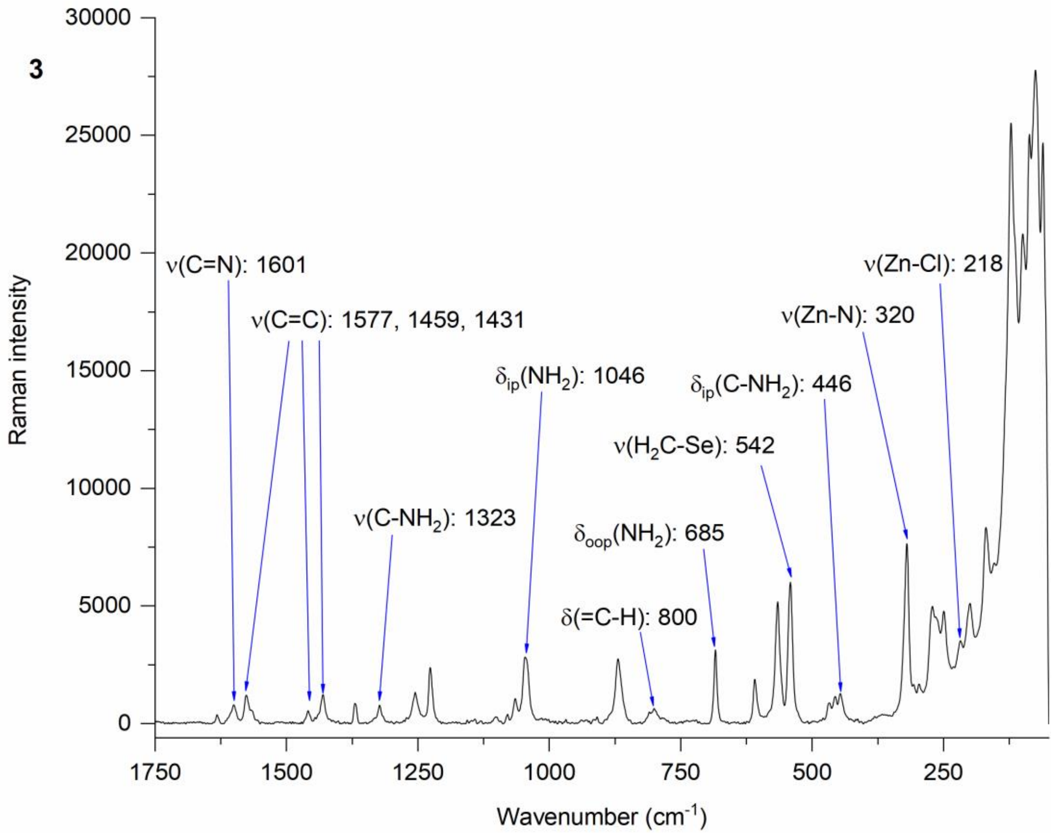


**Figure S35.** Raman spectrum of complex **3**.

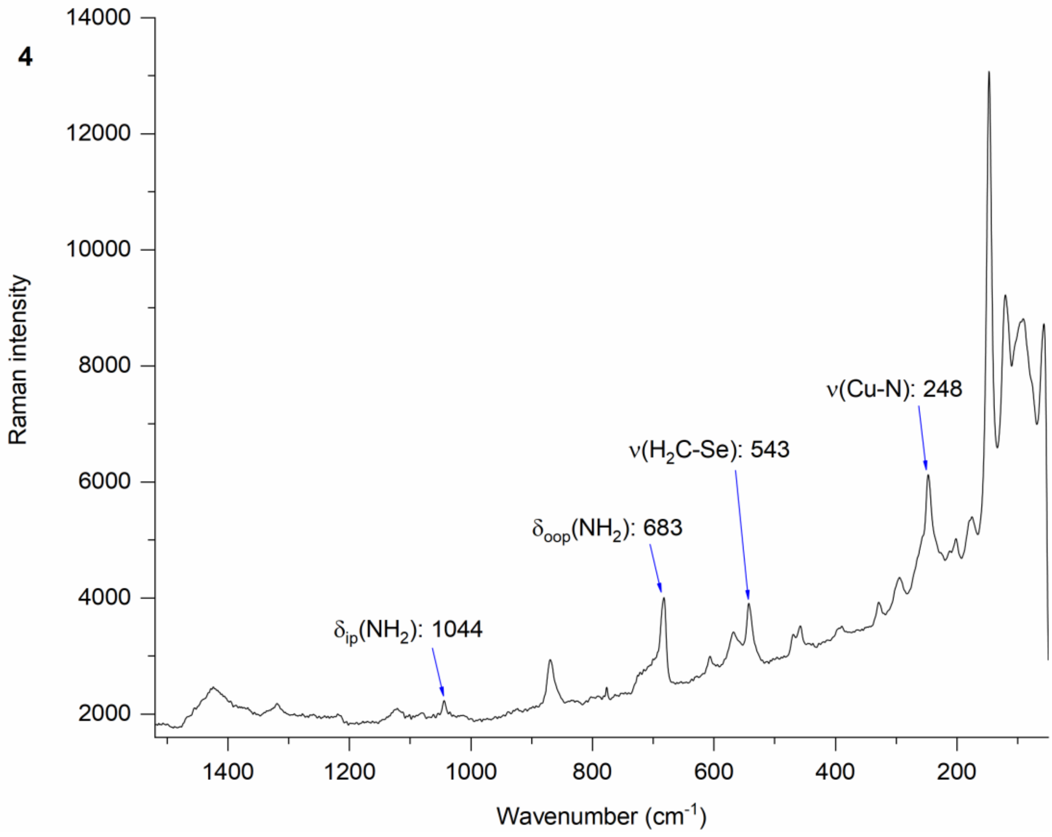


**Figure S36.** Raman spectrum of complex **4**.

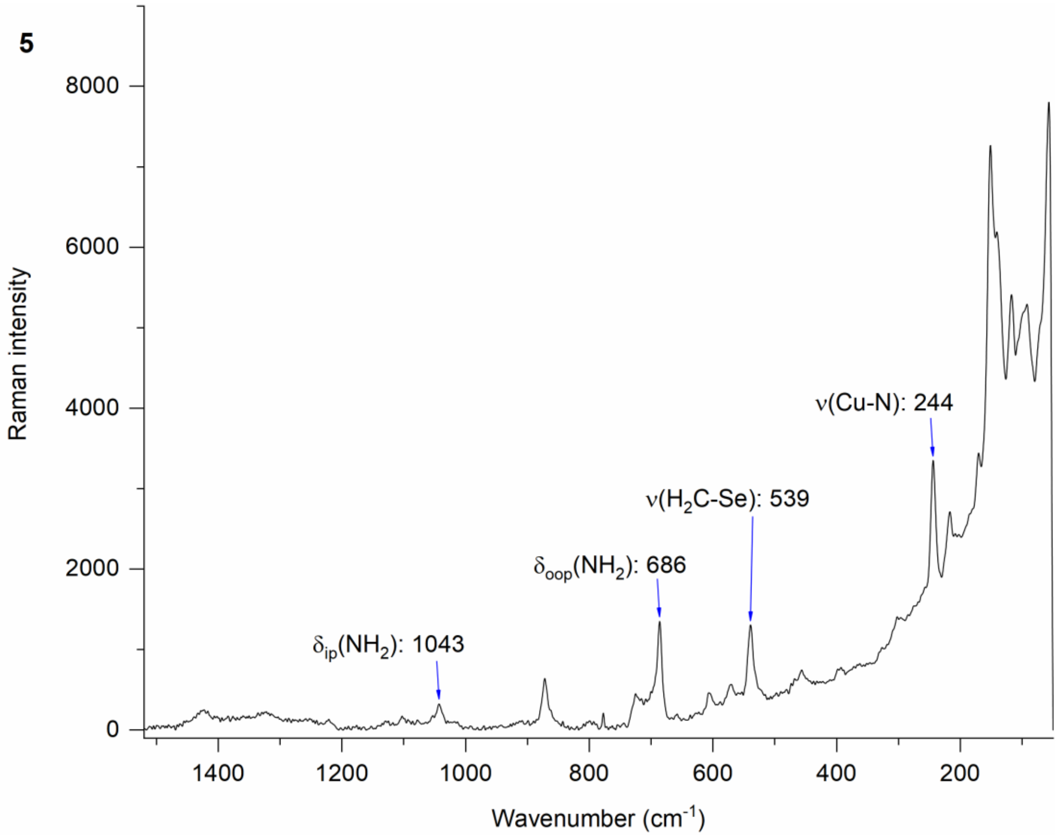


**Figure S37.** Raman spectrum of complex **5**.

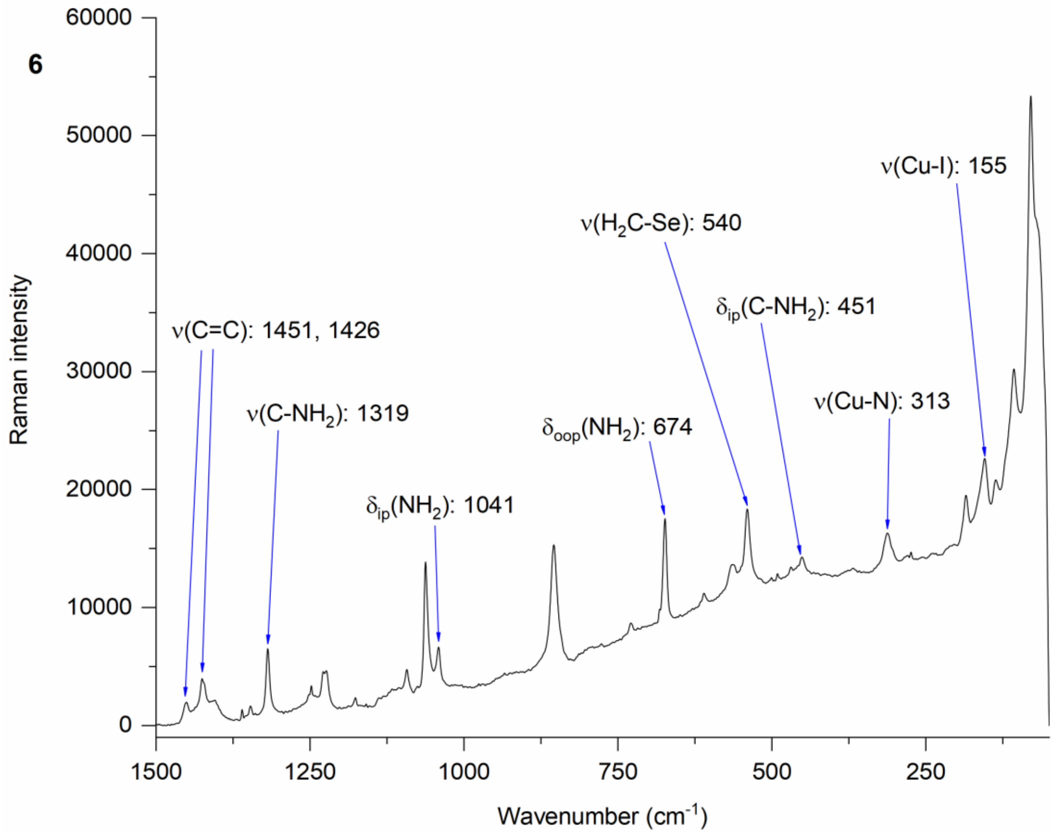


**Figure S38.** Raman spectrum of complex **6**.

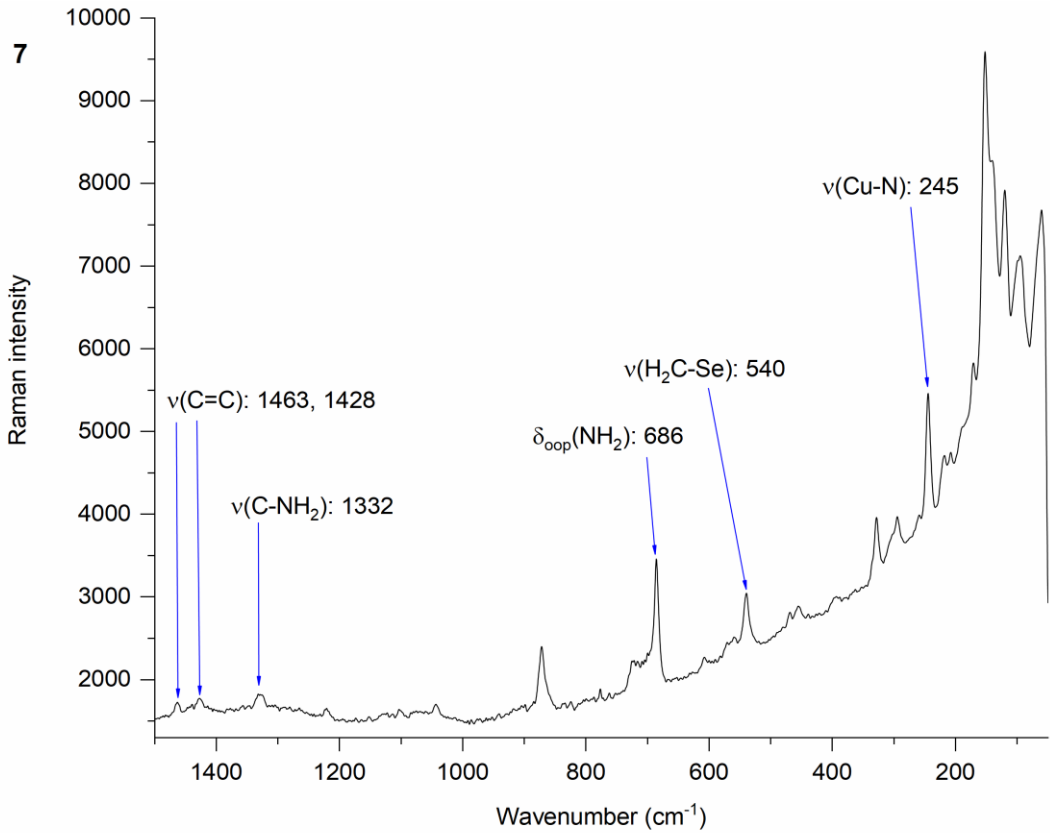


**Figure S39.** Raman spectrum of complex **7**.

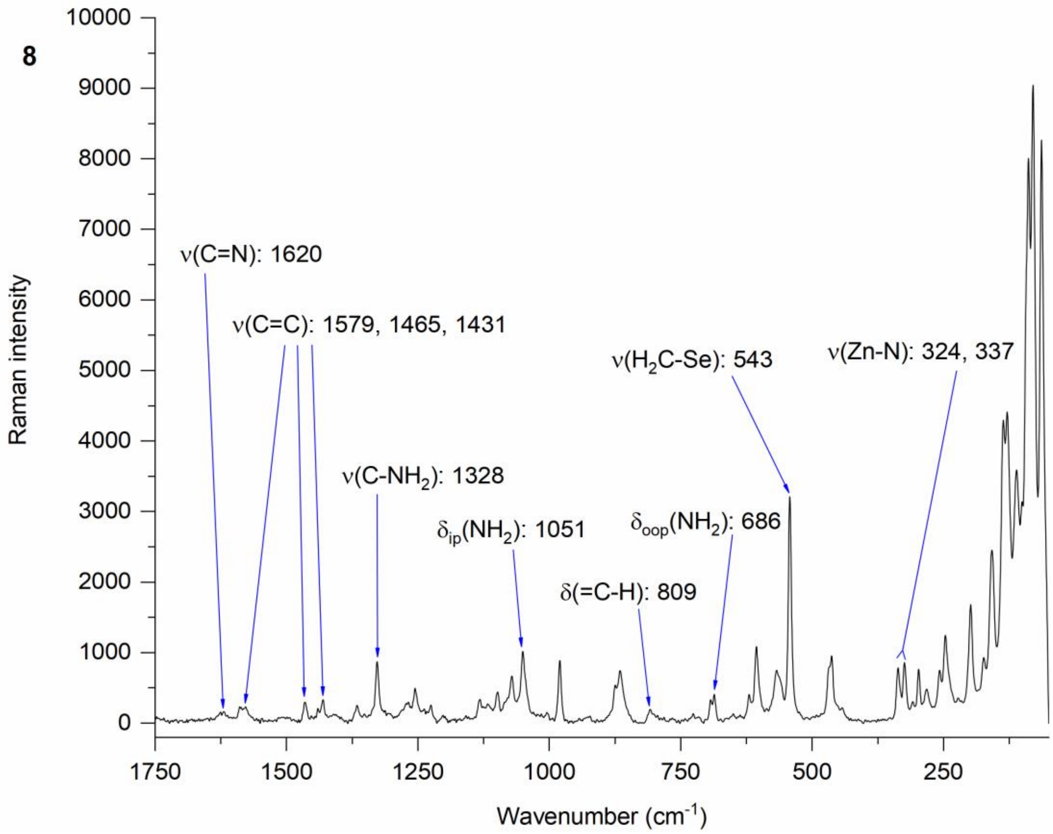


**Figure S40.** Raman spectrum of complex **8**.

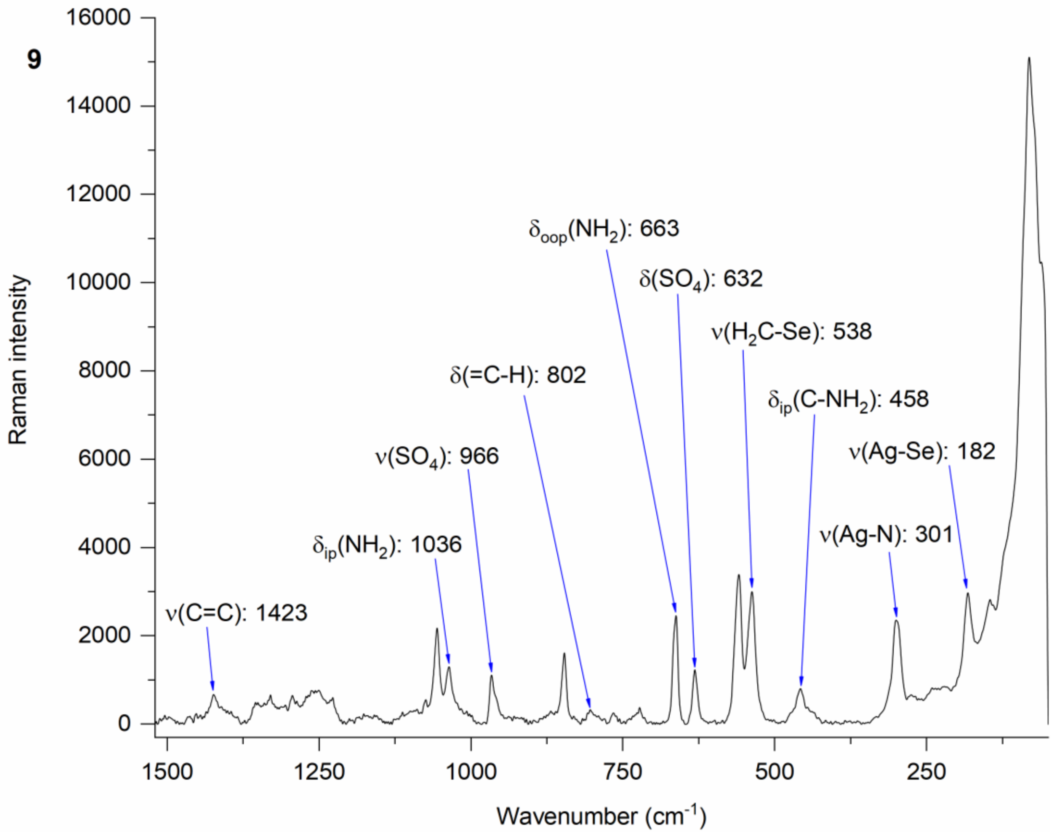


**Figure S41.** Raman spectrum of complex **9**.

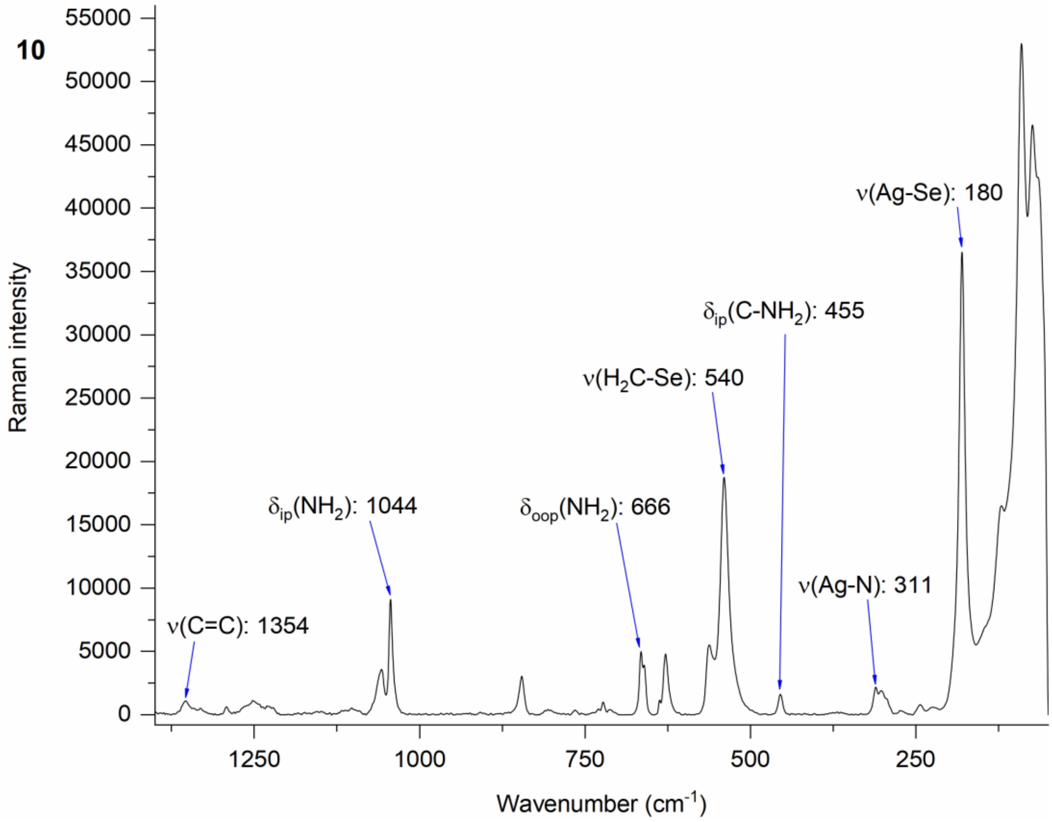


**Figure S42.** Raman spectrum of complex **10**.

## NUCLEAR MAGNETIC RESONANCE (NMR)

### 1D NMR spectra

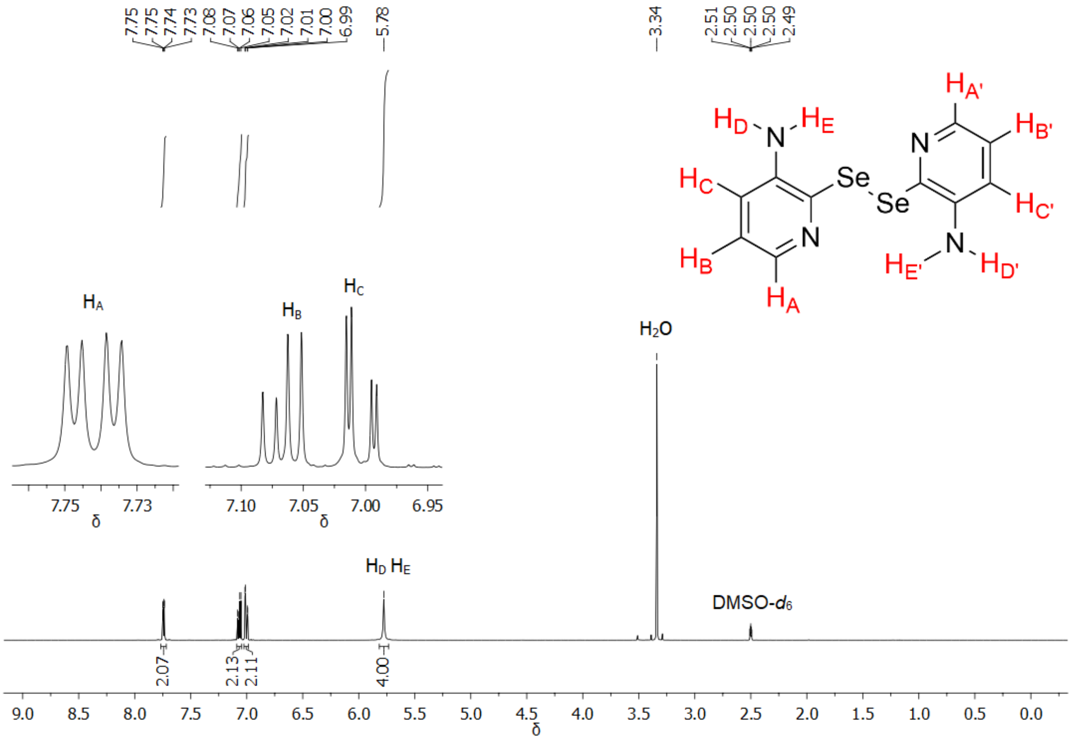


**Figure S43.** $^{1}H$ NMR (400 MHz, DMSO-$d_6$) spectrum of **(3-apySe)$_2$**.

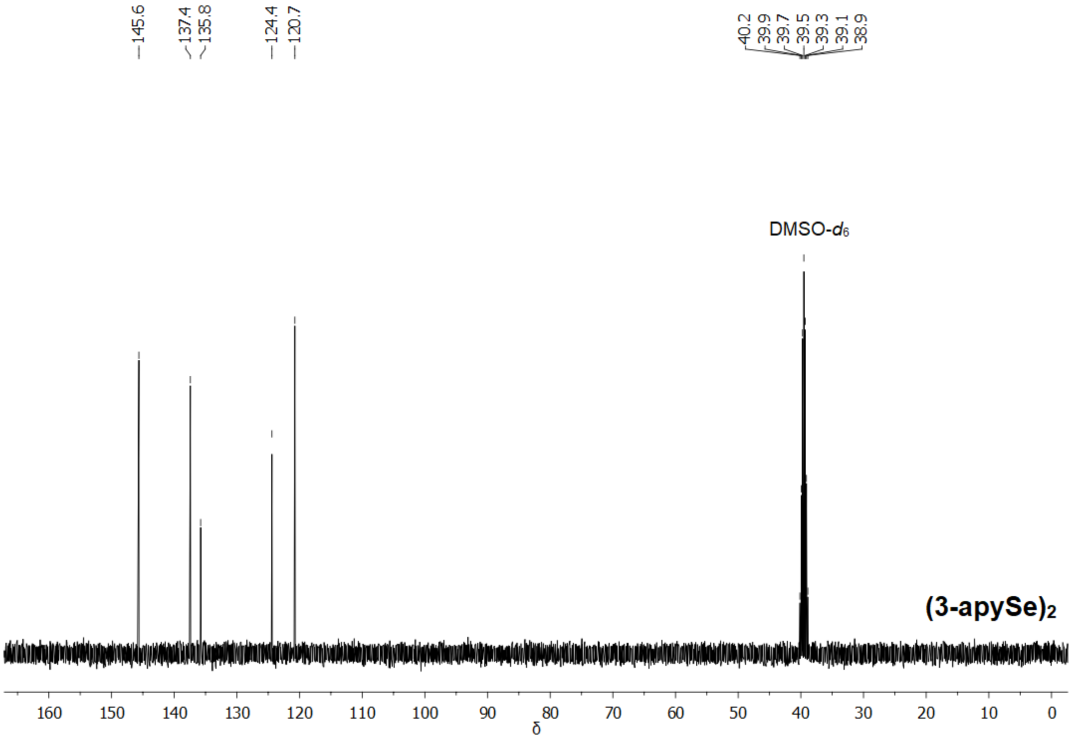


**Figure S44.** $^{13}C$ NMR (100 MHz, DMSO-$d_6$) spectrum of **(3-apySe)$_2$**.

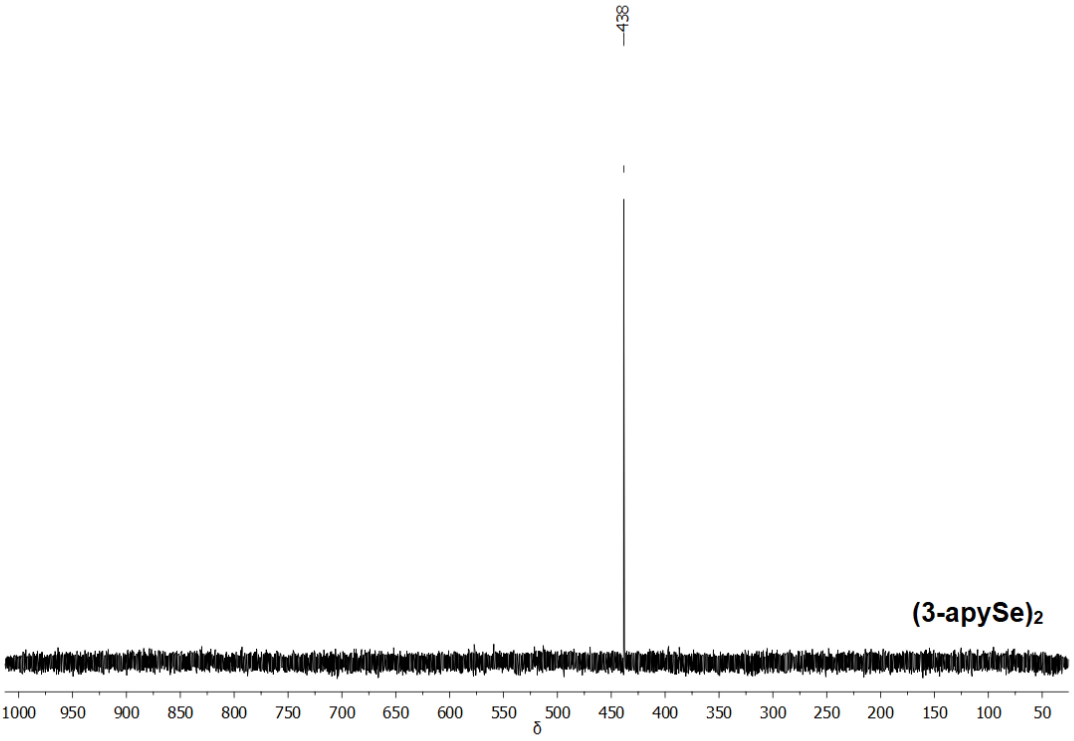


**Figure S45.** $^{77}$Se NMR (76 MHz, DMSO-$d_6$) spectrum of **(3-apySe)$_2$**.

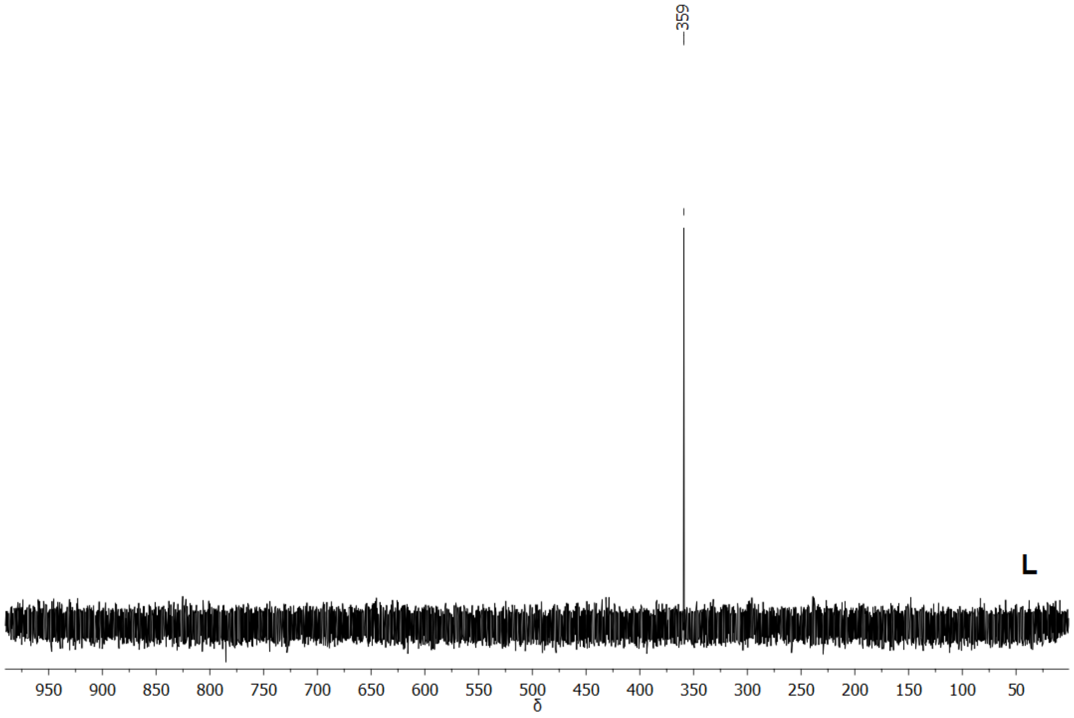


**Figure S46.** $^{77}$Se NMR (76 MHz, DMSO-$d_6$) spectrum of ligand **L**.

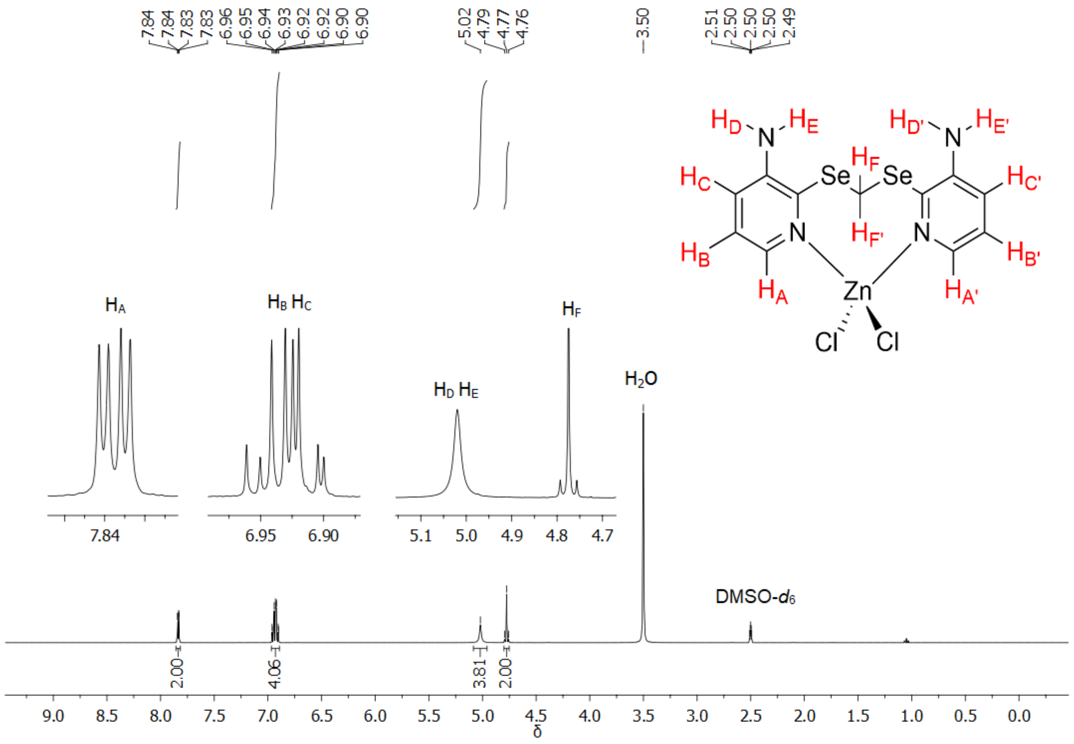

**Figure S47.** $^{1}H$ NMR (400 MHz, DMSO-$d_6$) spectrum of complex **3**.

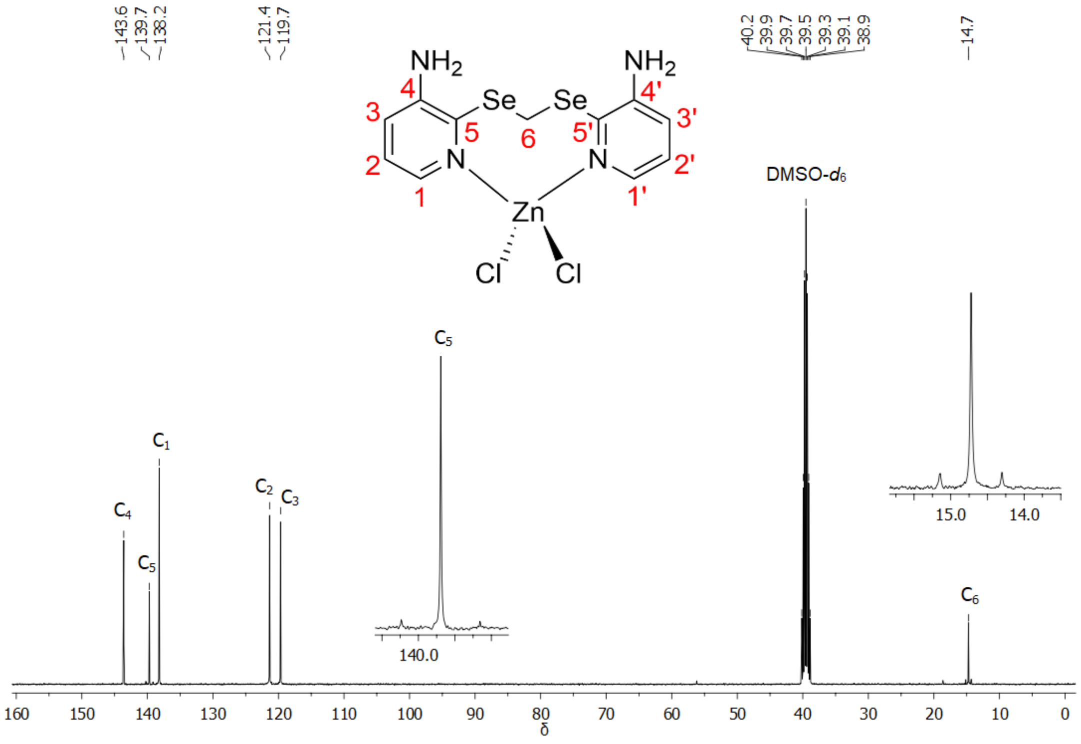

**Figure S48.** $^{13}C$ NMR (100 MHz, DMSO-$d_6$) spectrum of complex **3**.

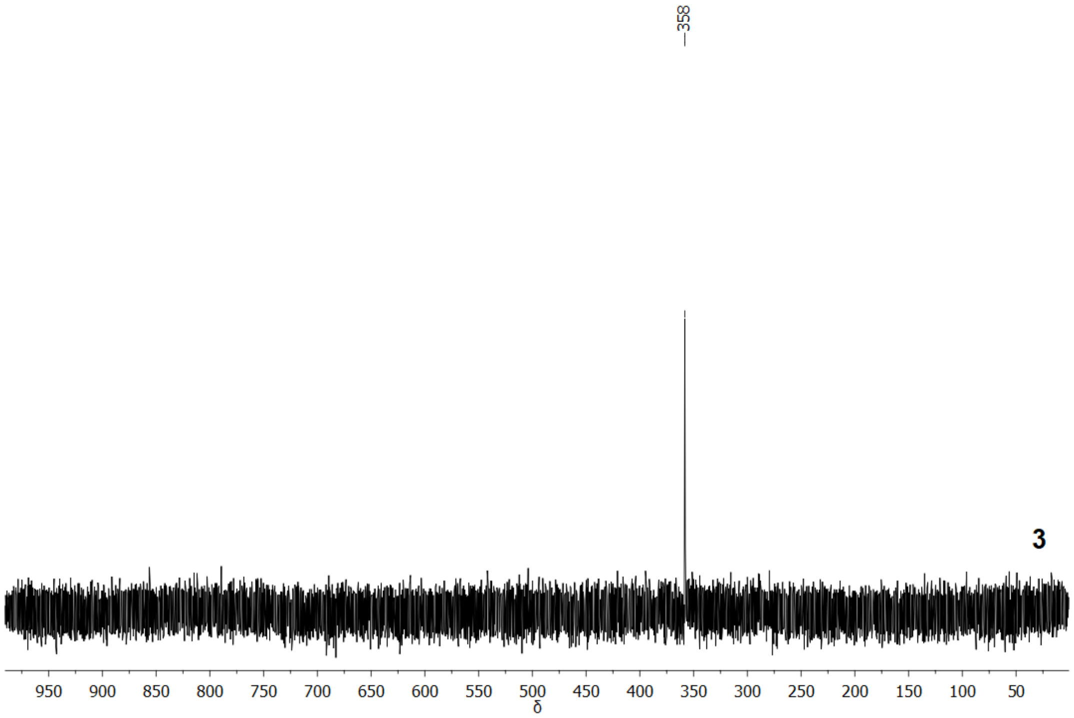


**Figure S49.** $^{77}$Se NMR (76 MHz, DMSO-$d_6$) spectrum of complex **3**.

**2D NMR spectra**

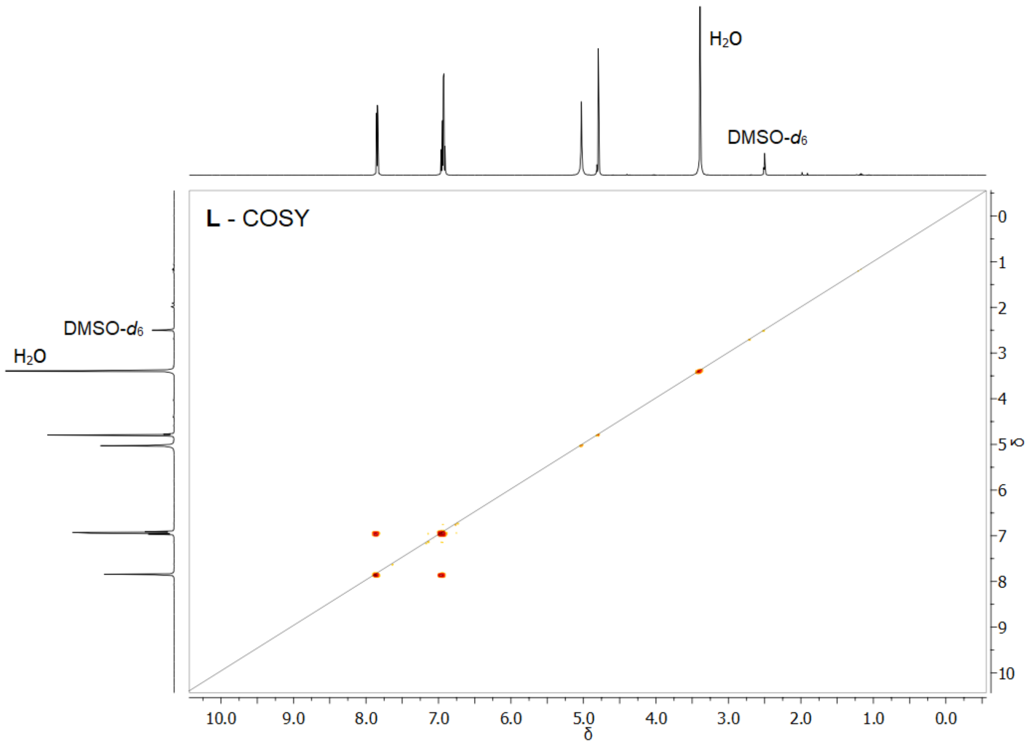


**Figure S50.** COSY NMR spectrum of ligand **L**.

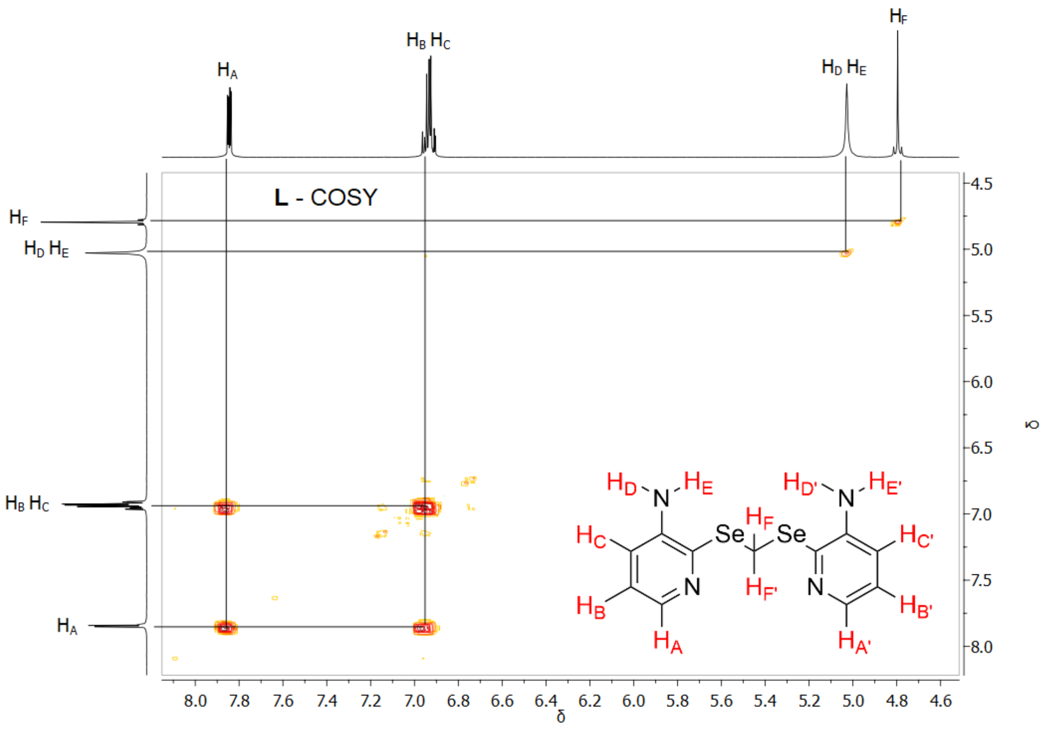


**Figure S51.** COSY NMR spectrum of ligand **L** (expansion).

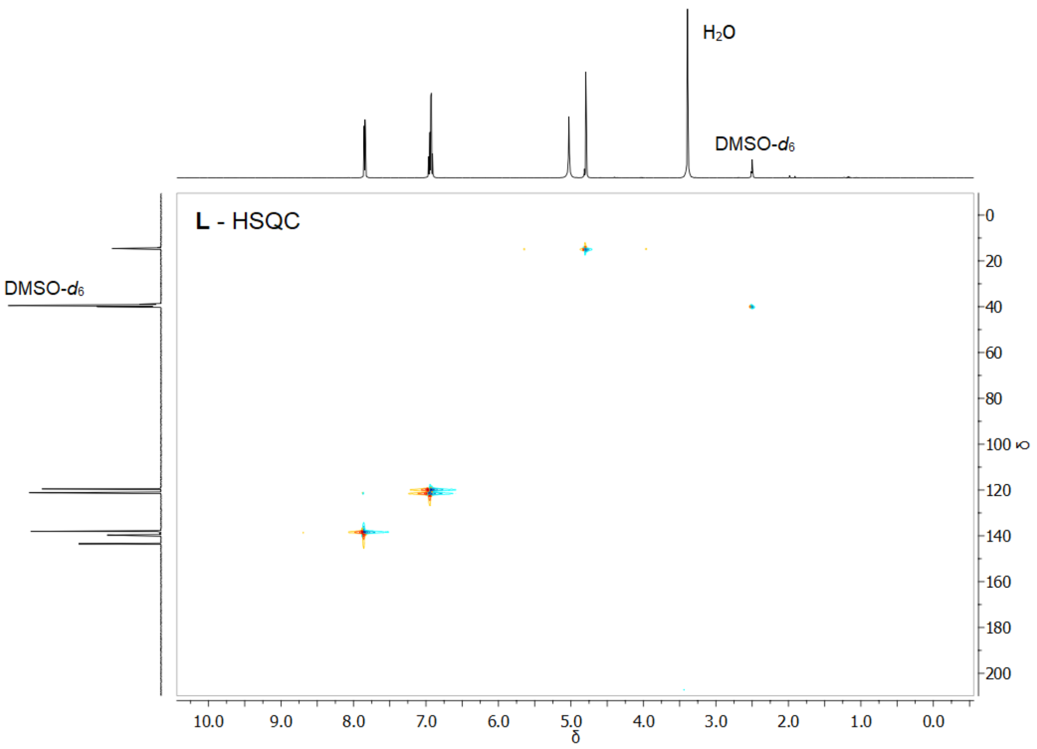


**Figure S52.** HSQC NMR spectrum of ligand **L**.

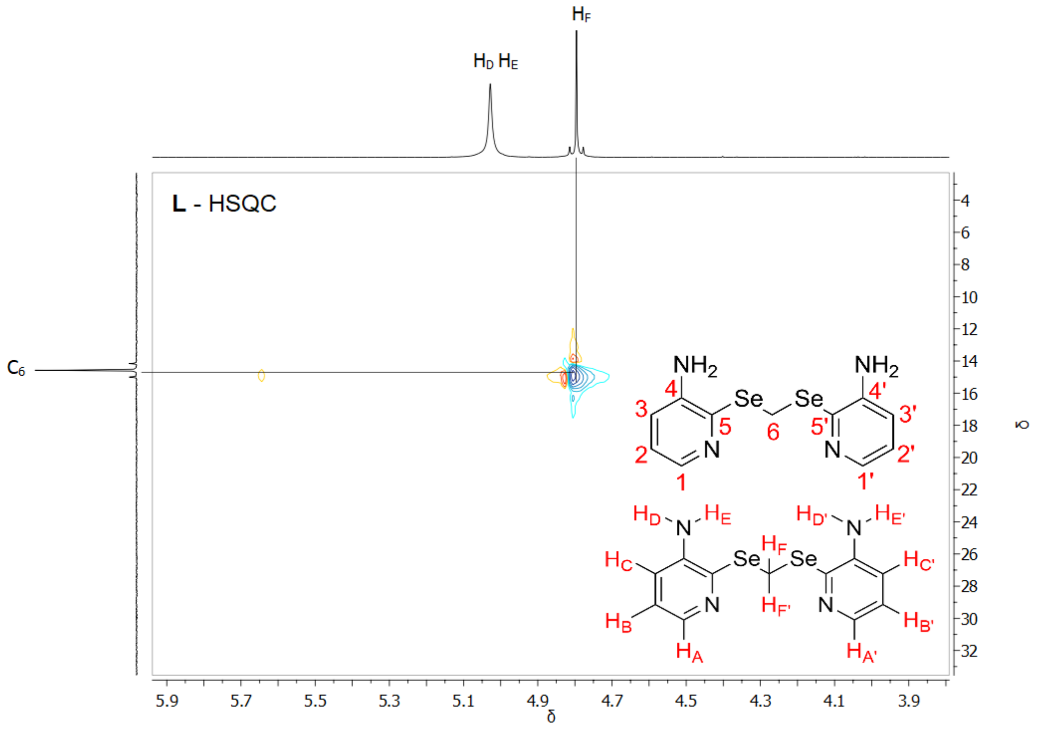


**Figure S53.** HSQC NMR spectrum of ligand **L** (first expansion).

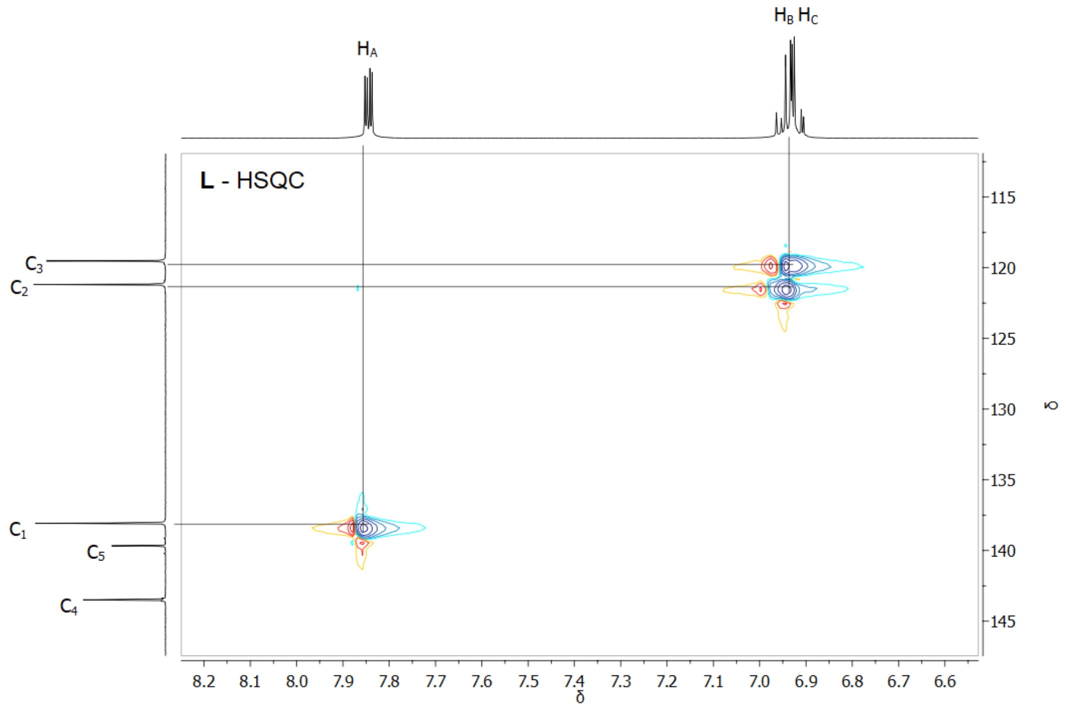


**Figure S54.** HSQC NMR spectrum of ligand **L** (second expansion).

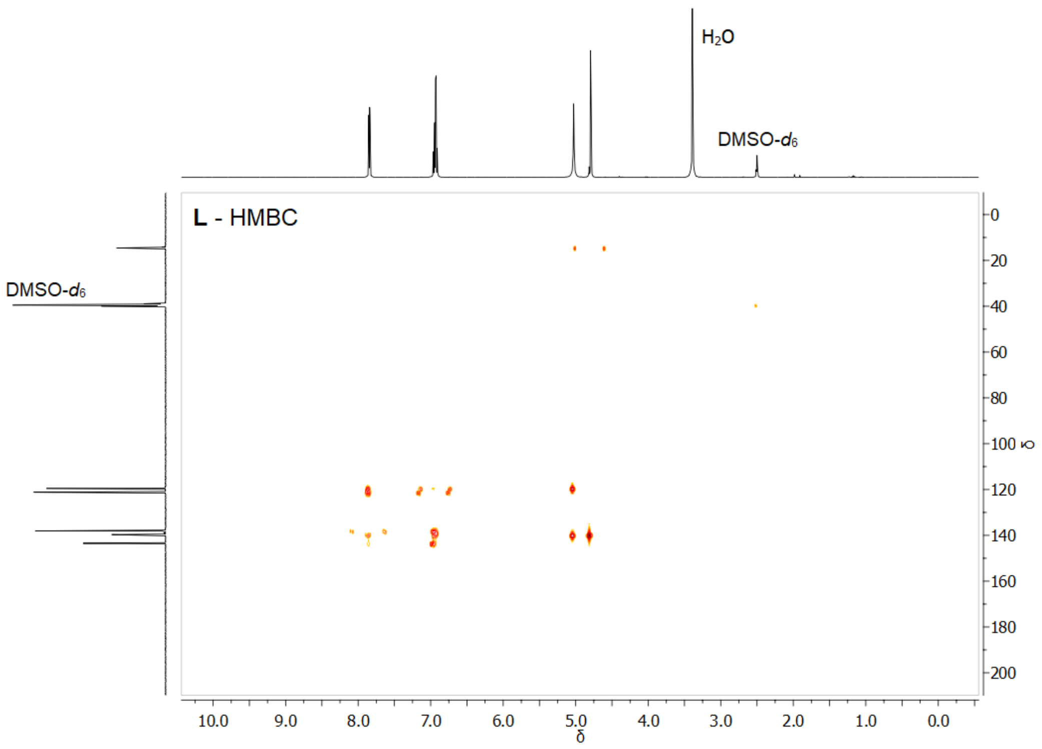


**Figure S55.** HMBC NMR spectrum of ligand **L**.

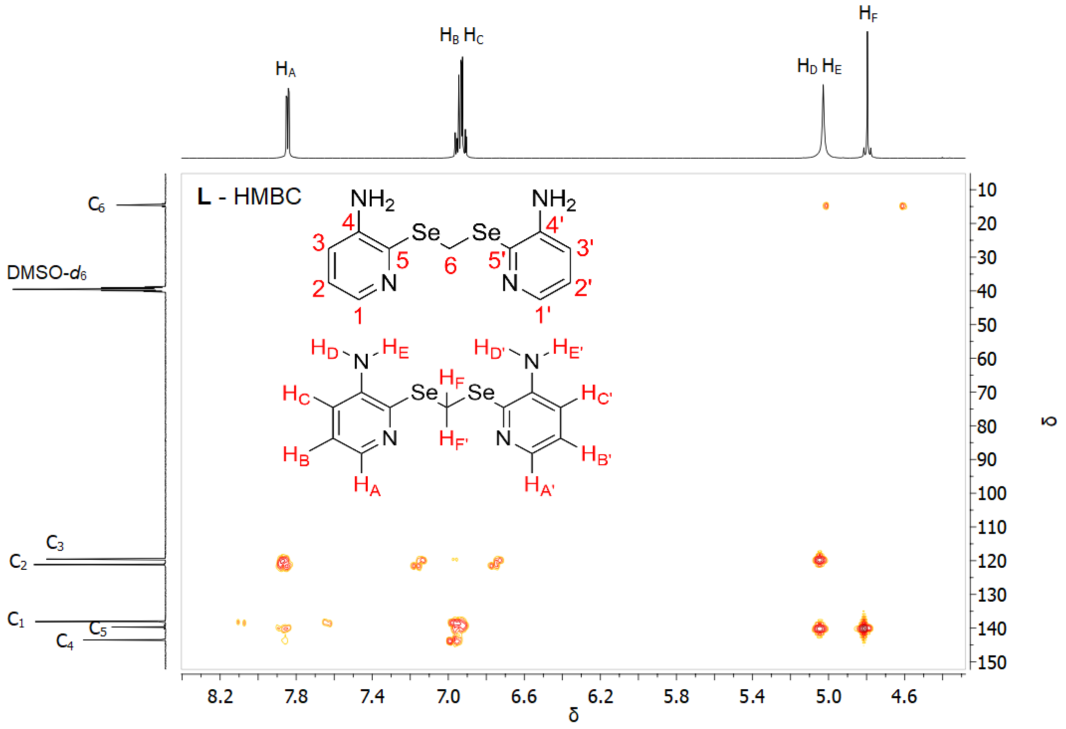


**Figure S56.** HMBC NMR spectrum of ligand **L** (first expansion).

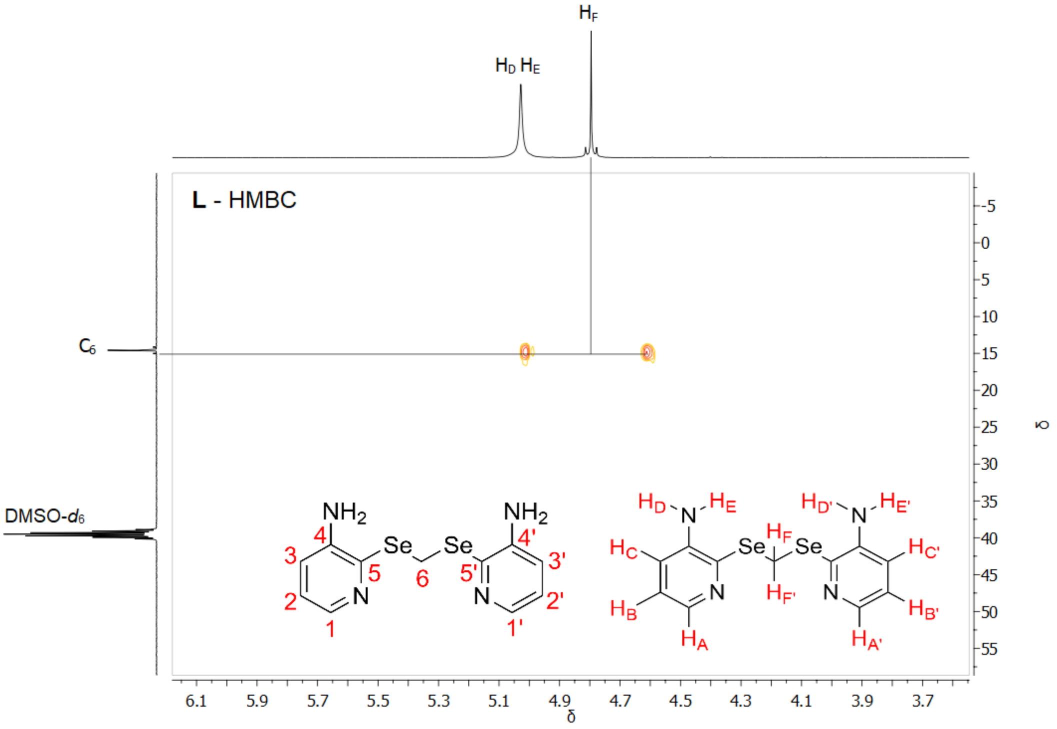


**Figure S57.** HMBC NMR spectrum of ligand **L** (second expansion).

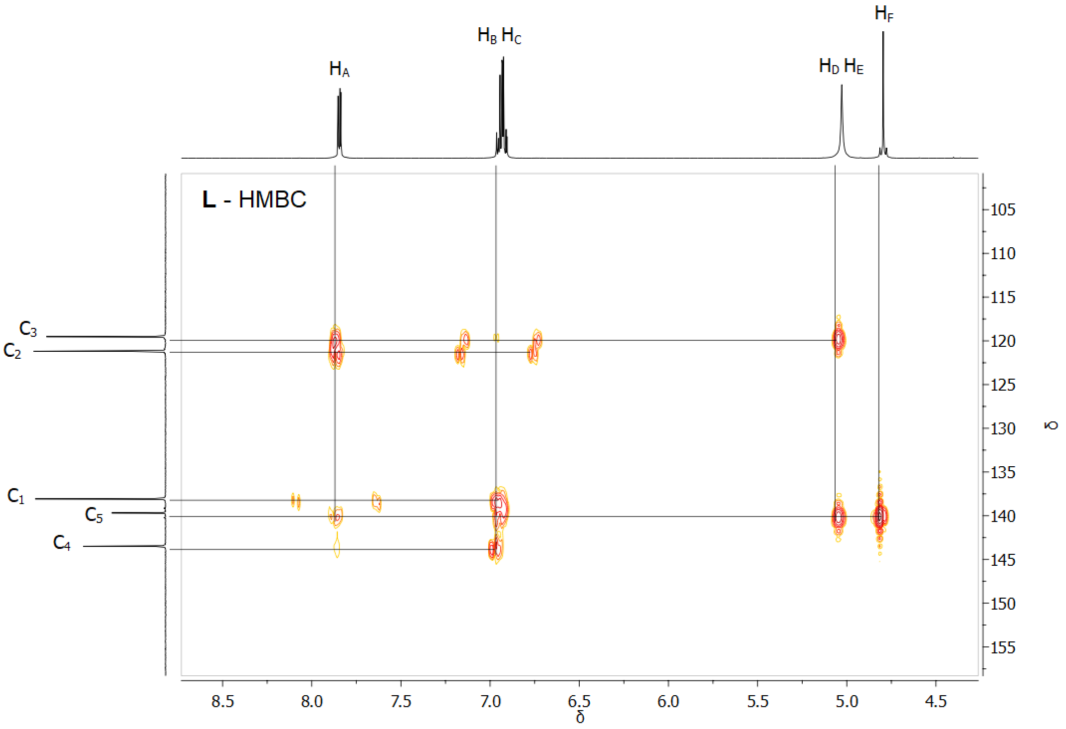


**Figure S58.** HMBC NMR spectrum of ligand **L** (third expansion).

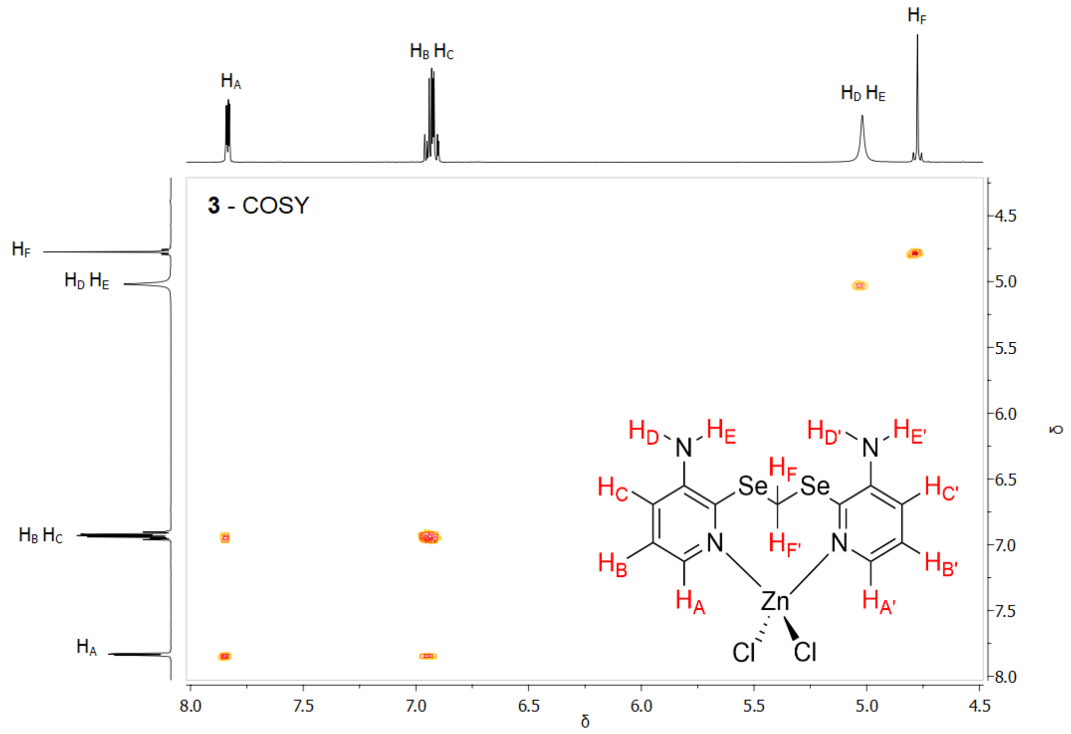


**Figure S59.** COSY NMR spectrum of complex **3**.

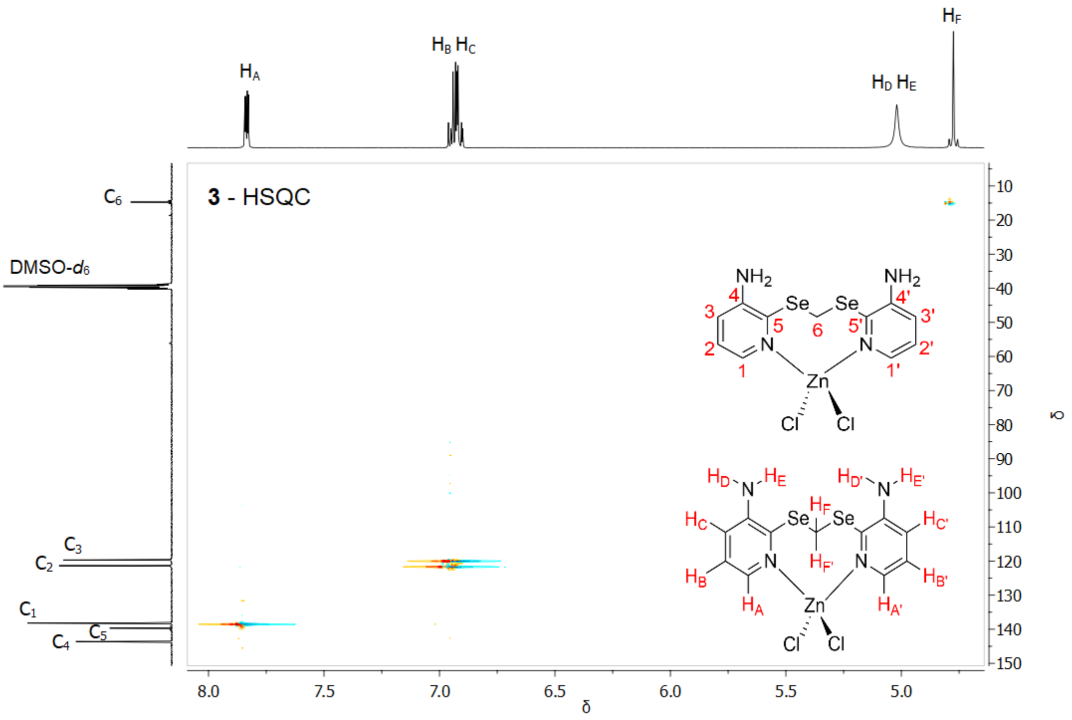


**Figure S60.** HSQC NMR spectrum of complex **3**.

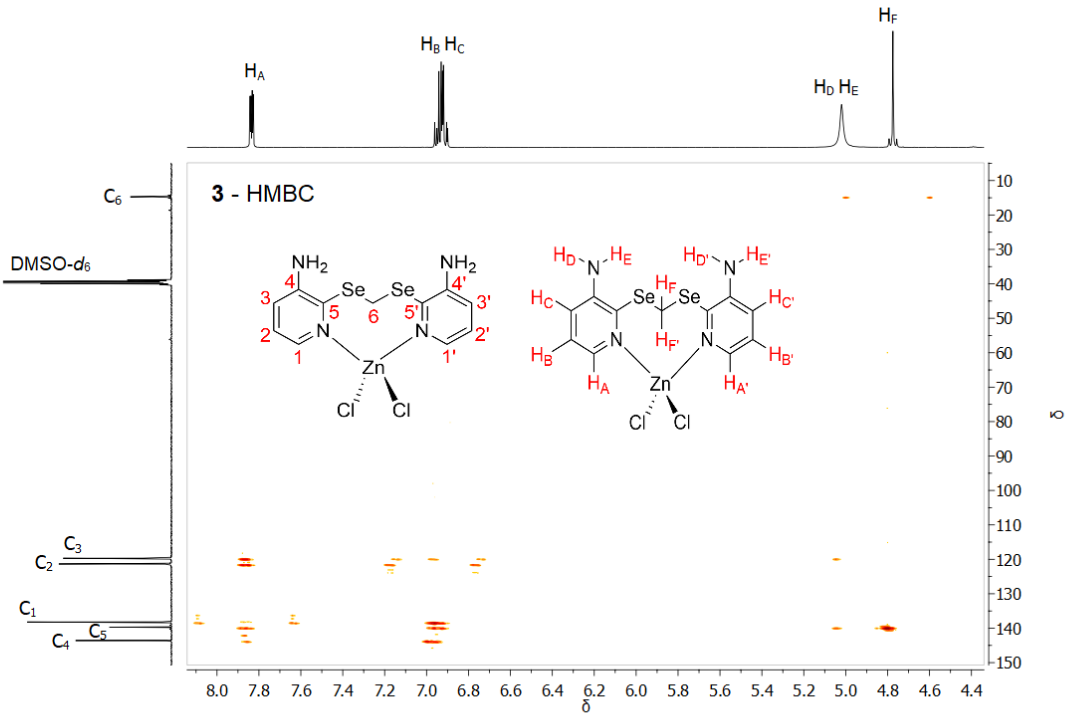


**Figure S61.** HMBC NMR spectrum of complex **3**.

## HIGH-RESOLUTION MASS SPECTROMETRY (HRMS)

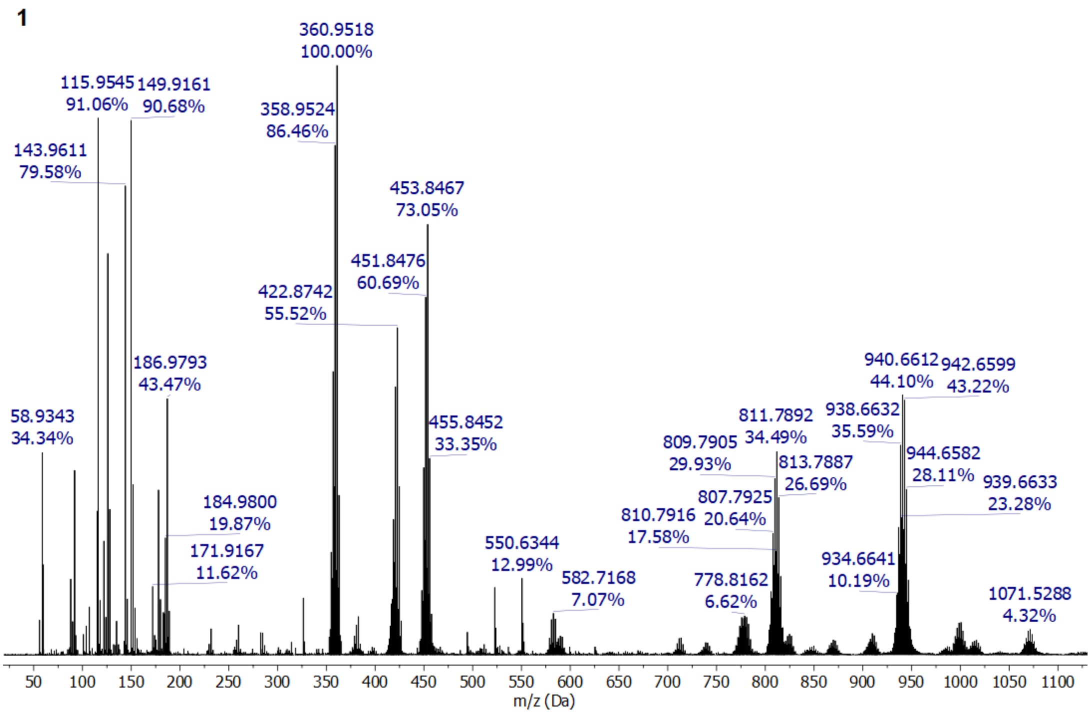


**Figure S62.** HRMS spectrum of complex **1**.

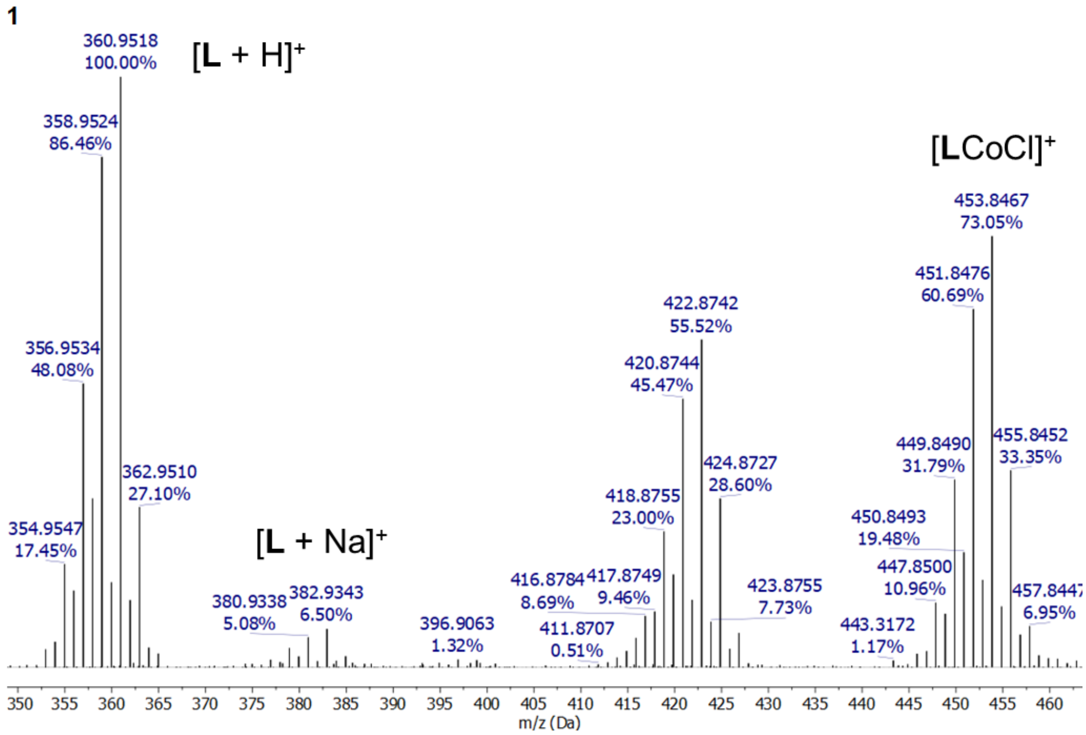


**Figure S63.** HRMS spectrum of complex **1** (expansion).

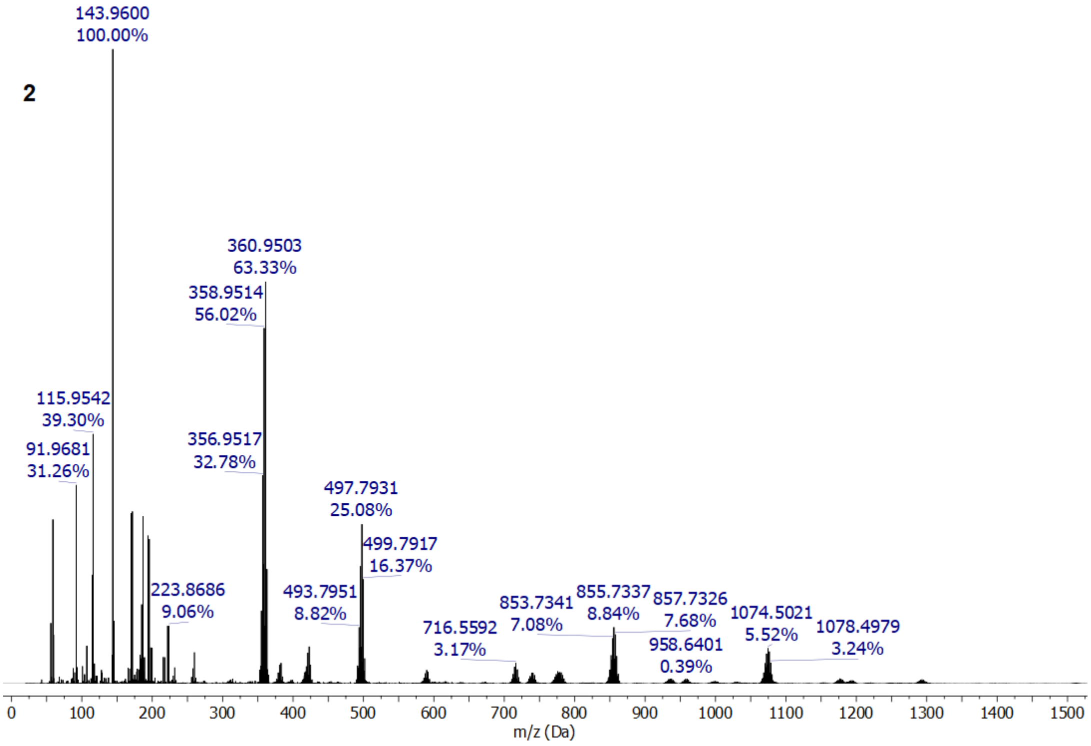


**Figure S64.** HRMS spectrum of complex **2**.

**2**

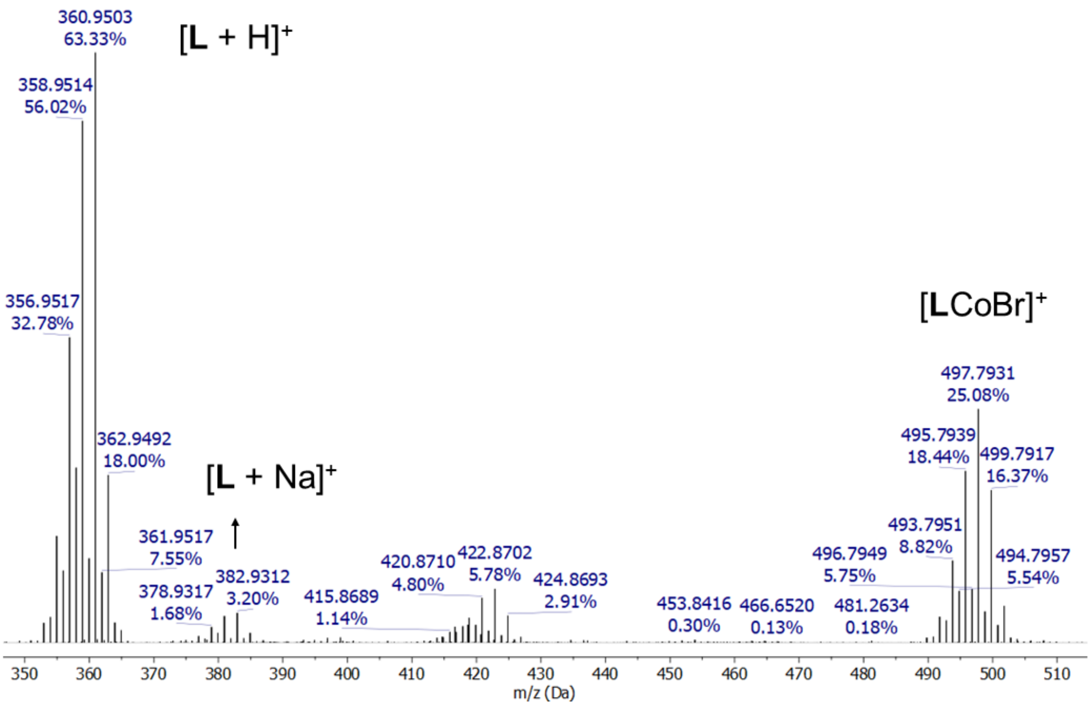


**Figure S65.** HRMS spectrum of complex **2** (expansion).

**3**

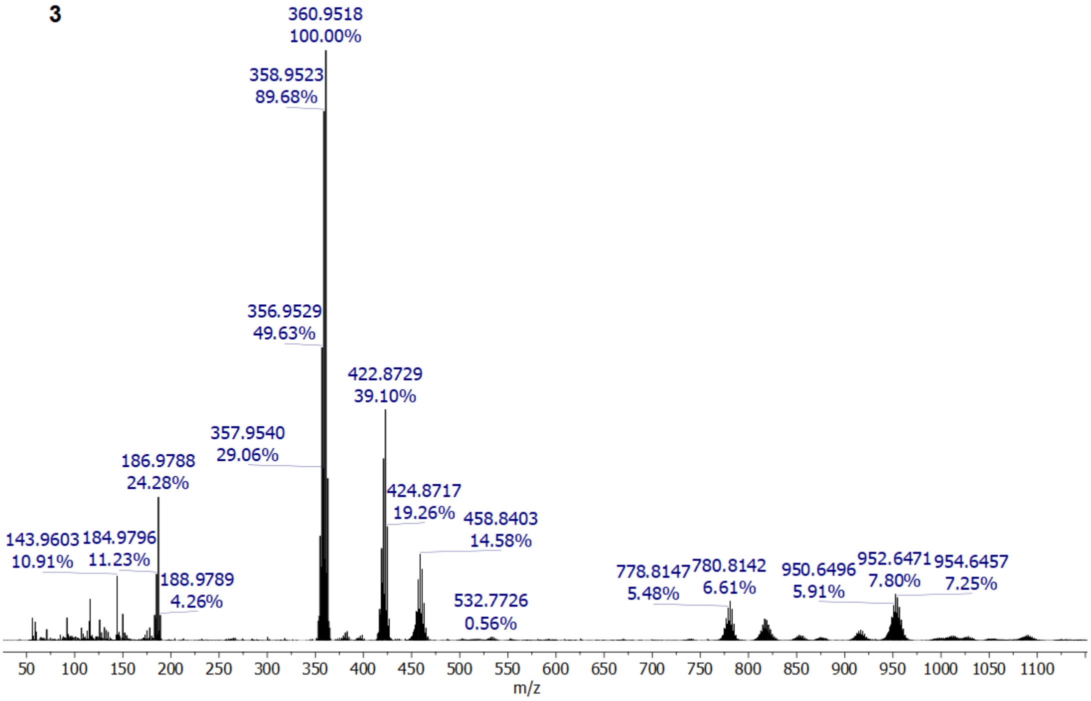


**Figure S66.** HRMS spectrum of complex **3**.

3

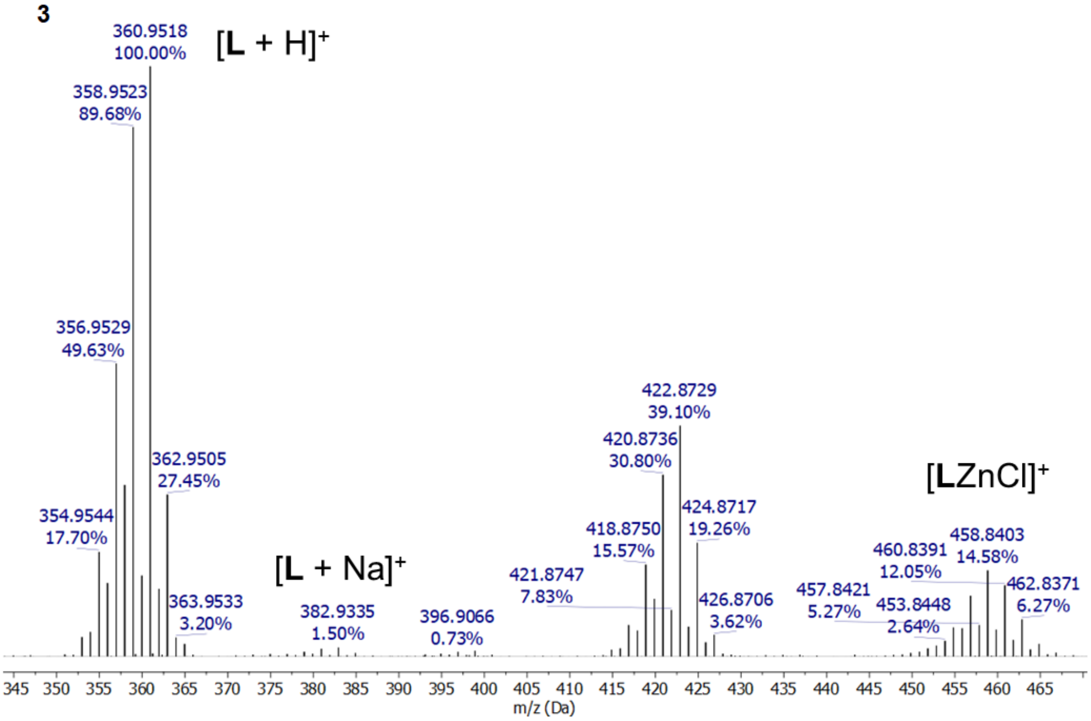


**Figure S67.** HRMS spectrum of complex **3** (expansion).

4

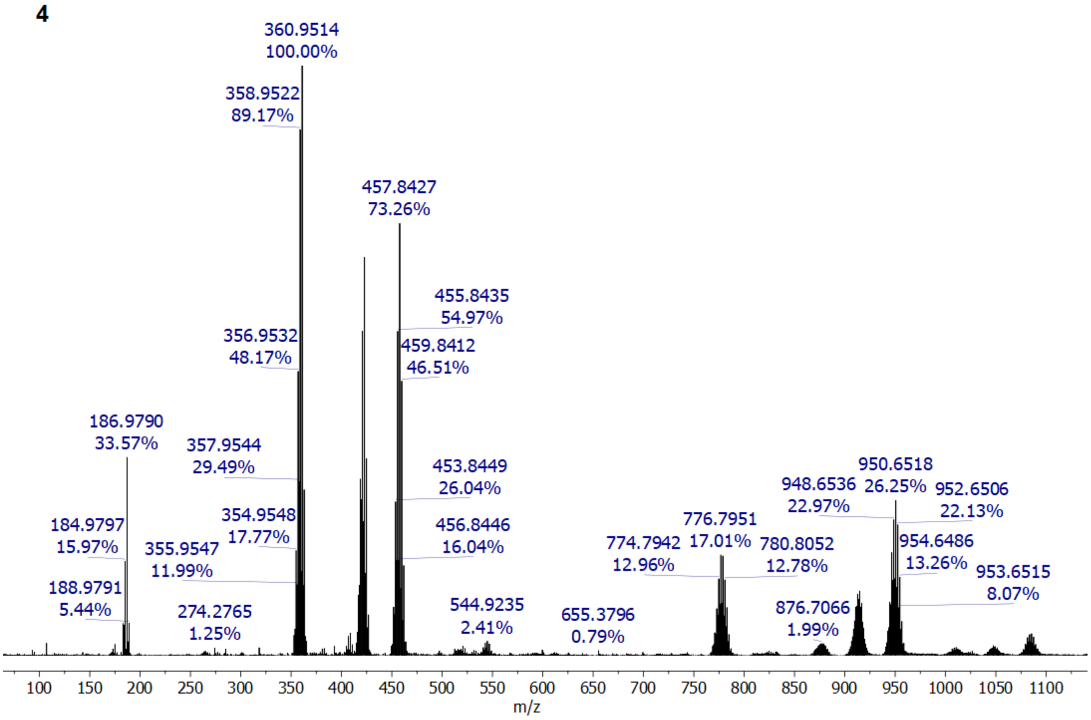


**Figure S68.** HRMS spectrum of complex **4**.

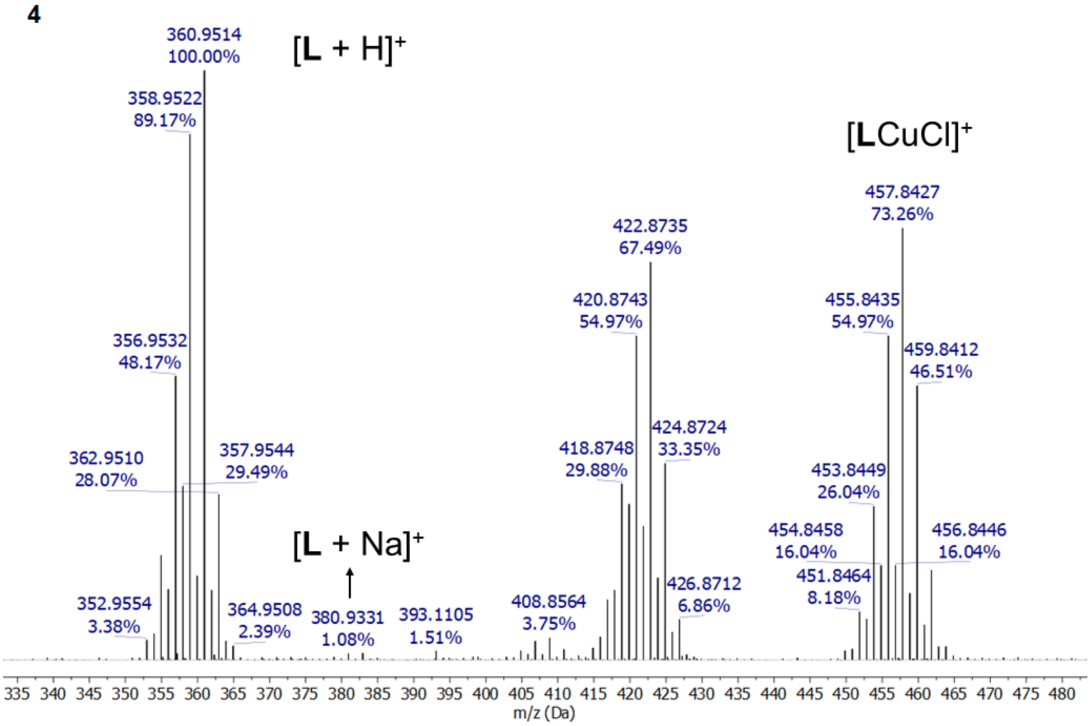


**Figure S69.** HRMS spectrum of complex **4** (expansion).

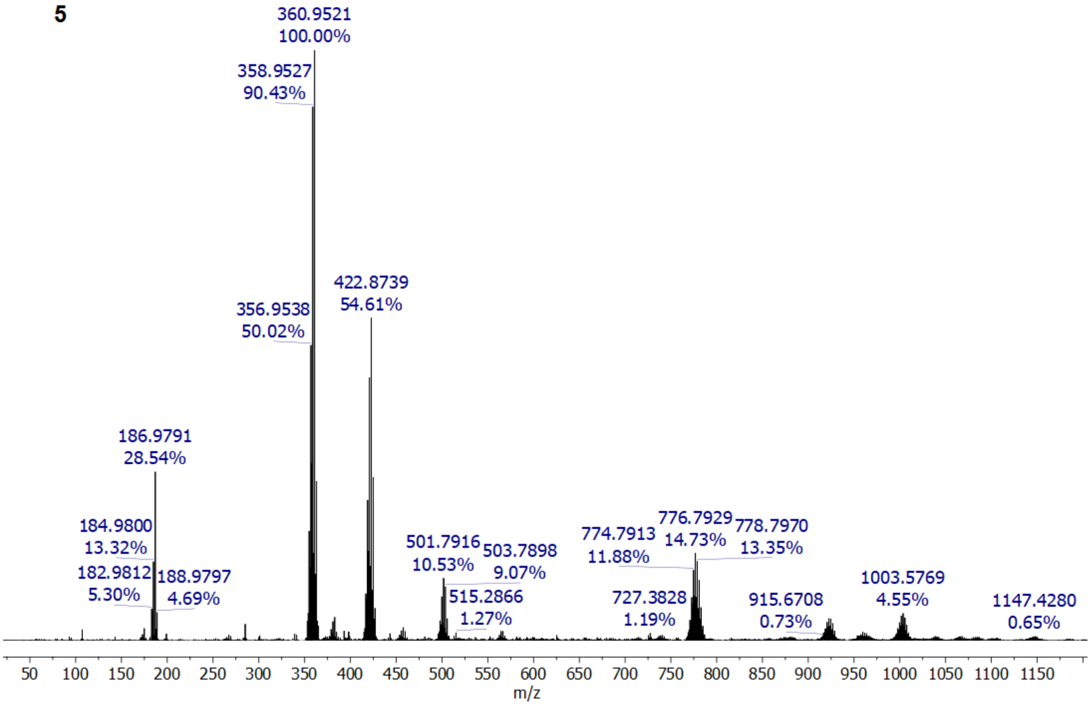


**Figure S70.** HRMS spectrum of complex **5**.

**5**

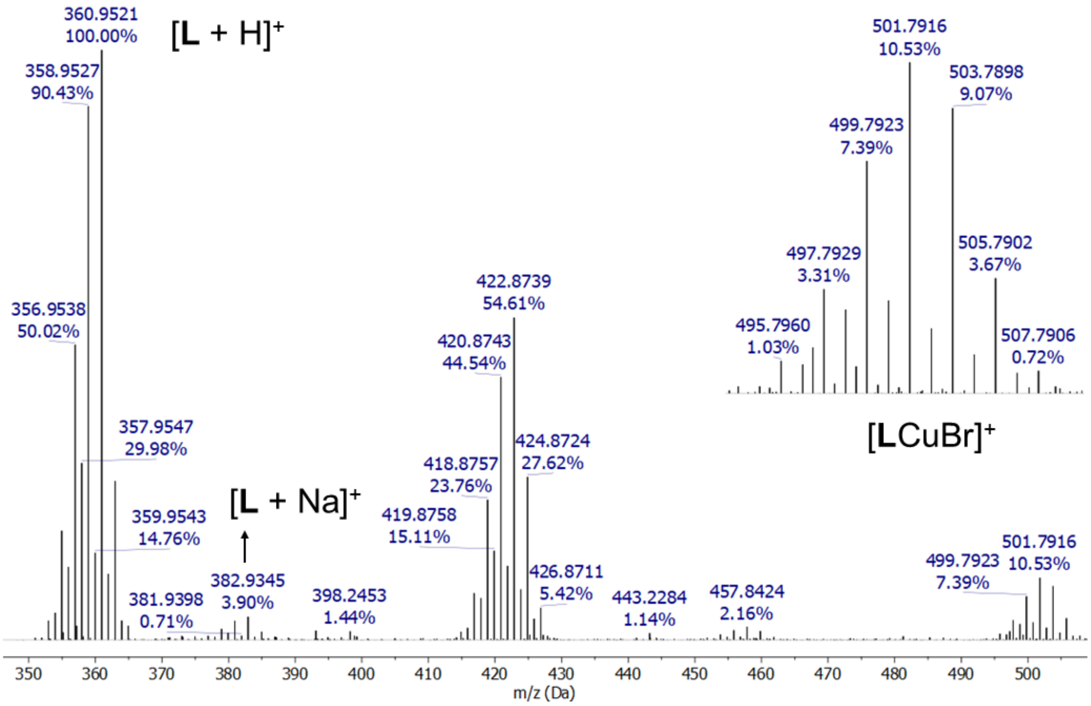


**Figure S71.** HRMS spectrum of complex **5** (expansion).

**6**

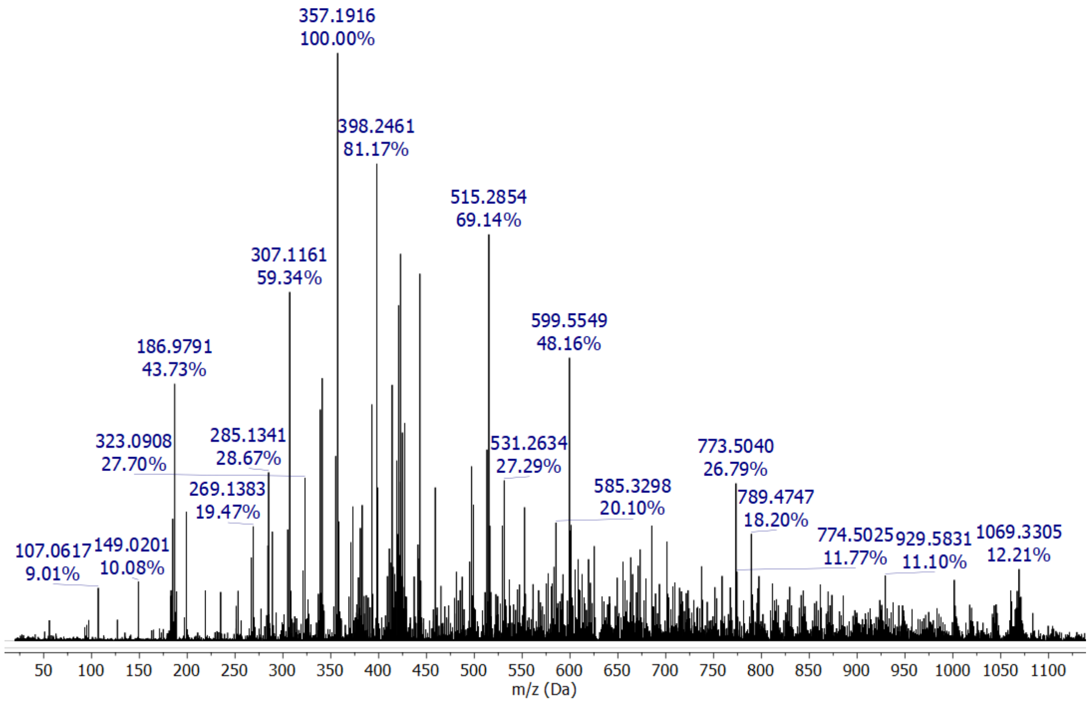


**Figure S72.** HRMS spectrum of complex **6**.

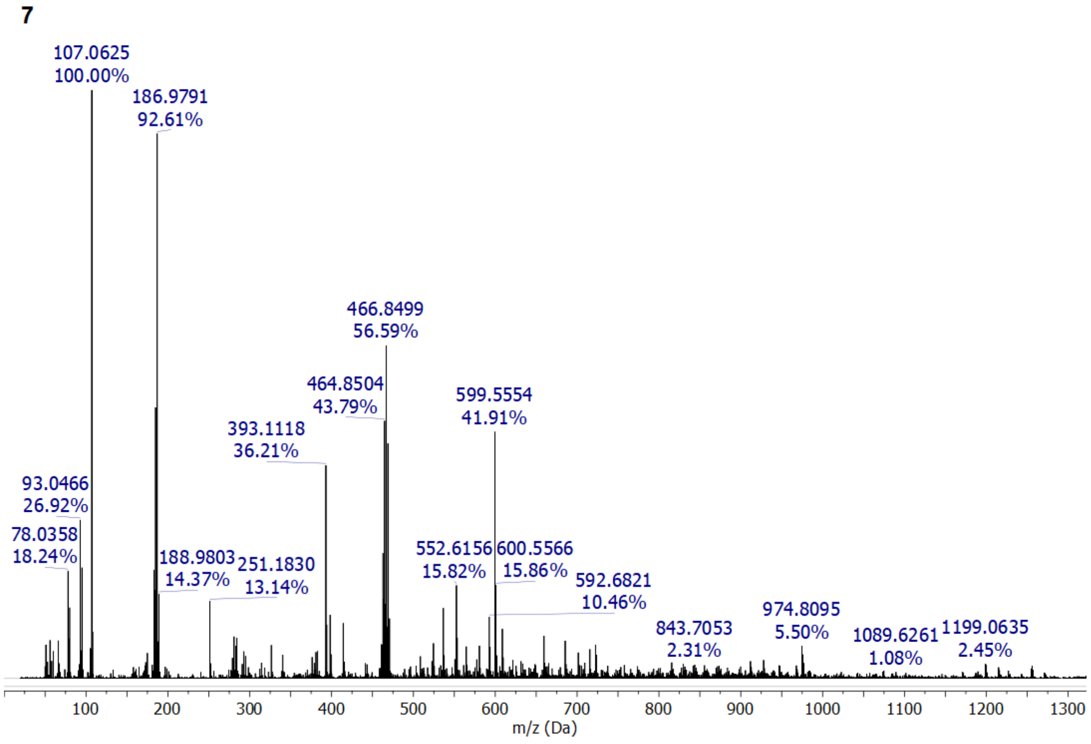


**Figure S73.** HRMS spectrum of complex **7**.

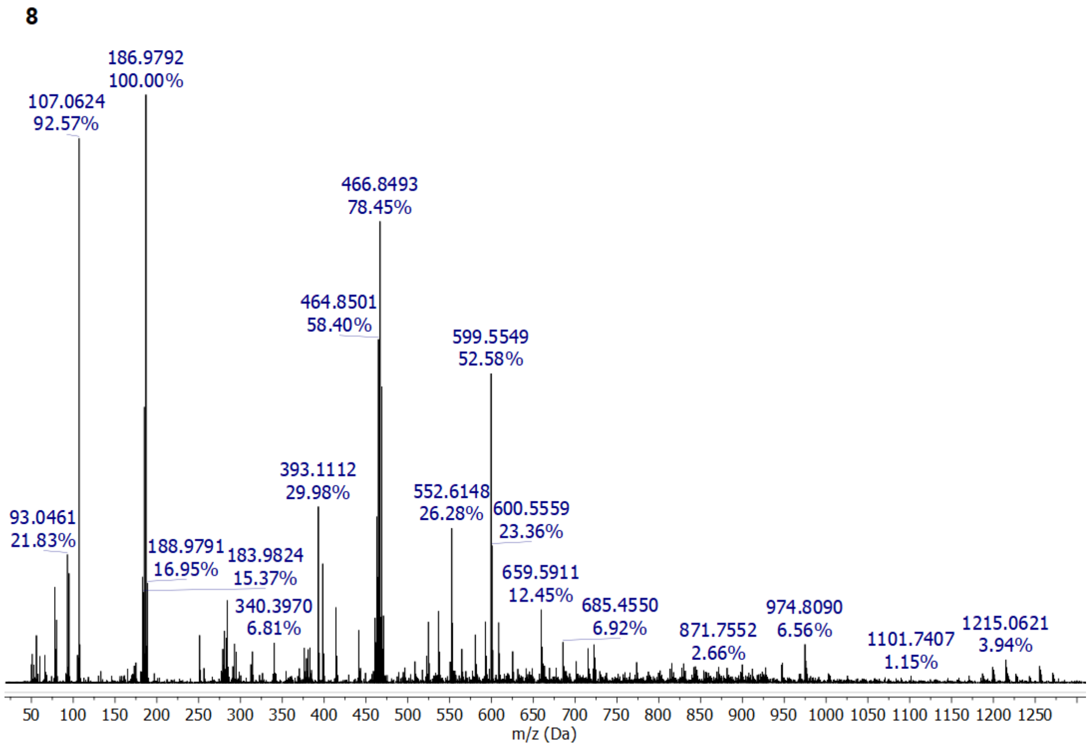


**Figure S74.** HRMS spectrum of complex **8**.

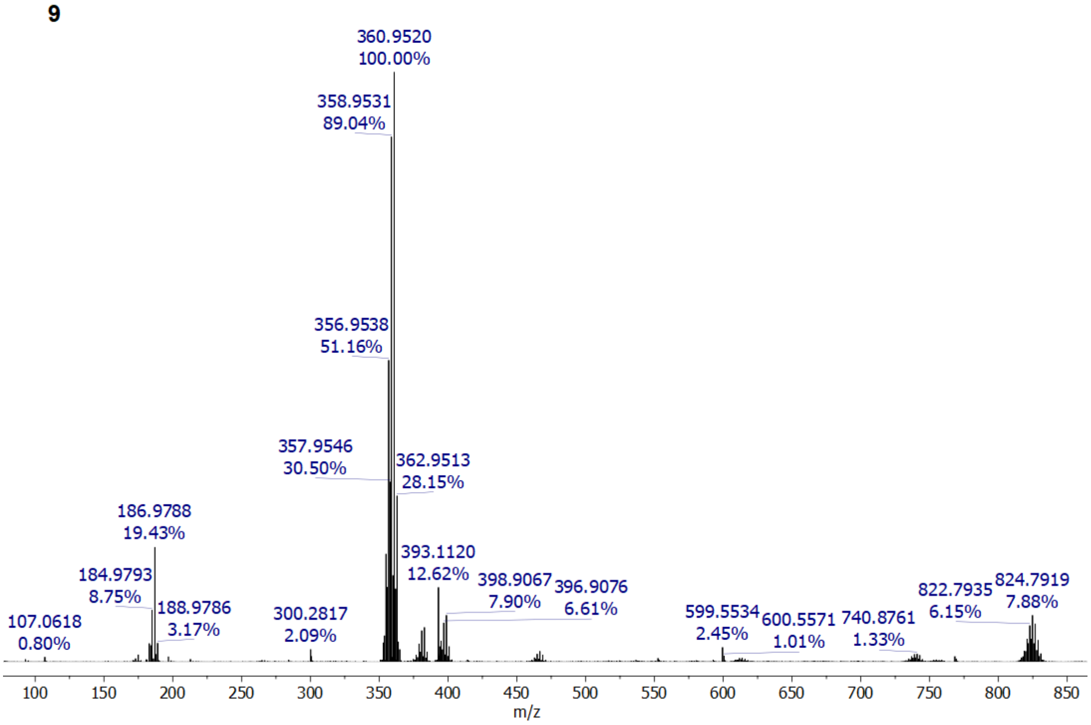


**Figure S75.** HRMS spectrum of complex **9**.

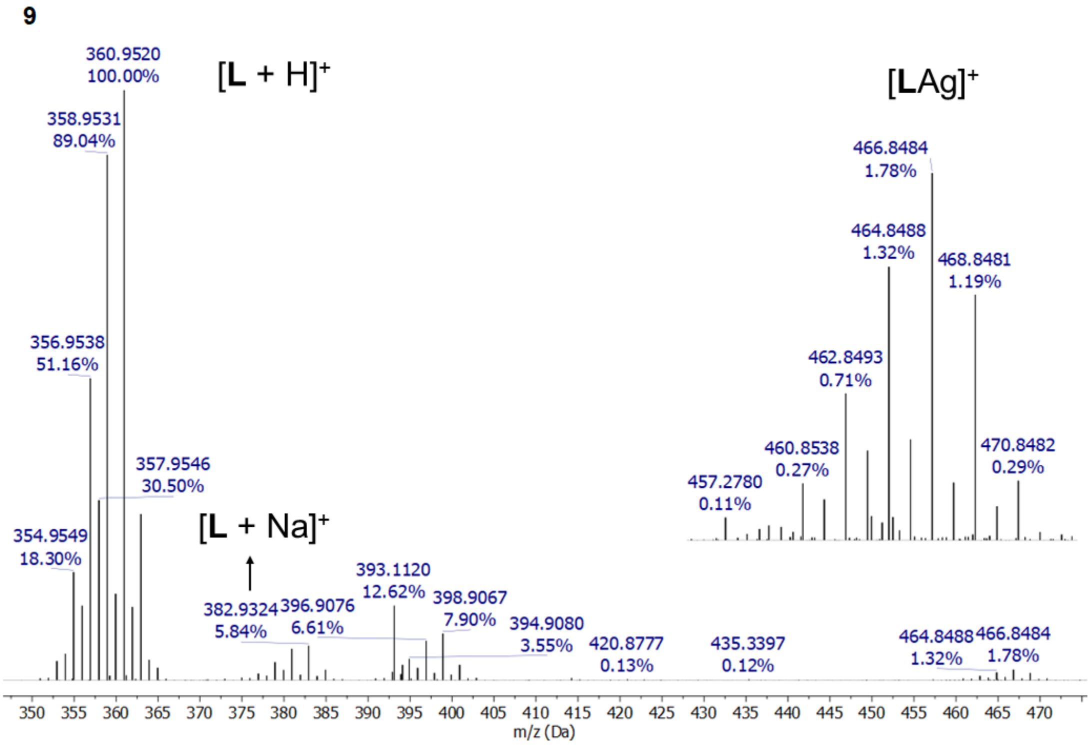


**Figure S76.** HRMS spectrum of complex **9** (first expansion).

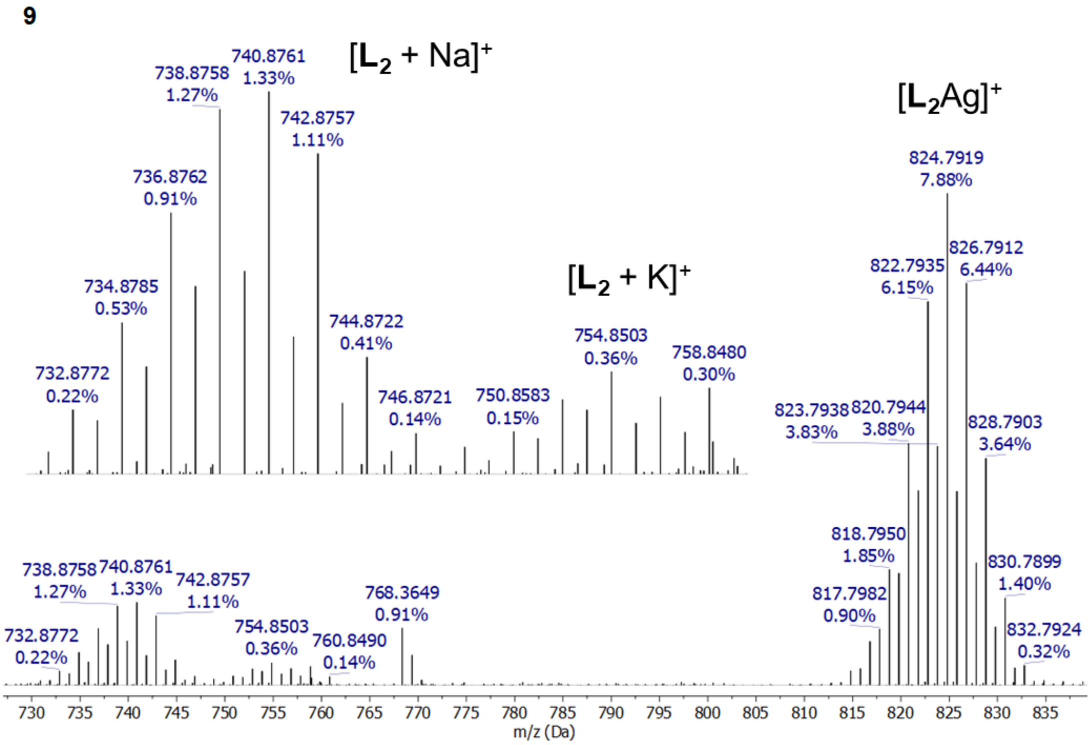


**Figure S77.** HRMS spectrum of complex **9** (second expansion).

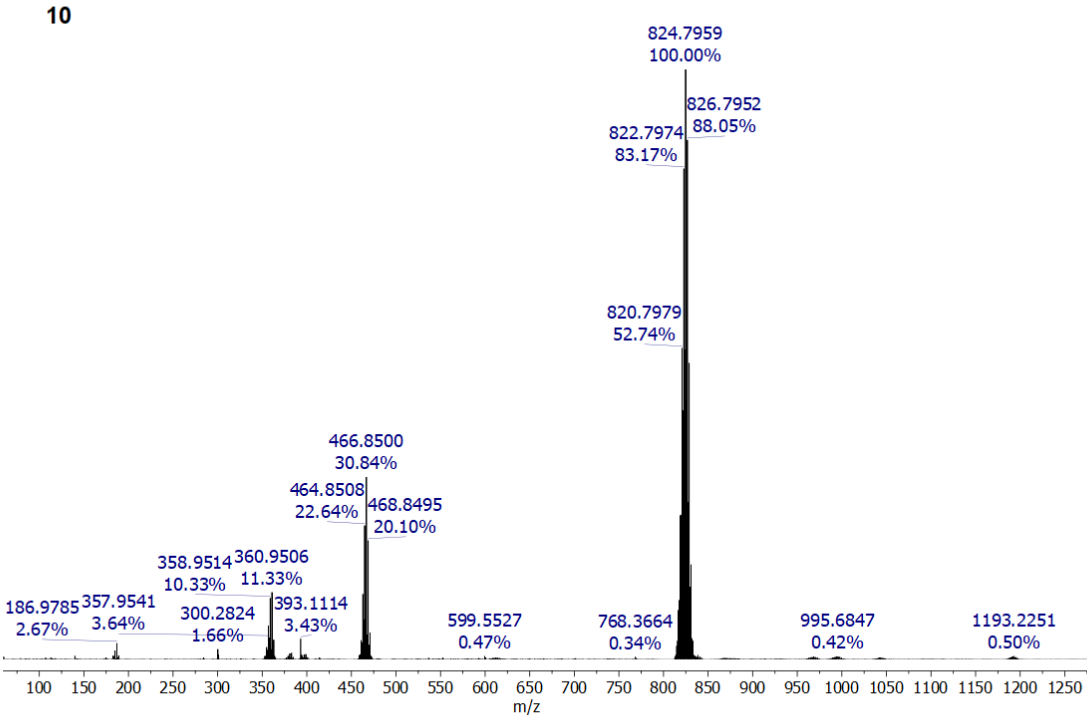


**Figure S78.** HRMS spectrum of complex **10**.

**10**

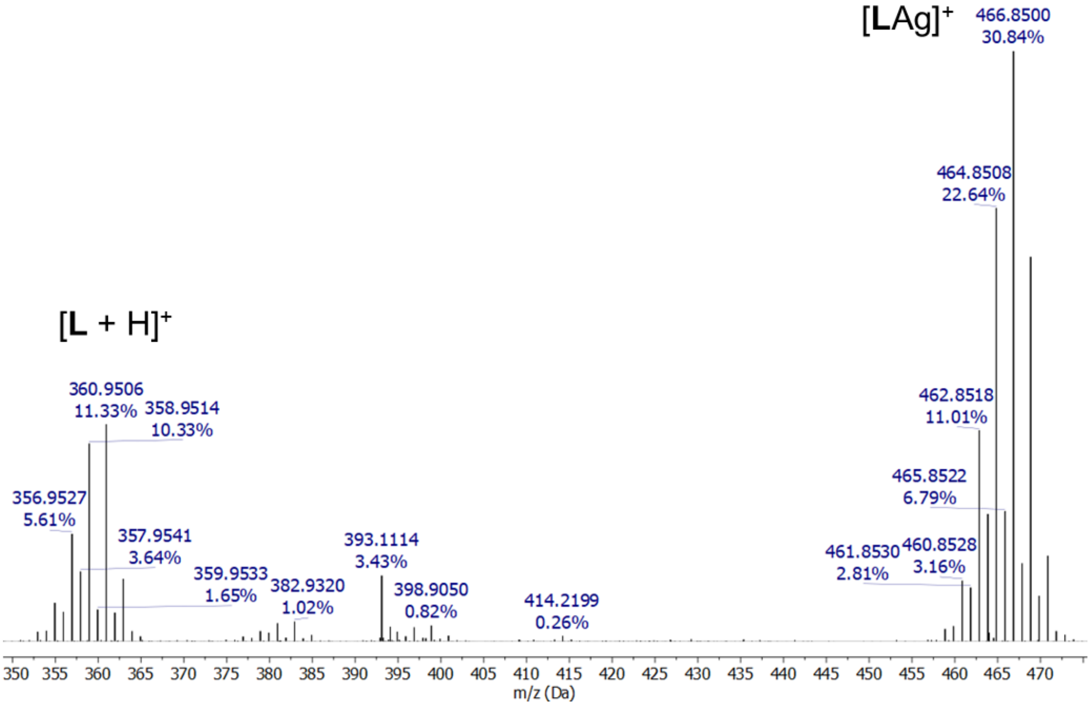


**Figure S79.** HRMS spectrum of complex **10** (first expansion).

**10**

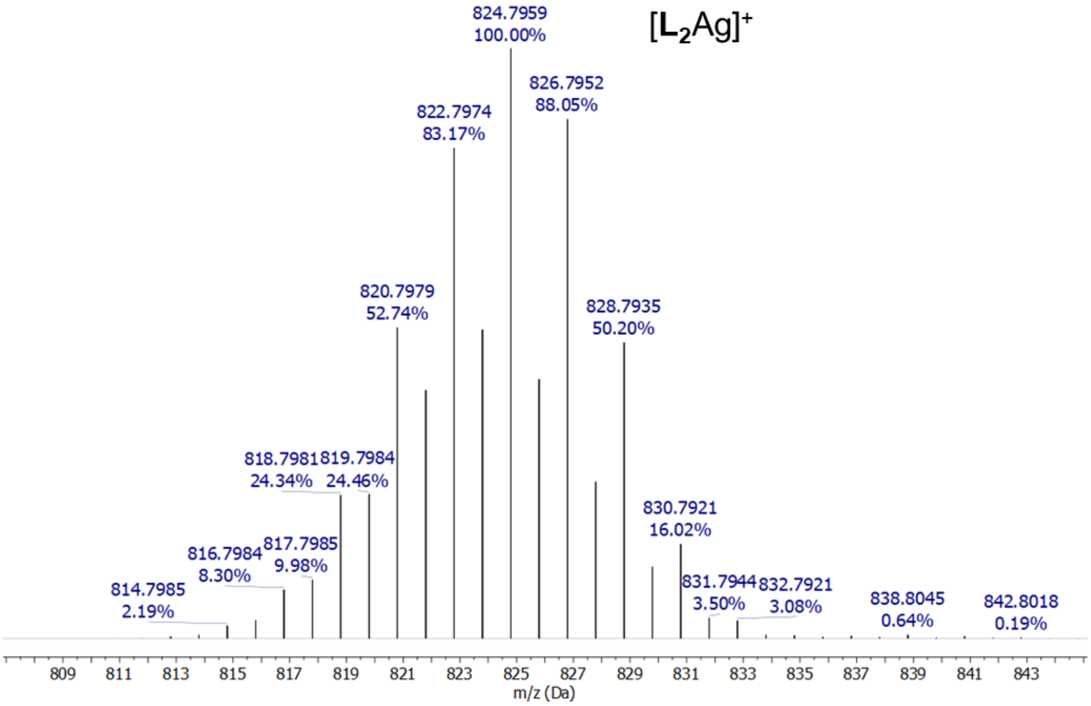


**Figure S80.** HRMS spectrum of complex **10** (second expansion).

## ULTRAVIOLET-VISIBLE SPECTROSCOPY (UV-VIS)

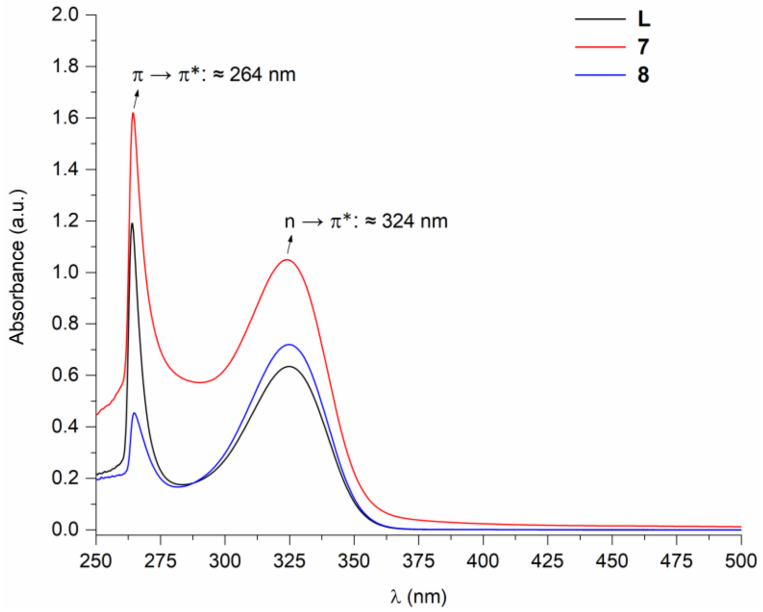


**Figure S81.** UV-Vis spectra of complexes **7** and **8** in DMF.

### Stability in solution over time

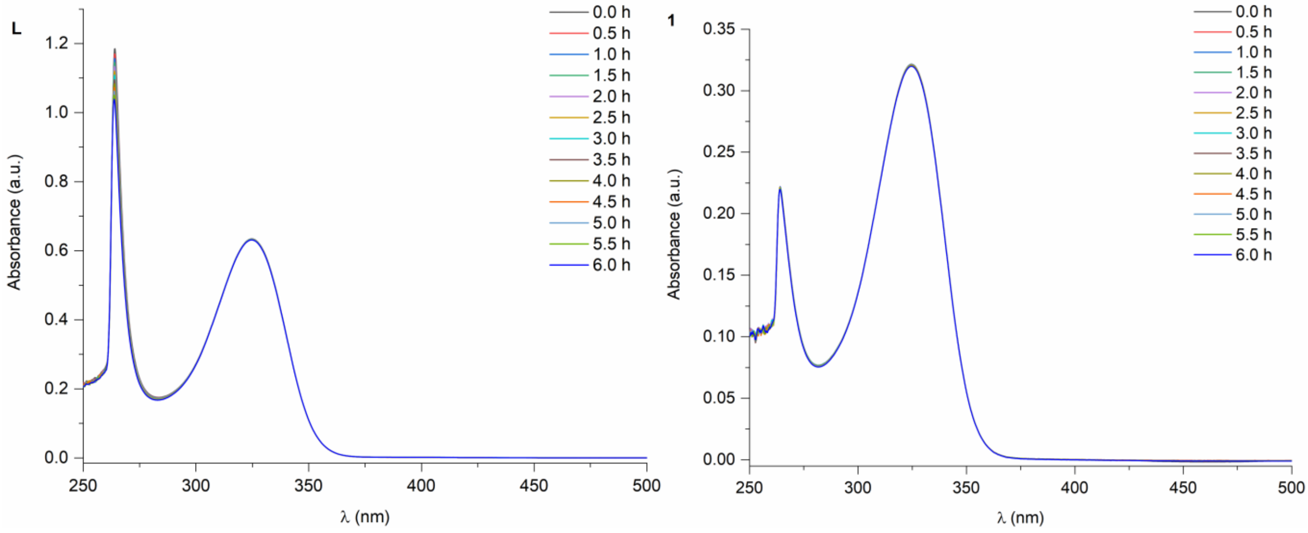


**Figure S82.** Stability in solution over time of ligand **L** and complex **1**.

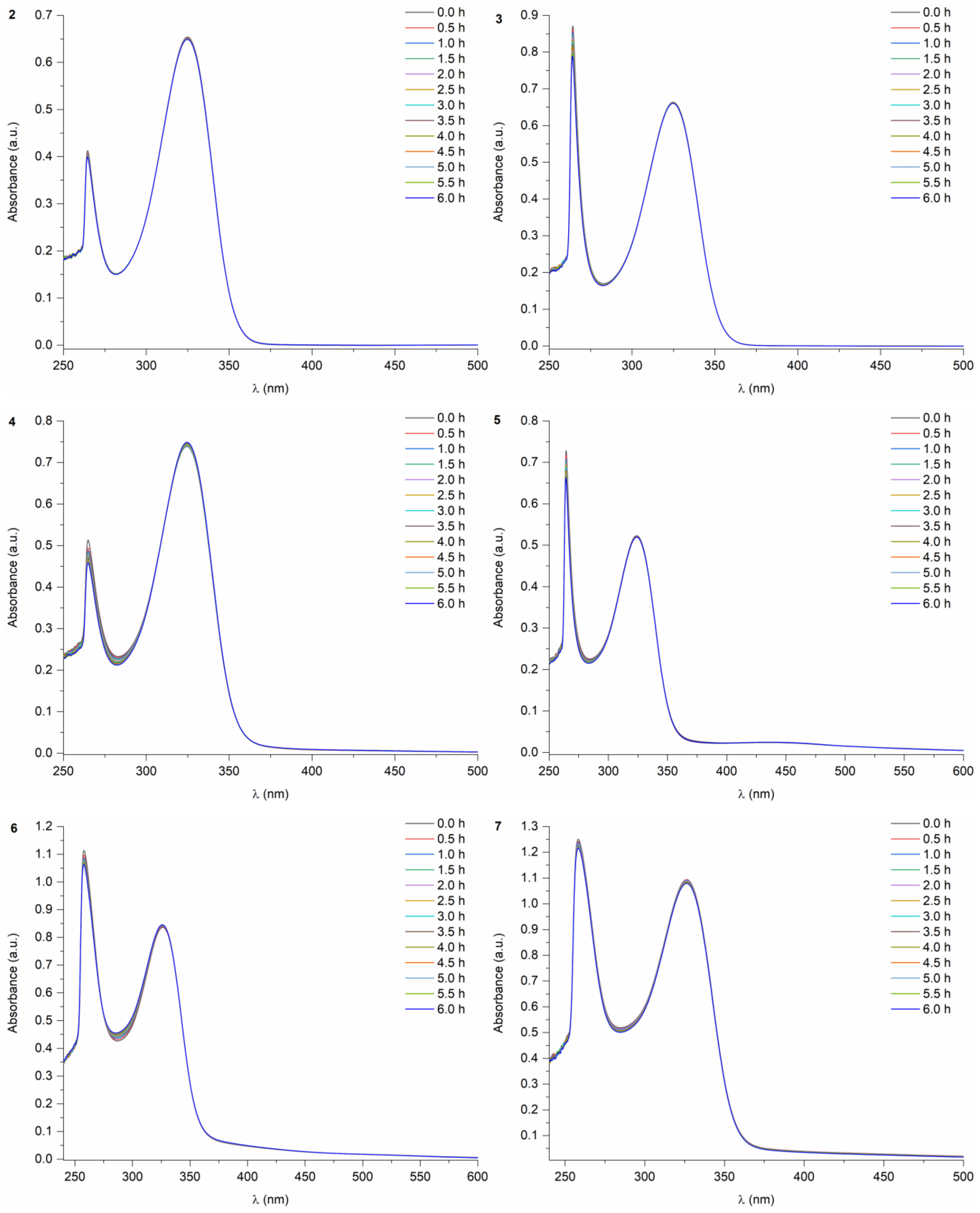


**Figure S83.** Stability in solution over time of complexes **2–7**.

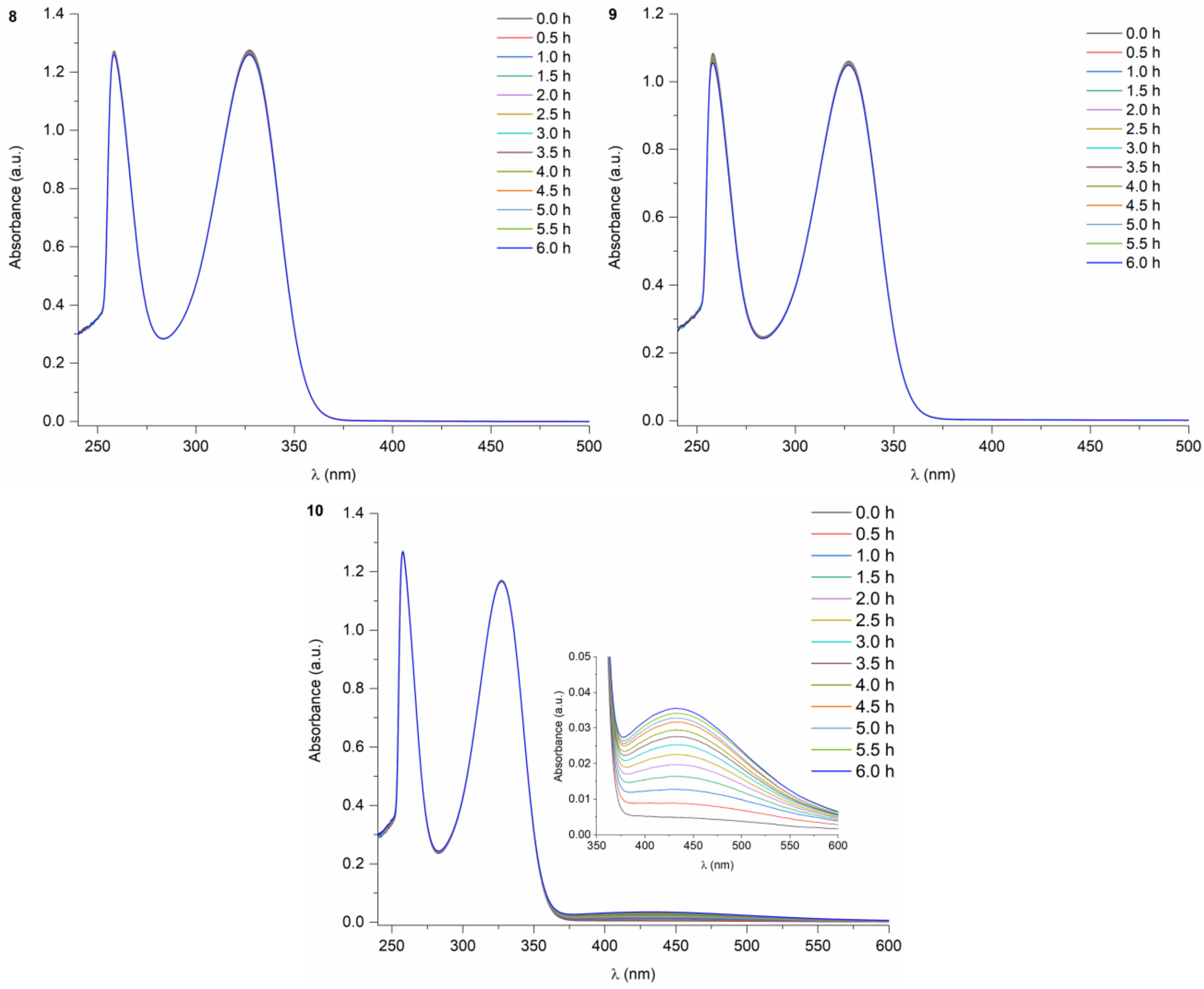


**Figure S84.** Stability in solution over time of complexes **8–10**.

## Solid-state spectra (DRS) and correspondingTauc plots

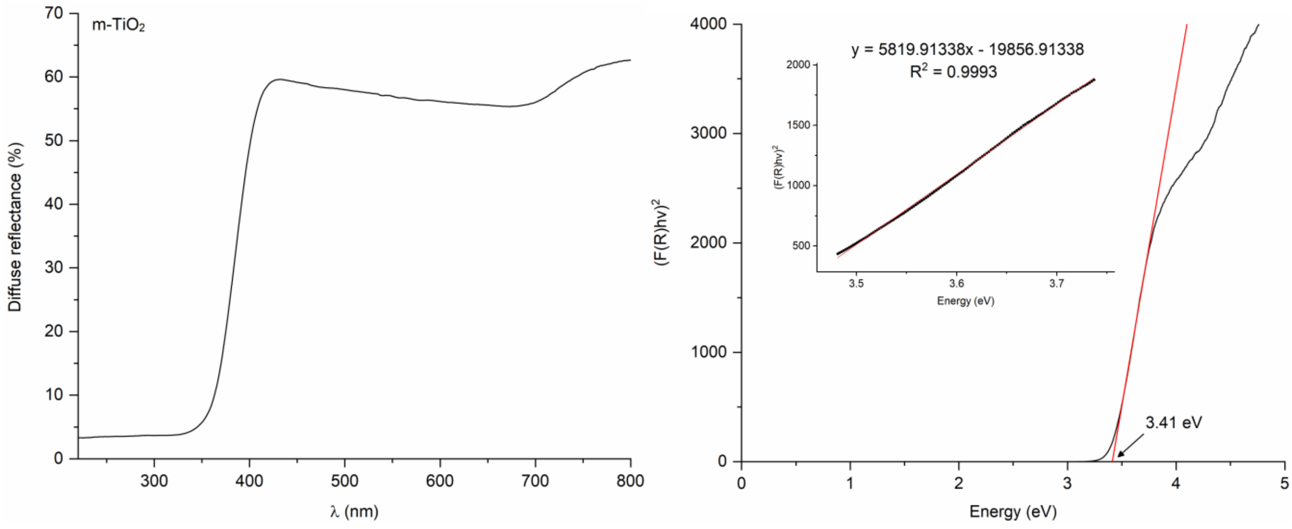


**Figure S85.** DRS spectrum and Tauc plot of m-$TiO_2$.

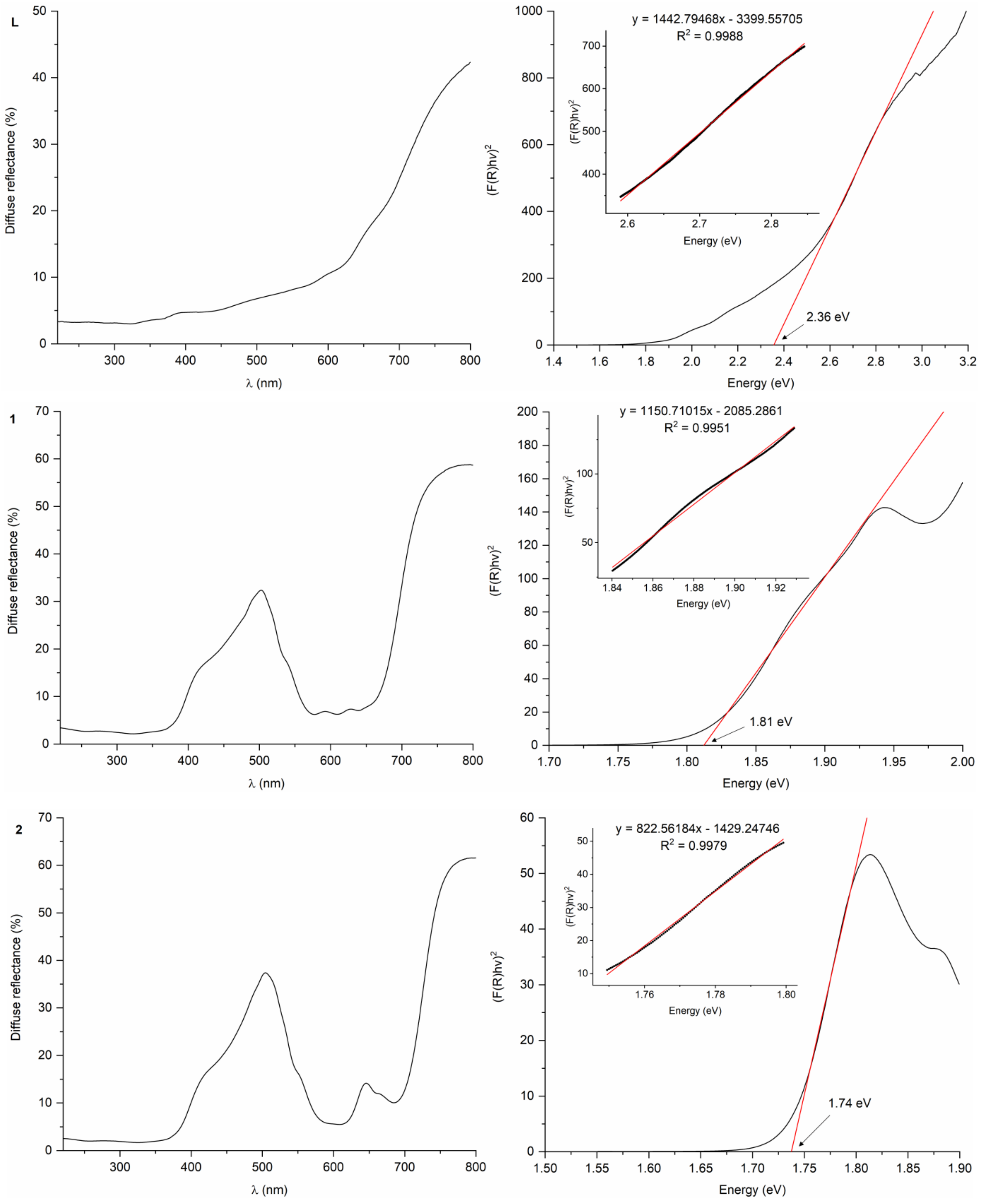


**Figure S86.** DRS spectra and Tauc plots of ligand **L** and complexes **1** and **2**.

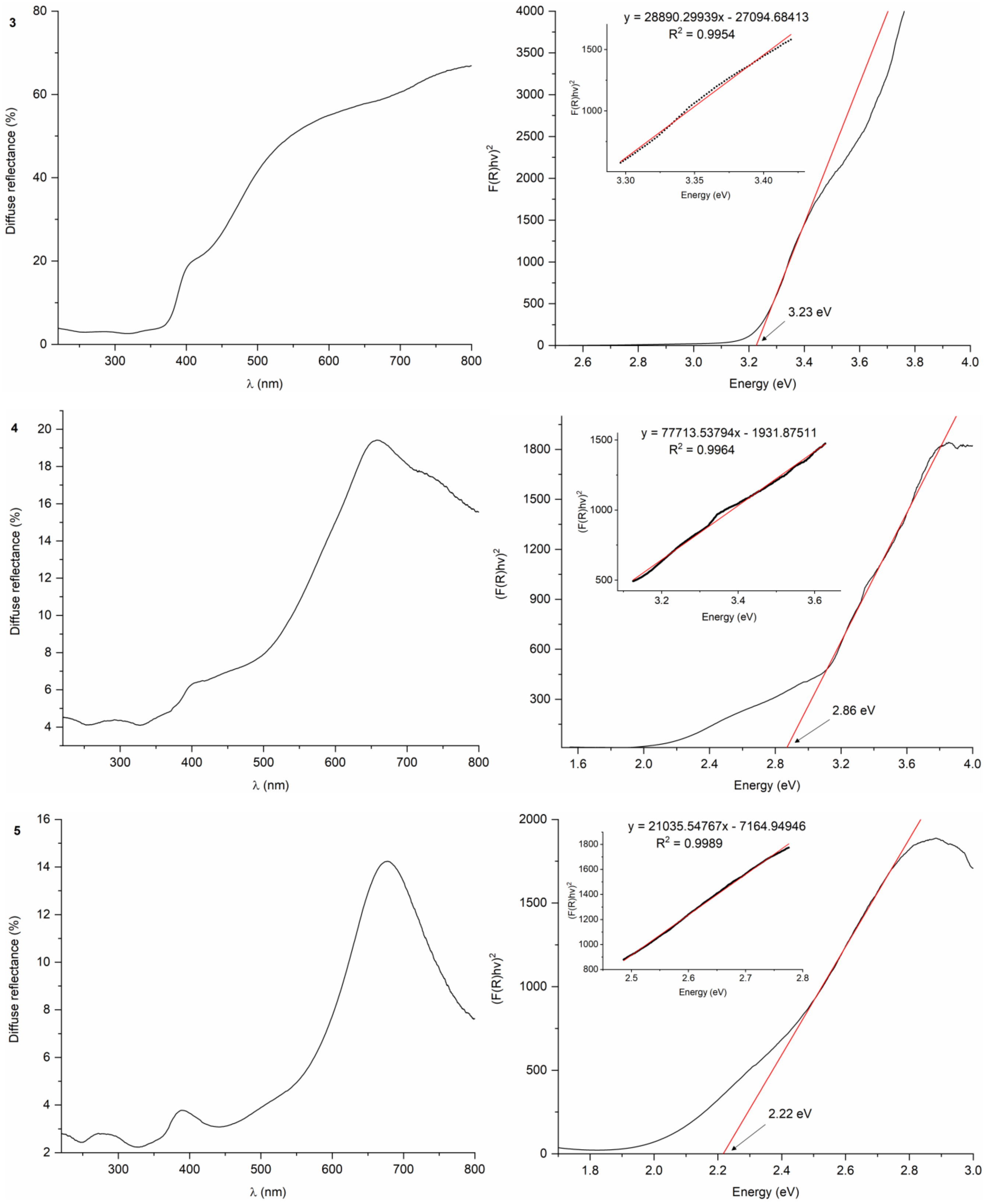


**Figure S87.** DRS spectra and Tauc plots of complexes **3–5**.

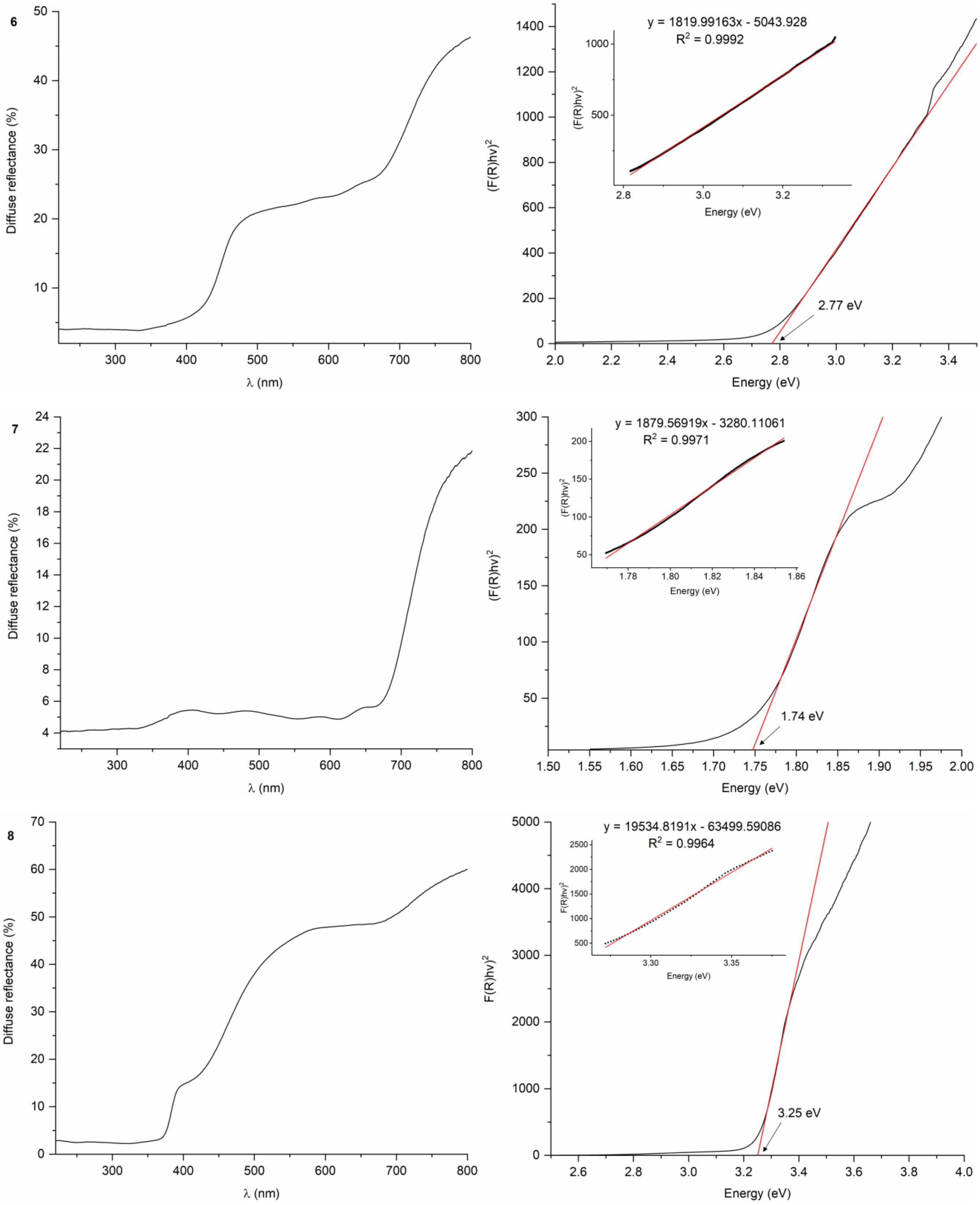


**Figure S88.** DRS spectra and Tauc plots of complexes **6–8**.

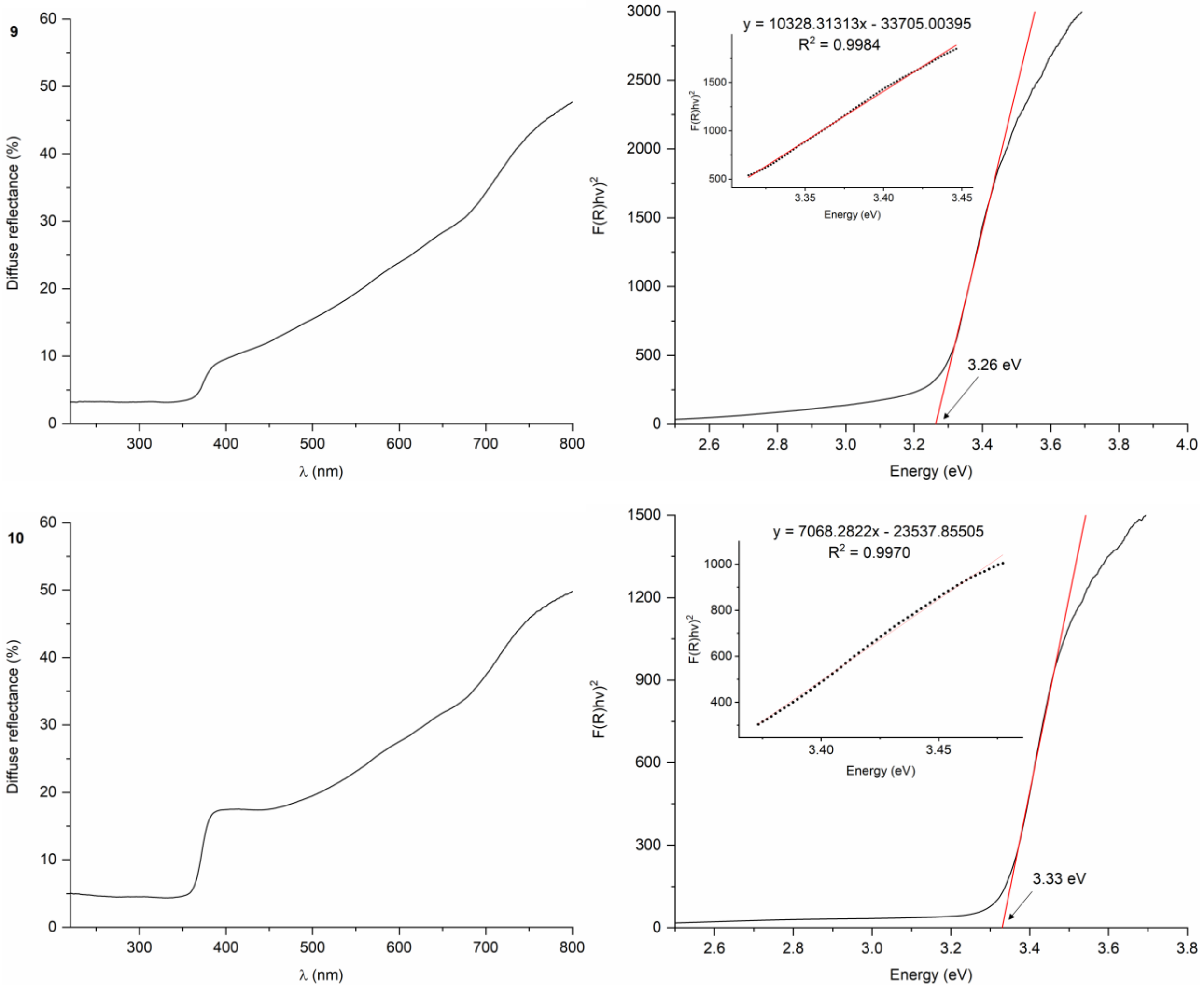


**Figure S89.** DRS spectra and Tauc plots of complexes **9** and **10**.

## Spectra of the dilutions and their respective straight-line equation graphs

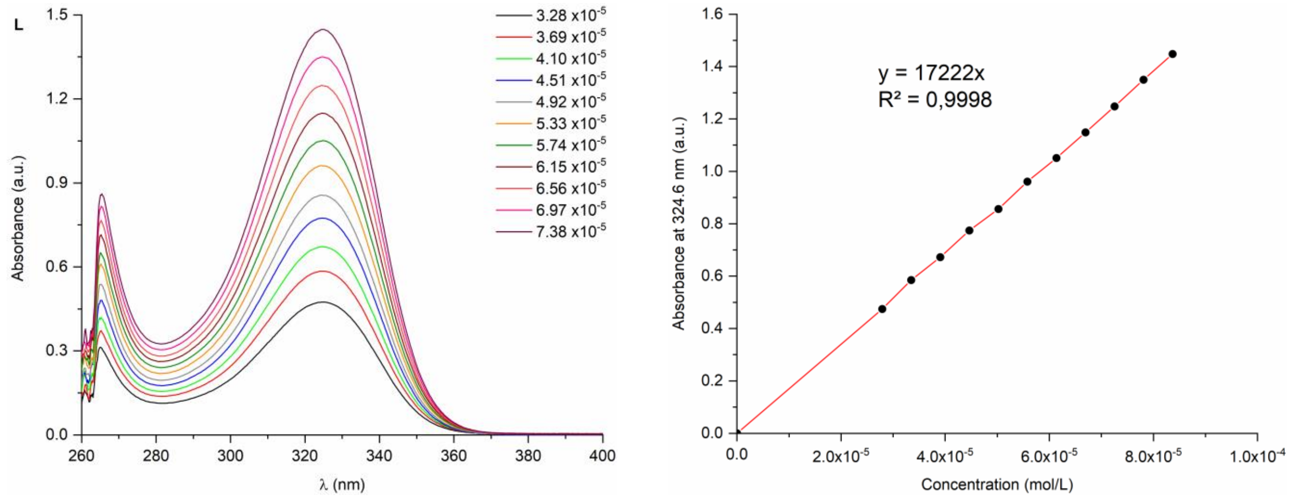


**Figure S90.** Dilution series and straight-line graph of ligand **L**.

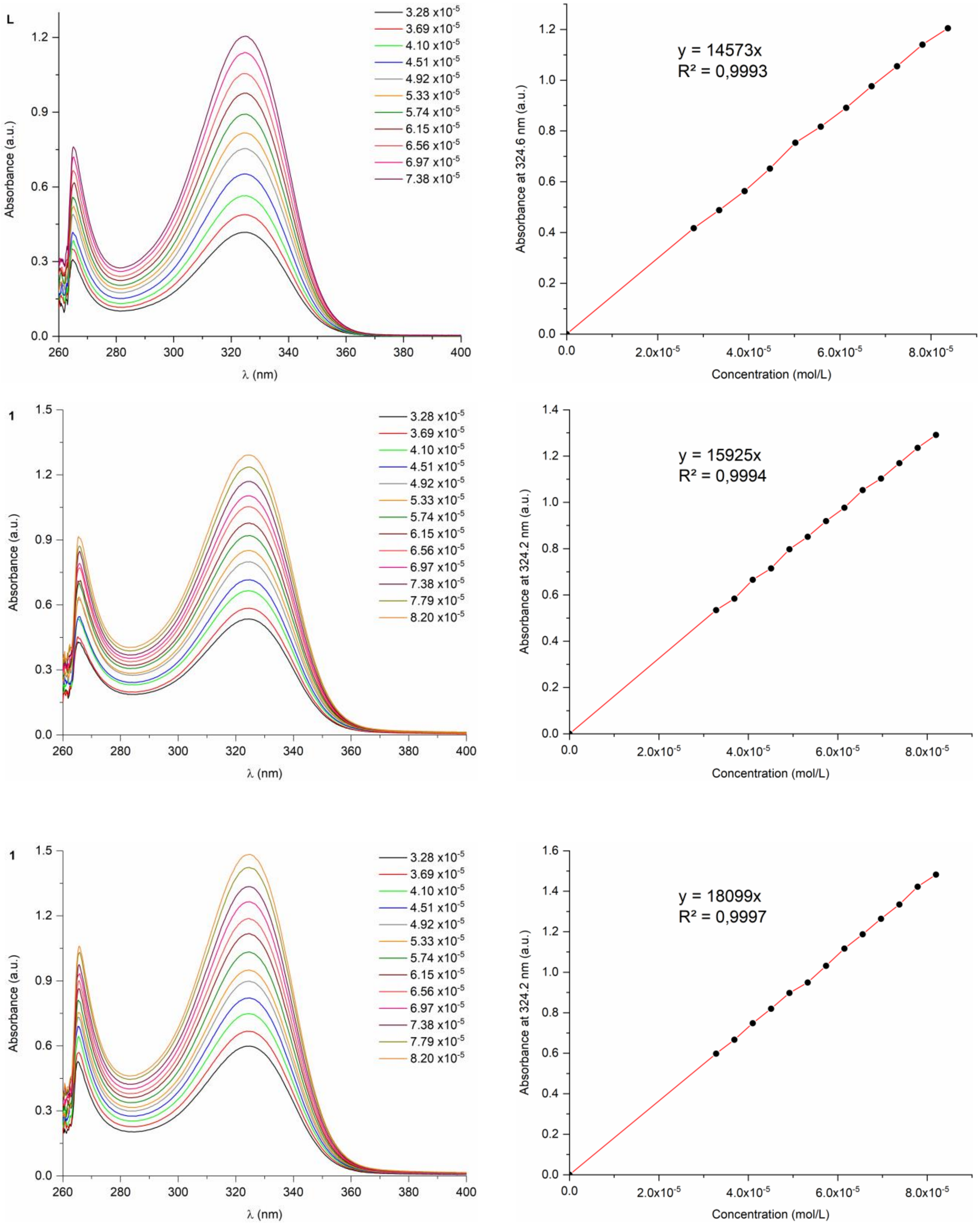


**Figure S91.** Dilutions series and straight-line graphs of ligand **L** and complex **1**.

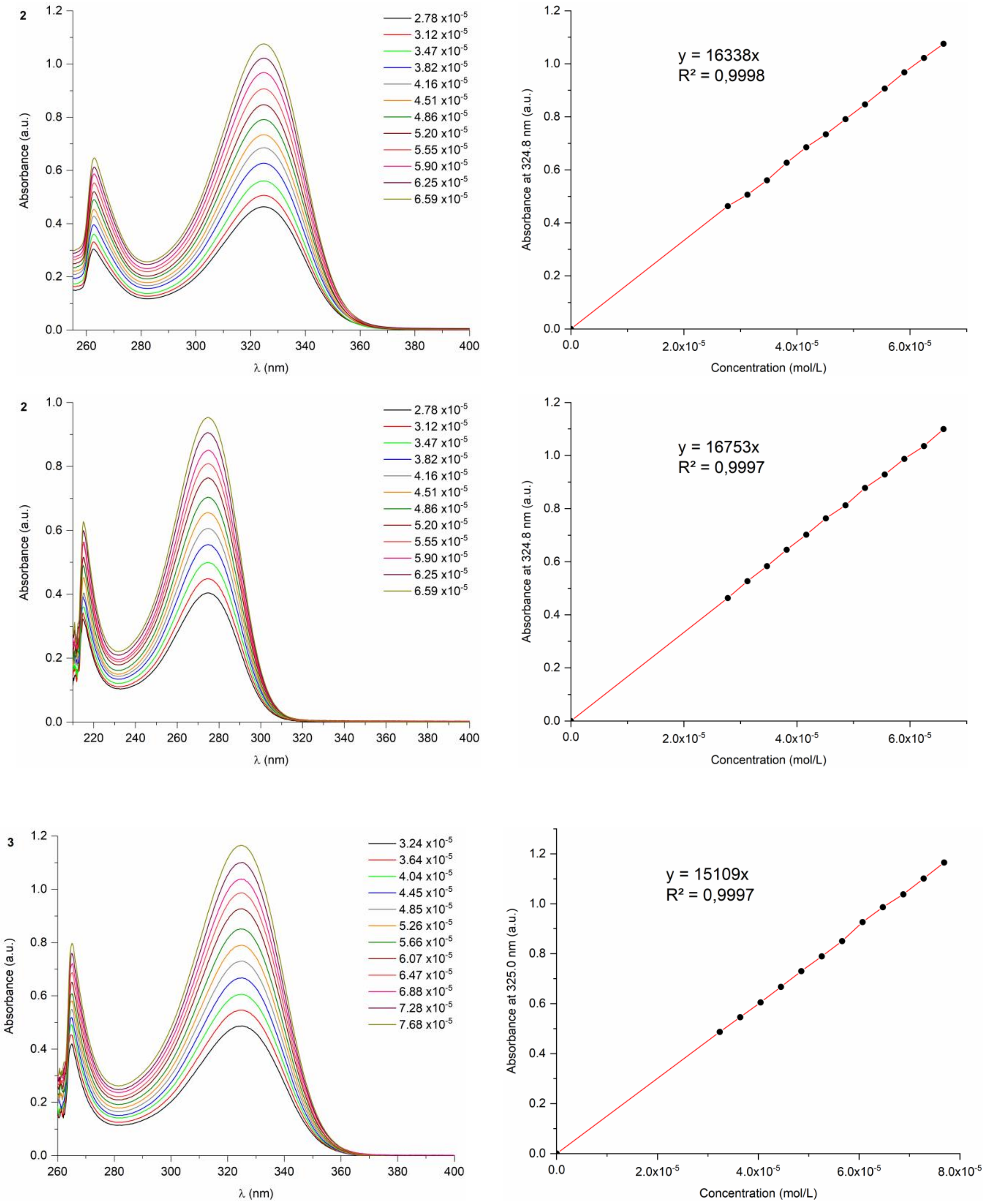


**Figure S92.** Dilutions series and straight-line graphs of complexes **2** and **3**.

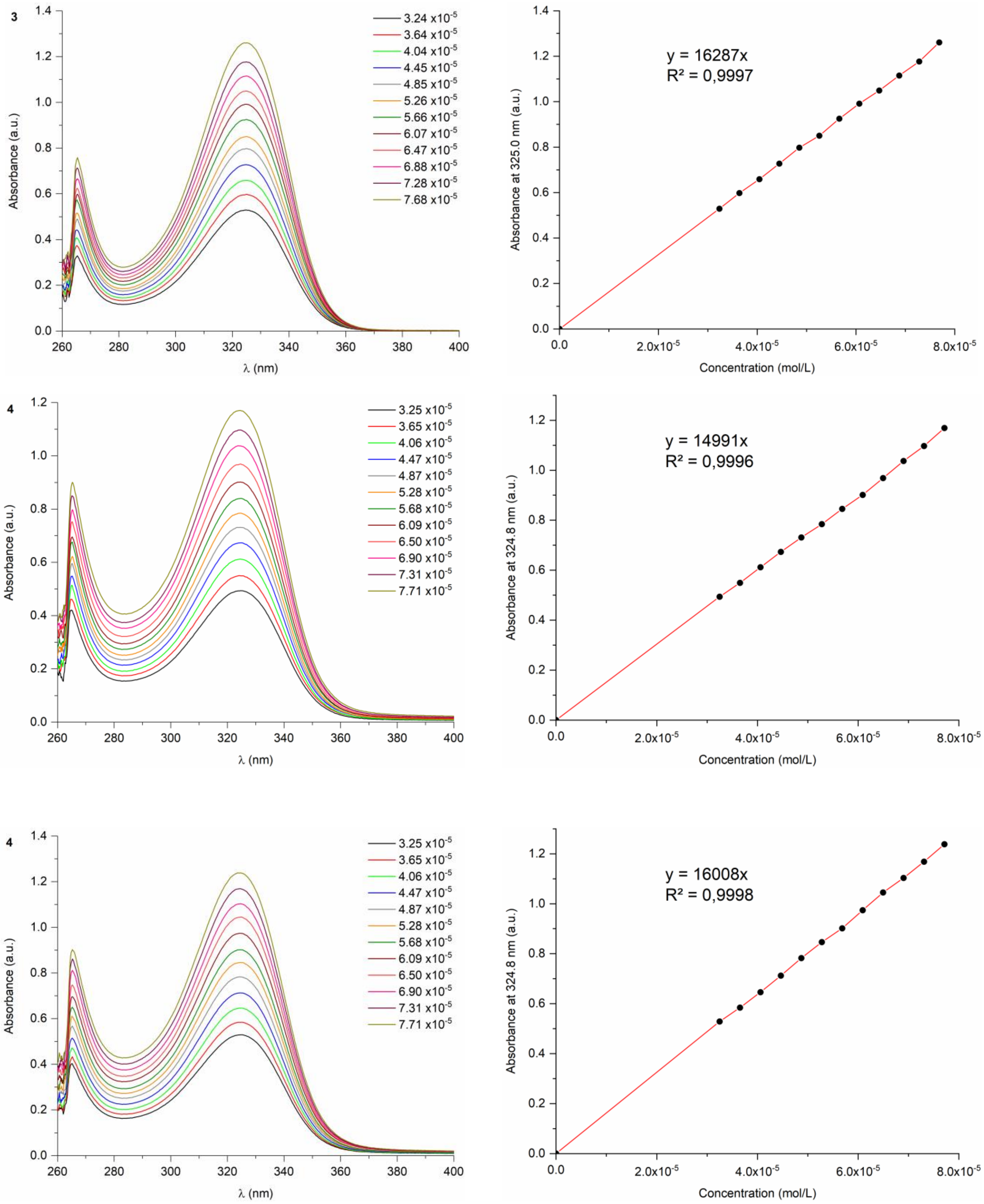


**Figure S93.** Dilutions series and straight-line graphs of complexes **3** and **4**.

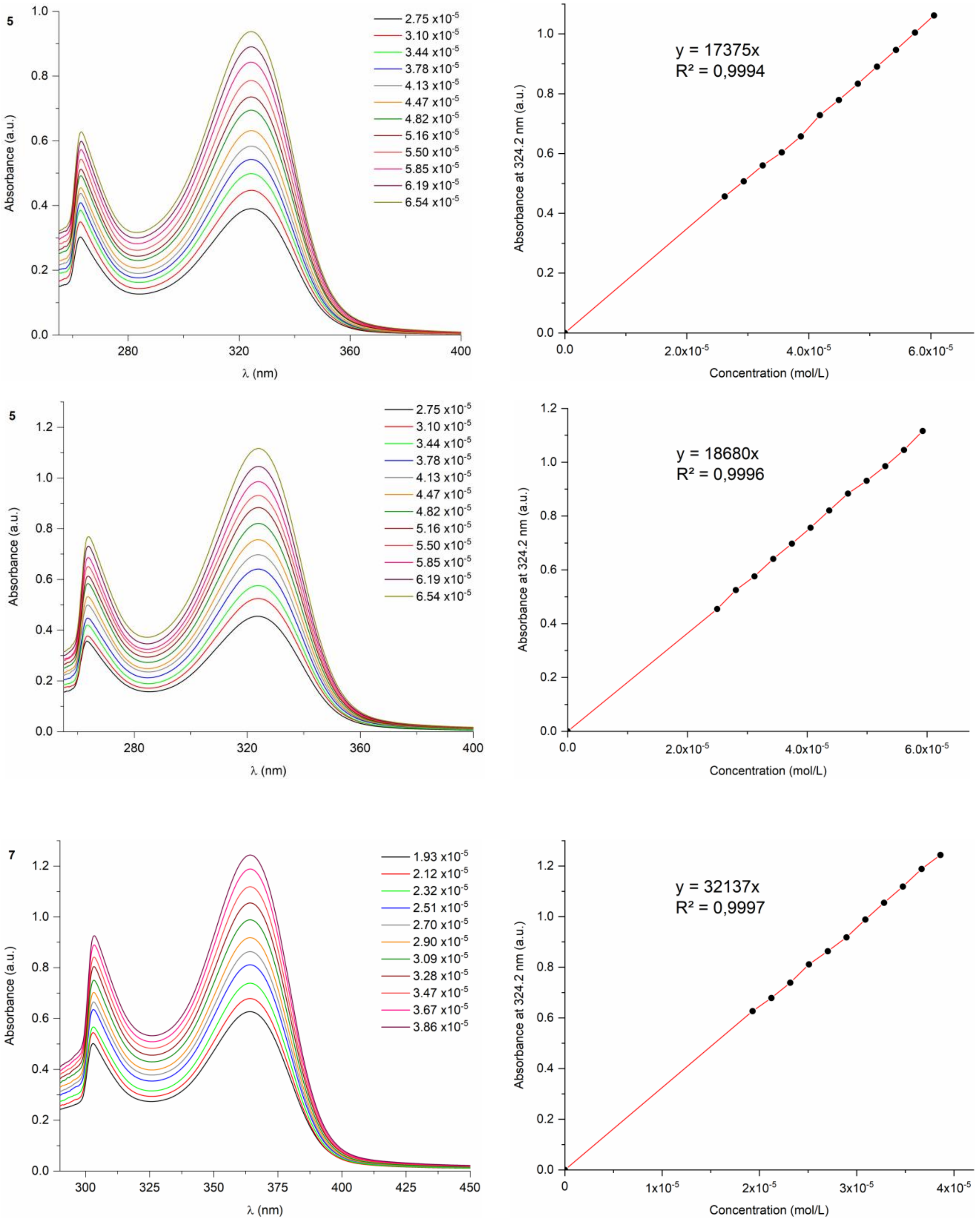


**Figure S94.** Dilutions series and straight-line graphs of complexes **5** and **7**.

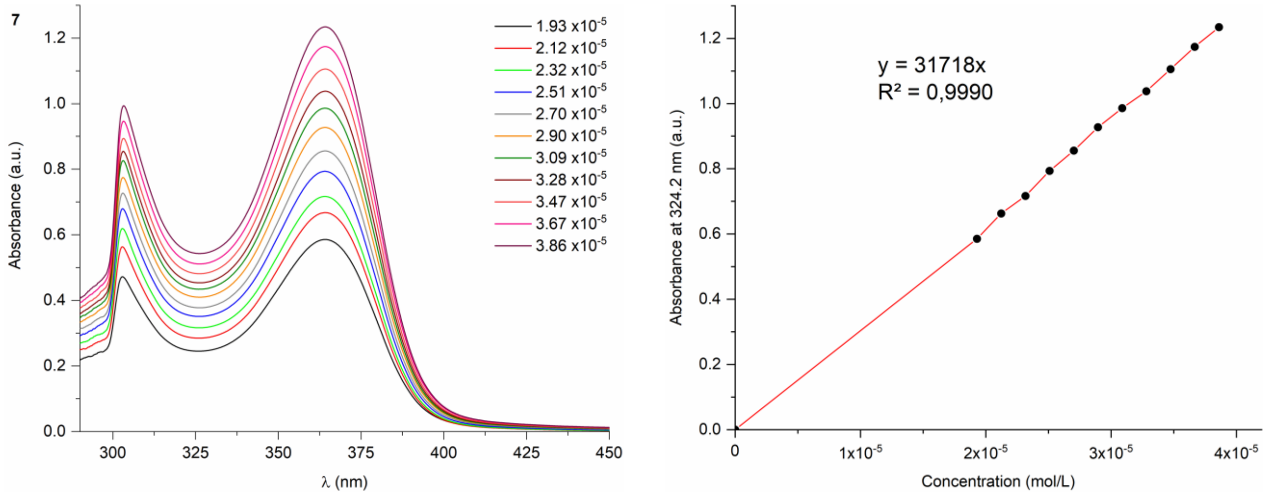


**Figure S95.** Dilution series and straight-line graph of complex **7**.

## CYCLIC VOLTAMMETRY (CV)

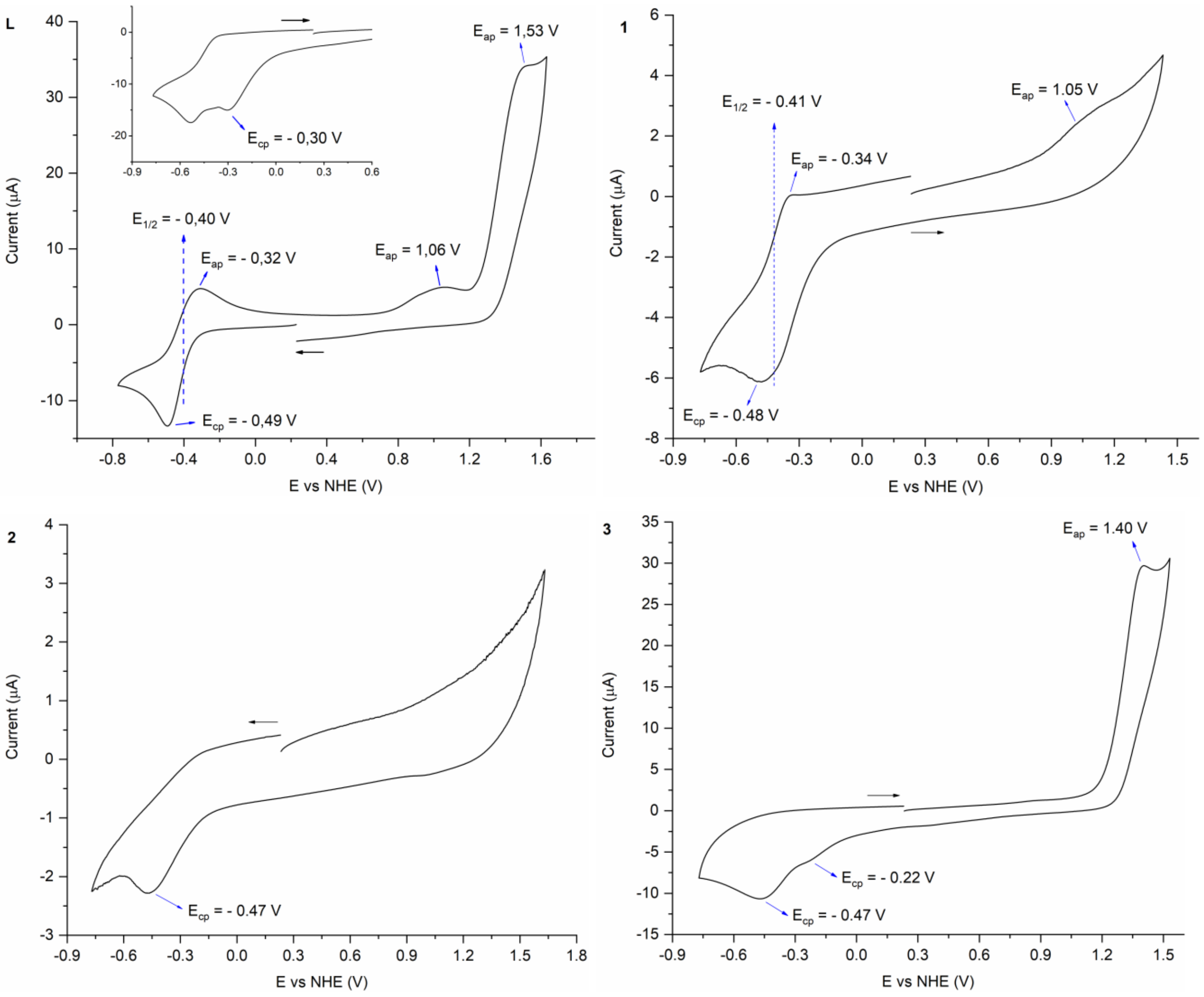


**Figure S96.** Voltammograms of ligand **L** and complexes **1–3**.

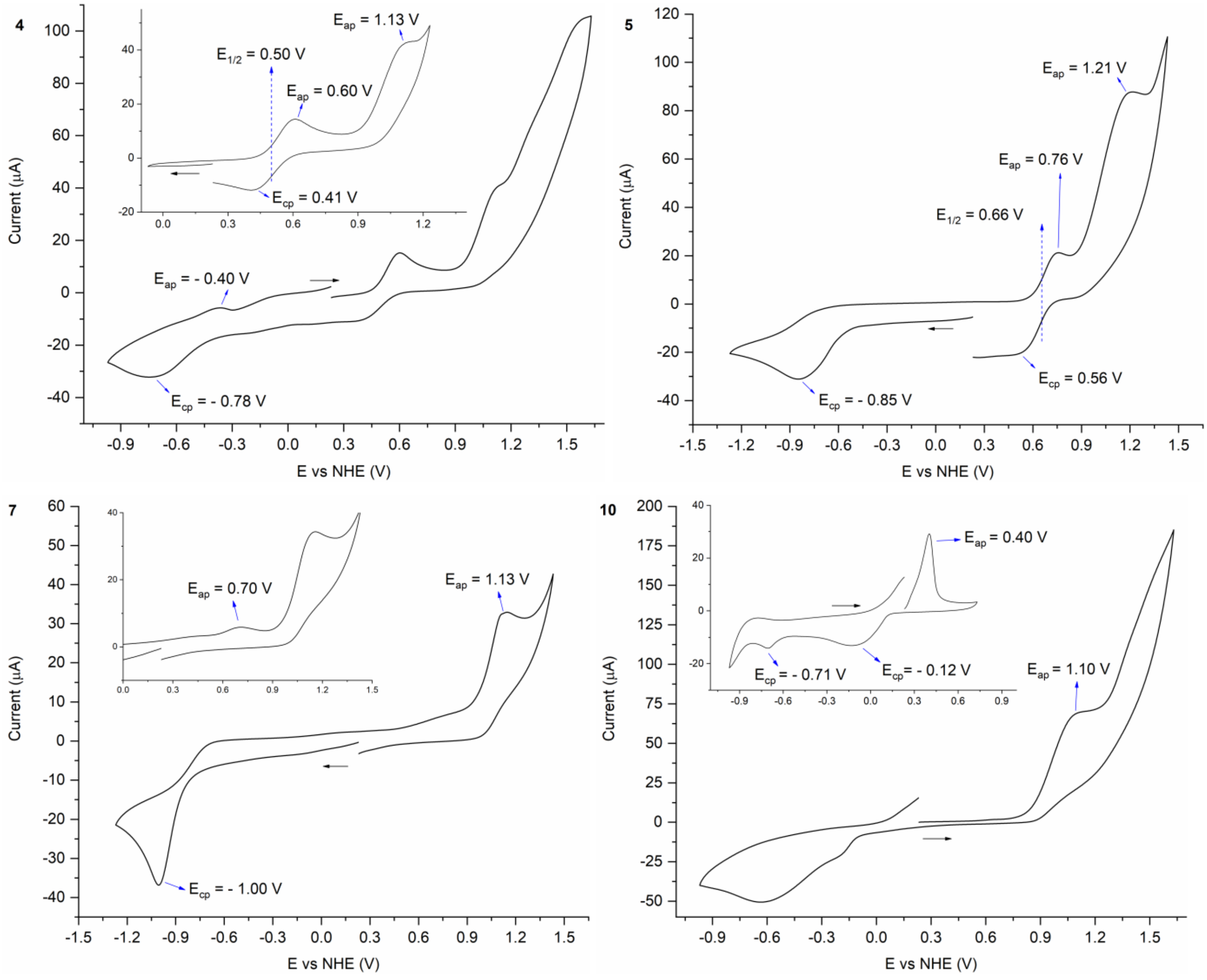


**Figure S97.** Voltammograms of complexes **4**, **5**, **7**, and **10**.

## CHARACTERIZATION OF THE PHOTOCATALYSTS

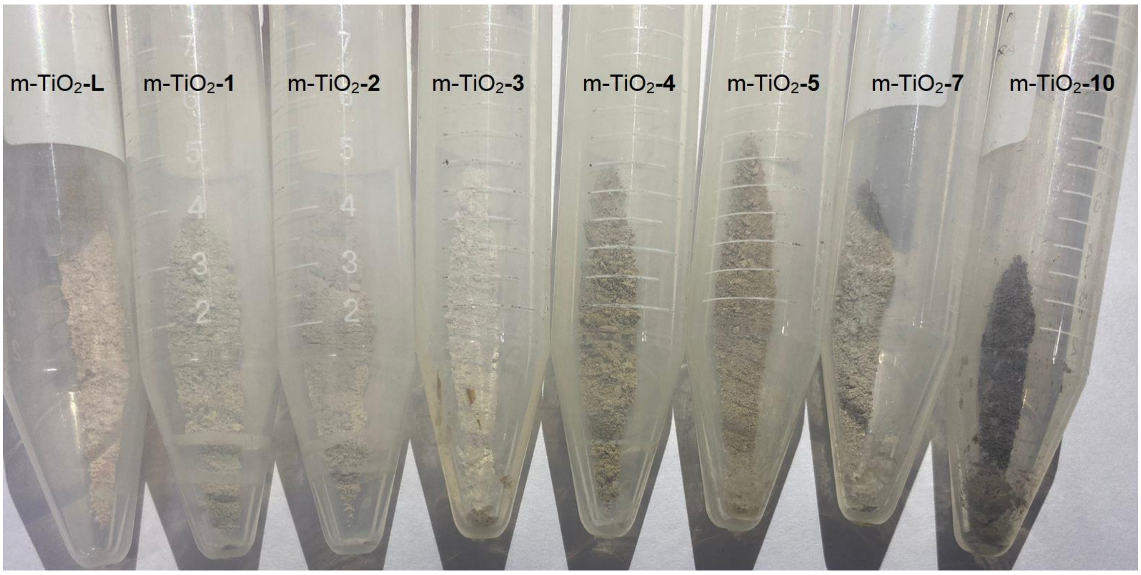


**Figure S98.** Photocatalysts m-$TiO_2$-**n**.

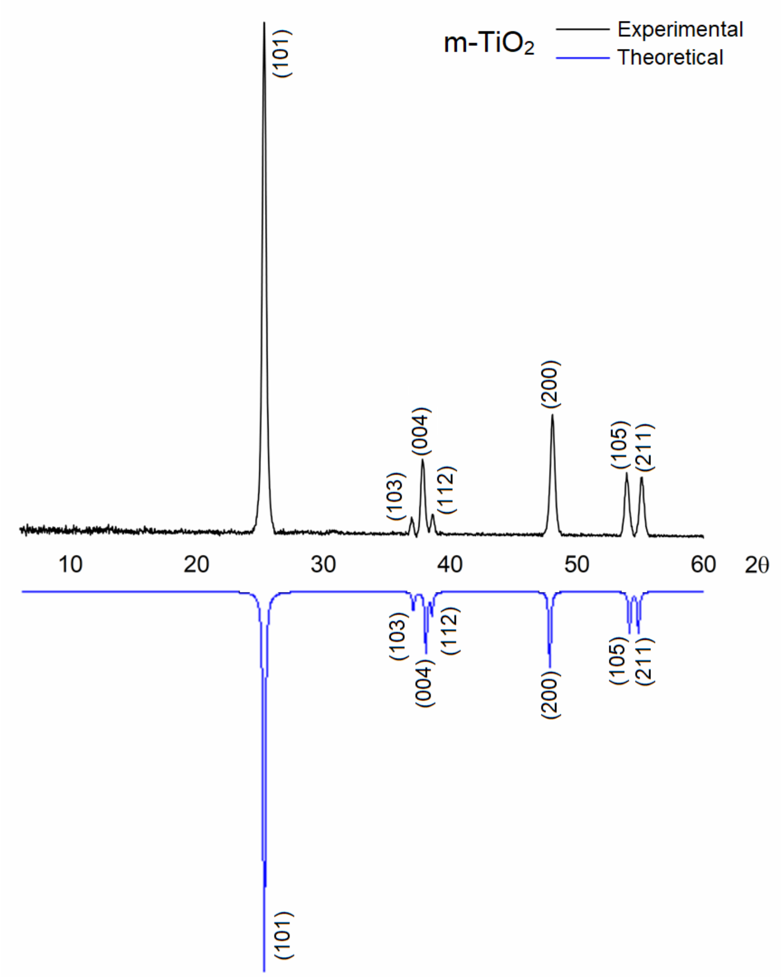


**Figure S99.** Theoretical and experimental powder diffractograms of m-$TiO_2$.

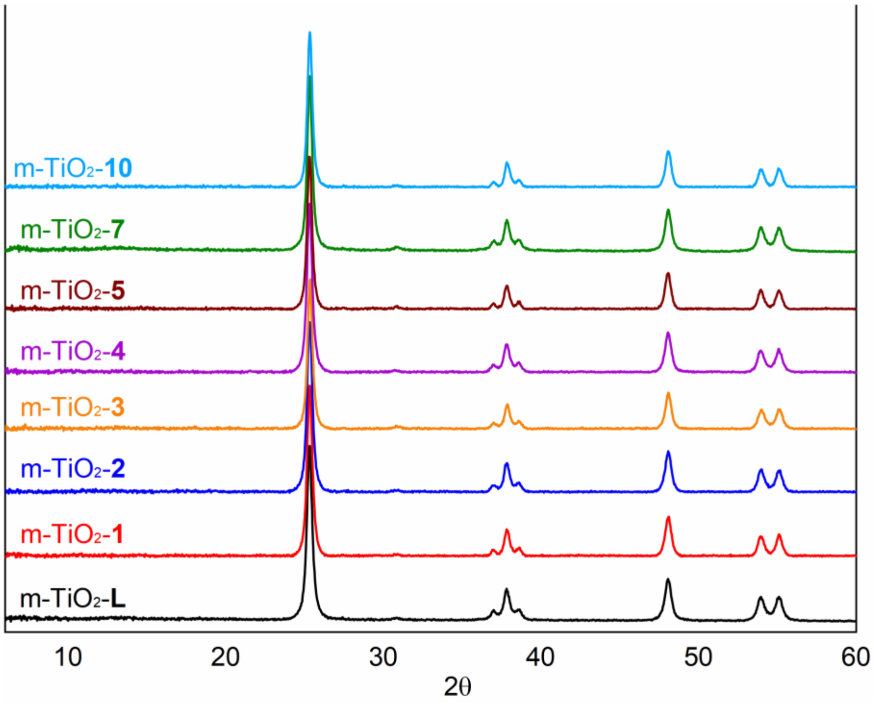


**Figure S100.** Powder diffractograms of the photocatalysts.

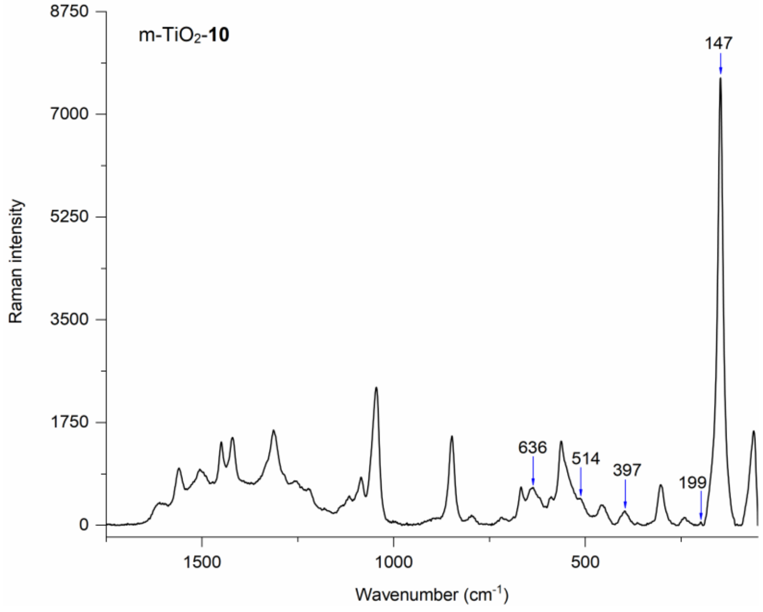


**Figure S101.** Raman spectrum of m-$TiO_2$-**10**.

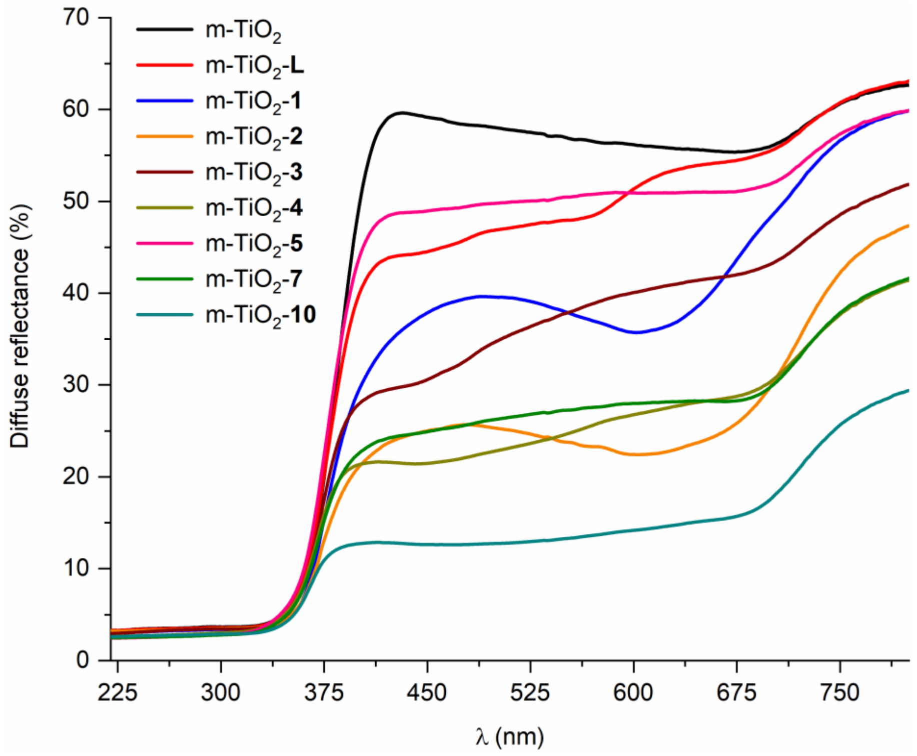


**Figure S102.** DRS spectra of the photocatalysts.

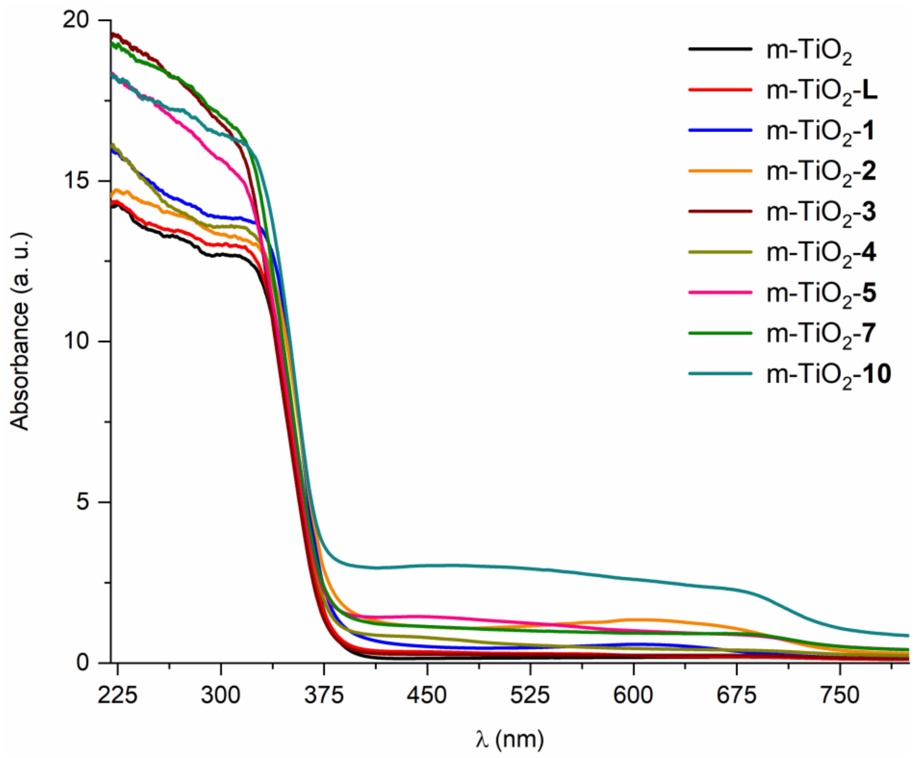


**Figure S103.** Absorbance spectra of the photocatalysts calculated from DRS data.

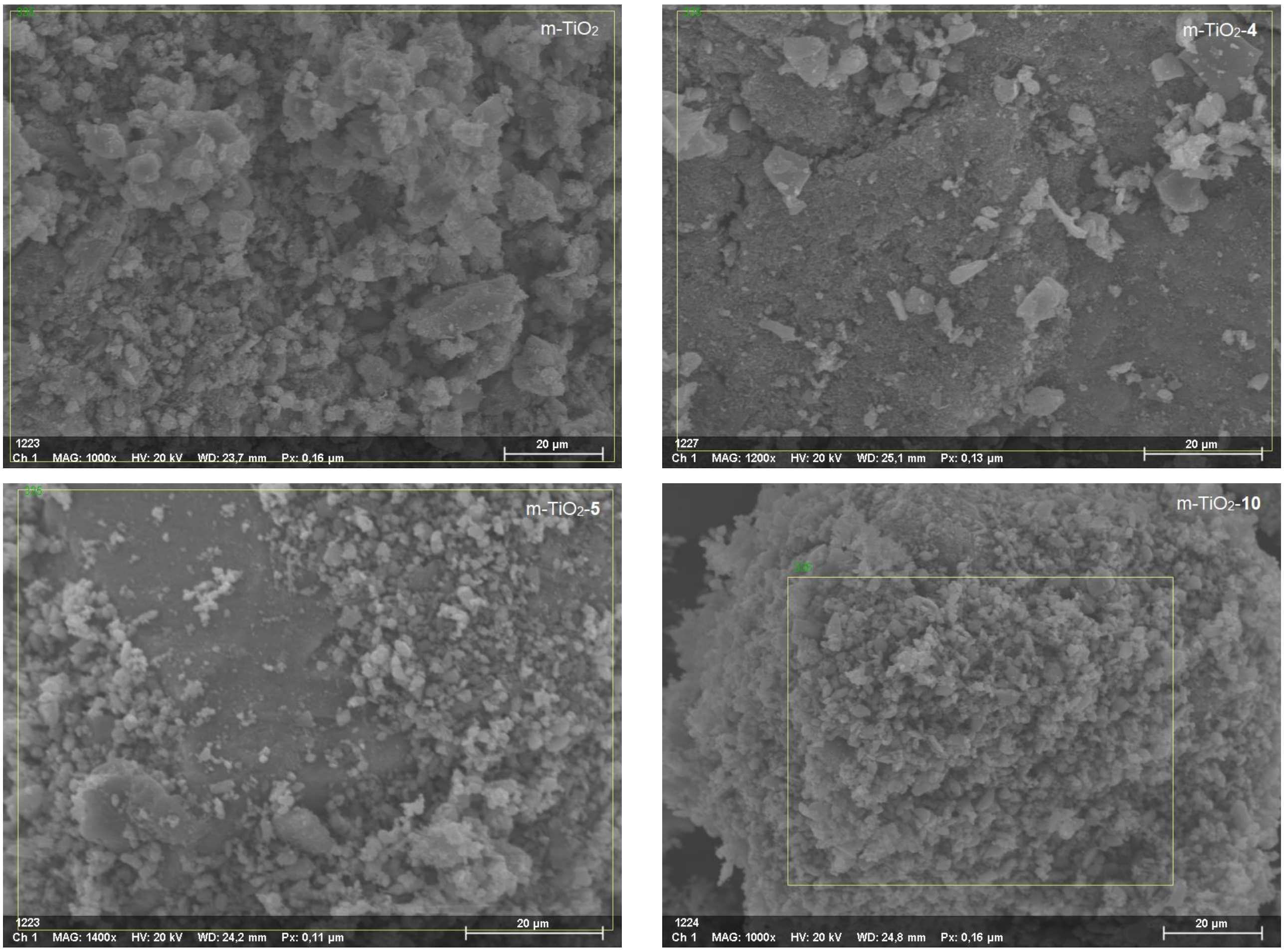


**Figure S104.** Scanning electron microscopy (SEM) images of the photocatalysts.

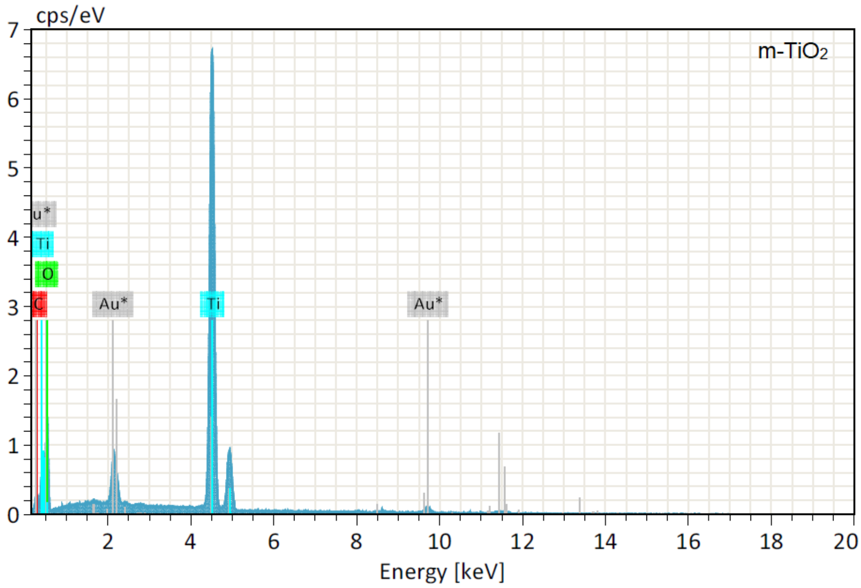


**Figure S105.** Energy-dispersive X-ray spectroscopy (EDS) spectrum of m-$TiO_2$.

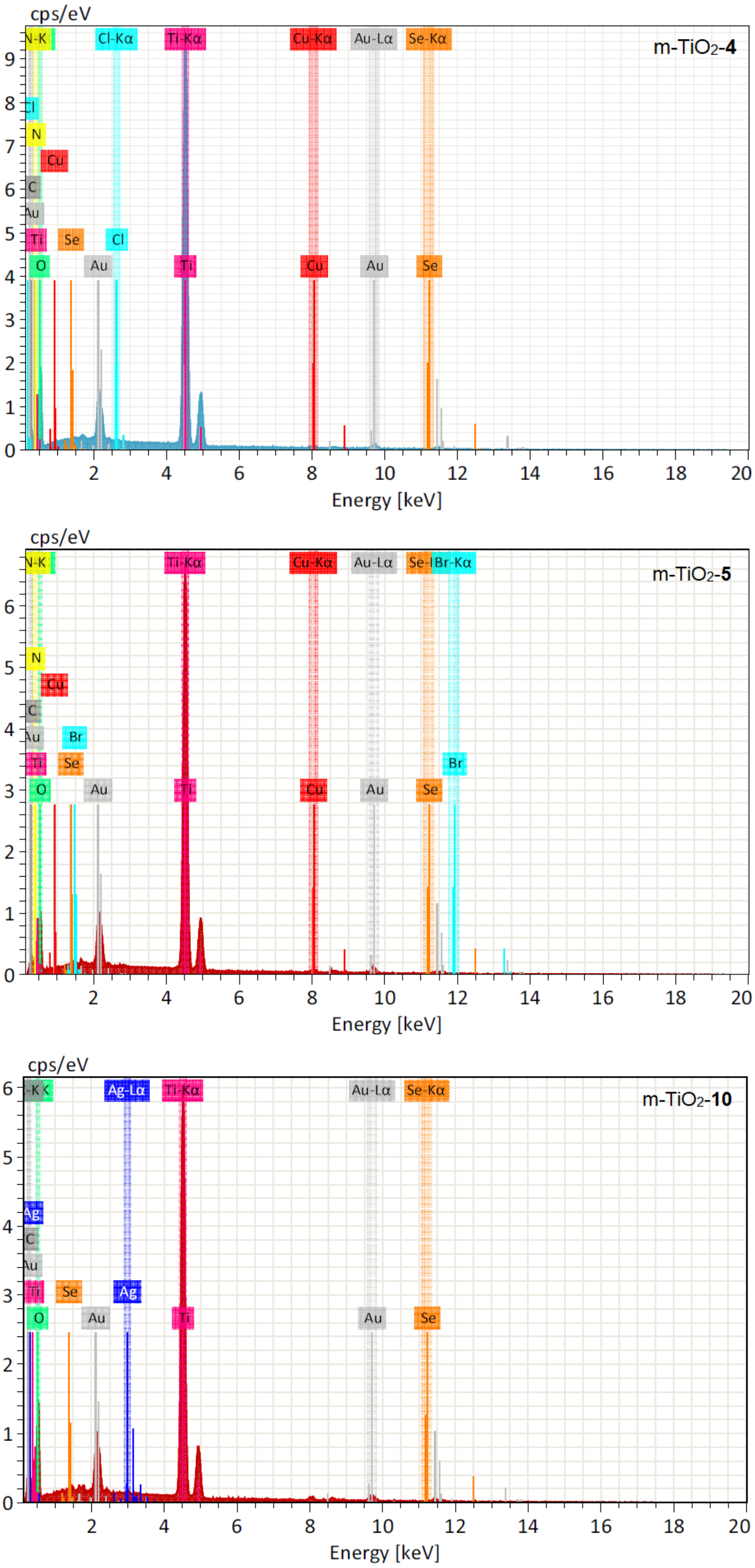


**Figure S106.** EDS spectra of the photocatalysts.

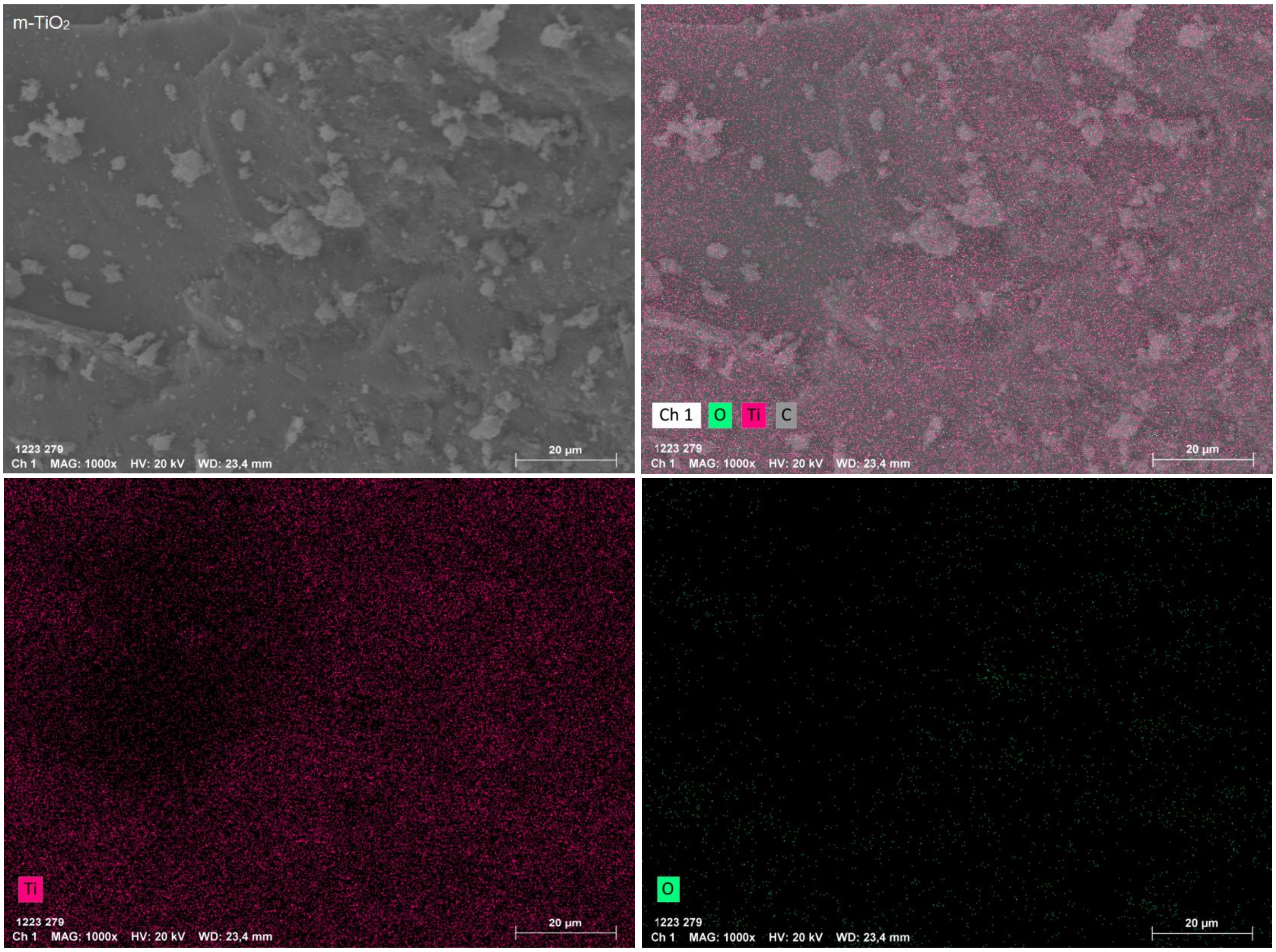


**Figure S107.** Elemental mapping of m-$TiO_2$.

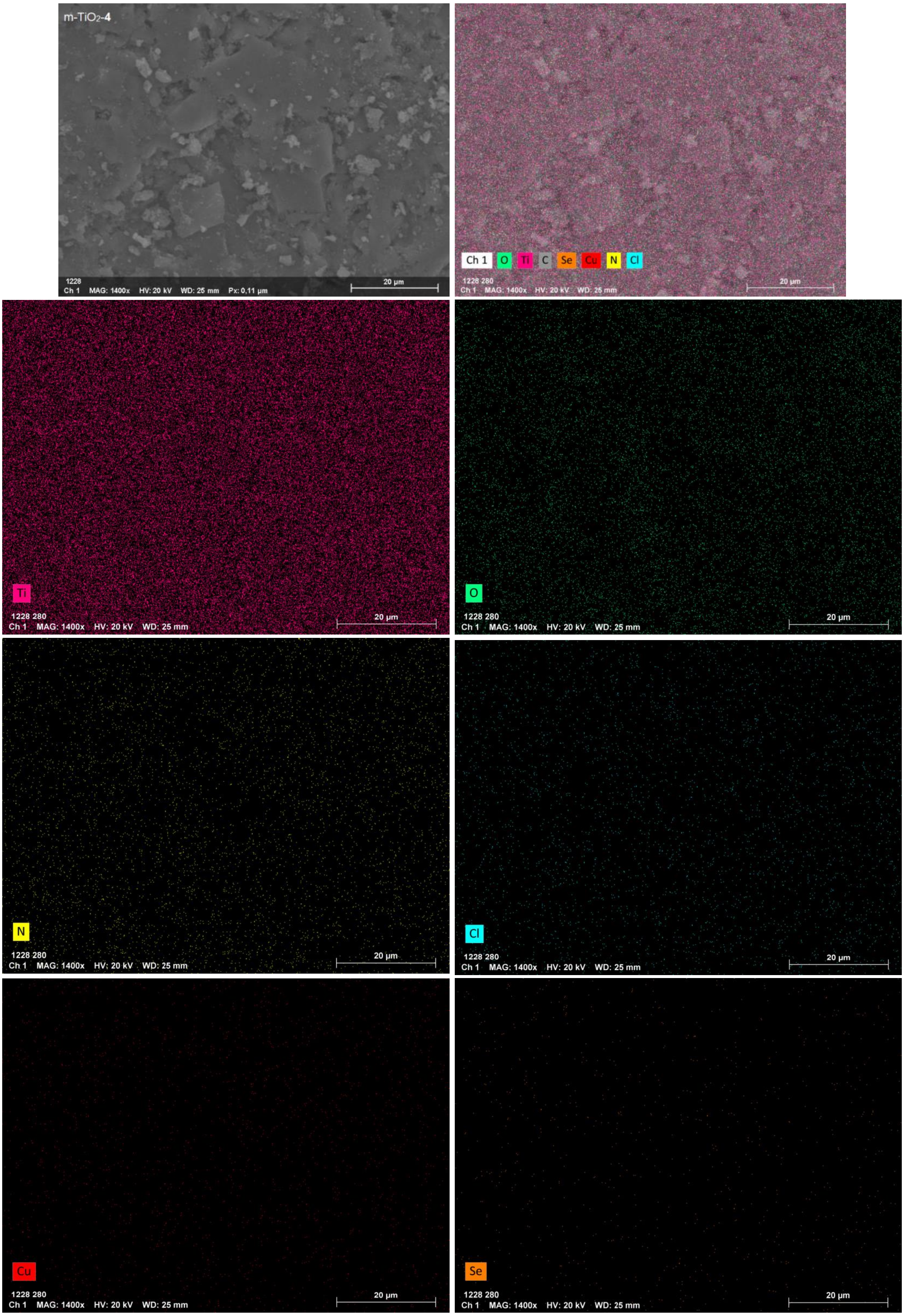


**Figure S108.** Elemental mapping of m-$TiO_2$-**4**.

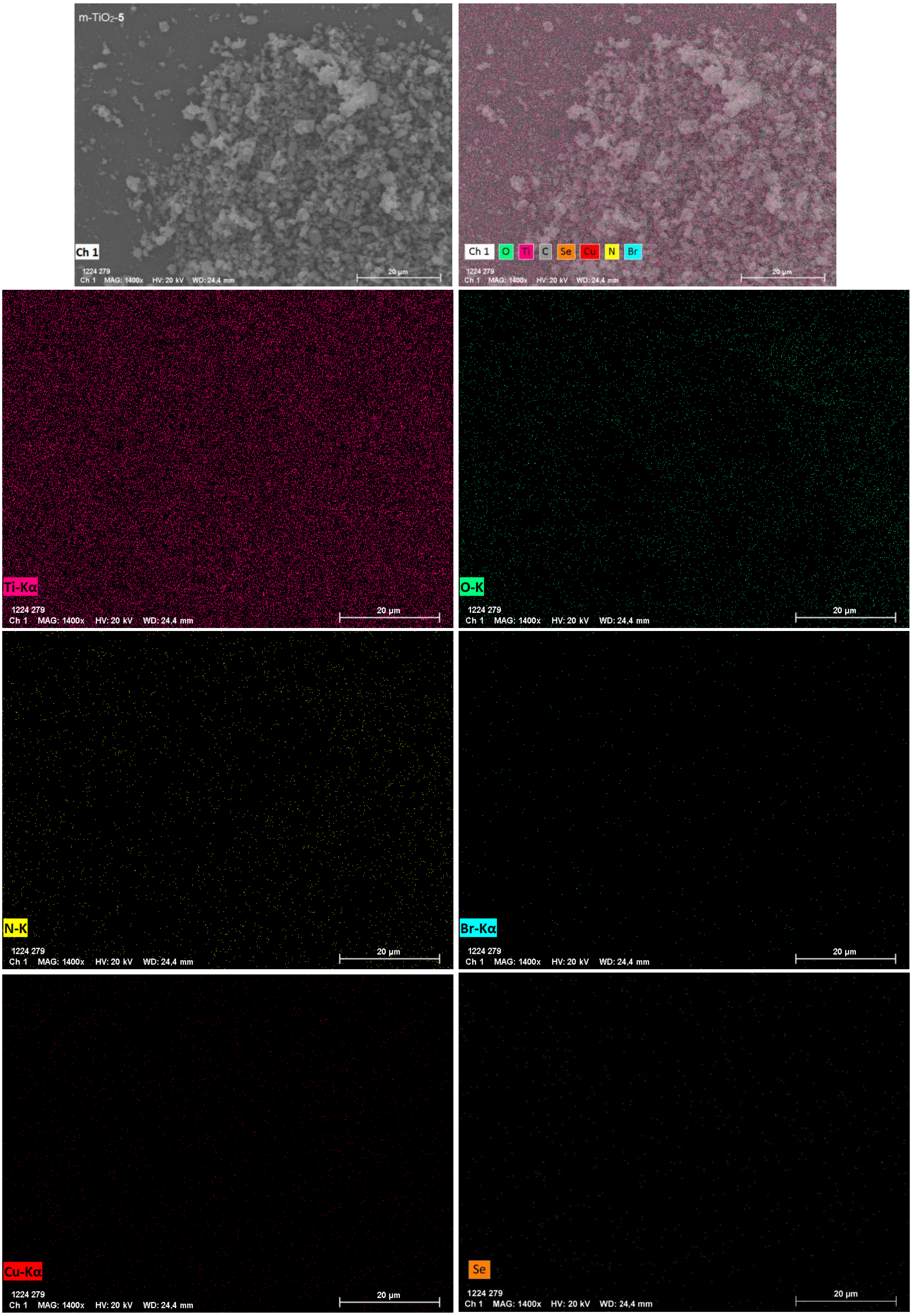


**Figure S109.** Elemental mapping of m-$TiO_2$-**5**.

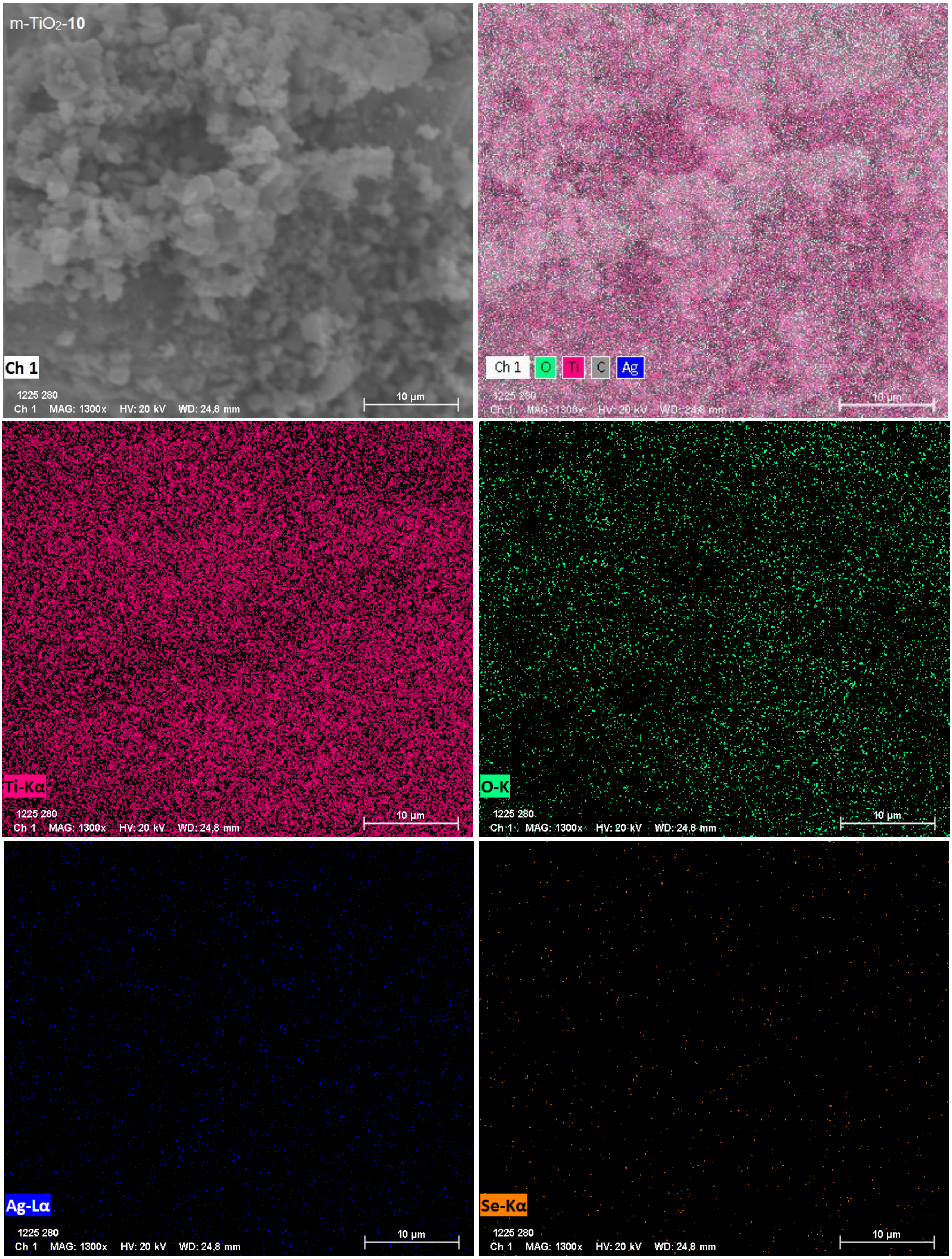


**Figure S110.** Elemental mapping of m-$TiO_2$-**10**.

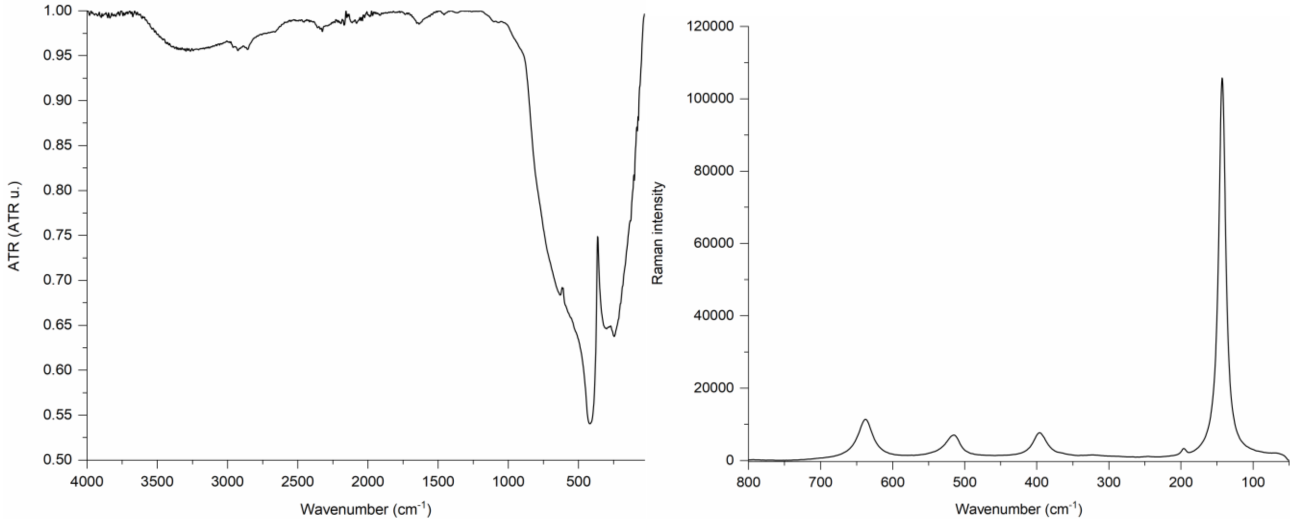


**Figure S111.** FT-IR and Raman spectra of m-$TiO_2$-**7** post-photocatalysis.

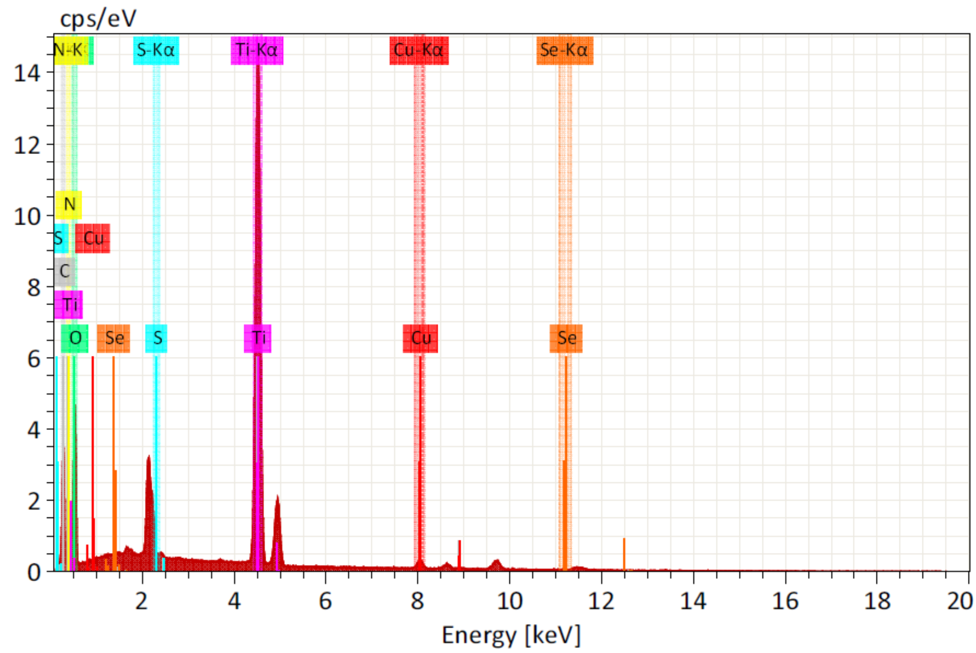


**Figure S112.** EDS spectrum of m-$TiO_2$-**7** post-photocatalysis.

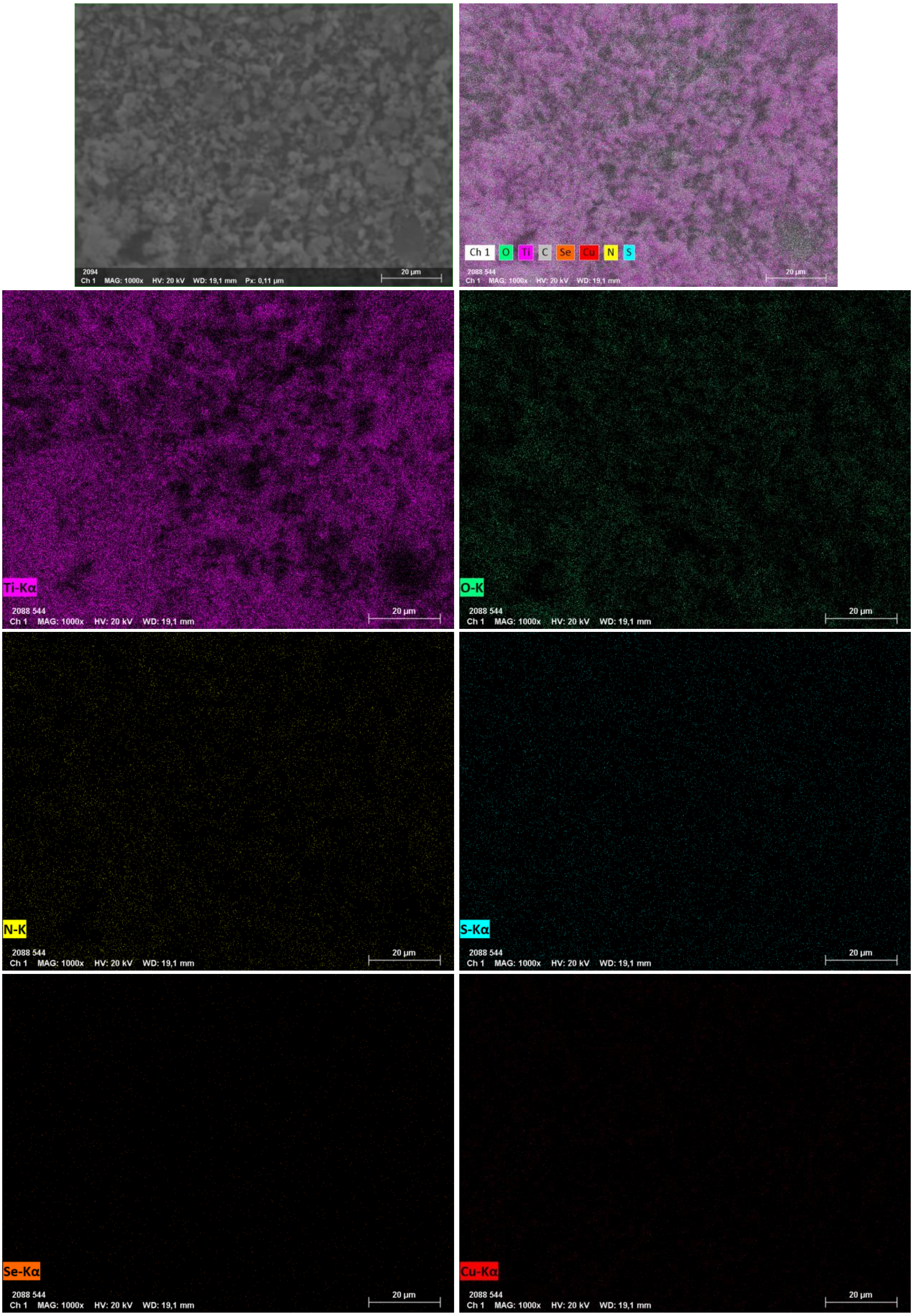


**Figure S113.** Elemental mapping of m-$TiO_2$-**7** post-photocatalysis.

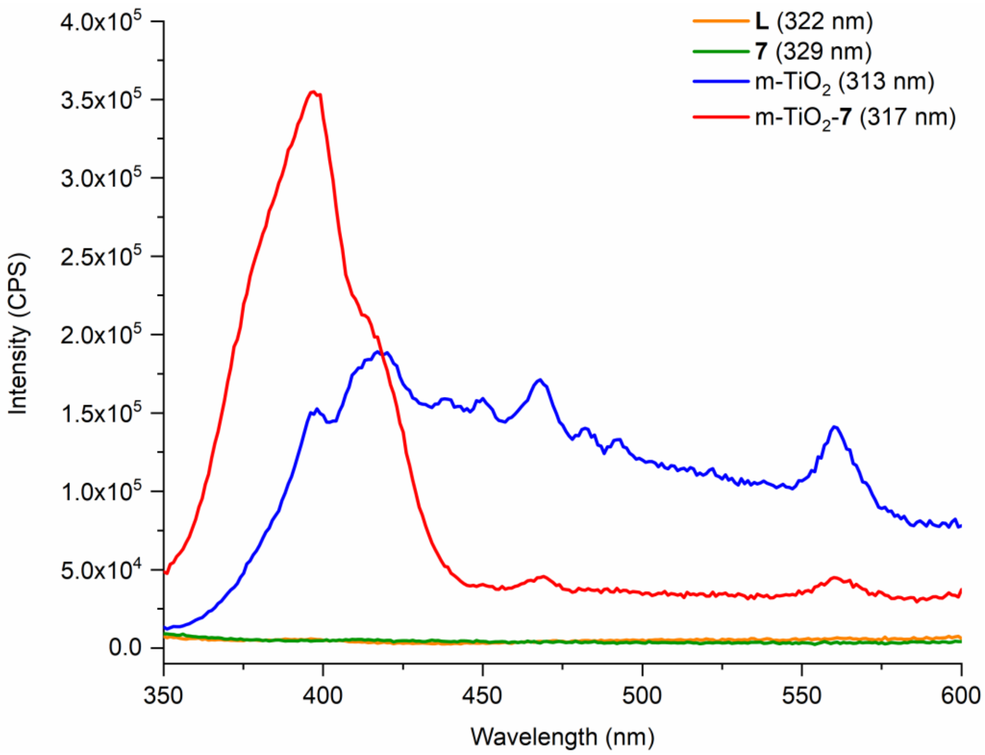


**Figure S114.** Solid-state photoluminescence (PL) emission spectra of ligand **L**, complex **7**, m-$TiO_2$, and m-$TiO_2$-**7**, along with their respective excitation wavelengths ($\lambda_{exc}$). The $\lambda_{exc}$ values were chosen based on the maximum absorbance wavelengths observed in the absorbance spectra obtained from DRS data.

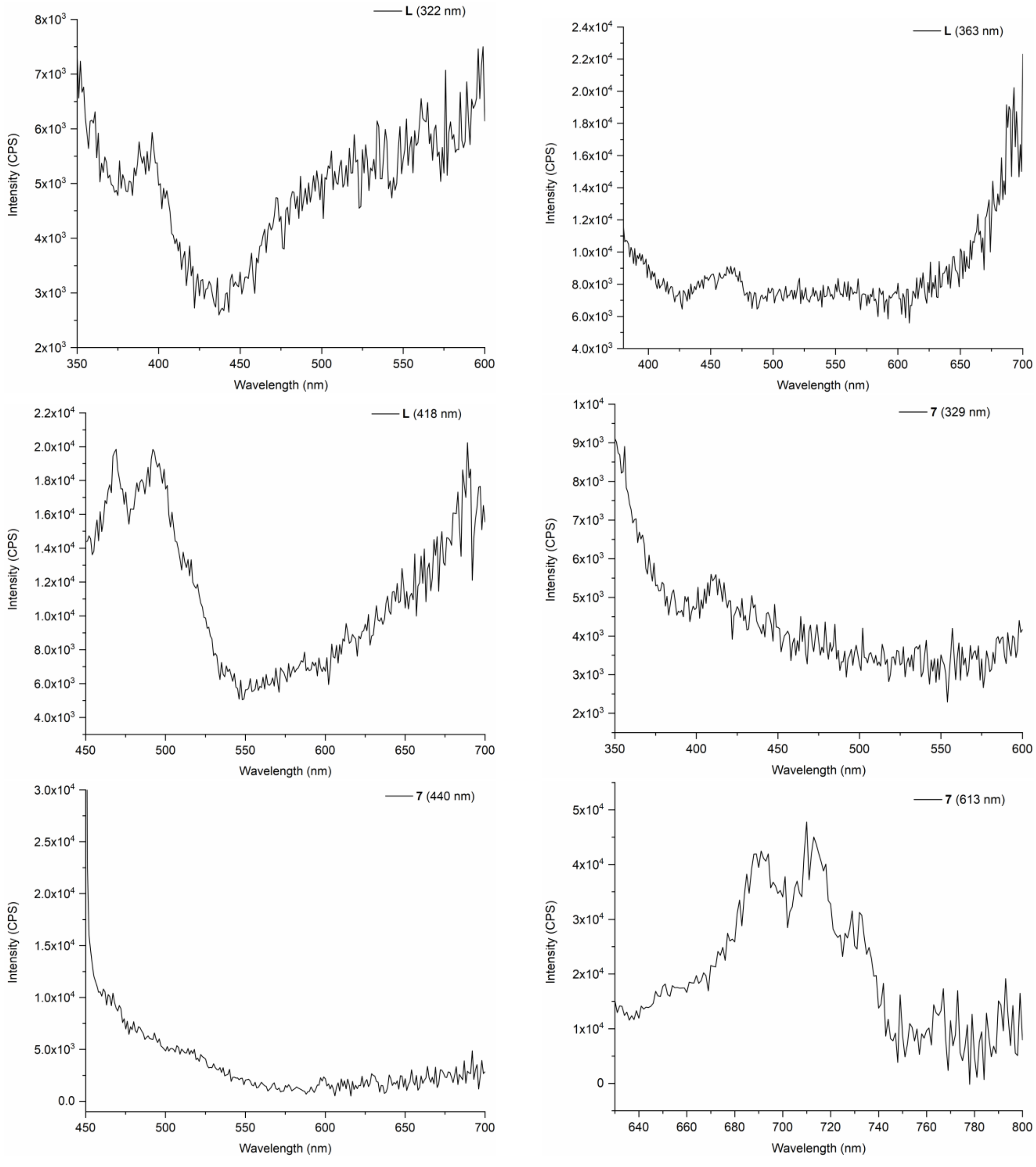


**Figure S115.** PL emission spectra of ligand **L** and complex **7** at different $\lambda_{exc}$.

## REFERENCES


(1) a) Hübschle, C. B.; Sheldrick, G. M.; Dittrich, B. *ShelXle*: a Qt graphical user interface for *SHELXL*. *J. Appl. Crystallogr*. **2011**, *44* (6), 1281-1284. b) Sheldrick, G. M. Crystal structure refinement with *SHELXL*. *Acta Cryst. C* **2015**, *71,* 3-8. c) Brandenburg, K. *Diamond, Crystal and Molecular Structure Visualization Software*, ver. 4.6.0. crystal Impact GbR, Bonn (Germany), **1997-2019**.

(2) CCDC. *Mercury, Crystal Structure Visualization, Exploration and Analysis Software*, ver 4.2.0, The Cambridge Crystallographic Data Centre, **2001-2019**.

(3) Djerdj, I.; Tonejc, A. M. Structural investigations of nanocrystalline $TiO_2$ samples. *J. Alloys Compd.* **2006**, *413* (1-2), 159-174.

(4) Langford, J. I.; Wilson, A. J. C. Scherrer after Sixty Years: A Survey and Some New Results in the Determination of Crystallite Size. *J. Appl. Crystallogr.* **1978**, *11* (2), 102-113.

(5) Karri, R.; Das, R.; Rai, R. K.; Gopalakrishnan, A.; Roy, G. Hg–C bond protonolysis by a functional model of bacterial enzyme organomercurial lyase MerB. *Chem. Commun*. **2020**, *56* (65), 9280-9283.

(6) a) Murphy, A. B. Band-gap determination from diffuse reflectance measurements of semiconductor films, and application to photoelectrochemical water-splitting. *Sol. Energ. Mat. Sol. C.* **2007**, *91* (14), 1326-1337. b) Tirloni, B.; Lang, E. S.; de Oliveira, G. M.; Piquinic, P.; Hörner, M. Synthesis, crystal structure, and optical characteristics of $[Pd_2Hg_4Cl_6\{Te(DMB)\}_6]\cdot 2DMF$, $[HgClTe(DMB)]_4$, and the ring-forming cluster $[Pd_{12}(TePh)_{24}]\cdot 2DMF$. *New J. Chem*. **2014**, *38* (6), 2394-2399. c) Makuła, P.; Pacia, M.; Macyk, W. How To Correctly Determine the Band Gap Energy of Modified Semiconductor Photocatalysts Based on UV−Vis Spectra. *J. Phys. Chem. Lett.* **2018**, *9* (23), 6814-6817.

(7) Jubu, P. R.; Yam, F. K.; Igba, V. M.; Beh, K. P. Tauc-plot scale and extrapolation effect on bandgap estimation from UV–vis–NIR data – A case study of [illegible]-$Ga_2O_3$. *J. Solid State Chem*. **2020**, *290,* 121576.

(8) OriginLab; *OriginPro, Data Analysis and Graphing Software*, ver. 2018, OriginLab Corporation, Northampton (USA), **1991-2017**.

(9) Strohalm, M.; Kavan, D.; Novák, P.; Volný, M.; Havlíček, V. *mMass* 3: A Cross-Platform Software Environment for Precise Analysis of Mass Spectrometric Data. *Anal. Chem.* **2010**, *82* (11), 4648-4651.

(10) a) Gagné, R. R.; Koval, C. A.; Lisensky, G. C. Ferrocene as an Internal Standard for Electrochemical Measurements. *Electrochem. Solid Electrodes* **1980**, *19* (9), 2854-2855. b) Bond, A. M.; Oldham, K. B.; Snook, G. A. Use of the Ferrocene Oxidation Process To Provide Both Reference Electrode Potential Calibration and a Simple Measurement (via Semiintegration) of the Uncompensated Resistance in Cyclic Voltammetric Studies in High-Resistance Organic Solvents. *Anal. Chem*. **2000**, *72* (15), 3492-3496. c) Manfredi, N.; Decavoli, C.; Boldrini, C. L.; Coluccini, C.; Abbotto, A. Ferrocene Derivatives Functionalized with Donor/Acceptor (Hetero)Aromatic Substituents: Tuning of Redox Properties. *Energies* **2020**, *13* (15), 3937.

(11) Peglow, T. J.; Schumacher, R. F.; Cargnelutti, R.; Reis, A. S.; Luchese, C.; Wilhelm, E. A.; Perin, G. Preparation of bis(2-pyridyl) diselenide derivatives: Synthesis of selenazolo[5,4-*b*]pyridines and unsymmetrical diorganyl selenides, and evaluation of antioxidant and anticholinesterasic activities. *Tetrahedron Lett.* **2017**, *58* (38), 3734-3738.

(12) Cargnelutti, R.; da Silva, F. D.; Abram, U.; Lang, E. S. Metal complexes with bis(2-pyridyl)diselenoethers: structural chemistry and catalysis. *New J. Chem*. **2015**, *39* (10), 7948-7953.

(13) Samsudin, E. M.; Hamid, S. B. A.; Juan, J. C.; Basirun, W. J.; Centi, G. Synergetic effects in novel hydrogenated F-doped $TiO_2$ photocatalysts. *Appl. Surf. Sci*. **2016**, *370,* 380-393.
